# Genome-wide association analysis of thirty one production, health, reproduction and body conformation traits in contemporary U.S. Holstein cows

**DOI:** 10.1186/1471-2164-12-408

**Published:** 2011-08-11

**Authors:** John B Cole, George R Wiggans, Li Ma, Tad S Sonstegard, Thomas J Lawlor, Brian A Crooker, Curtis P Van Tassell, Jing Yang, Shengwen Wang, Lakshmi K Matukumalli, Yang Da

**Affiliations:** 1 Animal Improvement Programs Laboratory, Agricultural Research Service, USDA, Beltsville, Maryland, USA; 2 Department of Animal Science, University of Minnesota, St. Paul, Minnesota, USA; 3 Bovine Functional Genomics Laboratory, Agricultural Research Service, USDA, Beltsville, Maryland, USA; 4 Holstein Association USA, Brattleboro, Vermont, USA

## Abstract

**Background:**

Genome-wide association analysis is a powerful tool for annotating phenotypic effects on the genome and knowledge of genes and chromosomal regions associated with dairy phenotypes is useful for genome and gene-based selection. Here, we report results of a genome-wide analysis of predicted transmitting ability (PTA) of 31 production, health, reproduction and body conformation traits in contemporary Holstein cows.

**Results:**

Genome-wide association analysis identified a number of candidate genes and chromosome regions associated with 31 dairy traits in contemporary U.S. Holstein cows. Highly significant genes and chromosome regions include: BTA13's *GNAS *region for milk, fat and protein yields; BTA7's *INSR *region and BTAX's *LOC520057 *and *GRIA3 *for daughter pregnancy rate, somatic cell score and productive life; BTA2's *LRP1B *for somatic cell score; BTA14's *DGAT1-NIBP *region for fat percentage; *BTA1*'s *FKBP2 *for protein yields and percentage, BTA26's *MGMT *and BTA6's *PDGFRA *for protein percentage; BTA18's 53.9-58.7 Mb region for service-sire and daughter calving ease and service-sire stillbirth; BTA18's *PGLYRP1*-*IGFL1 *region for a large number of traits; BTA18's *LOC787057 *for service-sire stillbirth and daughter calving ease; BTA15's *CD82*, BTA23's *DST *and the *MOCS1*-*LRFN2 *region for daughter stillbirth; and BTAX's *LOC520057 *and *GRIA3 *for daughter pregnancy rate. For body conformation traits, BTA11, BTAX, BTA10, BTA5, and BTA26 had the largest concentrations of SNP effects, and *PHKA2 *of BTAX and *REN *of BTA16 had the most significant effects for body size traits. For body shape traits, BTAX, BTA19 and BTA3 were most significant. Udder traits were affected by BTA16, BTA22, BTAX, BTA2, BTA10, BTA11, BTA20, BTA22 and BTA25, teat traits were affected by BTA6, BTA7, BTA9, BTA16, BTA11, BTA26 and BTA17, and feet/legs traits were affected by BTA11, BTA13, BTA18, BTA20, and BTA26.

**Conclusions:**

Genome-wide association analysis identified a number of genes and chromosome regions associated with 31 production, health, reproduction and body conformation traits in contemporary Holstein cows. The results provide useful information for annotating phenotypic effects on the dairy genome and for building consensus of dairy QTL effects.

## Background

Genome-wide association studies (GWAS) using single nucleotide polymorphism (SNP) markers provide a powerful approach for annotating phenotypic effects or mapping QTL of important dairy traits on the genome. Dense genome coverage allows detection of QTLs with greater accuracy than was previously possible [1-3]. Combined with bovine whole-genome sequence information [4,5], many SNP effects can be readily localized to specific genes or gene regions. Such QTL detection provides valuable information for understanding genetic mechanisms underlying dairy phenotypes and for identifying causal polymorphisms that lead to more rapid genetic improvement using genome selection [6,7] or gene-based selection [8]. Several dairy GWAS using the bovine 50 k SNP chip [1-3] have been reported, including a study of U.S. Holstein bulls for 27 dairy traits that focused on the size and distribution of QTL effects [9], a study of milk traits in Danish Jersey bulls [10], a study of fertility traits in Danish and Swedish Holstein bulls [11], and a genome-wide candidate gene study using 1,536 SNP markers of candidate genes of Canadian Holstein bulls for association analysis with 17 type and functional traits [12]. These genome-wide studies contributed considerable new information over the many QTL studies based on microsatellite markers as compiled at the Cattle QTL Database [13] and contribute towards building a consensus on dairy QTL effects.

In this study, we conducted genome-wide association analysis of 31 production, health, reproduction and body conformation traits of contemporary U.S. Holstein cows. We used the bovine 50 k SNP panel to identify SNP markers, genes and chromosome regions on the 29 bovine autosomes and the X chromosome associated with these 31 economically important dairy traits. Production, health and reproduction are fundamental dairy functions while body conformation (type) traits are related to functionality of the cow's body and are related to value of the cow as a show animal.

## Results

### Overview of SNP effects

**Figure 1:**
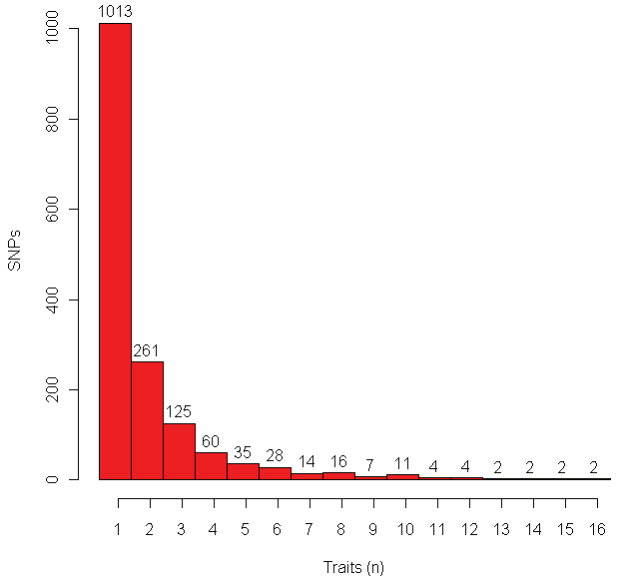
**Distribution of 1,586 SNPs by number of traits affected for each of 31 production, health, reproduction and body conformation traits of contemporary U.S. Holsteins**.

A global view of all additive SNP effects for each trait is presented in Manhattan plots [14,15] in Figure S1 (Additional file 1), which shows that a large number of additive SNP effects reached 5% genome-wide significance with the Bonferroni correction (*P *value < 10^-6.4^) for each trait. Therefore, only the top 100 effects for each trait are reported for a total of 3,100 effects of 1,586 SNPs with 573 (36%) in 486 genes based on the Btau_4.0 and UMD 3.0 genome assemblies. Of the 45,878 SNPs that were genotyped, 16,516 (36%) were in 7,434 genes. The majority of the 1,586 SNPs each affected one trait, whereas 27 SNPs each affected 10 or more traits (Figure 1). All SNP effects in this report were additive as was expected because PTAs predict only additive genetic merit. Genotypic effects for SNPs had nearly identical *P *values to those of additive SNP effects. Therefore, *P *values of additive effects were used to rank SNP markers for each trait. Permutation tests of all 45,878 SNPs using 1,000 permutes and the cutoff *P *value for the top 100 most significant effects for each trait produced no observed false positive effects.

Detailed test results of the 3,100 effects are given in Table S1 (Additional File 2); complete QTL maps are shown in Figure S2 (Additional file 3) for the 13 production, health and reproduction traits, and are shown in Figure S3 (Additional File 4) for the 18 body conformation traits. Detailed characterization of the top 20 effects of each trait are given in Table S2 (Additional File 5), including actual SNP alleles, UMD 3.0 and Btau_4.0 SNP positions, the favorable allele, the favorable allele frequency, gene or gene region of the SNP, observed P-value, and the effect size with standard deviation.

The 3,100 effects of the 1,586 SNPs were distributed over all 29 *Bos taurus *(BTA) autosomes and the X chromosome but the distribution was uneven, with certain chromosomes having large numbers of SNP effects, and different chromosomes generally were associated with different traits (Table 1, Table 2). In Table 2, the 18 body conformation traits were divided into six trait groups: body size, body shape, udder, teats, feet and legs, and final score. "Body size" includes four traits; stature, strength, body depth, and rump width. "Body shape" includes two traits; dairy form and rump angle. "Udder" includes four traits; fore udder attachment, rear udder height, udder depth, and udder cleft. "Teats' includes three traits; front teat placement, rear teat placement and teat length. "Feet and legs" includes four traits; foot angle, rear legs (side view), rear legs (rear view) and feet/legs score. Definition and graphical illustration of each body conformation trait are available online [16,17]. Chromosomes with a large number of effects for a trait did not necessarily have the most significant effect associated with that trait. For example, BTA18 did not have the largest number of SNP effects for any trait but did have a SNP (BFGL-NGS-117985) that had the most significant effect for five traits (Additional File 2: Table S1).

**Table 1:** Distribution of the top 100 most significant SNP effects for predicted transmitting abilities for 13 production, health, and reproduction traits of contemporary U.S. Holsteins by chromosome.

Chr	MY	FY	PY	FPC	PPC	PL	SCS	DPR	SCE	DCE	SSB	DSB	NM	All traits
1	2	4	4	4	5	**15**	4	**17**	7	6	7	3	**15**	**93**
2	3	0	0	0	1	1	**11**	2	0	0	0	2	0	**20**
3	**10**	4	1	4	1	9	7	**17**	4	0	4	6	1	**68**
4	3	2	2	0	0	4	0	0	2	0	3	0	0	**16**
5	2	2	2	7	**13**	0	4	0	7	3	**10**	0	2	**52**
6	2	2	5	1	6	2	9	0	0	6	0	1	5	**39**
7	0	6	1	1	2	**11**	**13**	**15**	4	**11**	0	6	4	**74**
8	2	1	0	0	0	4	1	3	0	0	0	3	1	**15**
9	7	6	1	1	2	0	0	0	4	3	1	6	4	**35**
10	5	0	0	0	3	0	2	6	1	0	4	0	0	**21**
11	3	3	3	1	3	0	1	0	1	0	0	5	0	**20**
12	2	1	1	2	3	0	6	0	8	2	6	1	1	**33**
13	**13**	**12**	8	4	1	0	1	2	1	0	0	7	4	**53**
14	2	1	1	**24**	2	0	0	2	0	0	6	4	0	**42**
15	1	2	1	2	0	1	0	0	1	1	4	5	1	**19**
16	1	0	0	0	0	2	**11**	1	0	0	3	3	0	**21**
17	4	7	6	**11**	**14**	9	1	5	**20**	**21**	7	2	**14**	**121**
18	8	4	6	5	4	8	2	5	9	**12**	6	4	**10**	**83**
19	2	3	2	0	0	0	0	0	0	0	0	3	0	**10**
20	0	2	**11**	0	2	0	2	0	1	1	1	5	0	**25**
21	4	1	5	0	5	0	0	0	2	1	6	5	1	**30**
22	1	0	0	0	0	3	2	3	0	1	0	2	1	**13**
23	5	5	9	2	3	0	1	0	2	1	1	**16**	3	**48**
24	2	1	2	2	2	1	1	0	1	1	3	0	2	**18**
25	0	2	0	3	0	0	5	1	0	0	1	0	0	**12**
26	0	7	6	6	**10**	9	3	4	6	**10**	9	1	**11**	**82**
27	5	6	7	0	1	1	1	0	3	1	0	1	3	**29**
28	0	0	5	0	1	2	0	3	3	1	1	1	3	**20**
29	0	0	0	0	0	1	2	1	0	2	1	3	1	**11**
X	**11**	**16**	**11**	**19**	**16**	**15**	9	**12**	**12**	**16**	**15**	3	**12**	**167**
U	0	0	0	1	0	2	1	1	1	0	1	2	1	**10**
P*	10^-12^	10^-24^	10^-23^	10^-17^	10^-22^	10^-29^	10^-23^	10^-25^	10^-38^	10^-23^	10^-24^	10^-21^	10^-34^	
R^2^	0.42	0.43	0.42	0.45	0.40	0.54	0.54	0.53	0.52	0.42	0.56	0.55	0.49	

SNP, single nucleotide polymorphism; Chr, chromosome; MY, milk yield; FY, fat yield; PY, protein yield; FPC, fat percentage; PPC, protein percentage; PL, productive life; SCS, somatic cell score; DPR, daughter pregnancy rate; SCE, service-sire calving ease; DCE, daughter calving ease; SSB, service-sire stillbirth; DSB, daughter stillbirth; NM, net merit; U, unknown. Boldface indicates ≥10 significant SNP effects. *Rounded cutoff for *P *value for top 100 most significant SNPs for trait. R^2^, variation accounted for by the top 100 SNPs for the trait.

**Table 2:** Distribution of the top 100 most significant SNP effects for predicted transmitting abilities for 18 conformation traits of contemporary U.S. Holsteins by chromosome.

Chr	STA	STR	BD	RW	DF	RA	FUA	RUH	UD	UC	FTP	RTP	TL	FA	RLS	RLR	FL	FS	All traits
1	3	3	3	1	5	2	3	5	5	4	2	7	8	3	**12**	1	2	3	**72**
2	1	1	1	1	4	5	3	**11**	1	5	6	**10**	2	0	6	7	5	3	**72**
3	1	4	0	1	**10**	2	1	1	3	3	1	2	2	0	7	0	0	0	**38**
4	3	3	0	1	0	1	1	1	1	0	8	1	7	1	0	4	2	2	**36**
5	8	6	**14**	5	0	**12**	7	4	7	0	4	1	6	7	4	3	**11**	9	**108**
6	0	6	3	**10**	0	7	9	5	2	8	**14**	5	4	5	2	3	5	8	**96**
7	2	2	0	0	**11**	0	5	5	5	**13**	7	7	2	0	3	0	0	5	**67**
8	1	1	1	1	6	**16**	0	0	0	4	1	1	1	1	1	0	1	0	**36**
9	1	0	1	0	0	**14**	2	0	1	1	7	5	0	3	0	0	0	0	**35**
10	7	1	**10**	**14**	**19**	4	**12**	**21**	4	3	2	4	0	0	1	0	0	**16**	**118**
11	**30**	**13**	**23**	2	6	2	4	**15**	2	5	0	4	**13**	4	1	**32**	**11**	**16**	**183**
12	3	0	0	0	1	3	0	0	3	3	1	3	5	0	4	0	1	3	**30**
13	3	**13**	9	5	2	1	4	1	3	1	4	3	1	8	1	4	9	1	**73**
14	0	0	1	0	5	0	0	1	0	3	1	0	0	1	2	1	2	0	**17**
15	1	2	1	2	0	2	1	0	3	6	0	5	0	2	0	0	1	0	**26**
16	1	3	4	8	5	2	7	2	4	1	8	7	3	2	**10**	2	1	3	**73**
17	1	1	0	0	3	0	2	0	6	1	2	1	**15**	**15**	6	1	5	0	**59**
18	0	0	0	0	0	3	0	0	1	1	0	2	3	7	8	1	2	0	**28**
19	3	2	2	**10**	1	1	7	3	4	8	4	5	0	0	0	1	0	5	**56**
20	7	5	4	1	0	0	7	3	7	1	4	3	0	**10**	1	9	3	2	**67**
21	1	0	0	2	0	0	2	1	9	4	5	1	9	2	3	1	2	1	**43**
22	1	2	2	2	2	5	2	1	3	1	3	1	2	1	4	2	4	3	**41**
23	2	1	1	1	1	1	2	2	2	2	4	1	0	0	1	0	0	2	**23**
24	0	0	0	0	2	0	0	0	2	1	1	2	3	1	4	0	0	0	**16**
25	0	2	2	0	2	0	1	0	3	1	1	1	0	2	0	1	1	1	**18**
26	3	7	2	4	2	2	8	4	**11**	1	1	1	4	**13**	5	9	**16**	5	**98**
27	0	0	0	0	1	0	1	0	2	**10**	0	6	0	0	3	0	1	0	**24**
28	1	0	0	0	0	0	1	2	0	0	4	1	0	0	0	0	1	1	**11**
29	0	0	0	1	0	4	0	0	0	1	1	1	0	2	1	0	1	0	**12**
X	**14**	**22**	**15**	**27**	**12**	9	8	**10**	6	7	2	9	**10**	**10**	7	**18**	**12**	**10**	**208**
U	2	0	1	1	0	2	0	2	0	1	2	0	0	0	3	0	1	1	**16**
P*	10^-21^	10^-20^	10^-18^	10^-18^	10^-20^	10^-11^	10^-18^	10^-16^	10^-19^	10^-18^	10^-12^	10^-12^	10^-13^	10^-20^	10^-15^	10^-17^	10^-17^	10^-19^	
R^2^	0.56	0.49	0.48	0.53	0.45	0.40	0.56	0.50	0.55	0.52	0.48	0.46	0.39	0.52	0.38	0.47	0.49	0.56	

SNP, single nucleotide polymorphism; Chr, chromosome; STA, stature; STR, strength; BD, body depth; RW, rump width; DF, dairy form; RA, rump angle; FUA, fore udder attachment; RUH, rear udder height; UD, udder depth; UC, udder cleft; FTP, front teat placement; RTP, rear teat placement; TL, teat length; FA, foot angle; RLS, rear legs (side view); RLR, rear legs (rear view); FL, feet/legs score; FS, final score; U, unknown. Boldface indicates ≥10 significant SNP effects. *Rounded cutoff for *P *value for top 100 most significant SNPs for trait. R^2^, variation accounted for by the top 100 SNPs for the trait.

The top 100 SNPs for each trait accounted for 38% to 56% of the PTA variation (Table 1, Table 2). Significance levels varied by trait. Among production, health and reproduction traits, sire calving ease had the highest significance (smallest cutoff for *P *value; *P *< 10^-38^) and milk yield had the lowest significance (*P *< 10^-12^) (Table 1). For body conformation traits, stature had the highest significance (*P *< 10^-21^) and rump angle had the lowest significance (*P *< 10^-11^) (Table 2).

### SNP effects in gene clusters, localized effect concentrations, highly significant genes

**Figure 2:**
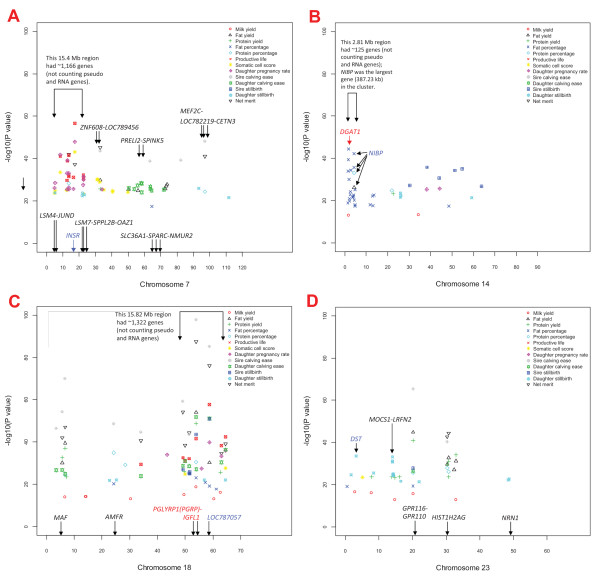
**Map of SNP position (Mb) on *Bos taurus *chromosomes 7 (A), 14 (B), 18 (C), and 23 (D) by *P*-value for 725 SNPs that comprise the top 100 effects for each of 13 production, health, and reproduction traits of contemporary U.S. Holsteins**.

**Figure 3:**
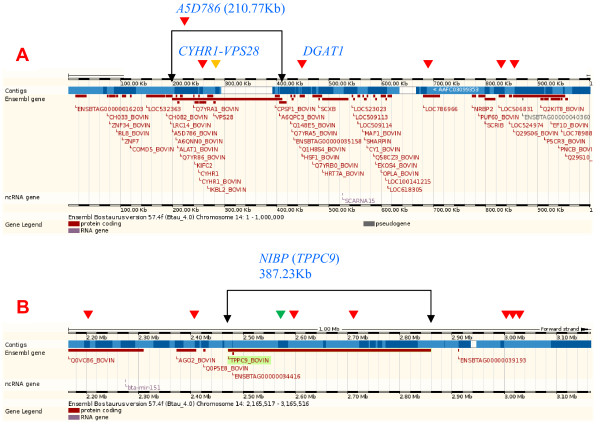
**A 2.81 Mb gene cluster on *Bos taurus *chromosome 14 that was associated with significant effects of single nucleotide polymorphisms for fat percentage. Red arrow, fat percentage effect; gold arrow, milk yield effect; green arrow, fat yield effect**. **A**) 1 Mb region with *A5D786*, *CYHR1*, *VPS28*, and *DGAT1 *genes; *A5D786 *was second largest gene in the cluster and contained *CYHR1 *and *VPS28*. **B**) 1 Mb region with *NIBP*, the largest gene in the cluster.

SNP effects for daughter pregnancy rate, somatic cell score and productive life overlapped with a large gene cluster of approximately 1,166 genes in a 15.4 Mb region of BTA7 (Figure 2A; Additional File 6: Figure S4A). The *insulin receptor *(*INSR*) gene in this cluster was 1.5 kb from the SNP with the most significant effect on somatic cell score and daughter pregnancy rate, and was third most significant for productive life. A narrow 2.81 Mb region of BTA14 with approximately 125 genes (Figure 2B; Additional File 6: Figure S4B) had 19 SNP effects for fat percentage, one SNP effect for milk yield associated with the *vacuolar protein sorting 2 homolog *(*VPS28*) gene, and one SNP effect each for fat yield and protein percentage in the *NIK and IKKβ binding protein *(*NIBP*) gene. A SNP in the *diacylglycerol O-acyltransferase homolog 1 *(*DGAT1*) gene (Figure 3A) had the most significant effect for fat percentage followed by a SNP in *NIBP*, which was the largest (387.23 kb) gene in this cluster (Figure 3B). The 15.82 Mb region of BTA18 with approximately 1,322 genes (Figure 2C; Additional File 6: Figure S4C) had SNP effects for many traits but was most pronounced for service-sire and daughter calving ease and service-sire stillbirth. The *peptidoglycan recognition protein 1 *(NCBI's *PGLYRP1 *or ENSEMBL's *PGRP*) gene and *IGF-like family member 1 *(*IGFL1*) gene in this cluster flanked a SNP that was highly significant for many traits. This SNP had the top effect for fat and protein yields, service-sire and daughter calving ease, and net merit; the eighth most significant effect for milk yield and service-sire stillbirth; the 16th most significant effect for productive life, and the 25th most significant effect for fat and protein percentages. The *zinc finger protein 415-like *gene (*LOC787057*) was most significant for service-sire stillbirth and second most significant for daughter calving ease. The 211.67 kb *MOCS1*-*LRFN2 *region of BTA23 (Figure 2D; Additional File 7: Figure S5A) included several SNPs with significant effects on daughter stillbirth, while a BTA15 marker between two *CD82 *genes had the most significant effect for daughter stillbirth (Additional File 7: Figure S5B). The *MOCS1 *gene is related to early infant death in humans [18,19]. The most significant effect for milk yield was near the *guanine nucleotide binding protein, alpha stimulating *(*GNAS*) locus of BTA13 (Additional File 7: Figure S5C).

Additional gene clusters with SNP effects were also observed on BTA3, BTA5, BTA7, BTA10, BTA17, BTA21, BTA23, BTA26, BTA29, and BTAX (Additional File 6: Figure S4D-N). Additional localized concentrations of SNP effects were also observed at 45 Mb on BTA3 and from 1 to 15 Mb on BTA13 for milk yield, at 135 Mb on BTA1 and at 91 Mb on BTA3 for daughter pregnancy rate, at 14 and 34 Mb on BTA17 for daughter calving ease, and at 49.5 Mb on BTA26 for fat yield, fat and protein percentages, and daughter calving ease.

**Figure 4:**
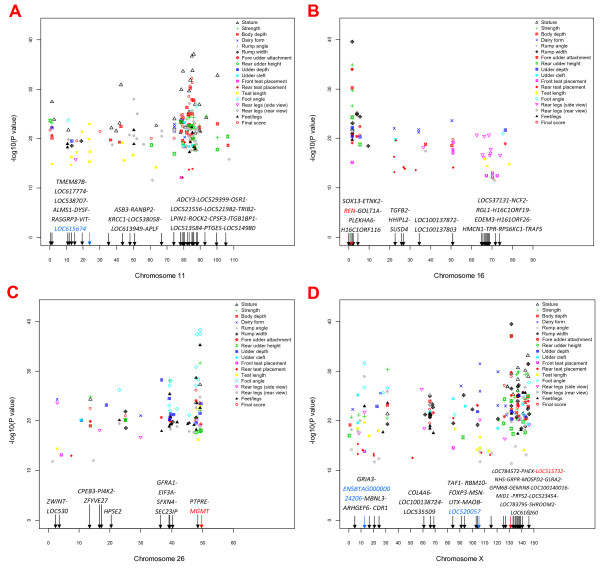
**Map of SNP position (Mb) on *Bos taurus *chromosomes 11 (A), 16 (B), 26 (C) and X (D) by *P*-value for 1,005 SNPs that comprise the top 100 effects for each of 18 conformation traits of contemporary U.S. Holsteins**.

For body conformation traits, BTAX's *phosphorylase kinase, alpha 2 (liver) *gene (*PHKA2*) was highly significant for body size traits. The top SNP effects in the 80 to 90 Mb region of BTA11 predominantly affected stature, strength, body depth, rear udder height, teat length, rear legs (rear view), feet/legs score, and final score (Figure 4A). The *renin *gene (*REN*) at the top telomere region of BTA16 was highly significant for 12 traits (the largest number of conformation traits affected by one gene). The 65 to 75 Mb region of BTA16 (Figure 4B) had a concentration of SNP effects for rear legs (side view). The 39 Mb region and the *O-6-methylguanine-DNA methyltransferase *(*MGMT*) gene region on BTA26 (Figure 4C) had an effect concentration for feet/legs traits. The 125 to 145 Mb region of BTAX (Figure 4D) had significant effects for body size traits, foot angle, rear legs (rear view), and final score. Other chromosomes with local effect concentrations included the 30 to 35 Mb region of BTA2 for dairy form, rear udder height, and feet/legs score; the 19 to 21 Mb region of BTA13 for foot angle and feet/legs score; the 56.2 to 57.6 Mb region of BTA19 for rump width; the 10 to 20 Mb region of BTA20 for udder depth and foot angle; and the 20 to 22 Mb region of BTA21 for teat length.

For the 31 traits, the X chromosome had the largest number of SNP effects (Table 1, Table 2) and most of these effects were in four regions; 5 to 25 Mb, 50 to 60 Mb, 85 to 105 Mb, and 125 to 140 Mb (Additional File 3: Figure S2; Additional File 4: Figure S3). The most significant X chromosome SNP effects were associated with daughter pregnancy rate, body size, dairy form, rear legs (rear view), productive life, udder attachment, rear teat placement, fat yield, protein yield, net merit, and final score of body conformation traits (Additional file 2: Table S1). Eighteen significant SNP markers were in the pseudo-autosomal region defined by UMD 3.0. However, based on male heterozygosity, only eight of the 18 markers could be truly in the pseudo-autosome region. Male heterozygosity for ten of these 18 SNPs (marked in yellow in Additional file 2: Table S1) were inconsistent with the pseudo-autosomal assignments because they had zero or a small number of heterozygous genotypes among 483 males (not included in this report). The region from 140,525,988 bp to 143,832,372 bp had 28 markers. Of these, one marker had three male heterozygous genotypes, two markers each had one male heterozygous genotype, and the other 25 markers had no male heterozygous genotypes. Similarly, of the 8 markers in the region from 139,306,649 bp to 139,975,594 bp, only one marker had 15 male heterozygous genotypes while the other seven markers had no male heterozygous genotype.

The second largest number of SNP effects for production, health and reproduction traits (Table 1) were in the 10 to 35 Mb region of BTA17 (Figure S1). These included SNP effects for daughter calving ease, sire calving ease and protein percentage, but most of the effects fell in gene-sparse areas.

### Summary of SNP effects by trait

The results in this study generally point to polygenic genetic mechanisms for all 31 dairy traits. However, some chromosome regions and genes had more striking association(s) with the traits in terms of statistical significance and known relevant biology, making these regions and genes more likely candidates for causal effects. The following is a brief summary of such regions and genes.

#### Milk, fat and protein yields

Milk, fat and protein yields had a tendency of sharing common SNP effects. The 58 Mb region of BTA13 with four genes near *GNAS *was highly significant for the three yield traits (Additional File 5: Table S2.1 to S2.3). A BTA1 region 220 kb upstream of *FKBP2*, which plays a role in immunoregulation and basic cellular processes involving protein folding and trafficking, was highly significant for fat yield, protein yield and protein percentage. The 47 Mb region of BTA3 had a strong effect for milk yield, while the 54 Mb region of BTA18 near *PGLYRP1-IGFL1 *and the 146 Mb X chromosome region in the *DOCK11*-*IL13RA1*-*AF074402*-*LOC616260 *gene cluster could be targets for fat and protein yields.

#### Fat and protein percentages

This study specifically identified a 2.81 Mb BTA14 gene cluster spanning *DGAT1*-*NIBP *(Figure 2B, Figure 3) as a region with a heavy concentration of SNP effects for fat percentage, accounting for 8% of the PTA variation. Within this cluster, *DGAT1 *had the most significant and *NIBP *had the second most significant SNP effects for fat percentage (Additional File 5: Table S2.4). The *VPS28 *gene, which is about 100 kb upstream of *DGAT1 *and is near the left end of the 2.81 Mb *DGAT1*-*NIBP *region, had a highly significant effect for milk yield, while *NIBP*, which is at the right end of this 2.81 Mb region, had a SNP at position 4.468 Mb (2.71 Mb according to Btau_4.0) with a highly significant effect for fat yield, fat percentage and protein percentage (Figure 3). The 49 Mb BTA26 region near *MGMT *had highly significant effects on protein percentage as well as protein yield, and the 71 Mb BTA6 region near *PDGFRA *included highly significant SNPs affecting protein percentage (Additional File 5: Table S2.5).

#### Productive life, somatic cell score, daughter pregnancy rate

These three traits had two common regions and some trait-specific regions. The first common region for these three traits was the 15.4 Mb BTA7 gene cluster of approximately 1,166 genes (Figure 2A). Although the exact location of the causal effect may be difficult to dissect for such a tightly linked gene cluster (7.5 genes per 100 kb), our data placed the most significant effect in this region near *INSR *(Additional File 5: Table S2.6 to S2.8; Figure 2A; Additional File 6: Figure S4A). The second common region was the 106 Mb BTAX region near *LOC520057 *(similar to *type 1 protein phosphatase inhibitor*). Trait-specific regions include the *MIR2353-STK39 *and *LRP1B *(*low density lipoprotein-related protein 1B) *regions of BTA2 for somatic cell score, the 129 to 141 Mb region of BTA1, the 90 Mb region of BTA3 for daughter pregnancy rate, and the *ATP1B4 *(*ATPase, Na+/K+ transporting, beta 4 polypeptide*) and *GRIA3 *(g*lutamate receptor 3*) genes on BTAX for daughter pregnancy rate.

Productive life measures the cow's longevity in the herd and is affected by production, health and reproduction. Somatic cell score is a measure of udder health and daughter pregnancy rate is a measure of cow fertility. The fact that productive life shared many common SNP effects with somatic cell scores and daughter pregnancy rate and did not share many SNP effects with production and calving traits indicates productive life was more genetically related with health and fertility traits than with production and calving traits.

#### Service-sire calving ease, daughter calving ease, service-sire stillbirth

These three traits shared a common region on BTA18 in the 15.82 Mb gene cluster with approximately 1,322 genes (Figure 2C; Additional File 6: Figure S4C). The *PGLYRP1*-*IGFL1 *region and *LOC787057 *had the most significant effects for these three traits (Additional File 5: Table S2.9-S2.11).

#### Daughter stillbirth

Nine of ten SNPs in the 211.67 kb *MOCS1*-*LRFN2 *region of BTA23 were among the top 100 SNPs that were significant for daughter stillbirth (Figure 2D), which is a measure of the cow's effect on calf stillbirth. The SNP marker between two *CD82 *genes on BTA15 (Additional File 2: Figure S1**; **Additional File 5: Table S2.12) had the most significant effect. The second most significant SNP effect was in the *dystonin *gene (*DST*) on BTA23.

#### Lifetime net merit index

Highly significant chromosome regions and genes for this composite trait involved the significant regions and genes for milk, fat and protein yields, protein percentage, service sire calving ease, daughter calving ease, and service sire stillbirth (Additional File 5: Table S2.13).

#### Body size traits - stature, strength, body depth, rump width

Stature and body depth shared many common SNP markers on BTAX, BTA11 and BTA5, while strength and rump width shared common SNP effects on BTAX and BTA16 (Additional File 5: Table S2.14 to S2.17). The *PHKA2 *gene on BTAX (Additional File 8: Figure S6A) was highly significant for all four body size traits, and the BTAX region from the *gem (nuclear organelle) associated protein 8 *gene (*GEMIN8*) to the *glycoprotein M6B *gene (*GPM6B*) (Figure 4D) included SNPs among the top 20 effects for all four body size traits.

The largest numbers of significant SNP effects for stature were found on BTA11 and BTAX, with 30 and 14 effects, respectively (Table 1). Genes on BTA11 that included or were near SNPs with the top 20 effects were *lipin 1 *(*LPIN1*; second most significant SNP effect), *tribbles homolog 2 *(*TRIB2*; third and fourth SNP effects), *odd-skipped related 1 *(*OSR1*), *aromatic-preferring amino acid transporter-like *(*LOC529399*), and *neuroblastoma amplified sequence *(*LOC521982*) (Additional File 5: Table S2.14; Additional File 8: Figure S6B).

The *REN *gene on BTA16 was highly significant for body strength (ranked #2), depth (ranked #4) and rump width (ranked #1). This gene was located in the middle of a 70-kb gene cluster with five tightly linked genes: *SRY (sex determining region Y)-box 13 *(*SOX13*), *ethanolamine kinase 2 *(*ETNK2*), *REN*, *KiSS-1 metastasis-suppressor *(*KISS1*), and *golgi transport 1A *(*GOLT1A*) (Additional File 8: Figure S6C). The *pleckstrin homology domain containing, family A member 6 *(*PLEKHA6*) gene was 70 kb downstream of these five tightly linked genes and had the ninth most significant SNP effect for strength.

The most significant BTA13 SNP effect for strength was ranked seventh for the trait and was in the *pitrilysin metallopeptidase 1 *gene (*PITRM1*), which also was linked to two of the top 20 effects for stature and three of the top 20 effects for body depth. A SNP on BTA23 at position 18,197,600 bp had the second most significant effect for body depth and was the third most significant for strength.

Strength and rump width are related to body width but likely involved different genes because <25% of the top 100 SNPs (Additional File 2: Table S1) and only four of the top 20 SNPs were in common for the two traits. In addition, none of the significant SNP effects on BTA19 for strength were among the top 20 for rump width. Of the seven BTA19 SNPs among the top 20 for rump width, five were within genes; *GPRC5C *(ranked 3rd), *RNF157 *(ranked 5th), *SRP68 *(ranked 8th), *LOC789539 *(ranked 11th), and *OTOP3 *(ranked 15th). The six most significant BTA19 SNPs were in a 1.8 Mb region of 57.2 to 59.0 Mb (Additional File 8: Figure S6D).

#### Body shape traits - dairy form and rump angle

Although both dairy form and rump angle are measures of the cow's body shape, these two traits likely involved different genes. The top 20 SNP effects for dairy form involved eight chromosomes (Additional File 5: Table S2.18). The most significant SNP for dairy form was 30.9 kb upstream of *LOC520059*, the same SNP that was highly significant for daughter pregnancy rate and productive life. The top 20 effects for dairy form only had four SNPs in genes; *immunoglobulin superfamily, member 5 *(*LOC511594*) of BTA1, *myocyte enhancer factor 2C *(*MEF2C*) of BTA7, and *phospholipase A2, group IVF *(*PLA2G4F*) and *calpain 3 (p94) *(*CAPN3*) of BTA10. For rump angle, BTA5 and BTA9 had the most significant effects (Additional File 5: Table S2.19). Two of the top 20 effects for rump angle were SNPs located in the *NADPH oxidase 4 *(*NOX*) and *SH3 and multiple ankyrin repeat domains 2 *genes (*LOC618649*) on BTA29.

#### Udder traits - fore udder attachment, rear udder height, udder depth, udder cleft

Fore udder attachment shared some common SNP effects with body size traits and udder height, but essentially had no common SNP effects with udder depth and udder cleft (Additional File 5: Table S2.20-S2.23). Fore udder attachment also shared some effects with teat traits, daughter pregnancy rate, somatic cell score and productive life. A SNP in *REN *of BTA16 and a SNP in *PHKA2 *of BTAX were most significant for udder attachment. Four SNPs in the BTA19 region associated with rump width were among the top 20 effects for fore udder attachment. Two BTA2 SNPs that were 19.2 to 58.3 kb upstream of the *T-box, brain, 1 *(*TBR1*) gene had the first and fourth most significant effects, and two BTA10 SNPs in the *adenomatous polyposis coli *(*APC*) and *CAPN3 *genes had the second and third most significant effects, respectively. The most significant effects for udder depth were located on BTA25, BTA22, BTA7, BTA26, BTA20 and BTA23. The SNP 1.5 kb downstream of *INSR *on BTA7 was the tenth most significant SNP for udder depth (Additional File 5: Table S2.23). The most significant effects for udder cleft were located on BTA22, BTA7, and BTA25. Six of the top 20 SNP effects for udder cleft (Additional File 5: Table S2.24) were found on BTA7. Two of the top 20 effects for udder cleft were BTA6 SNPs in the *leucine zipper-EF-hand containing transmembrane protein 1 *(*LETM1*) and *Wolf-Hirschhorn syndrome candidate 2 *(*WHSC2*) genes. The same BTA6 and BTA7 SNP markers were also highly significant for teat placement traits, which indicated that udder cleft and teat placement involved some common genes. The tenth most significant SNP for udder cleft was on BTA19 SNP and was just downstream from a gene cluster that affected rump width and fore udder attachment (Additional File 8: Figure S6D).

#### Teat traits - front teat placement, rear teat placement, teat length

Front and rear teat placements involved different and common SNP effects. Teat length and teat placement traits appeared to have been associated with different genes. Two BTA6 SNPs in the *LETM1 *and *WD repeat and FYVE domain containing 3 *(*WDFY3*) genes were the top two most significant SNPs for front teat placement and were among the top 20 effects for rear teat placement (Additional File 5: Table S2.25 to S2.27). The *LETM1 *SNP was also ranked sixth in significance for udder cleft. A relatively gene-sparse region of BTA7, 347.5-412.1 kb upstream from the *centrin EF-hand protein 3 *gene (*CETN3*), was highly significant for both rear teat placement and udder cleft. The *TAF1 RNA polymerase II, TATA box binding protein-associated factor, 250 kDa *gene (*TAF1*) on BTAX had the second most significant SNP effect for rear teat placement and the 16th for udder cleft. The *GPRC5C *gene on BTA19 (Additional File 8: Figure S6D) had the tenth most significant SNP for rear teat placement and the second for udder cleft. These results indicate that the same chromosome regions were involved in rear teat placement and udder cleft and that the *LETM1 *and *WHSC2 *genes on BTA6 had a major role in udder cleft and teat placement traits. The most significant SNP effect for teat length was on BTA11, 98.5 kb downstream from *LOC615674*, a *ribosomal protein L36-like *gene, followed by a BTA26 SNP 80.8 kb upstream from *MGMT*. The three BTA21 SNPs among the top 20 effects for teat length were in a gene cluster (Additional File 8: Figure S6E), with one SNP in the hypothetical protein *LOC613997 *(*MGC129355*; ENSEMBL *CO038*) and one SNP in the *abhydrolase domain containing 2 *gene (*ABHD2*).

#### Feet/legs traits - foot angle, rear legs (side view), rear legs (rear view), feet/legs score

Three BTA26 SNPs that spanned a 1.09 Mb region in or upstream from *MGMT *had the top three effects for foot angle, and another four BTA26 SNPs were also among the top 20 effects for foot angle (Additional File 5: Table S2.28). BTA1 had the most significant SNP for rear legs (side view), whereas BTA18 had the largest number of significant SNPs (five effects), followed by BTA1, BTA16, and BTAX with three effects each (Additional File 5: Table S2.29). The top 20 effects for rear legs (rear view) involved only four chromosomes: BTA11, BTAX, BTA20, and BTA26. The most significant SNP was on BTAX, followed by three BTA11 SNPs. The most significant SNP for foot angle and for feet/legs score was in *MGMT *on BTA26 (Additional File 5: Table S2.27, Table S2.30). This SNP was the tenth most significant SNP for rear legs (rear view). The side and rear views of the legs apparently were associated with different sets of chromosome and gene regions. Of the top 20 effects, BTA26 and BTA12 had the most SNPs (five each), followed by BTA5 and BTAX (four each). The top 20 SNP effects for feet/legs score were predominantly the same as those for foot angle and rear legs (rear view).

#### Final score (also known as PTA type)

The most significant SNP for final score (Additional File 5: Table S2.31) was a BTAX SNP in *PHKA2*, which was also the most significant SNP for stature, strength, and body depth, the second most significant for rump width and fore udder attachment, and the 11th most significant for rear udder height. The second most significant SNP for final score was in BTA16's *REN*, which was among the top 20 effects for five other conformation traits. The third most significant SNP for final score was in BTA10's *APC*, which was second most significant for rear udder height, eighth for fore udder attachment, and ninth for rump width.

## Discussion

### Comparison with reported results

Most previously reported QTL locations were based on genetic distances in units of Morgan (or centiMorgan) rather than the physical distances (Mb or kb) for QTL locations reported in this study. Because exact and complete translation of bovine genetic distances into physical distances is not available, the results in this study could be compared only with studies that reported genes or markers with known physical locations.

This study confirmed widely reported findings that the BTA14 region containing *DGAT1 *was important for fat percentage [20] and provided some evidence of QTL effects in this region on milk and fat yields and protein percentage. This study specifically identified a 2.81 Mb BTA14 gene cluster with 125 genes with a concentration of SNP effects for fat percentage (Figure 2B), with *A5D786 *near the left end (Figure 3A) and *NIBP *at the right end (Figure 3B). The largest and second largest genes in this cluster were *NIBP *and *A5D786*, respectively.

Two significant SNP effects for calving traits were found in a region on BTA18 that Cole et al. [9] had reported to be associated with calving traits for U.S. Holsteins. That region included BTA-29287-no-rs that ranked 4th for service-sire calving ease, 2nd for daughter calving ease, and 1st for service-sire stillbirth and ARS-BFGL-BAC-36087 that ranked 92nd for daughter stillbirth (Additional file 2: Table S1). The most significant genes for calving traits identified in our study were *PGLYRP1-IGFL1 *and *LOC787057*. The *PGLYRP1-IGFL1 *region was 360 kb upstream and *LOC787057 *was 124 kb downstream from *SIGLEC12 *reported in Cole et al. [9]. Results from this study and from Cole et al. [9] indicate the 0.36 Mb region of *PGLYRP1-IGFL1-SIGLEC12-LOC787057 *should be an interesting target for factors that affect calving traits. Other dairy GWAS [10-12] had only a small number of traits that overlapped with this study. The *PGLYRP1-IGFL1 *region we identified was 150 kb upstream from *LOC538513 *that was reported to be associated with a direct calving ease effect. We also identified a SNP at BTAU_4.0 136,742,669 bp of BTA1 that ranked 69th for daughter pregnancy effect and this was close to a previously reported fertility effect at position 136,499,200 bp [12].

### Gene association with conformation traits

The SNP significance tests showed that different traits generally were associated with different genes or gene regions, but many of the traits also shared common genes or gene regions (Additional File 2: Table S1). Traits within a phenotype group (e.g., body size or udder conformation) had a greater tendency to have genes or gene regions with significant SNPs in common than did traits in different phenotype groups.

All four traits related to body size (stature, strength, body depth, and rump width) had top-20 SNPs in BTA16's *REN*, BTAX's *PHKA2 *and BTAX's *GPM6B*. The *REN *gene is part of the renin-angiotensin system that regulates cellular growth in response to developmental, physiological, and pathological processes [21]. In humans, *PHKA2 *is responsible for glycogen storage diseases [22]. These known biological functions of *REN *and *PHKA2 *are consistent with the highly significant effects of *REN *and *PHKA2 *on body size traits. The two body height traits (stature and body depth) had many significant SNPs with overlapping gene regions of BTA11. The two body width traits (strength and rump width) also had top-20 SNPs on chromosomes other than BTA16 and BTAX (e.g., BTA13 and BTA26 for strength and BTA19 for rump width). The two body shape traits (dairy form and rump angle) also had top-20 SNPs on BTA3, BTA7, BTA10 (for dairy form) and BTA5 and BTA9 (for rump angle).

Four udder traits (fore udder attachment, rear udder height, udder depth, and udder cleft) had top-20 SNPs with gene regions in common. Fore udder attachment and udder depth had BTA22 SNPs near the *succinate-CoA ligase, GDP-forming, beta subunit *(*SUCLG2*) gene, a BTA7 SNP in the *CREB regulated transcription coactivator 1 *(*CRTC1*) gene, and a BTA23 SNP in the *collagen, type XXI, alpha 1 *(*COL21A1*) gene. Rear udder height shared only one top 20 SNP on BTA7 with udder cleft but shared two BTA11 SNPs with body depth. Many top-20 SNPs in gene regions overlapped for udder cleft and teat placement traits; four BTA7 SNPs (with three near *CETN3*), two BTA6 SNPs in *LETM1 *and *WHSC2*, one BTA19 SNP in *GPRC5C*, and one BTAX SNP in *TAF1*. Udder cleft appears to be more related genetically to teat placement traits than to other udder traits. It is interesting to note that the most significant genes for body size, *REN *and *PHKA2*, also were most significant for fore udder attachment and were highly significant for rear udder height. Significant SNPs for front and rear teat placement had considerable effect overlap with udder cleft, indicating that teat positions and udder shape may share common genes. In contrast, teat length had little gene region overlap with SNPs for teat position or udder shape, indicating the likely involvement of different genes for these traits.

The three individual feet/legs traits (excluding feet/legs score) mostly involved different genes. The *MGMT *gene of BTA26 was most significant for foot angle and feet/legs score and was among the top 20 effects for rear legs (rear view). Most of the highly significant effects for rear legs (rear view) were on BTA11 and BTA18 while BTA1, BTA18 and BTA16 had most of the top 20 effects for rear legs (side view). Feet/legs score had more effects that overlapped with foot angle than with the two rear leg traits.

### Effect of X chromosome

Only limited research on the X chromosome is available, compared to the extensive literature on autosomes. In this study, the X chromosome had the largest number of significant SNP effects (Table 1) and the highest chromosomal frequency for the top 20 SNP effects for fat yield (along with BTA13), protein yield, protein percentage, productive life, somatic cell score (along with BTA2, BTA6, and BTA7), daughter pregnancy rate, service-sire stillbirth, and net merit. This was observed even though the X chromosome had the smallest number of SNPs among all chromosomes (1.67% for X chromosome compared with 3.33% per chromosome if the 45,878 SNPs had been distributed equally over all 30 chromosomes).

The most significant X chromosome effects based on effect ranking relative to autosome effect were on daughter pregnancy rate, productive life, protein percentage, somatic cell score and fat yield. The 106 Mb BTAX region near *LOC520057 *contained significant SNPs for daughter pregnancy rate, productive life and somatic cell score. Type 1 protein phosphatase has been implicated in the control of a range of cellular processes, including the cell cycle, gene expression, cell adhesion, and glycogen metabolism [23,24]. Two interesting gene clusters bracketed the *LOC520057 *gene. The *NDP-MAOA-MAOB *gene cluster was about 1.2 Mb upstream and *MAOB *plays an important role in the metabolism of neuroactive and vasoactive amines in the central nervous system and peripheral tissues [25]. The *LOC100138543-MID1IP1-MIR2488 *cluster was about 1.0 Mb downstream and *LOC100139224 *is the largest and most complicated enzyme of the electron transport chain [26]. The *MID1IP1 *portion of this cluster is *MID1 interacting protein 1 gastrulation specific G12 homolog (zebrafish)*. Gastrulation is an early phase of embryonic development so *MID1IP1*'s biology could be relevant to daughter pregnancy rate and productive life. The *MIR2488 *portion is a microRNA involved in post-transcriptional regulation of gene expression in multicellular organisms and affects both the stability and translation of mRNAs [26]. In addition, daughter pregnancy rate was associated with SNPs in the *ATP1B4 *and *GRIA3 *genes on BTAX. The protein encoded by *ATP1B4 *interacts with the nuclear transcriptional coregulator SKIP and may be involved in the regulation of TGF-beta signaling [25]. Glutamate receptors are the predominant excitatory neurotransmitter receptors [25].

### Significance and frequency

**Figure 5:**
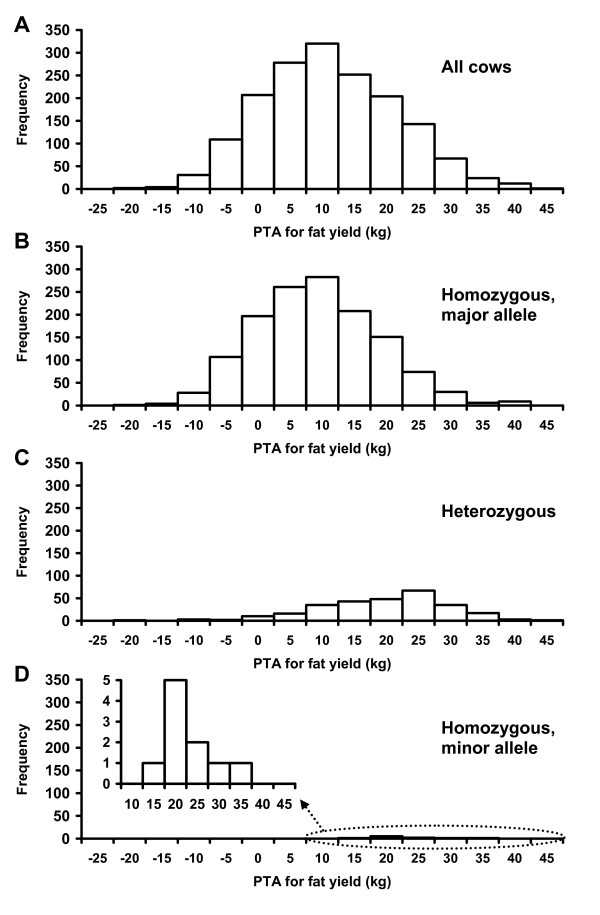
**Distribution of cows for predicted transmitting ability (PTA) for fat yield by allele combination for SNP BFGL-NGS-117985 in the *PGLYRP*-*IGFL1 *region of *Bos taurus *chromosome 18**. This SNP explained 13.68% of PTA variation (R^2 ^= 0.1368) of fat yield, was among the top 20 significant SNP effects for fat and protein yields, service-sire and daughter calving ease, service-sire stillbirth, net merit (ranked 1st), milk yield (ranked 9th), and productive life (ranked 16th), and was among the top 100 effects for fat and protein percentages. **A**) All cows. **B**) Cows homozygous for major allele of SNP. **C**) Cows heterozygous for SNP. **D**) Cows homozygous for minor allele of SNP.

Most of the top 20 SNP effects had the minor allele (frequency of <0.5) as the favorable allele (Additional File 5: Table S2), including the most significant SNP for fat percentage (ARS-BFGL-NGS-4939), which was located in *DGAT1*. Frequencies of favorable alleles were particularly low for protein yield and percentage, service-sire and daughter calving ease, and net merit. Daughter stillbirth was the only exception with major alleles (frequency of ≥0.5) as the favorable alleles for over half of the top 20 effects. Minor alleles were the favorable allele primarily because they were associated with high PTAs. For example, cows that were homozygous for the minor allele of BTA18's BFGL-NGS-117985, which was among the top 100 significant SNP effects for 10 of the thirteen production, health, and reproduction traits, had a PTA for fat yield of ≥15 kg, whereas cows homozygous for the major allele had PTAs between -21 and 41 kg, with a mean of 9 kg (Figure 5). The frequency of the favorable allele of this marker was 9.1% in the 1,654 contemporary U.S. Holstein cows. Because of low allele frequencies, some of the highly favorable associations with minor alleles could be due to sampling rather than biological effects.

The majority of the 1,005 SNPs for body conformation traits had intermediate allele frequencies. Only 94 of the 1,005 SNPs had a minor allele frequency of <0.10 (Additional File 2: Table S1). Because an intermediate value may be optimal for many conformation traits, few SNPs were driven towards fixation. The intermediate frequencies should allow considerable flexibility in genetic selection for improving conformation traits and associated functionality.

### Sensitivity of association results to PTA variations

PTA values from different individuals had different accuracies measured by reliability [27]. Results reported in this study were based on PTA values without being adjusted by the reliability of each PTA value. This approach allowed the use of all PTA values including PTA values with zero estimates of reliability. To study the effects of different accuracies on the SNP results, we also analyzed the data using a weighted least squares analysis, with the reliability as the weight of each PTA value. The results from this weighted least squares analysis were similar to the original analysis without considering PTA accuracies (Additional File 9: Table S3). The four calving traits had 360 individuals with zero reliability values, but the weighted least squares results were similar to the original results in terms of effect ranking and statistical significance, particularly for the top 50 effects. For daughter calving ease, the original least squares effects ranked below 50^th ^had relatively poor overlap with the weighted least squares effects.

## Conclusions

Genome-wide association analysis of U.S. contemporary Holstein cows produced comprehensive descriptions of genes and chromosome regions associated with 31 production, health, reproduction and body conformation phenotypes and provided a large quantity of genome annotation details for phenotypic effects based on the latest bovine genome sequencing results and SNP chip development. The results of this study should significantly contribute to the process of building consensus of dairy QTL effects. The results support the polygenic hypothesis for all 31 traits in this study. Production, health and reproduction traits involved more gene clusters of tightly linked genes than body conformation traits, indicating that genetic mechanisms of production, health and reproduction were more complex than those of body conformation traits.

## Methods

### Phenotypic data, study population and SNP genotyping

Thirty one dairy traits, including 13 production, health and reproduction traits and 18 body conformation traits were studied. Traditional predicted transmitting abilities (PTAs) for each trait calculated by the U.S. Department of Agriculture (Beltsville, MD) were phenotypic data for association with SNPs. The 13 production, health and reproduction traits were milk, fat and protein yields, fat and protein percentages, productive life, somatic cell score, daughter pregnancy rate, service-sire and daughter calving ease, service-sire and daughter stillbirth, and a genetic-economic index for lifetime net merit. The 18 conformation traits as defined by Holstein Association USA and the World Holstein Friesian Federation [16,17] were stature (distance from the top of the spine in between the hips to the ground), strength (inside surface between the top of the front legs; also known as chest width), body depth (distance at the last rib from the top of the spine to the bottom of the barrel), dairy form (angle and openness of the ribs combined with the flatness of bone to avoid coarseness; also known as angularity), rump angle (angle of the rump structure from the hips to the pins), rump width (distance between the most posterior point of the pin bones; also known as thurl width), fore udder attachment (strength of the attachment of the fore udder to the abdominal wall), rear udder height (distance from the bottom of the vulva to milk-secreting tissue in relation to the height of the animal), udder depth (distance from the lowest part of the udder floor to the hock), udder cleft (depth of the cleft at the base of the rear udder; also known as central ligament), front teat placement (position of the front teat relative to the center of the udder quarter), rear teat placement (position of the rear teat relative to the center of the udder quarter), teat length (length of the front teat), foot angle (angle at the front of the rear right hoof measured from the floor to the hairline), rear legs (side view) (angle measured at the front of the hock; also known as set of rear legs), rear legs (rear view) (direction of feet when viewed from the rear of the animal), feet/legs score (an overall score assigned by a classifier based on side and rear views of rear legs, locomotion, feet, thurl position, hocks, bone, and pasterns), and final score (an overall conformation score assigned by a classifier based on front end and body capacity, dairy strength, rump, feet and legs and udder; also known as PTA type).

The design of the study population aimed at having a broad representation of contemporary U.S. Holstein cows. The 1,654 cows in the study population included elite and average Holstein cows for which DNA was supplied by Genetic Visions (Middleton, WI), Genex Cooperative (Shawano, WI), Holstein Association USA (Brattleboro, VT), Iowa State University (Ames, IA), Pennsylvania State University (University Park, PA), the University of Florida (Gainesville, FL), the University of Minnesota (St. Paul, MN), and Virginia Polytechnic Institute and State University (Blacksburg, VA).

A total of 45,878 SNP markers from the BovineSNP50 BeadChip (Illumina, San Diego, CA) were selected for a dual purpose research of association analysis in this study and a selection signature analysis [28]. This SNP set required an allele frequency difference of ≥2% between the study population and a group of 301 Holstein cattle that have remained unselected since 1964 to allow identification of near-fixed alleles in the contemporary population due to selection. Of the 45,878 SNP markers, 45,461 had known chromosome positions with mean marker spacing of 58.45 kb. Extraction of DNA and SNP genotyping were performed at the Bovine Functional Genomics Laboratory (Agricultural Research Service, U.S. Department of Agriculture, Beltsville, MD). Marker genotypes were scored using GenomeStudio software (version 1.1.9; Illumina, San Diego, CA).

### Data analyses

Statistical tests of SNP effects were conducted using the epiSNP computer package [29,30]. The epiSNP package implements the extended Kempthorne model that allows linkage disequilibrium between SNPs and Hardy-Weinberg disequilibrium for each SNP [31]. Normality of phenotypic residuals of each trait was evaluated using the R package [32] and residual values for all traits were found to satisfy the bell shaped normal distribution. Since PTA values are predicted additive genetic effects after removing fixed non-genetic effects such as herd-year-season, the statistical model did not need to consider fixed non-genetic effects. The statistical model for testing SNP-phenotype association used a single-locus model: PTA = μ + g + e, where μ = common mean, g = SNP genotypic effect, and e = random residual. Based on estimates of SNP genotypic values from least squares (LS) regression, the epiSNP package tests three effects for each locus by default; the marker genotypic effect, additive and dominance effects. The marker genotypic effect was tested using *F*-test, while additive and dominance effects were tested using t-test by the following t-statistic [31]: , where **s**_i _is a function of marginal and conditional probabilities calculated from SNP genotypic frequencies and is a row vector of contrast coefficients of the SNP genotypic effects for defining additive or dominance effect, and  is a column vector of LS estimates of three SNP genotypic effects. Although we did not expect to detect dominance effects because PTA values are estimated additive genetic effects, the test of dominance effects provided a check on whether the statistical tests produced unexpected genetic effects. The results were as expected. Only spurious dominance effects were observed and no dominance effect was among the top 100 effect for any trait.

The PTA values from different individuals had different accuracies measured by reliability [27]. The statistical analysis described above did not consider different PTA accuracies of different individuals but allowed the use of all PTA values including PTA values with zero estimates of reliability. To study the effects of different accuracies on SNP test results, we analyzed the data using a weighted least squares analysis (WLS), with the reliability as the weight of each PTA value. This approach gave more weight to PTA values with higher reliability values and ignored PTA values with zero reliability estimates. The  was estimated by (**X**'**WX**)^-1^(**X**'**Wz**)^-1^, where  is a column vector of WLS estimates of three SNP genotypic effects, **W **is diagonal matrix with reliability estimates as the diagonal elements, **X **is the model matrix for PTA values as deviation from the common mean, and **z **is a column vector of PTA values as deviation from the common mean. The t-test under WLS replaced (**X**'**X**)^-1 ^in the standard deviation of  with (**X**'**WX**)^-1^.

A genome-wide 5% type-I error with the Bonferroni correction was considered as the threshold P value (10^-6.4^) for genome-wide significance. The contribution of the top 100 SNP effects for each trait was measured by the coefficient of determination (R^2^) and calculated using the linear regression procedure (PROC REG) of SAS [33]. Gene and SNP locations were identified based on the University of Maryland bovine genome assembly (UMD 3.0) [5,34]. Location of SNPs based on the Baylor College of Medicine bovine genome assembly Build 4.0 (Btau_4.0) from NCBI [24] and ENSEMBL [35] are noted in the results. Figures of gene clusters were from ENSEMBL based on Btau_4.0 because such figures based on the UMD assembly were not available.

## List of abbreviations

**GWAS**: genome-wide association study; **SNP**: single nucleotide polymorphism; **bp**: base pair; **kb**: kilo base pairs = 1000 base pairs; **Mb**: mega bases pairs = 1000 kb = 1 million base pairs; **MAF**: minor allele frequency; **PTA**: predicted transmitting ability; **QTL**: quantitative trait locus; **LS**: least squares; **WLS**: weighted least squares; **R^2^**: coefficient of determination; **Chr**: chromosome; **MY**: milk yield; **FY**: fat yield; **PY**: protein yield; **FPC**: fat percentage; **PPC**: protein percentage; **PL**: productive life; **SCS**: somatic cell score; **DPR**: daughter pregnancy rate; **SCE**: service-sire calving ease; **DCE**: daughter calving ease; **SSB**: service-sire stillbirth; **DSB**: daughter stillbirth; **NM**: net merit; **STA**: stature; **STR**: strength; **BD**: body depth, **RW**: rump width; **DF**: dairy form; **RA**: rump angle; **FUA**: fore udder attachment; **RUH**: rear udder height; **UD**: udder depth; **UC**: udder cleft; **FTP**: front teat placement; **RTP**: rear teat placement; **TL**: teat length; **FA**: foot angle; **RLS**: rear legs (side view); **RLR**: rear legs (rear view); **FL**: feet/legs score; **FS**: final score.

## Competing interests

The authors declare that they have no competing interests.

## Authors' contributions

YD, TSS, GRW, JBC, CPVT and BAC organized and implemented this study. JBC, GRW and YD led the manuscript preparation. TSS directed the genotyping work. YD, LM, JY and SW led the data analysis. TJL provided the Holstein body conformation data and input in interpreting the results. LKM and TSS provided support for UMD 3.0 bovine genome assembly. All authors read and approved this manuscript.

## Supplementary Material

Additional file 1 **Figure S1. Global view of P-values of 45,878 SNP effects per trait for 31 production, health, reproduction and body conformation traits by Mahattan plot**. MY, milk yield; FY, fat yield; PY, protein yield; FPC, fat percentage; PPC, protein percentage; SCS, somatic cell score; DPR, daughter pregnancy rate; PL, productive life; SCE, sire calving ease; DCE, daughter calving ease; SSB, sire stillbirth; DSB, daughter stillbirth; NM, net merit; STA, stature; STR, strength; BD, body depth; RW, rump width; DF, dairy form; RA, rump angle; FUA, fore udder attachment; RUH, rear udder height; UD, udder depth; UC, udder cleft; FTP, front teat placement; RTP, rear teat placement; TL, teat length; FA, foot angle; RLS, rear legs (side view); RLR, rear legs (rear view); FL, feet and legs; FS, final score. Click here for file

Additional file 2 **Table S1. Output file of all 1,586 SNP markers with 1,300 effects on 31 dairy traits by chromosome (Chr)**. Chr30 is the X chromosome, and Chr32 indicates markers with unknown chromosome locations. MY, milk yield; FY, fat yield; PY, protein yield; FPC, fat percentage; PPC, protein percentage; SCS, somatic cell score; DPR, daughter pregnancy rate; PL, productive life; SCE, sire calving ease; DCE, daughter calving ease; SSB, sire stillbirth; DSB, daughter stillbirth; NM, net merit; STA, stature; STR, strength; BD, body depth; RW, rump width; DF, dairy form; RA, rump angle; FUA, fore udder attachment; RUH, rear udder height; UD, udder depth; UC, udder cleft; FTP, front teat placement; RTP, rear teat placement; TL, teat length; FA, foot angle; RLS, rear legs (side view); RLR, rear legs (rear view); FL, feet and legs; FS, final score. Columns B and C show UMD 3.0 chromosome and positions, columns D and E show Btau_4.0 chromosome and position, respectively; column G is A1F, A1 allele frequency; column H is MAF, minor allele frequency; columns J to AN are P values (top 100 effects in red); columns AO to CY are t values (those corresponding to top 100 effects for P values in blue); column CZ is N, number of traits for which SNP was among top 100. Click here for file

Additional file 3 **Figure S2. Map of SNP position (Mb) by *P*-value and *Bos taurus *chromosome for 725 SNPs that comprise the top 100 effects for each of 13 production, health, and reproduction traits of contemporary U.S. Holsteins**. Click here for file

Additional file 4 **Figure S3. Map of SNP position (Mb) by *P*-value and *Bos taurus *chromosome for 1,005 SNPs that comprise the top 100 effects for each of 18 conformation traits of contemporary U.S. Holsteins**. Click here for file

Additional file 5 **Table S2. Top 20 most significant SNP effects for each of the thirty one traits**. Click here for file

Additional file 6 **Figure S4. Gene clusters that overlapped localized concentrations of SNP effects or contained significant SNP effects on *Bos taurus *(BTA) chromosomes**. **A**) BTA7 15.4 Mb gene cluster (Btau_4.0:4807980-20004663) of ~1,166 genes (not counting pseudo and RNA genes) with a mean of 75 genes/Mb. Left end was *LSM4*-*JUND *region; right end was *LSM7*-*SPPL2B*-*OAZ1 *region. This cluster included ARS-BFGL-NGS-4774 [1.5 kb downstream from *INSR *(*Q95M43*; highlighted in green)], which was significant for somatic cell score (1st) and daughter pregnancy rate (1st), productive life (3rd), and net merit (78th)] as well as all 10 significant SNP effects on this chromosome for productive life, 15 of the 16 significant SNP effects for daughter pregnancy rate, and 6 of the 12 significant SNP effects for somatic cell score. **B**) BTA14 2.81 Mb gene cluster (Btau_4.0:50872-2,859,132) of ~125 genes with a mean of 44 genes/Mb; *NIBP *(highlighted in green) was the largest gene (387.23 kb) in the cluster. This cluster included 19 significant SNP effects for fat percentage [SNP in *DGAT1 *was 1st and SNP in *NIBP *was 2nd], one SNP in *VPS28 *with effect for milk yield, and one SNP in *NIBP *with effects for fat yield and protein percentage. **C**) BTA18 15.82 Mb gene cluster (Btau_4.0:48755332-64574451) of ~1,322 genes with a mean of 83 genes/Mb. The *PGLYRP1(PGRP)*-*IGFL1 *(highlighted in green) region had the most significant SNP effects in the cluster [fat and protein yields, service-sire and daughter calving ease, and net merit (1st); service-sire stillbirth (8th); milk yield (9th); productive life (16th); and fat and protein percentages (25th)]. **D**) BTA3 gene cluster with significant SNPs for fat yield [BFGL-NGS-113990 (49th) and INRA-304 (53rd)]. **E**) BTA5 gene cluster with ARS-BFGLNGS-36745 (associated with *SREBF2*) and ARS-BFGL-NGS-71946 in *LOC535121*, which were among top 100 SNP effects for fat and protein percentages and service-sire calving ease and stillbirth, as well as other significant SNPS for service-sire stillbirth [ARS-BFGL-NGS-2337 in *TCF20 *(39th) and ARS-BFGL-NGS-7380 in *SEPT3 *(76th). **F**) BTA7 gene cluster with ARS-BFGL-NGS-76638 in *RGS14*, which was significant for somatic cell score (77th). **G**) BTA10 gene cluster with Hapmap41316-BTA-62253, which was significant for milk yield (60th). **H**) BTA13 gene cluster with significant SNPs for milk yield [ARS-BFGL-BAC-16372 (65th) and Hapmap41228-BTA-32897 (87th)]. **I**) BTA17 gene cluster with significant SNPs for milk yield [(ARS-BFGL-NGS-17192 (39th) and BTB-01992588 (63rd)] and fat percentage [ARS-BFGL-NGS-34106 (19th) and Hapmap40427-BTA-41914 (78th)]. **J**) BTA21 gene cluster with significant SNP effects for service-sire calving ease [BFGL-NGS-113671 (87th) and service-sire stillbirth [BFGL-NGS-116152 (46th), Hapmap39755-BTA-52639 (73rd), and BFGL-NGS-113671 (83rd). **K**) BTA23 gene cluster with ARS-BFGL-NGS-72191 in *ZNF192 *and BTA-68781-no-rs, which were among top 100 SNP effects for fat yield, protein yield and percentage, service-sire calving ease, and net merit. **L**) BTA26 gene cluster with BFGL-NGS-111739, the 14th most significant SNP for service-sire stillbirth. **M**) BTA29 gene cluster with ARS-BFGL-NGS-24998 in *LOC787296*, 49th most significant SNP for daughter stillbirth. **N**) BTAX gene cluster with ARS-BFGL-NGS-42972 in *TIMP1*, 45th most significant SNP for service-sire stillbirth. Click here for file

Additional file 7 **Figure S5. Examples of gene regions associated with significant effects of SNPs for daughter stillbirth and milk yield**. **A**) The 211.67 kb *MOCS1*-*LRFN2 *region of *Bos taurus *(BTA) chromosome 23 with nine highly significant effects (red arrows) for predicted transmitting ability (PTA) for daughter stillbirth. **B**) The most significant SNP effect (red arrow) for daughter stillbirth was 23.9 kb upstream from the second *CD82 *gene on BTA15. **C**) The BTA13 region with the most significant SNP effect (19.7 kb downstream from *GNAS2 *or *GNAS*) for PTA for milk yield. Click here for file

Additional file 8 **Figure S6. Examples of gene regions associated with significant SNP effects for body conformation traits; a boxed gene contained at least one top-100 effect, and numbers above red arrows (significant SNP effects) indicate rank of SNP effect**. **A) **The 1 Mb region of *Bos taurus *(BTA) chromosome X with the most significant SNP effect (*LOC515732 *is *PHKA2*) for predicted transmitting ability (PTA) for stature. **B) **The 10.2 Mb region of BTA 11 with eight of the top 20 SNP effects for stature. **C) **The 1 Mb region of BTA16 with the most significant SNP effect (*REN*) for PTA for strength. **D) **The 1.75 Mb region of BTA19 with five genes with highly significant SNP effects for rump width and udder cleft. **E) **A BTA21 gene cluster with three genes associated with teat length. Click here for file

Additional file 9 **Table S3. Output file of 725 SNP markers with top 100 effects per trait from least squares analysis and weighted least squares analysis**. MY, milk yield; FY, fat yield; PY, protein yield; FPC, fat percentage; PPC, protein percentage; SCS, somatic cell score; DPR, daughter pregnancy rate; PL, productive life; SCE, sire calving ease; DCE, daughter calving ease; SSB, sire stillbirth; DSB, daughter stillbirth; NM, net merit. Subscript 'w' indicates P-values from weighted least squares analysis. Trait name with subscript 'w' indicates tests using weighted least squares, and trait name without subscript indicates tests using least squares. Click here for file
