# Supplementary material for: Genome-wide association analysis of thirty one production, health, reproduction and body conformation traits in contemporary U.S. Holstein cows
Source: BMC Genomics. 2011 Aug 11;12:408. doi: 10.1186/1471-2164-12-408 (PMC3176260; doi:10.1186/1471-2164-12-408)

Manhattan Plot: MY

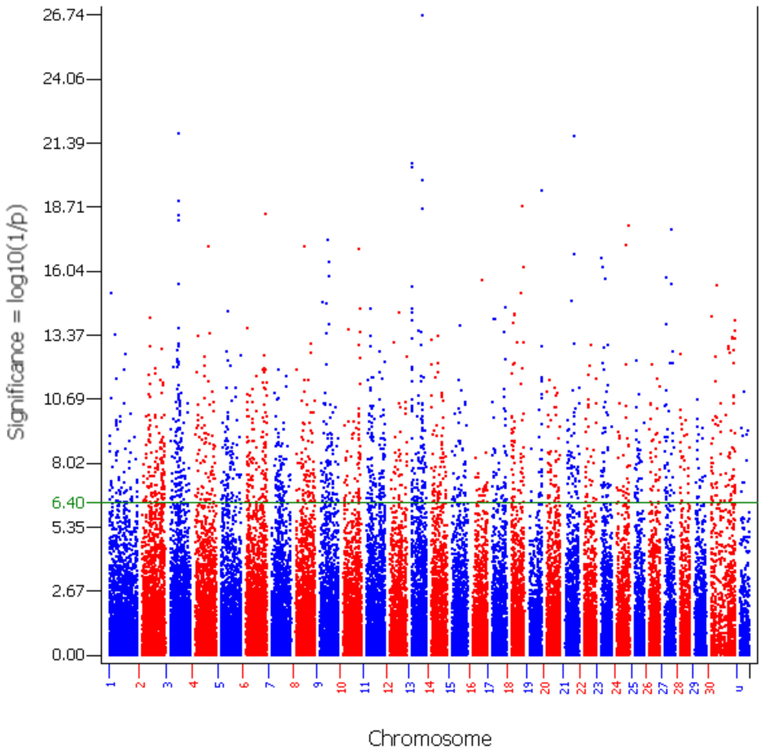

Manhattan Plot: FY

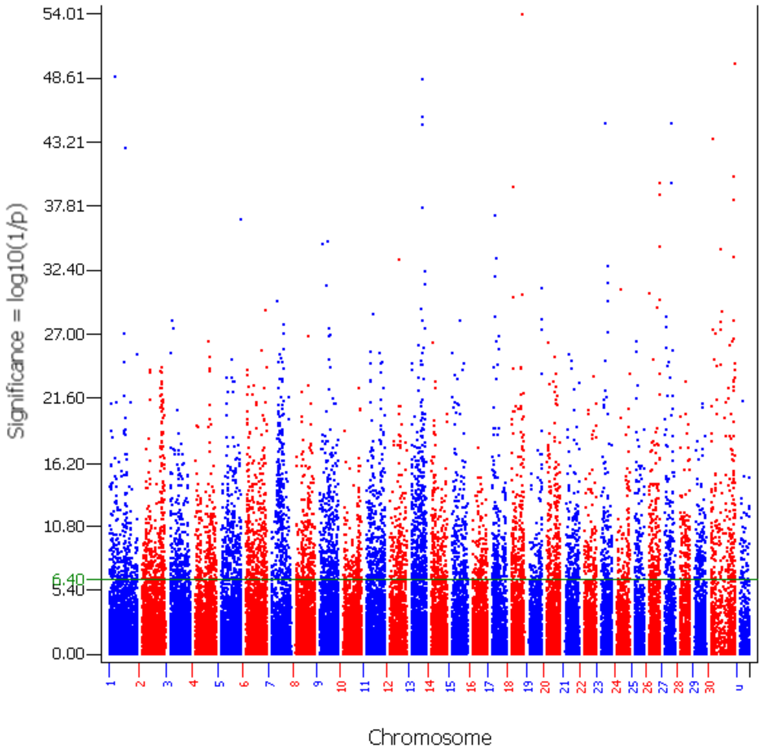

Manhattan Plot: PY

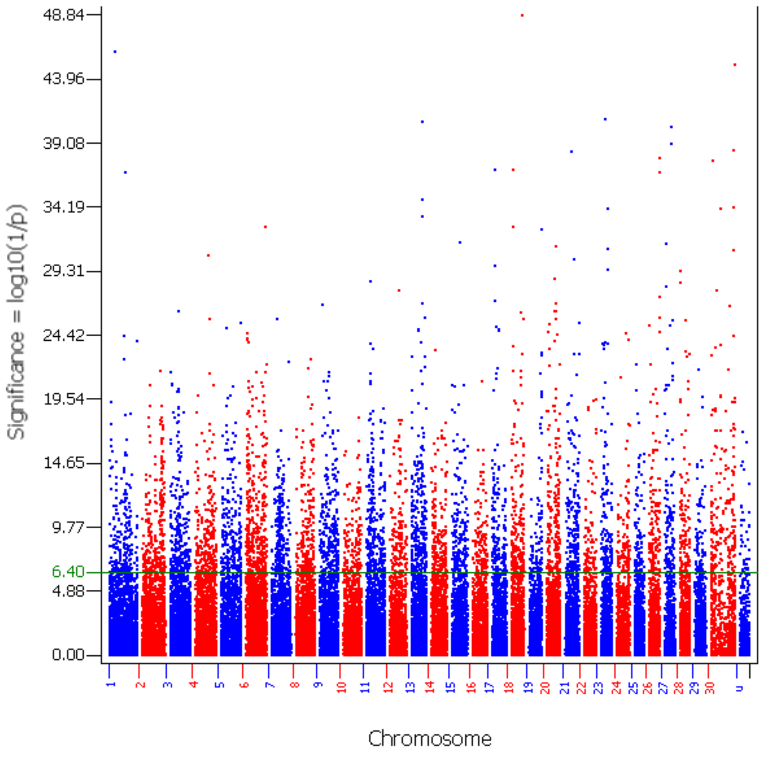

Manhattan Plot: FPC

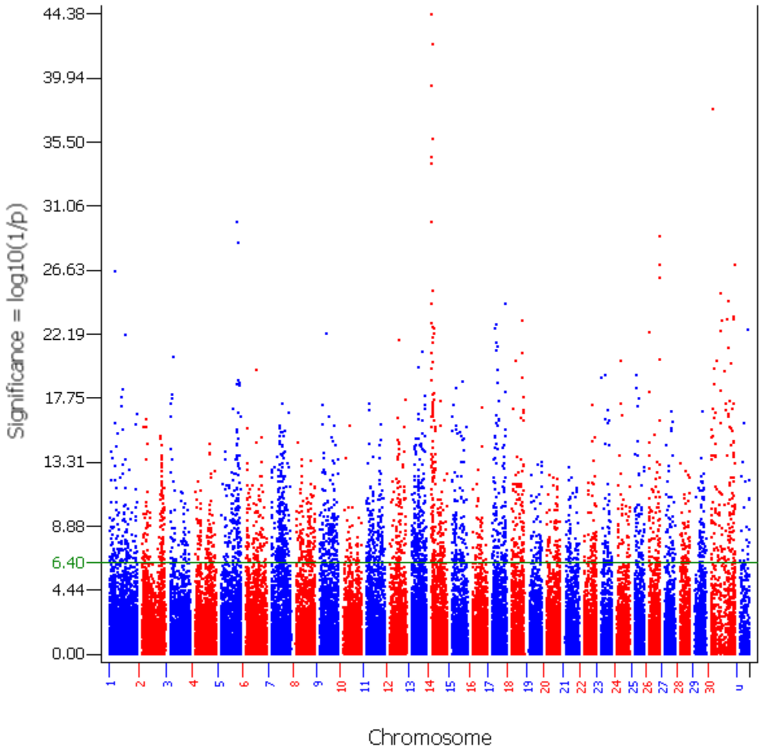

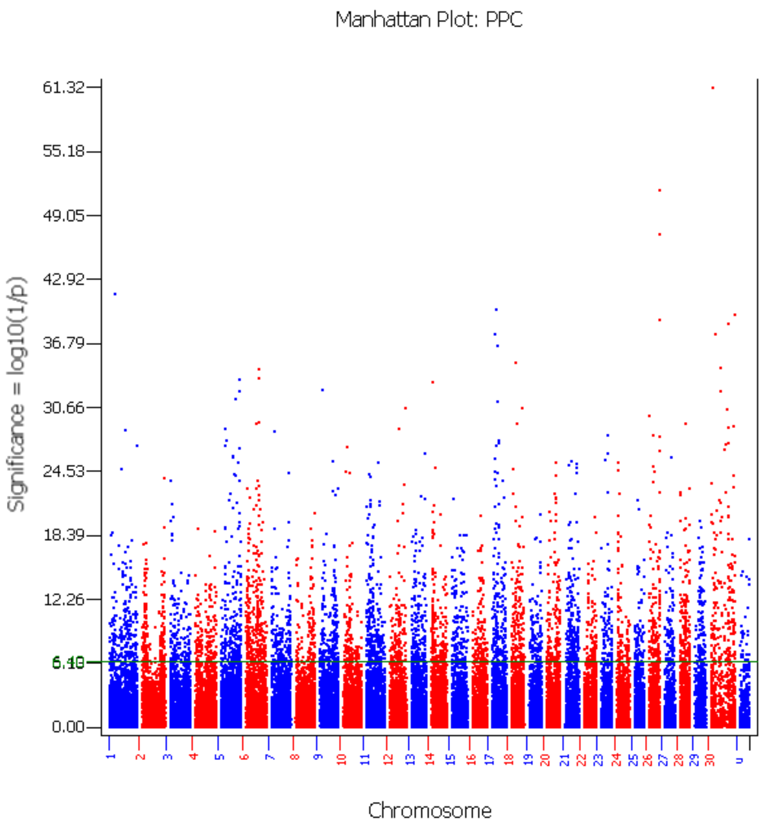
Manhattan Plot: PPC
Significance = log10(1/p)
Chromosome

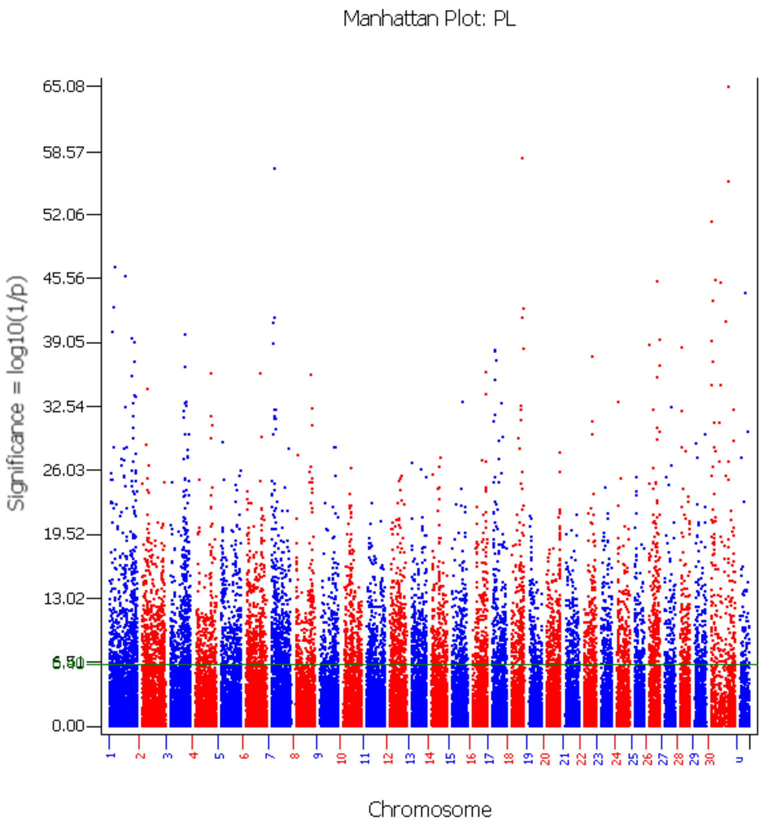
Manhattan Plot: PL
Significance = log10(1/p)
Chromosome

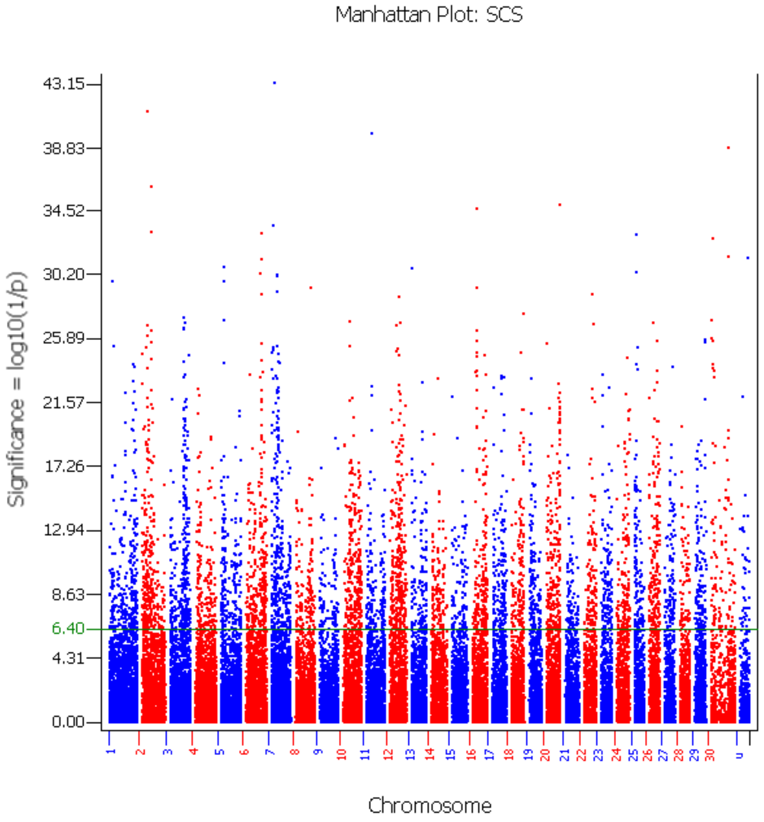
Manhattan Plot: SCS
Significance = log10(1/p)
43.15
38.83
34.52
30.20
25.89
21.57
17.26
12.94
8.63
6.40
4.31
0.00
1 2 3 4 5 6 7 8 9 10 11 12 13 14 15 16 17 18 19 20 21 22 23 24 25 26 27 28 29 30 u
Chromosome

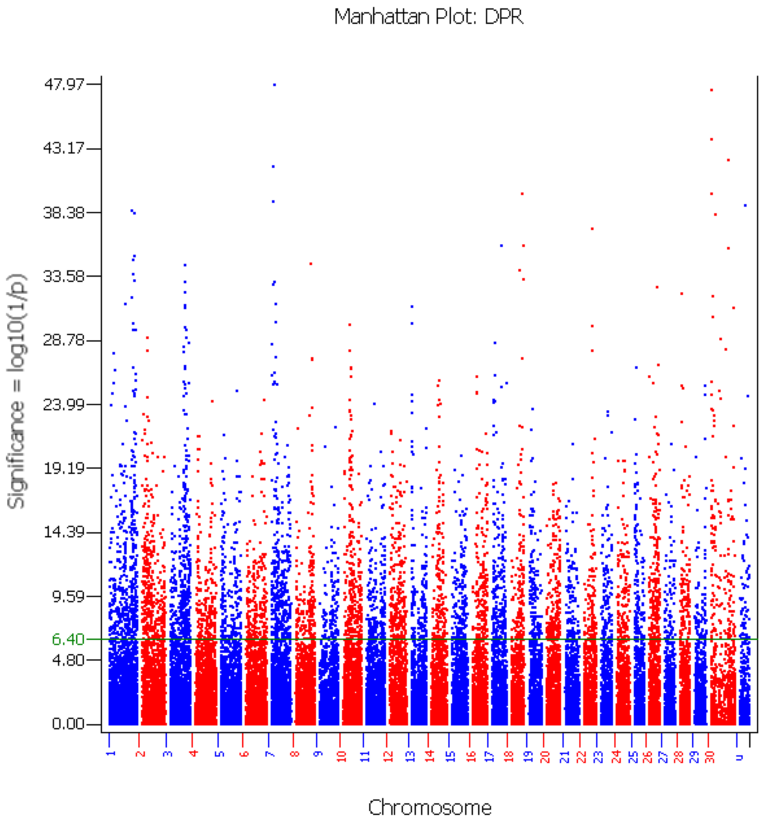
Manhattan Plot: DPR
Significance = log10(1/p)
47.97
43.17
38.38
33.58
28.78
23.99
19.19
14.39
9.59
6.40
4.80
0.00
1 2 3 4 5 6 7 8 9 10 11 12 13 14 15 16 17 18 19 20 21 22 23 24 25 26 27 28 29 30 u
Chromosome

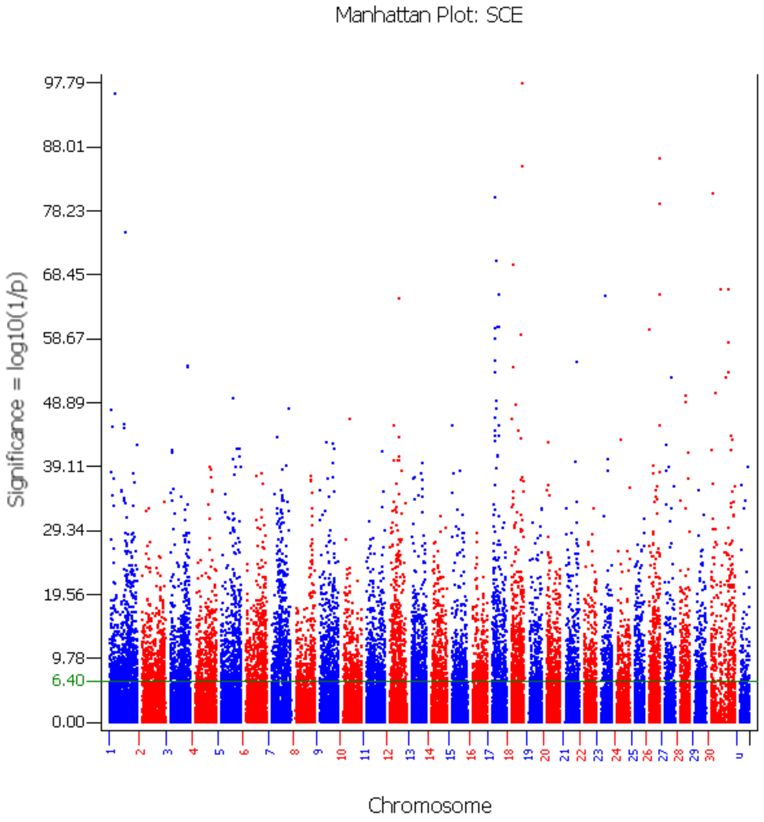
Manhattan Plot: SCE
Significance = log10(1/p)
97.79
88.01
78.23
68.45
58.67
48.89
39.11
29.34
19.56
9.78
6.40
0.00
1 2 3 4 5 6 7 8 9 10 11 12 13 14 15 16 17 18 19 20 21 22 23 24 25 26 27 28 29 30 u
Chromosome

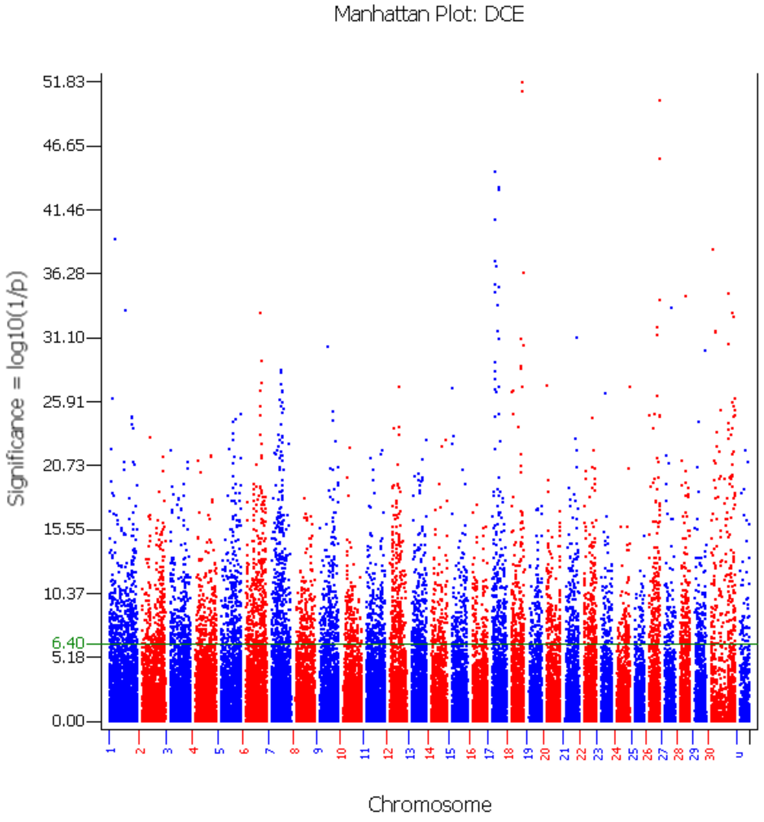
Manhattan Plot: DCE
Significance = log10(1/p)
51.83
46.65
41.46
36.28
31.10
25.91
20.73
15.55
10.37
6.40
5.18
0.00
1 2 3 4 5 6 7 8 9 10 11 12 13 14 15 16 17 18 19 20 21 22 23 24 25 26 27 28 29 30 u
Chromosome

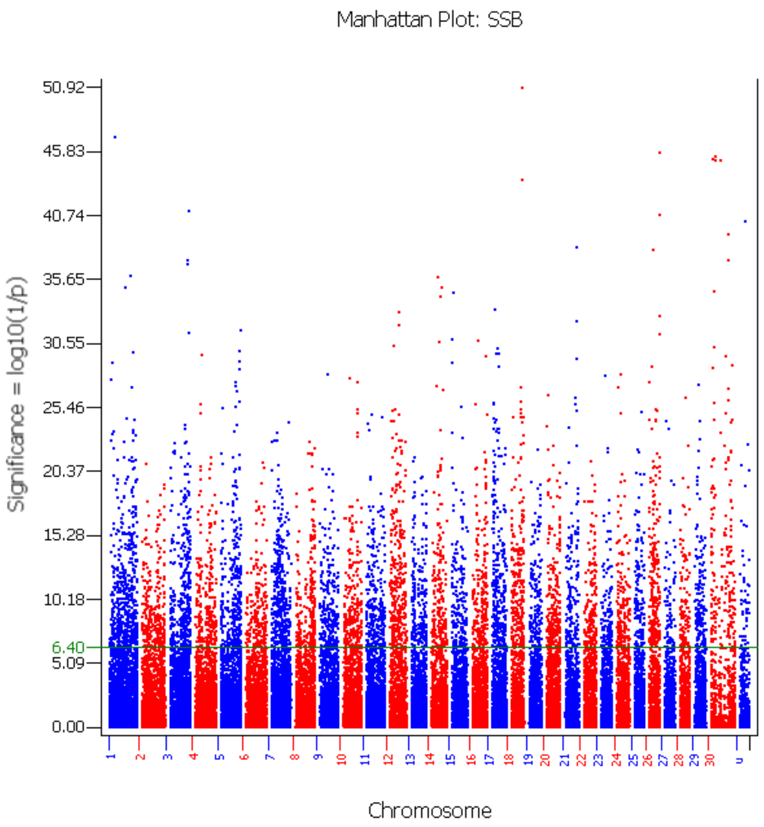
Manhattan Plot: SSB
Significance = log10(1/p)
50.92
45.83
40.74
35.65
30.55
25.46
20.37
15.28
10.18
6.40
5.09
0.00
1 2 3 4 5 6 7 8 9 10 11 12 13 14 15 16 17 18 19 20 21 22 23 24 25 26 27 28 29 30 u
Chromosome

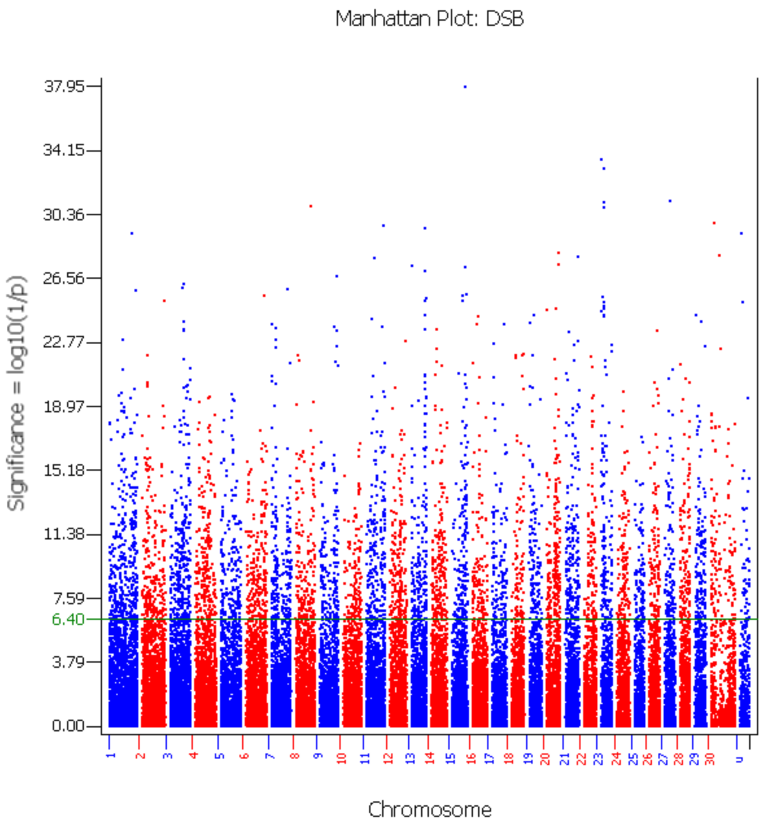
Manhattan Plot: DSB
Significance = log10(1/p)
37.95
34.15
30.36
26.56
22.77
18.97
15.18
11.38
7.59
6.40
3.79
0.00
1 2 3 4 5 6 7 8 9 10 11 12 13 14 15 16 17 18 19 20 21 22 23 24 25 26 27 28 29 30 u
Chromosome

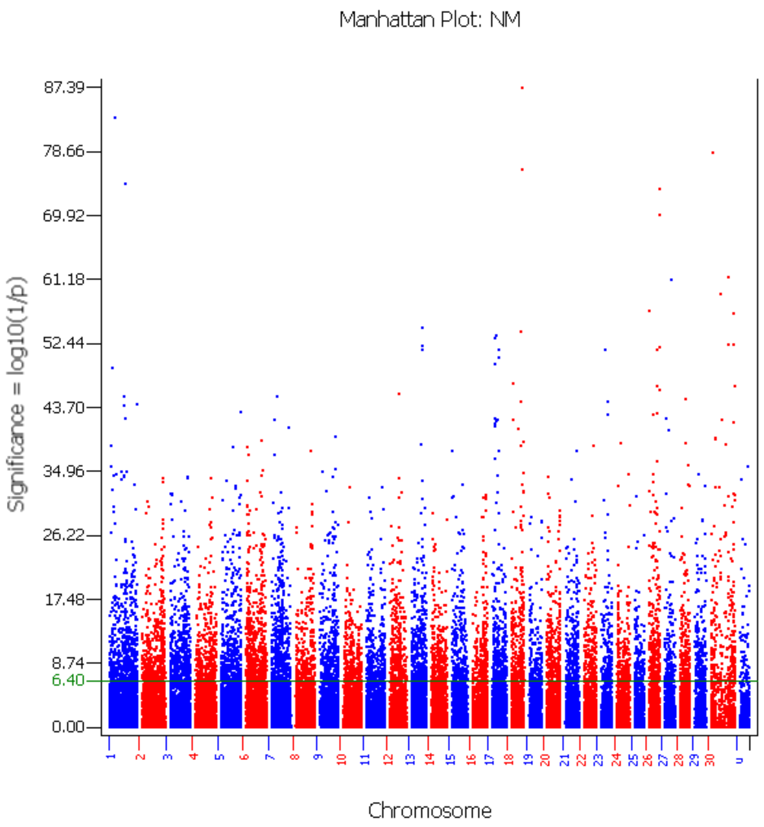
Manhattan Plot: NM
Significance = log10(1/p)
87.39
78.66
69.92
61.18
52.44
43.70
34.96
26.22
17.48
8.74
6.40
0.00
1 2 3 4 5 6 7 8 9 10 11 12 13 14 15 16 17 18 19 20 21 22 23 24 25 26 27 28 29 30 u
Chromosome

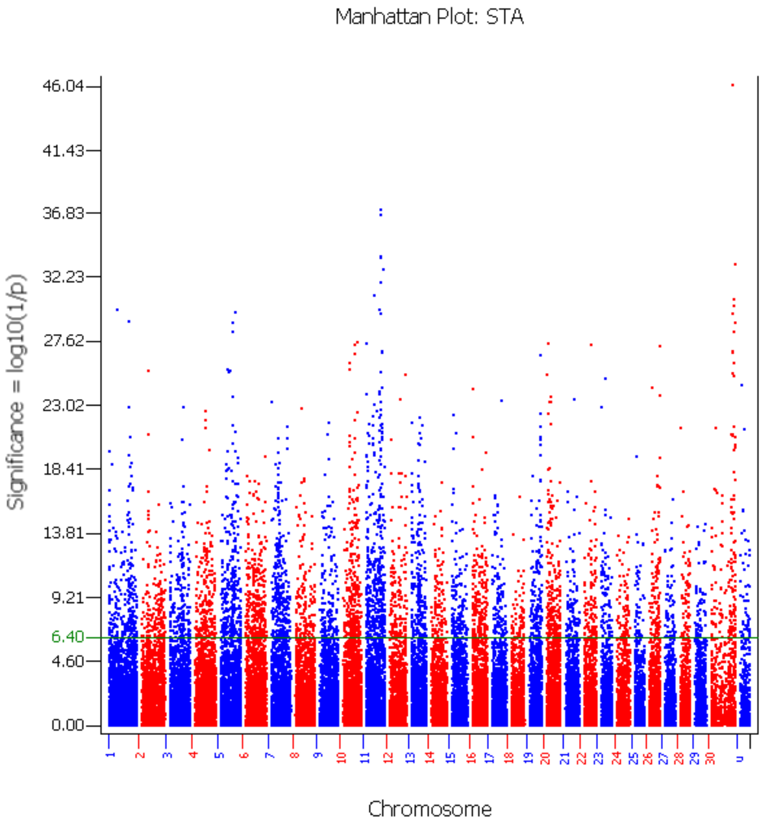
Manhattan Plot: STA
Significance = log10(1/p)
46.04
41.43
36.83
32.23
27.62
23.02
18.41
13.81
9.21
6.40
4.60
0.00
1 2 3 4 5 6 7 8 9 10 11 12 13 14 15 16 17 18 19 20 21 22 23 24 25 26 27 28 29 30 u
Chromosome

Manhattan Plot: STR

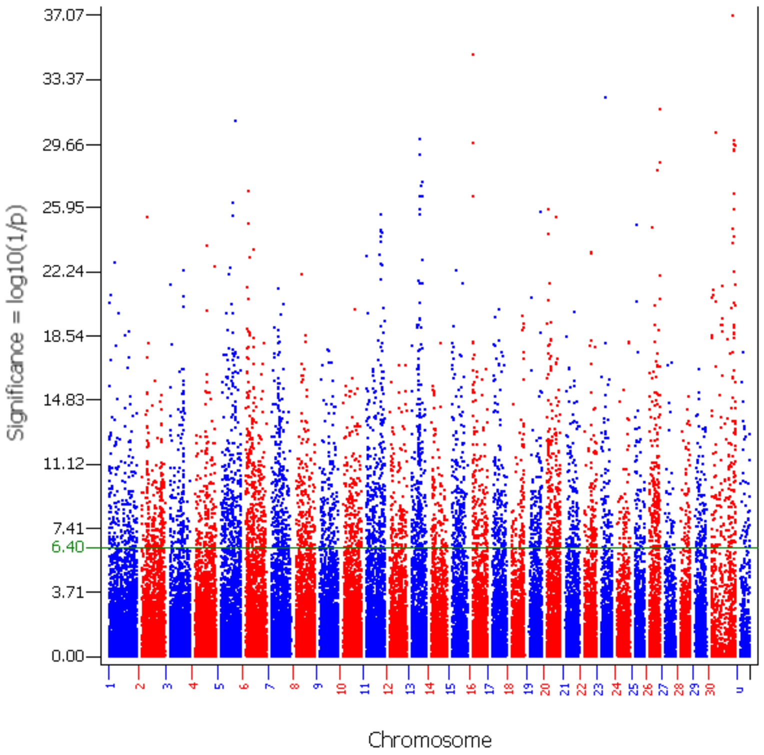

Manhattan Plot: BD

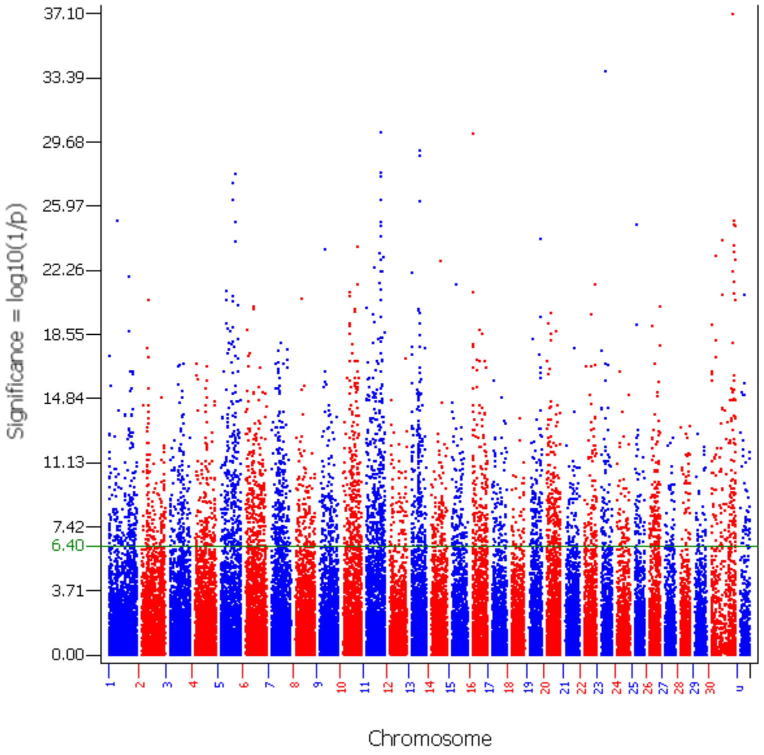

Manhattan Plot: RW

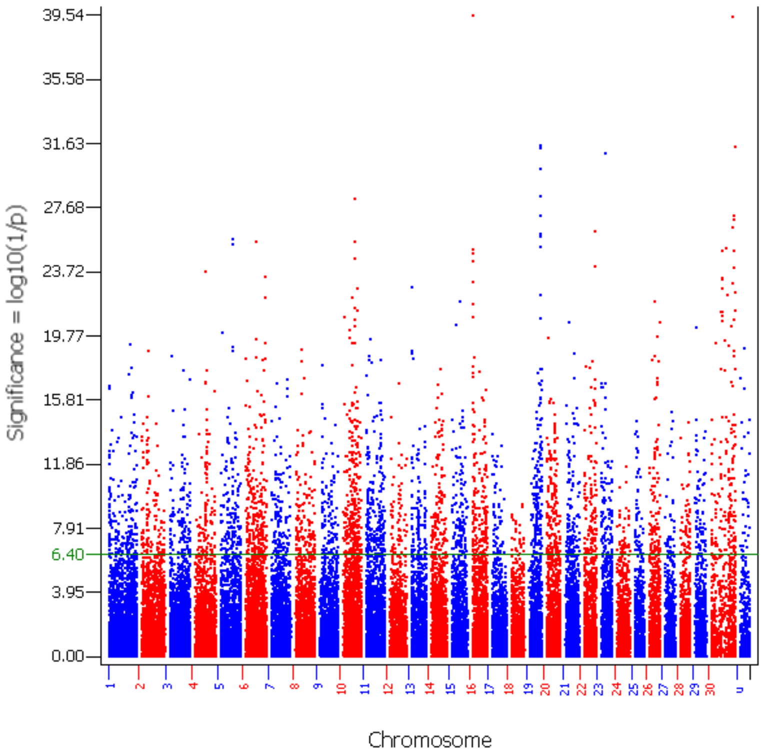

Manhattan Plot: DF

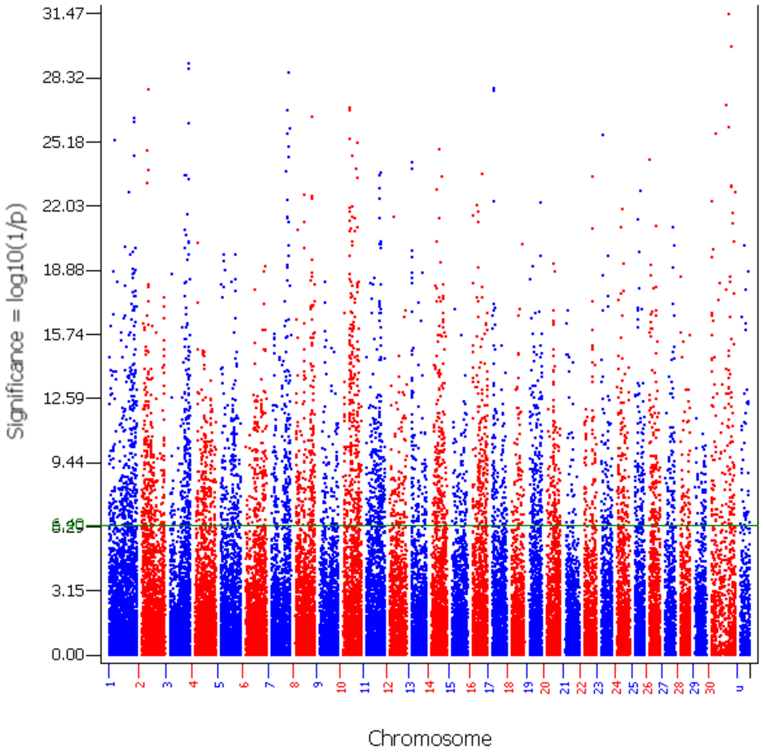

Manhattan Plot: RA

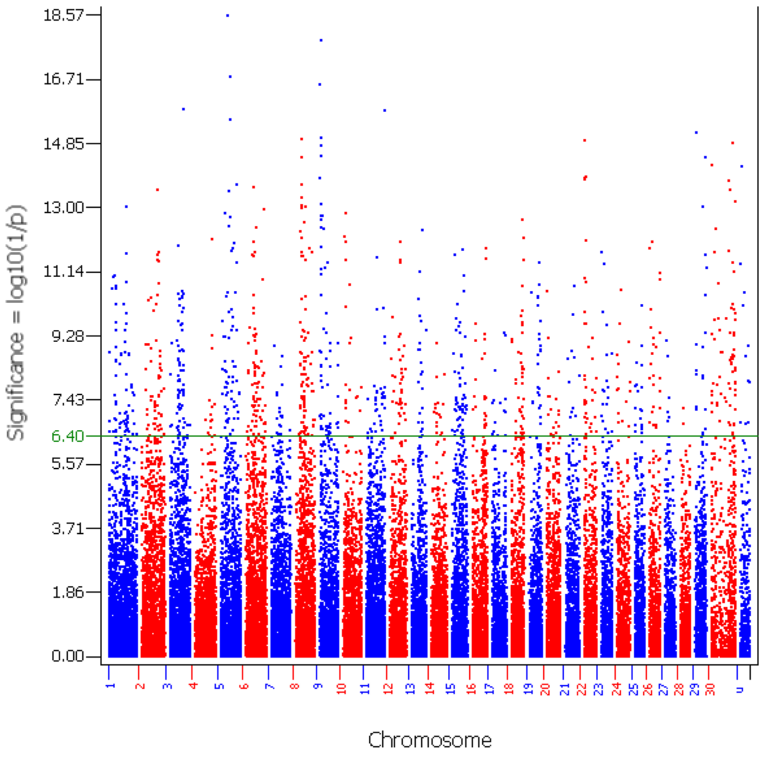

Manhattan Plot: FUA

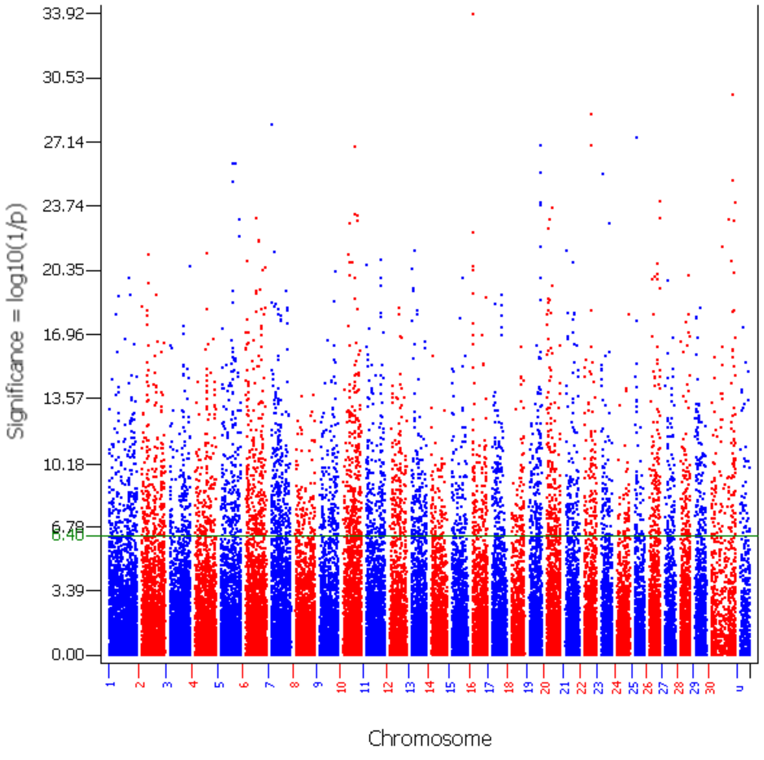

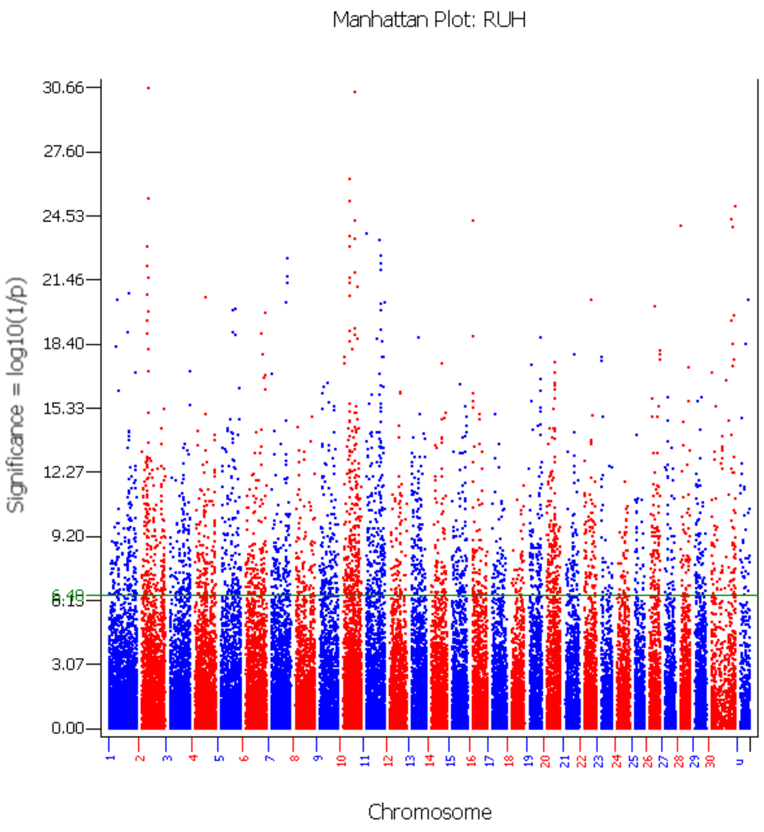
Manhattan Plot: RUH
Significance = log10(1/p)
30.66
27.60
24.53
21.46
18.40
15.33
12.27
9.20
6.48
6.13
3.07
0.00
1 2 3 4 5 6 7 8 9 10 11 12 13 14 15 16 17 18 19 20 21 22 23 24 25 26 27 28 29 30 u
Chromosome

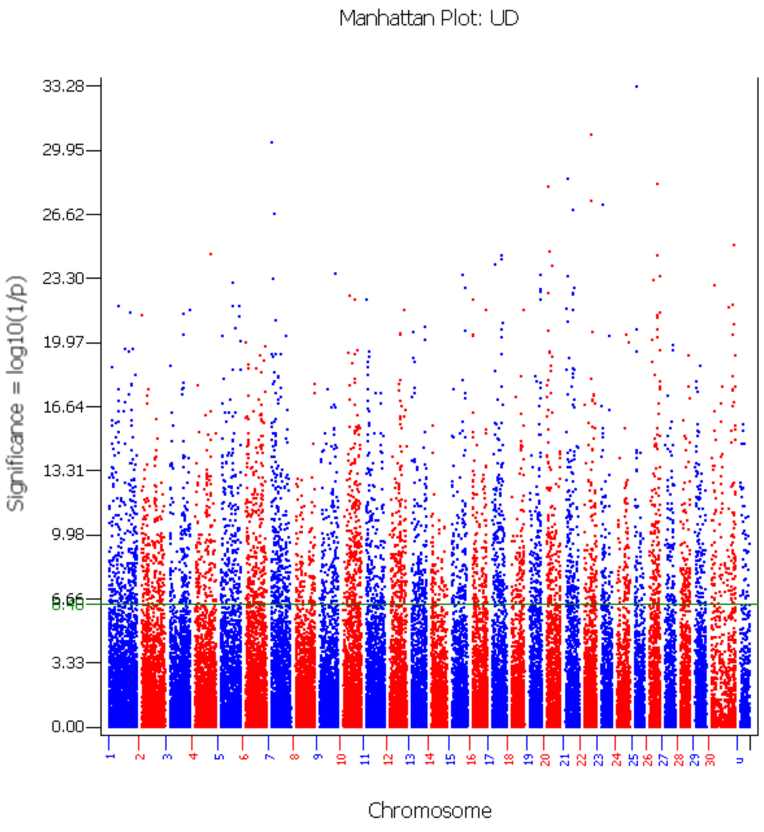
Manhattan Plot: UD
Significance = log10(1/p)
33.28
29.95
26.62
23.30
19.97
16.64
13.31
9.98
6.66
6.48
3.33
0.00
1 2 3 4 5 6 7 8 9 10 11 12 13 14 15 16 17 18 19 20 21 22 23 24 25 26 27 28 29 30 u
Chromosome

Manhattan Plot: UC

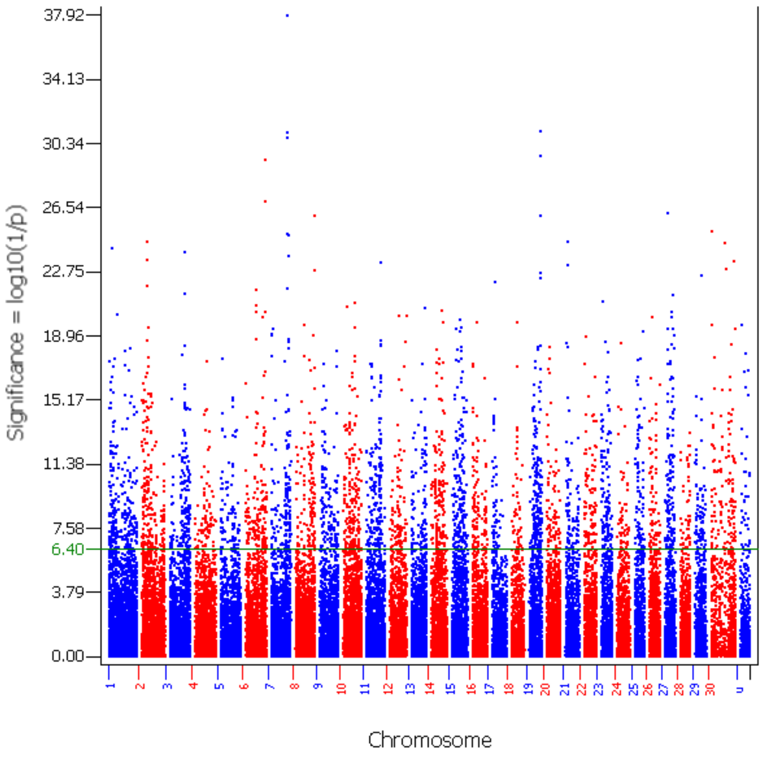

Manhattan Plot: FTP

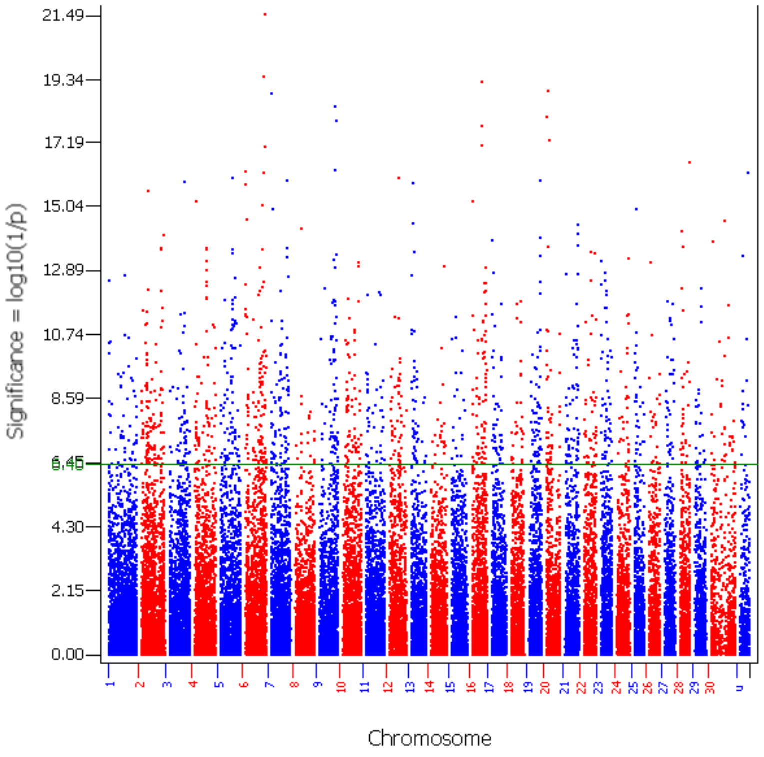

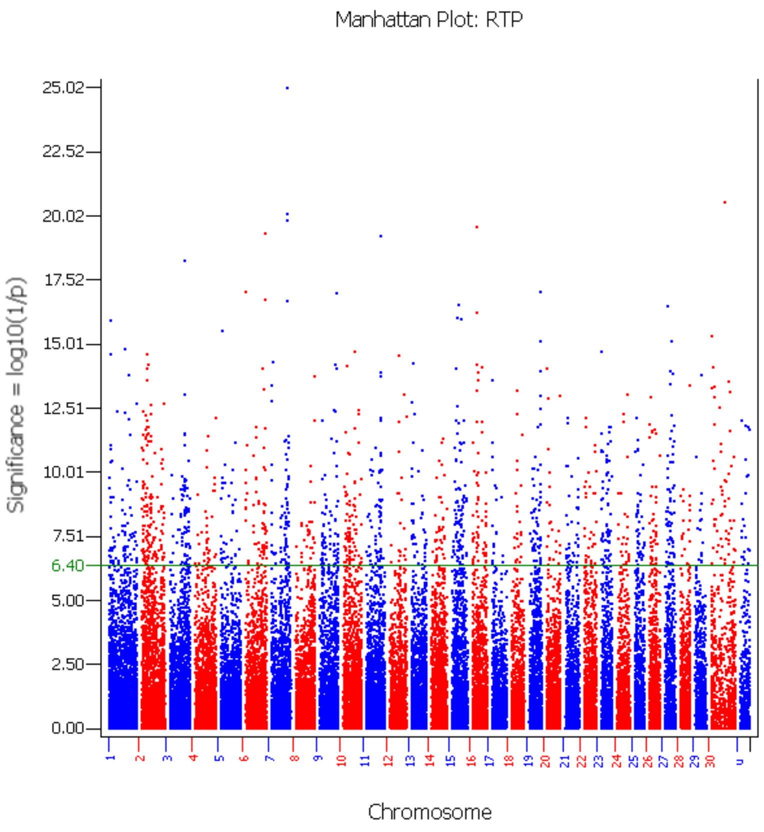
Manhattan Plot: RTP
Significance = log10(1/p)
25.02
22.52
20.02
17.52
15.01
12.51
10.01
7.51
6.40
5.00
2.50
0.00
1 2 3 4 5 6 7 8 9 10 11 12 13 14 15 16 17 18 19 20 21 22 23 24 25 26 27 28 29 30 u
Chromosome

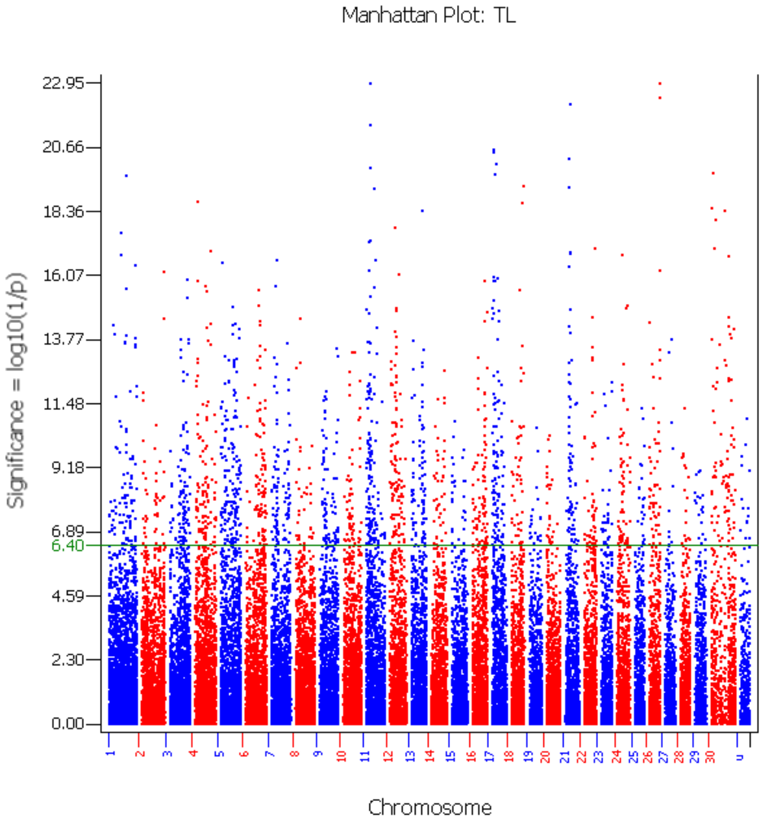
Manhattan Plot: TL
Significance = log10(1/p)
22.95
20.66
18.36
16.07
13.77
11.48
9.18
6.89
6.40
4.59
2.30
0.00
1 2 3 4 5 6 7 8 9 10 11 12 13 14 15 16 17 18 19 20 21 22 23 24 25 26 27 28 29 30 u
Chromosome

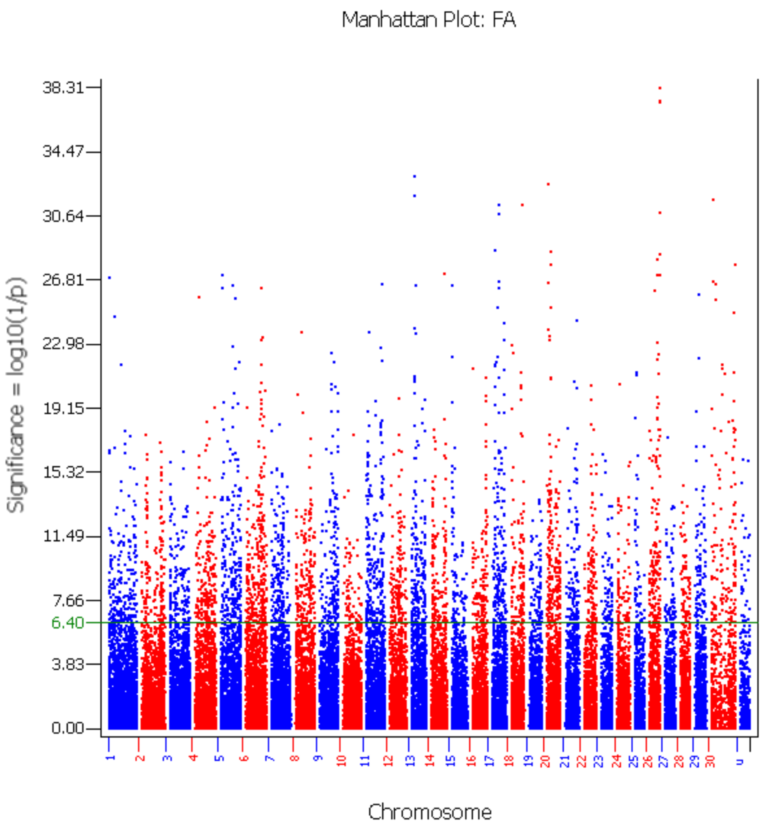
Manhattan Plot: FA
Significance = log10(1/p)
38.31
34.47
30.64
26.81
22.98
19.15
15.32
11.49
7.66
6.40
3.83
0.00
1 2 3 4 5 6 7 8 9 10 11 12 13 14 15 16 17 18 19 20 21 22 23 24 25 26 27 28 29 30 u
Chromosome

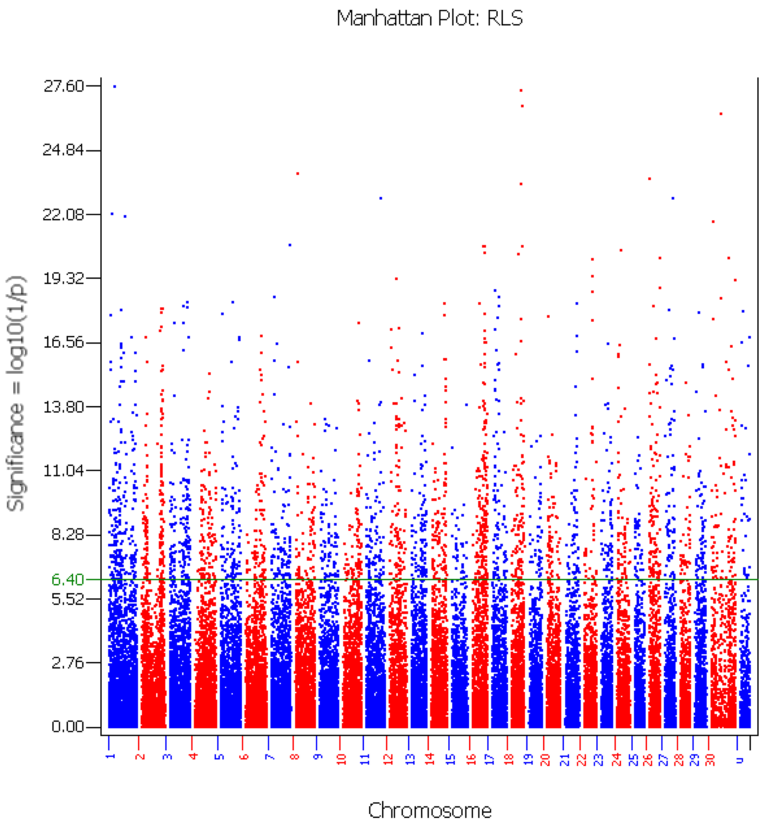
Manhattan Plot: RLS
Significance = log10(1/p)
27.60
24.84
22.08
19.32
16.56
13.80
11.04
8.28
6.40
5.52
2.76
0.00
1 2 3 4 5 6 7 8 9 10 11 12 13 14 15 16 17 18 19 20 21 22 23 24 25 26 27 28 29 30 u
Chromosome

Manhattan Plot: RLR

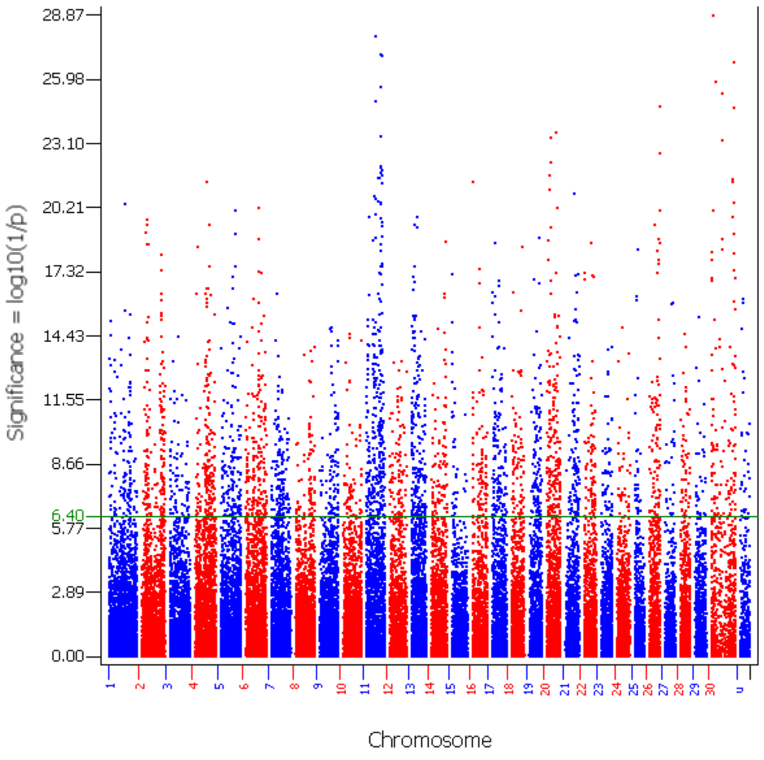

Manhattan Plot: FL

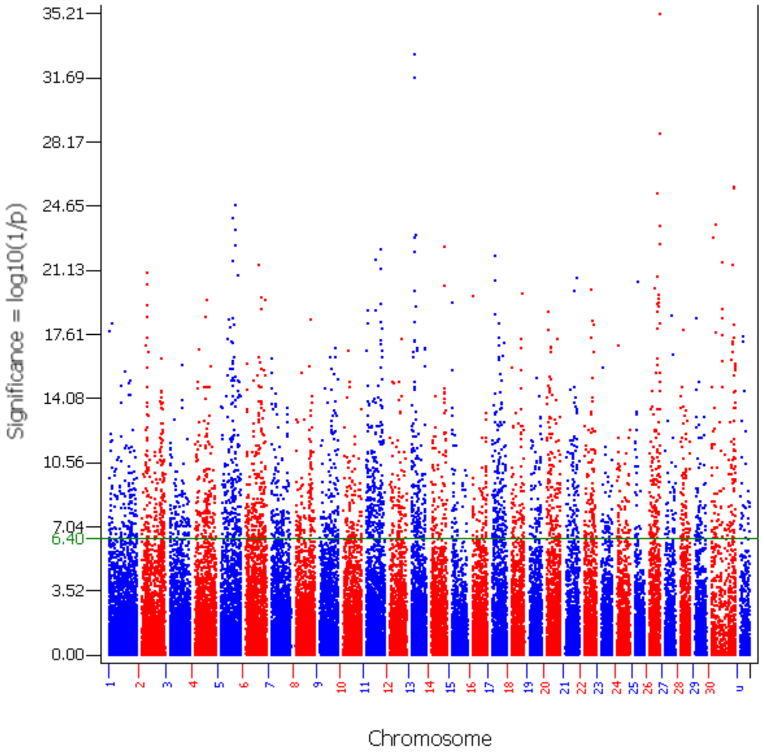

Manhattan Plot: FS

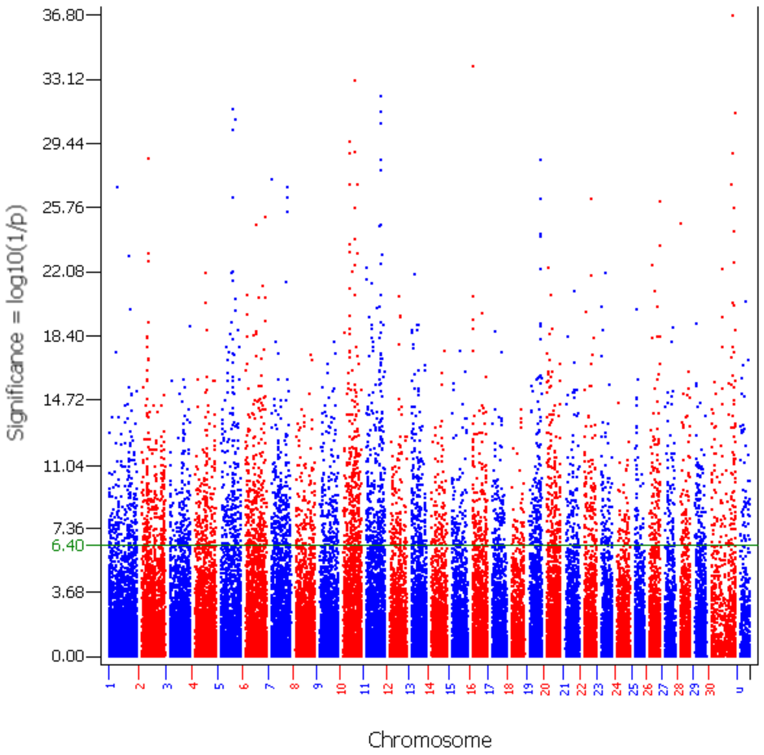

Supplement: Additional file 1 — Figure S1. Global view of P-values of 45,878 SNP effects per trait for 31 production, health, reproduction and body conformation traits by Mahattan plot. MY, milk yield; FY, fat yield; PY, protein yield; FPC, fat percentage; PPC, protein percentage; SCS, somatic cell score; DPR, daughter pregnancy rate; PL, productive life; SCE, sire calving ease; DCE, daughter calving ease; SSB, sire stillbirth; DSB, daughter stillbirth; NM, net merit; STA, stature; STR, strength; BD, body depth; RW, rump width; DF, dairy form; RA, rump angle; FUA, fore udder attachment; RUH, rear udder height; UD, udder depth; UC, udder cleft; FTP, front teat placement; RTP, rear teat placement; TL, teat length; FA, foot angle; RLS, rear legs (side view); RLR, rear legs (rear view); FL, feet and legs; FS, final score. [file 1471-2164-12-408-S1.PDF]
