# Supplementary figures and images for: Genome-wide association analysis of thirty one production, health, reproduction and body conformation traits in contemporary U.S. Holstein cows
Source: BMC Genomics. 2011 Aug 11;12:408. doi: 10.1186/1471-2164-12-408 (PMC3176260; doi:10.1186/1471-2164-12-408)

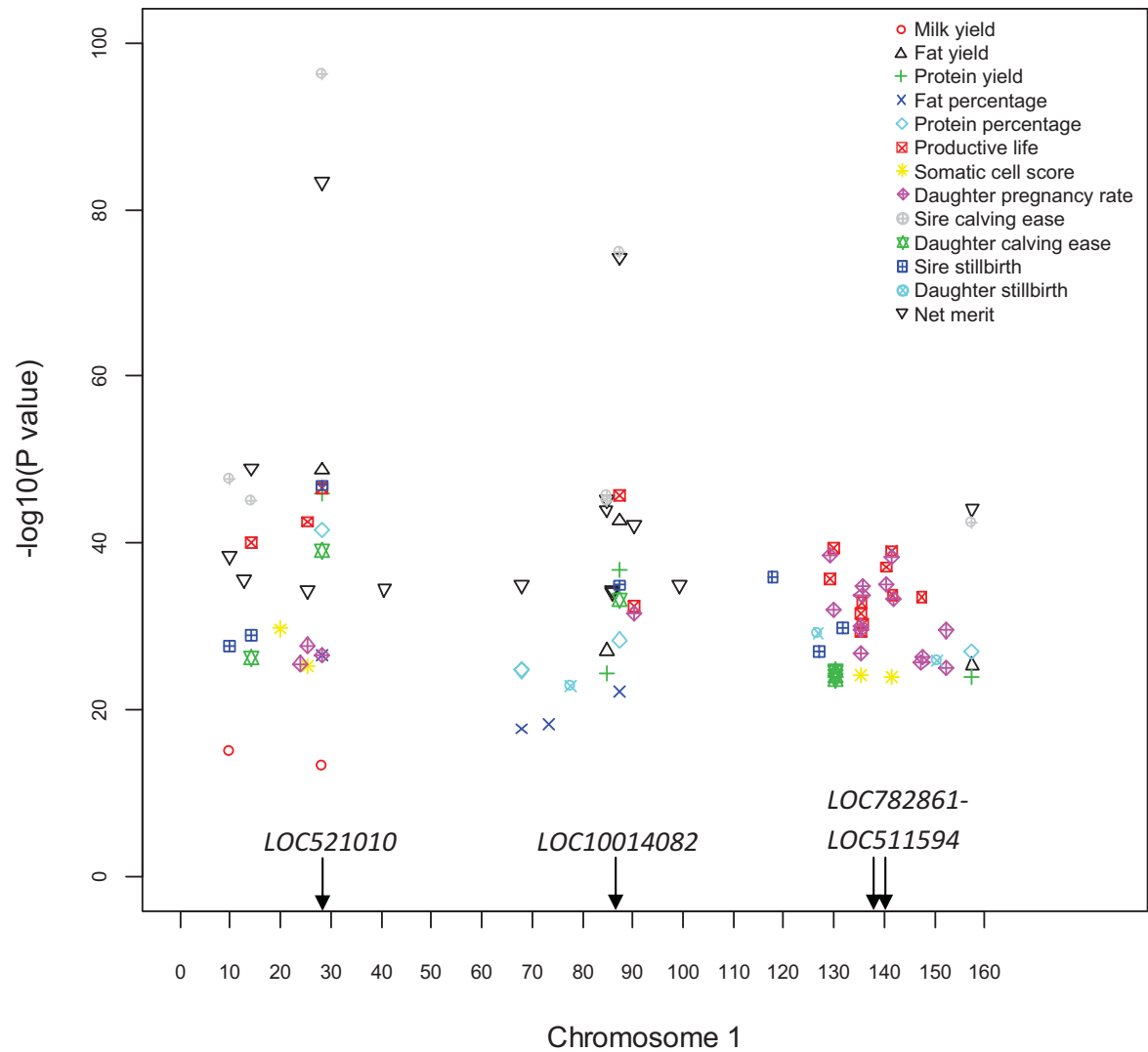

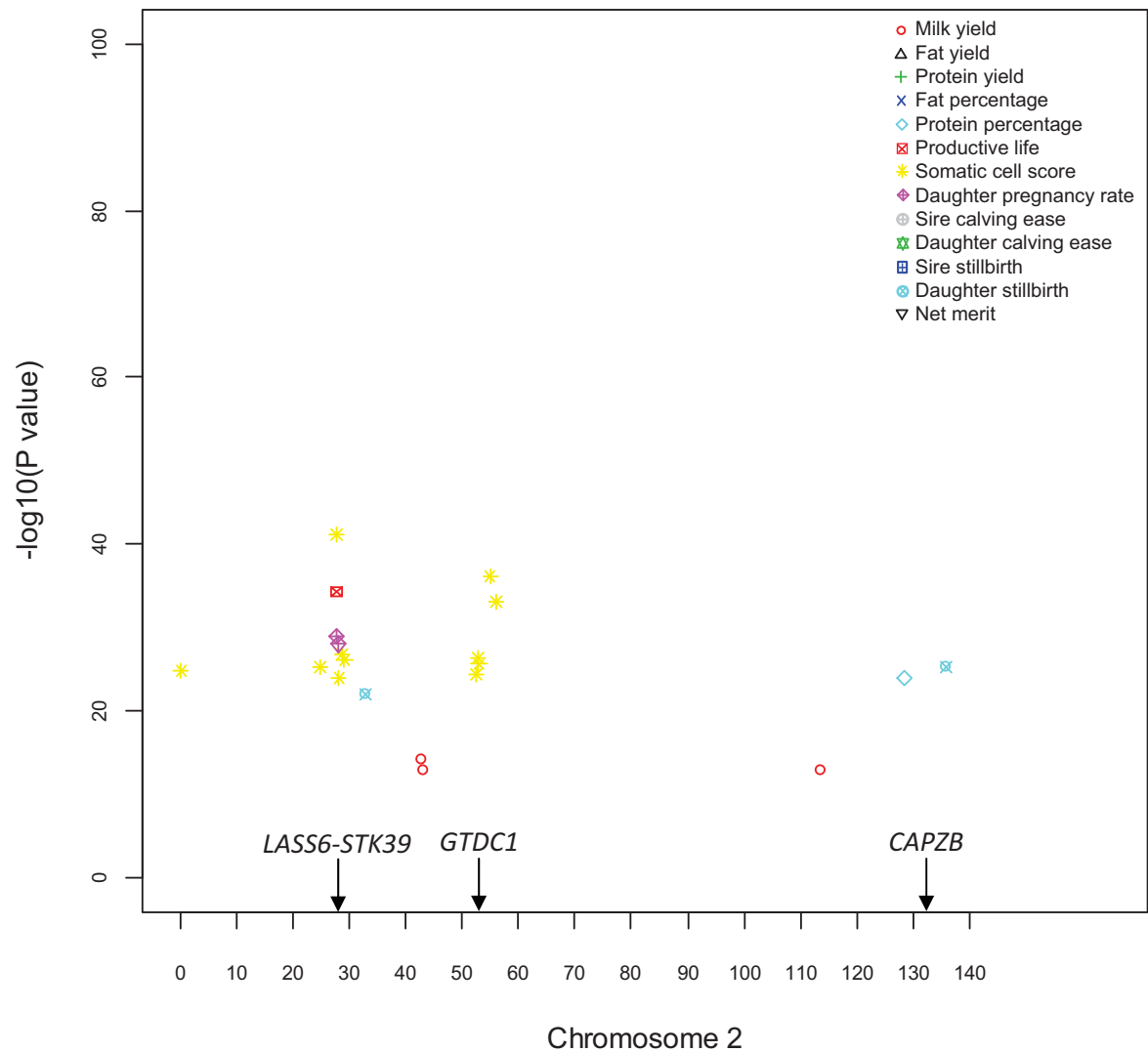

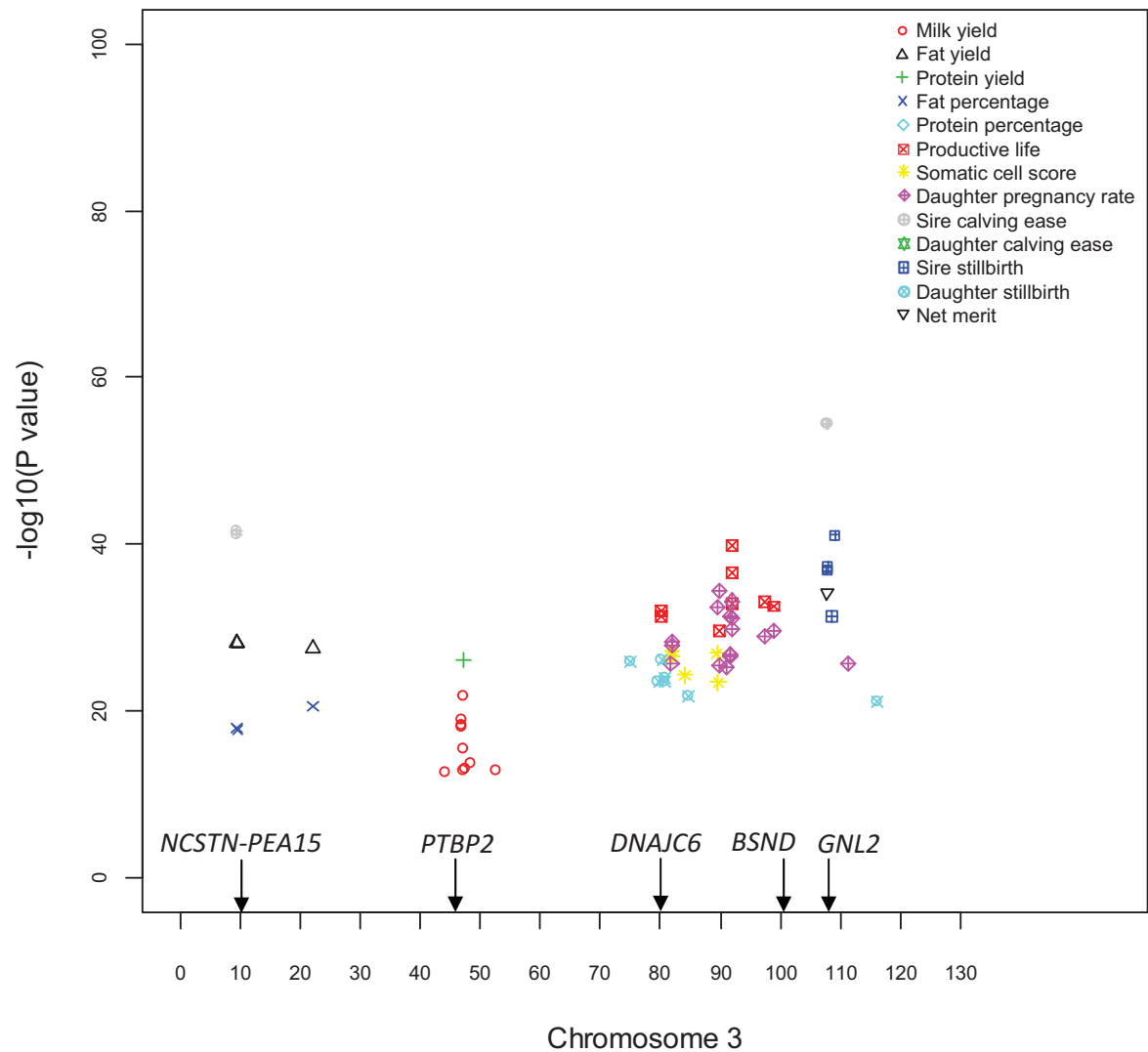

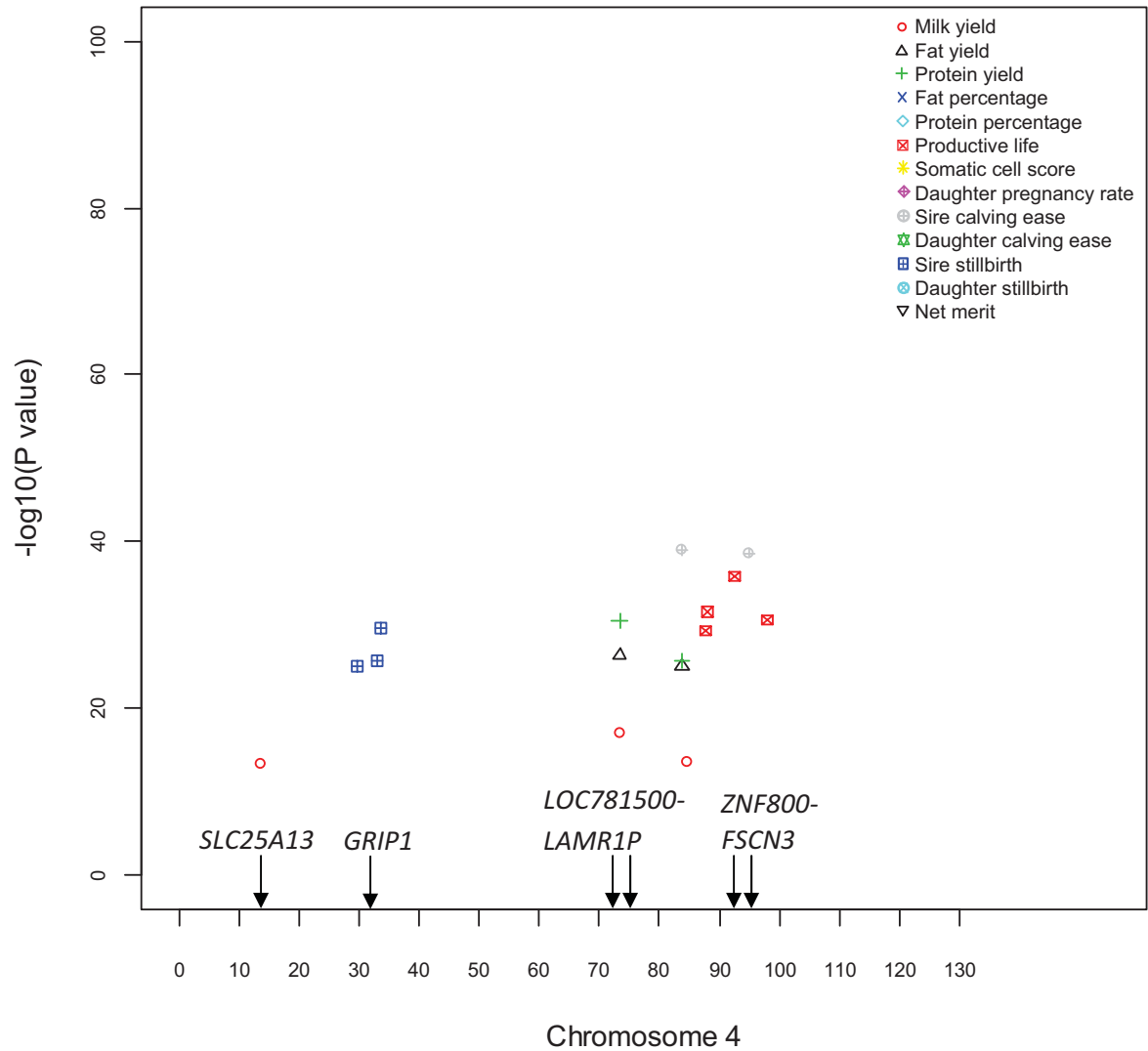

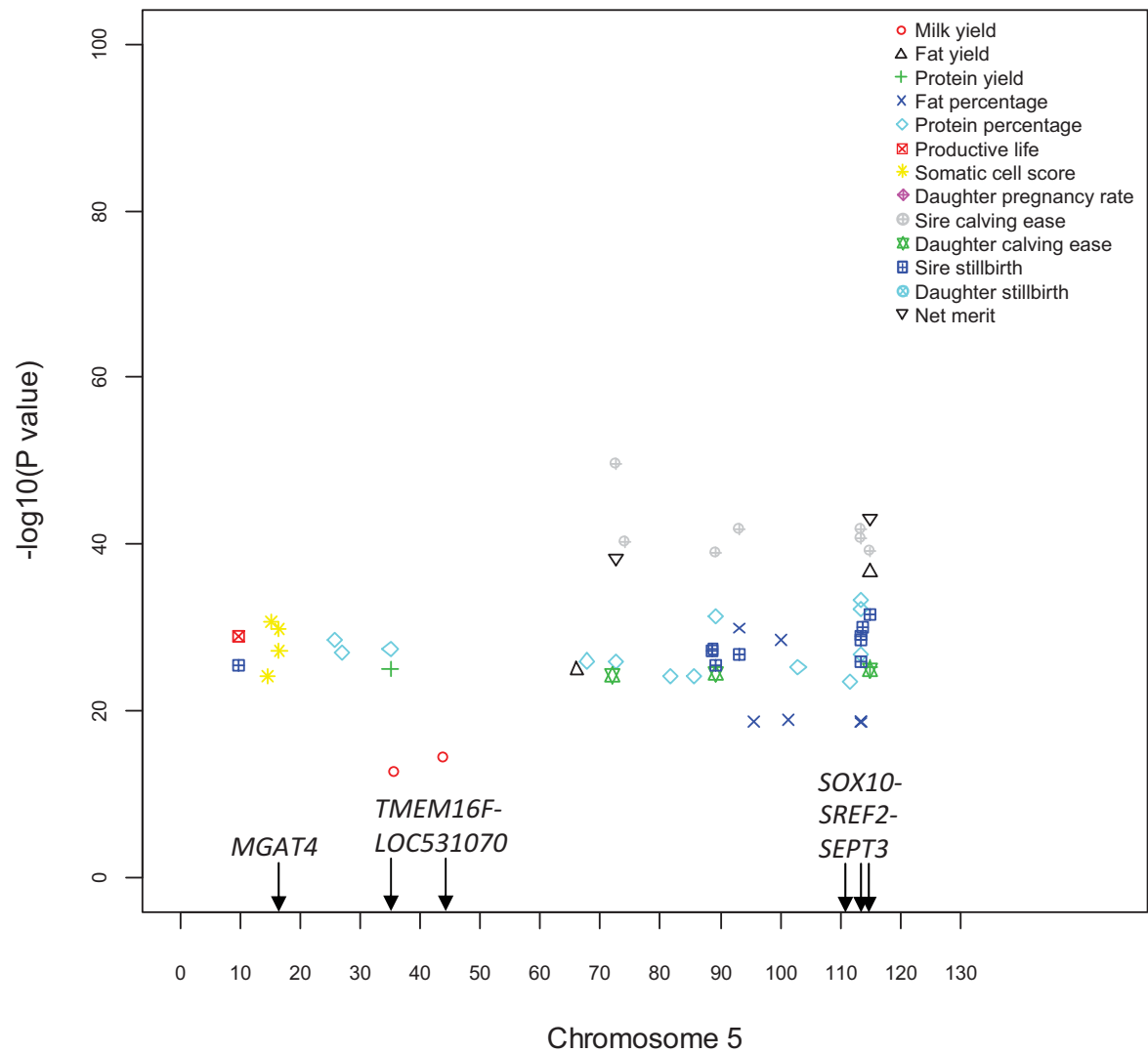

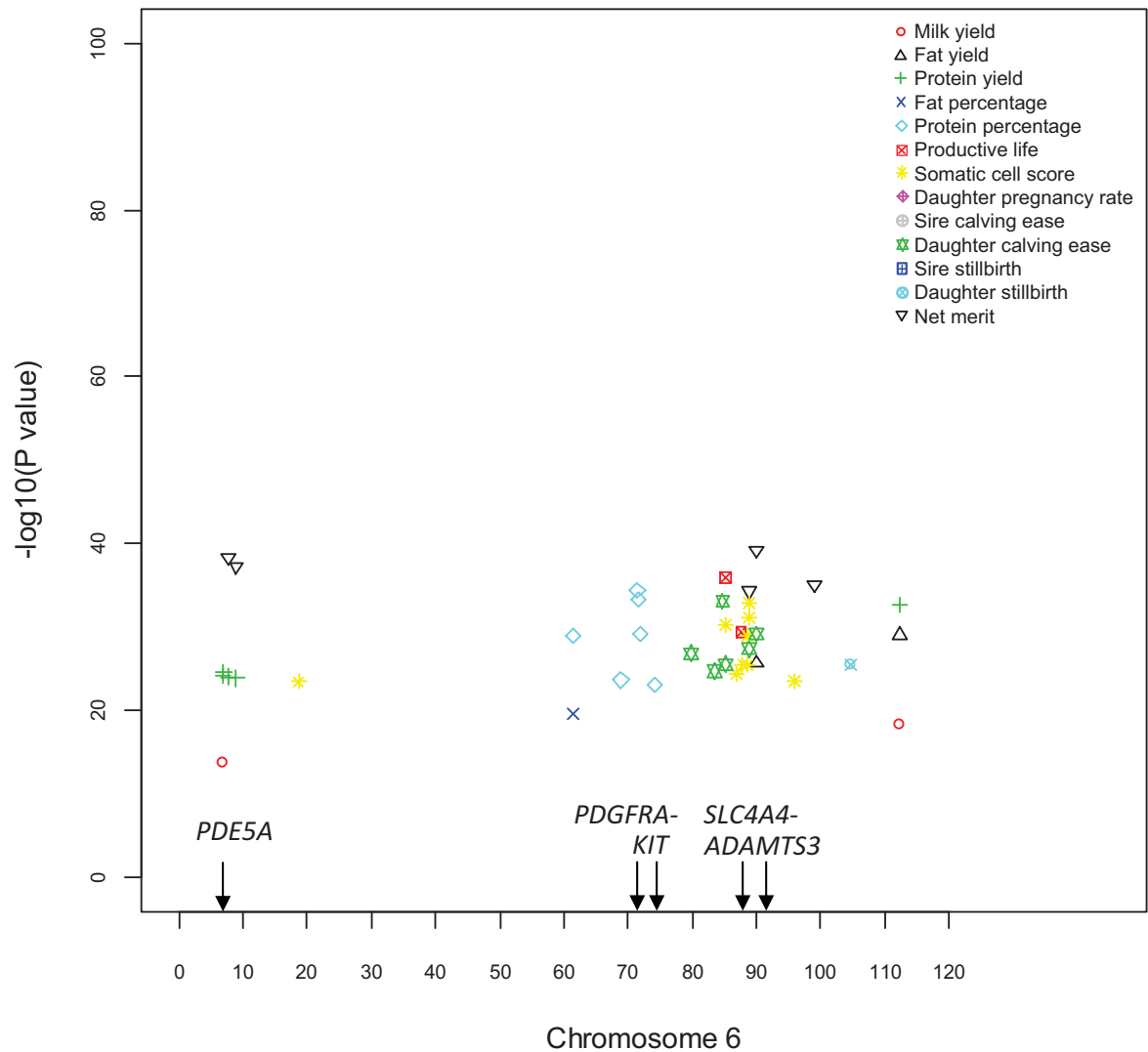

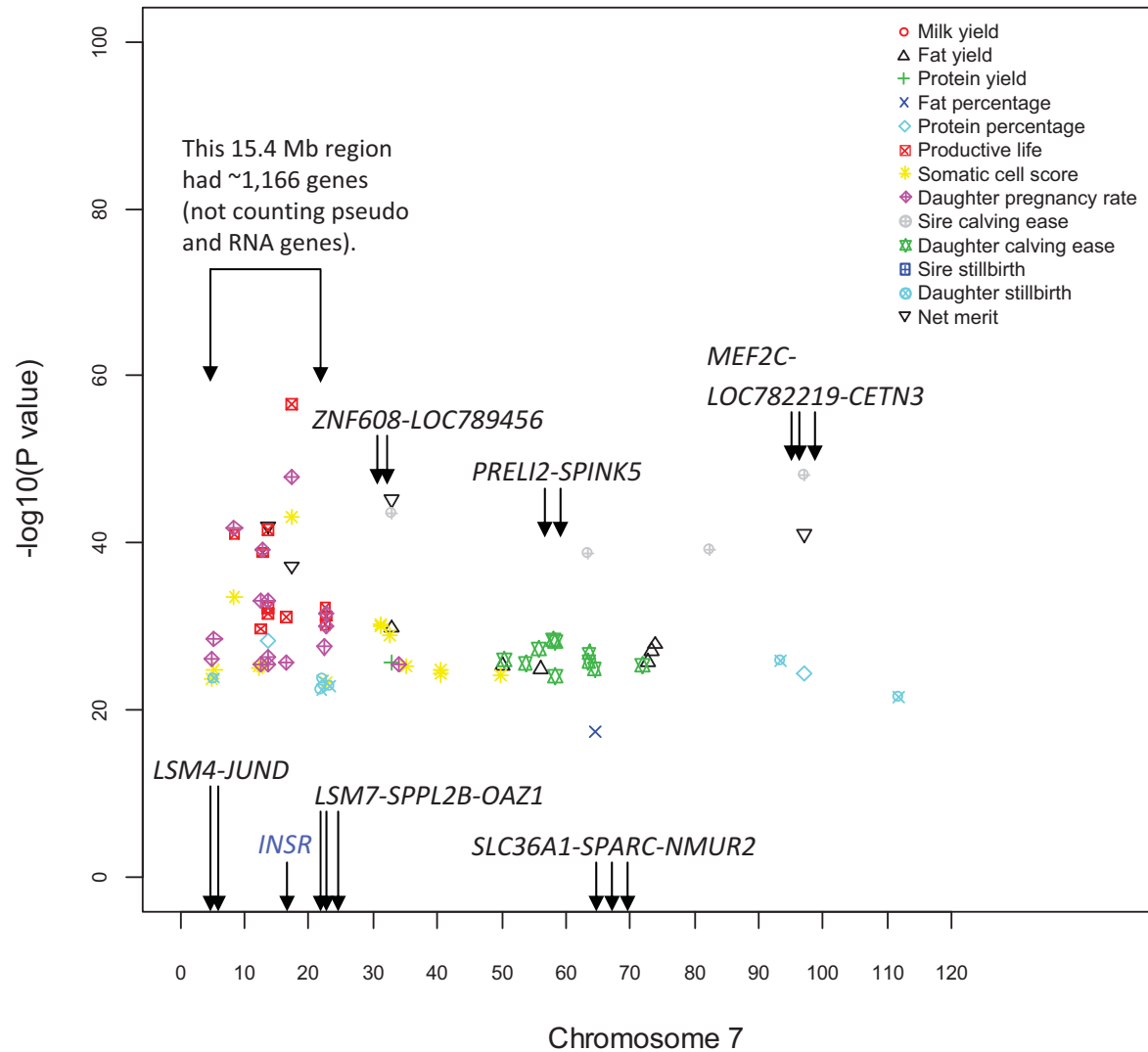

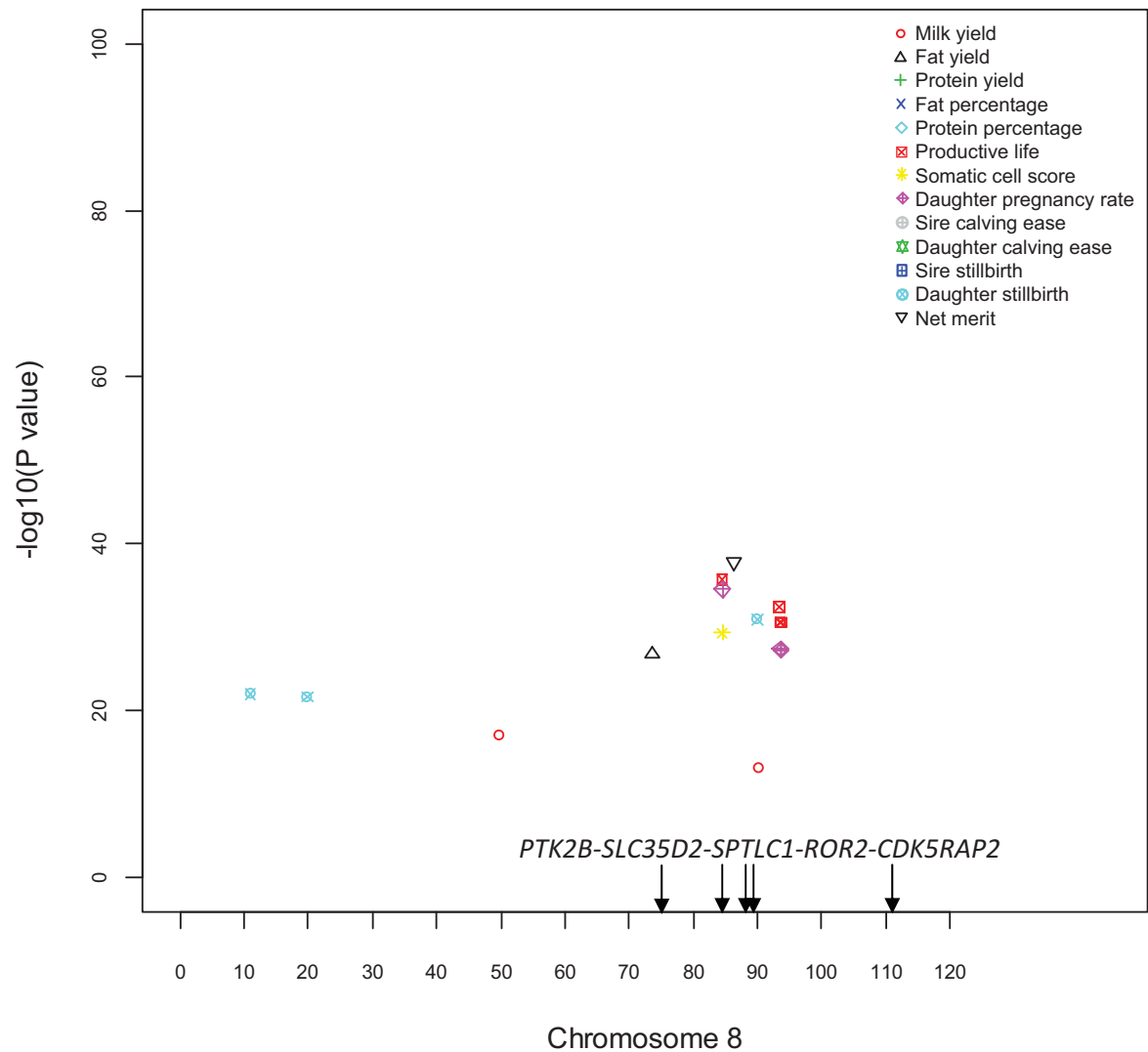

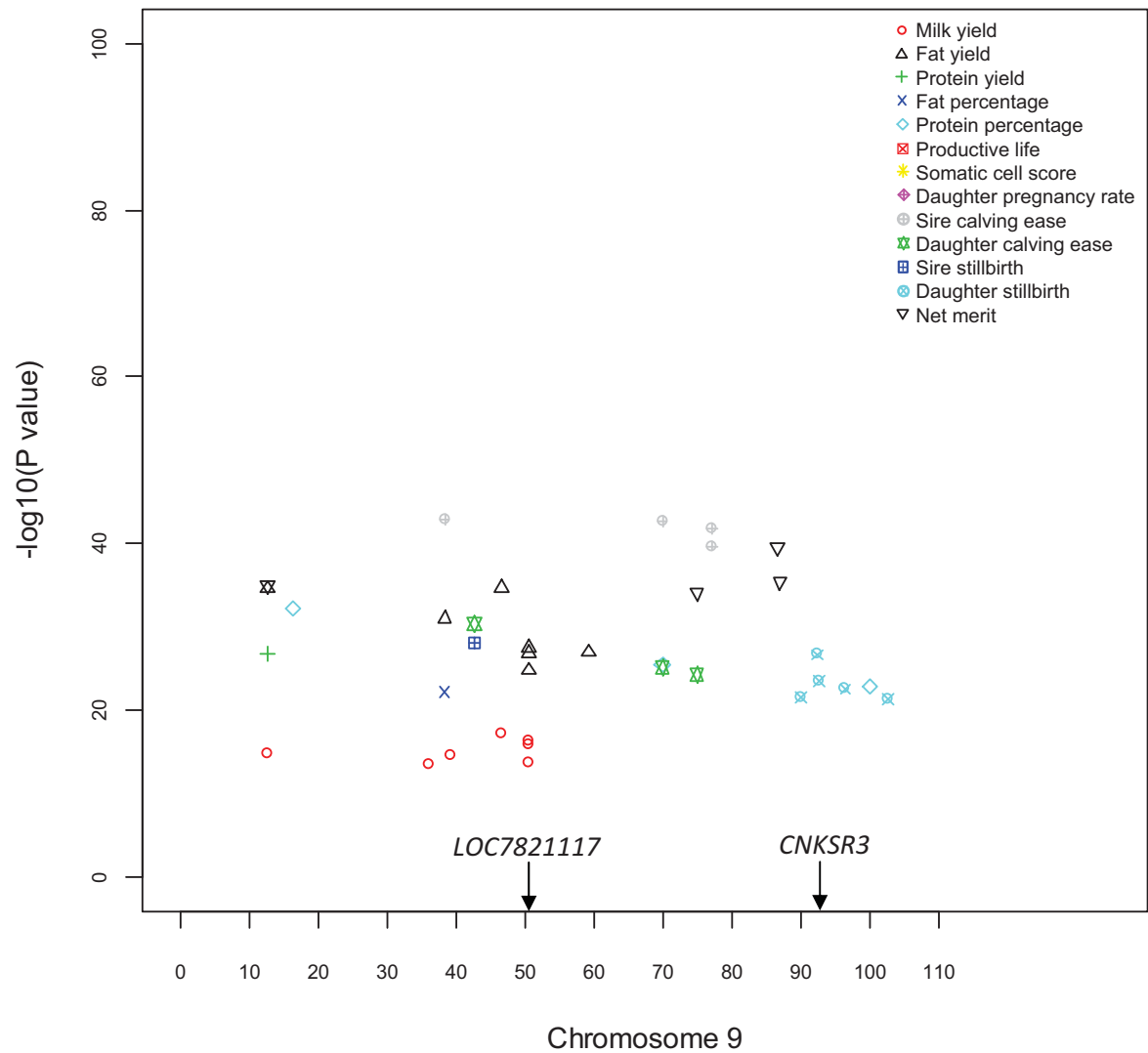

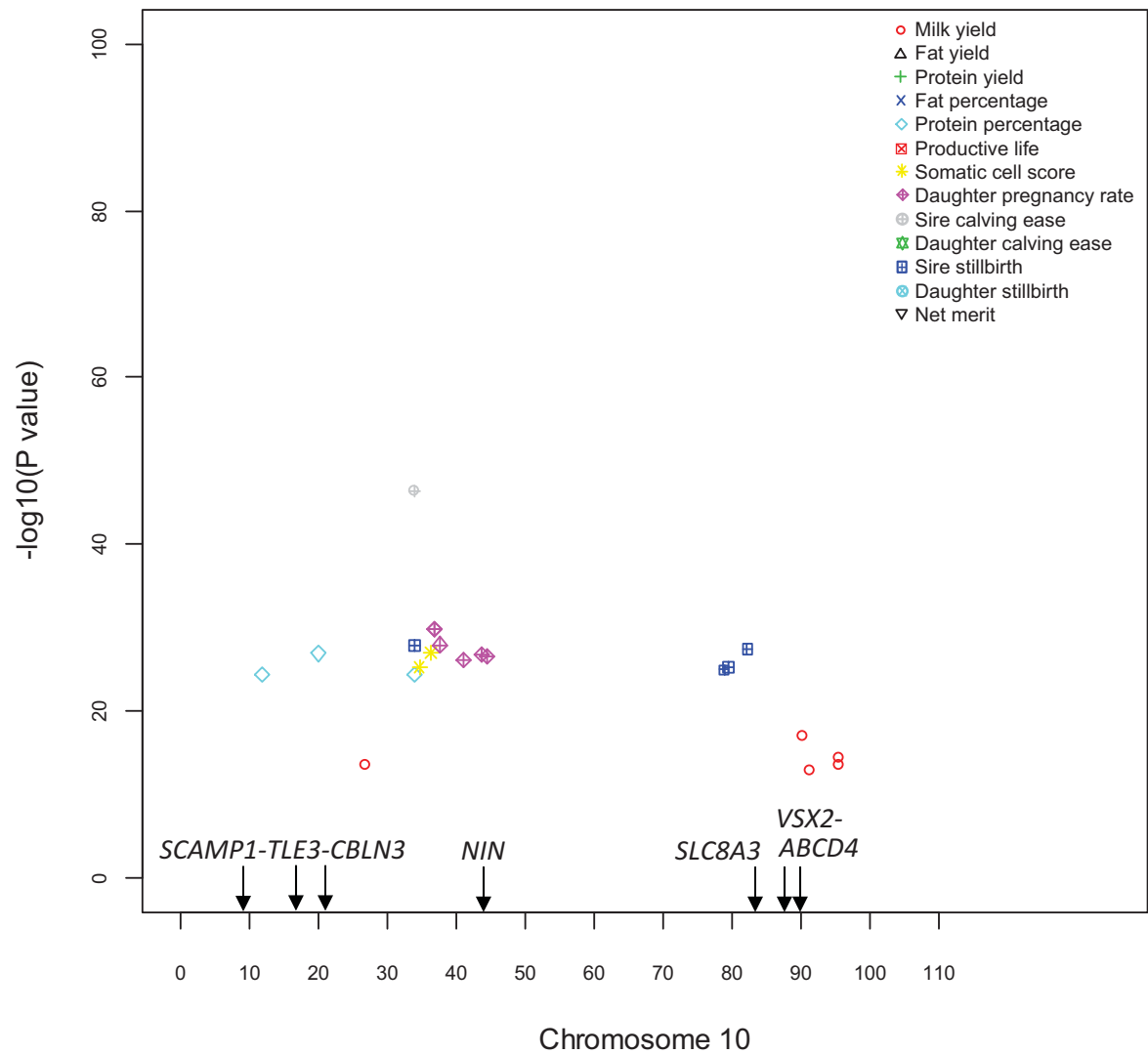

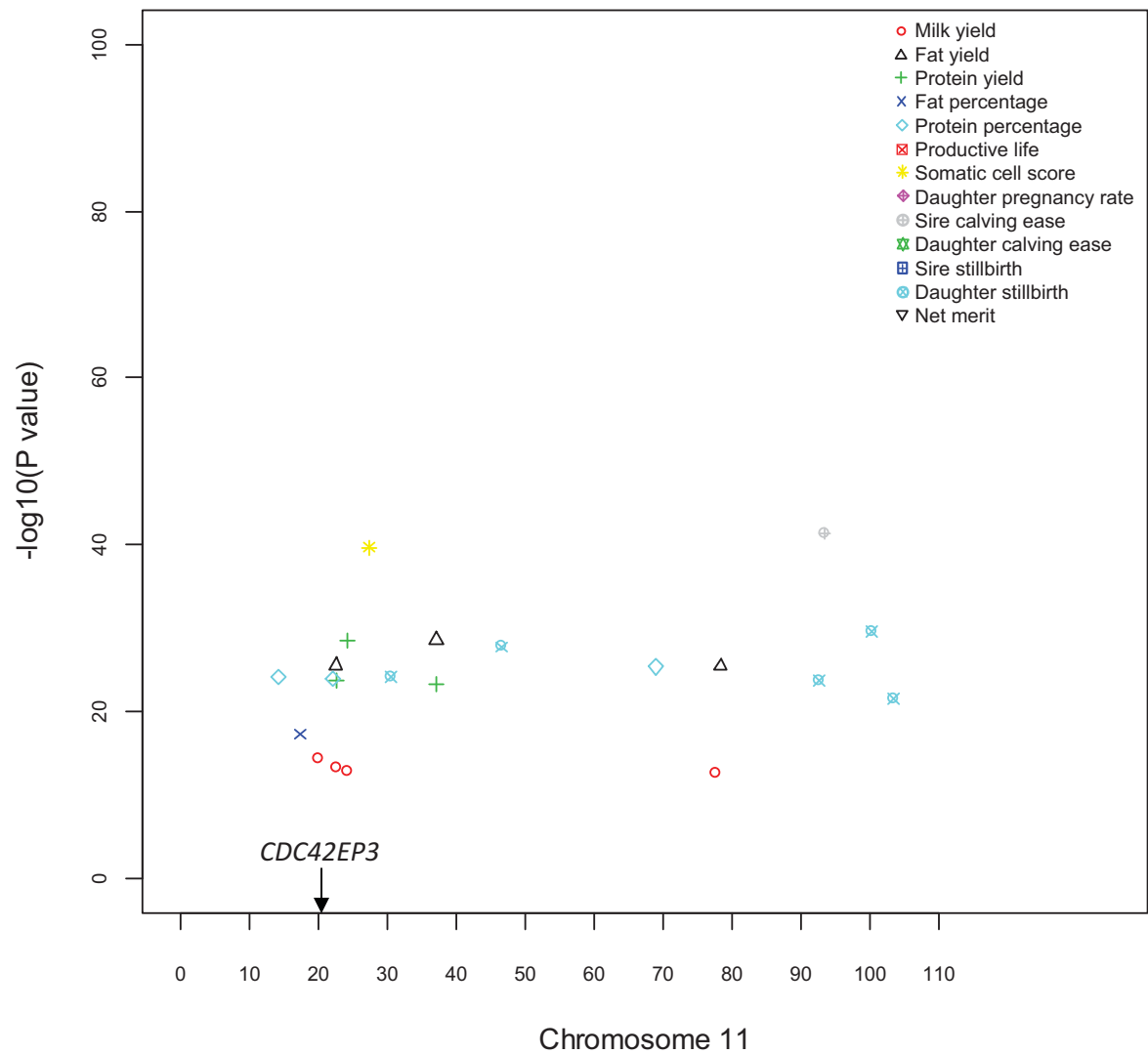

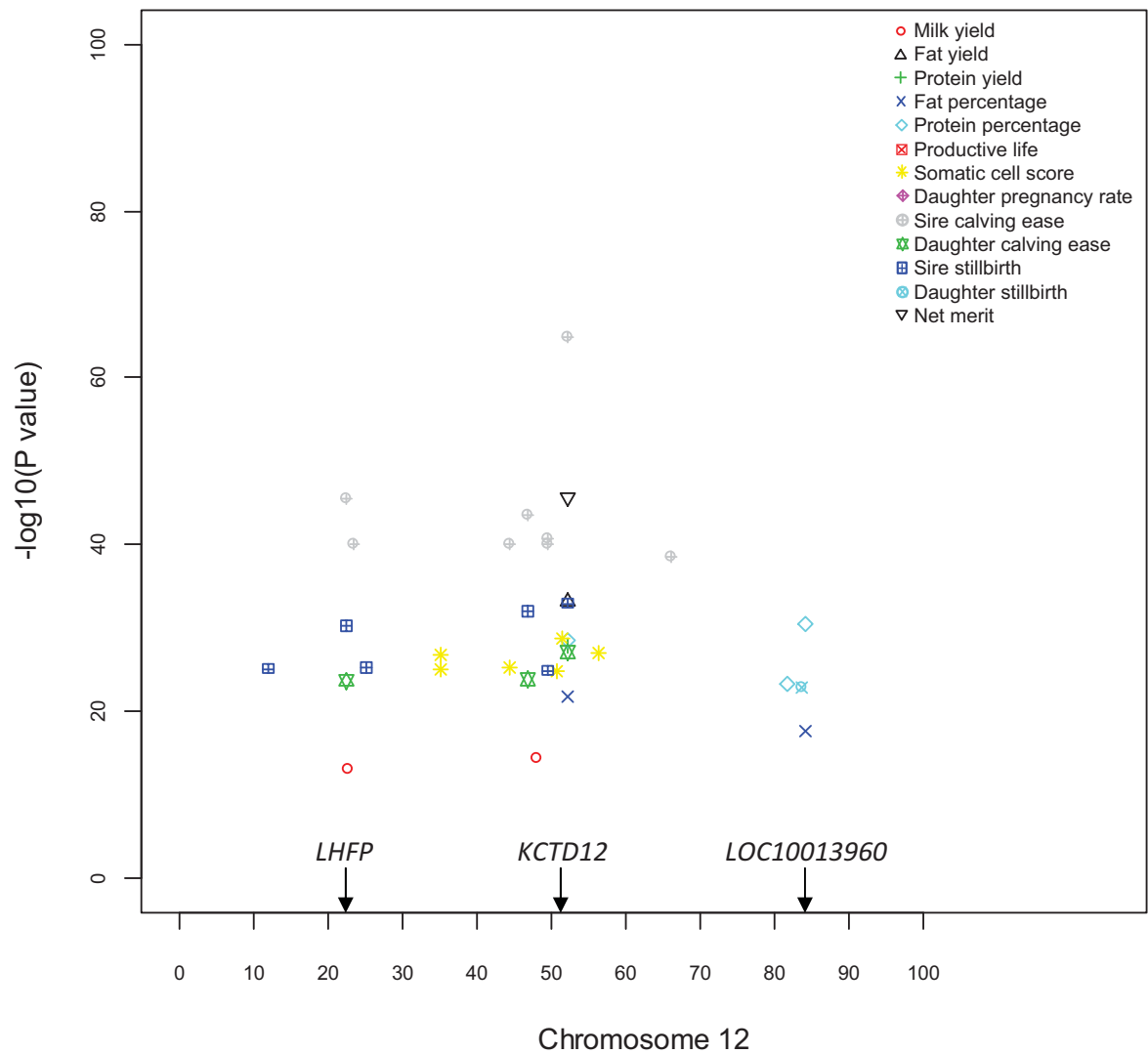

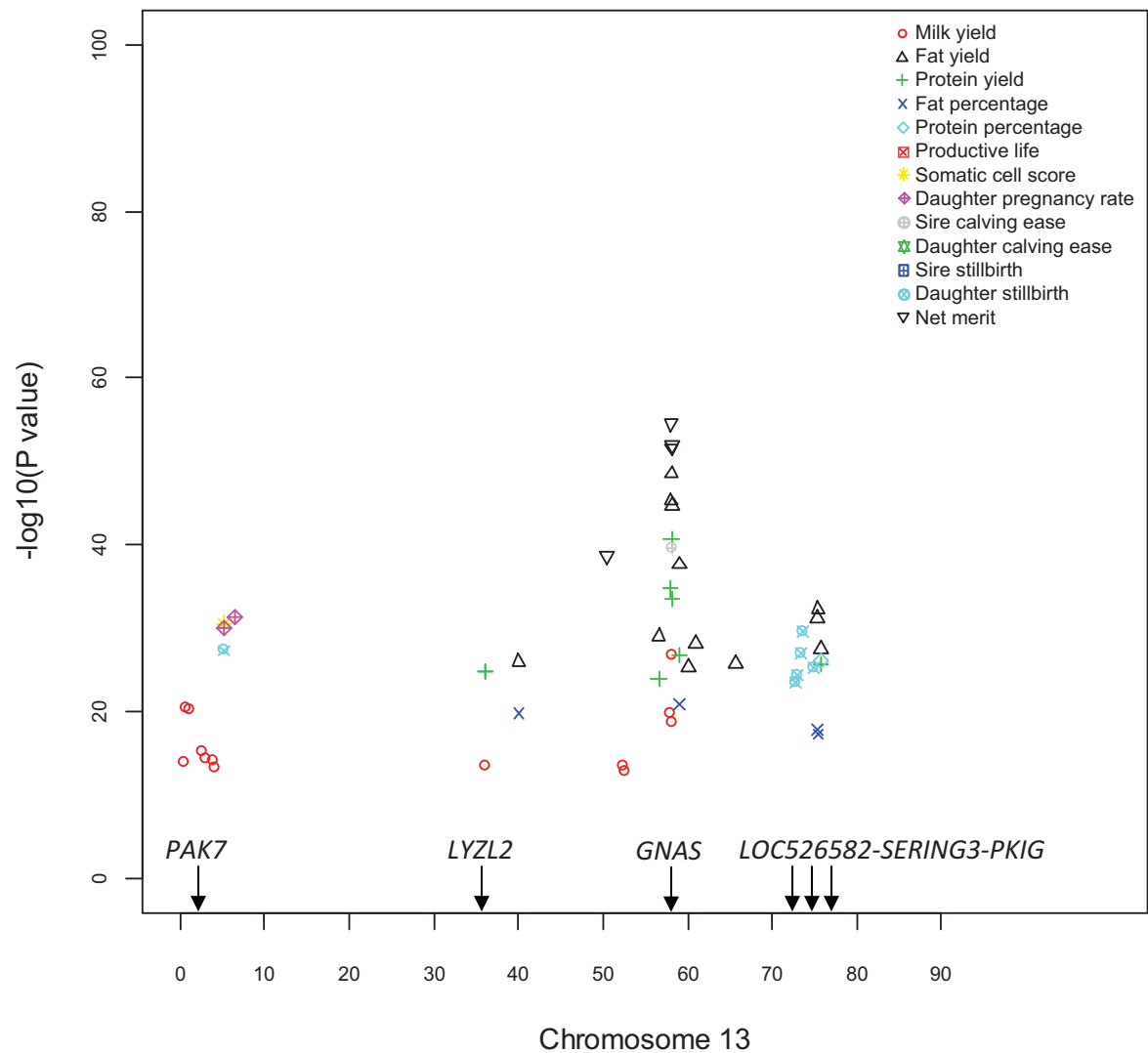

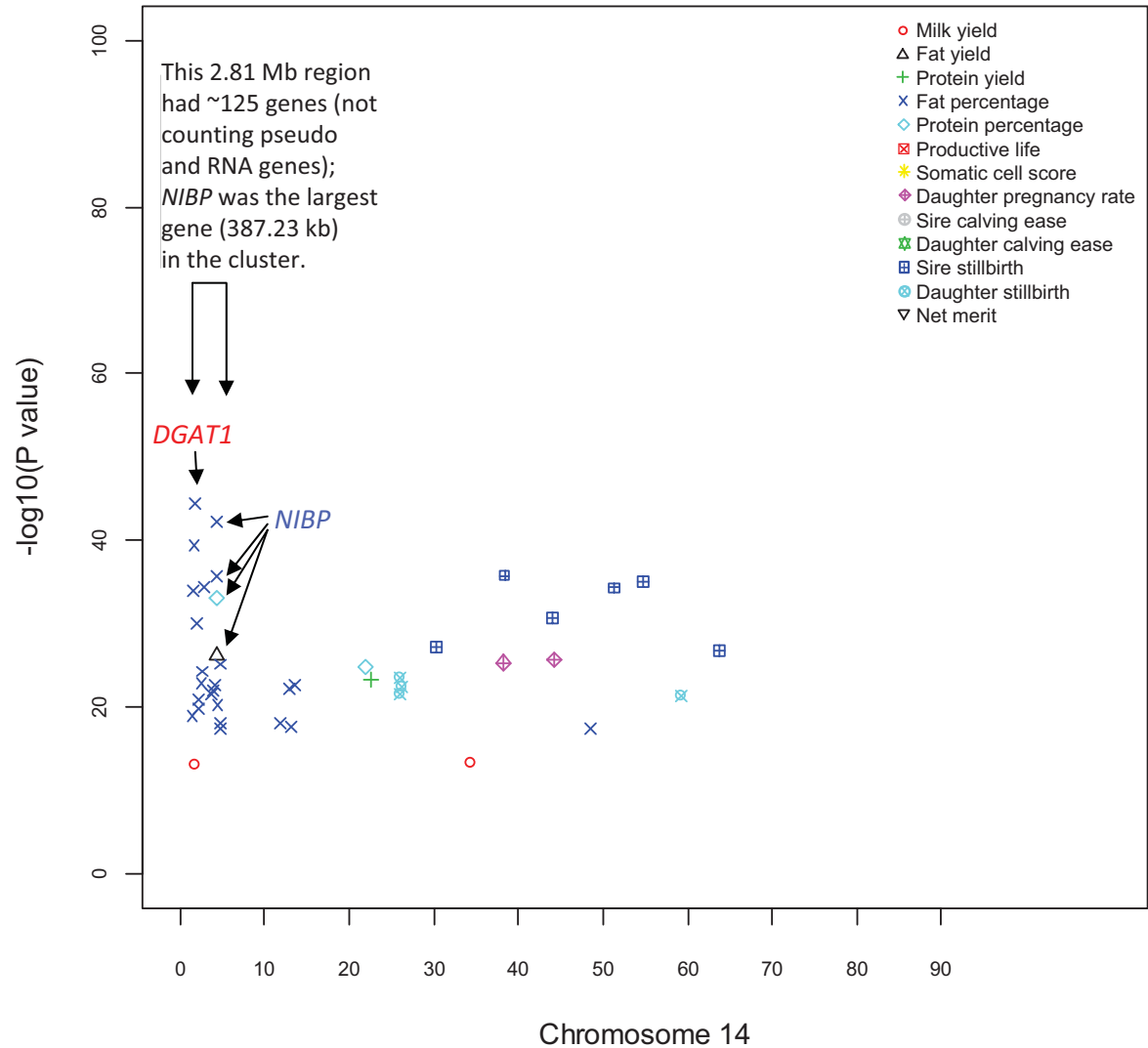

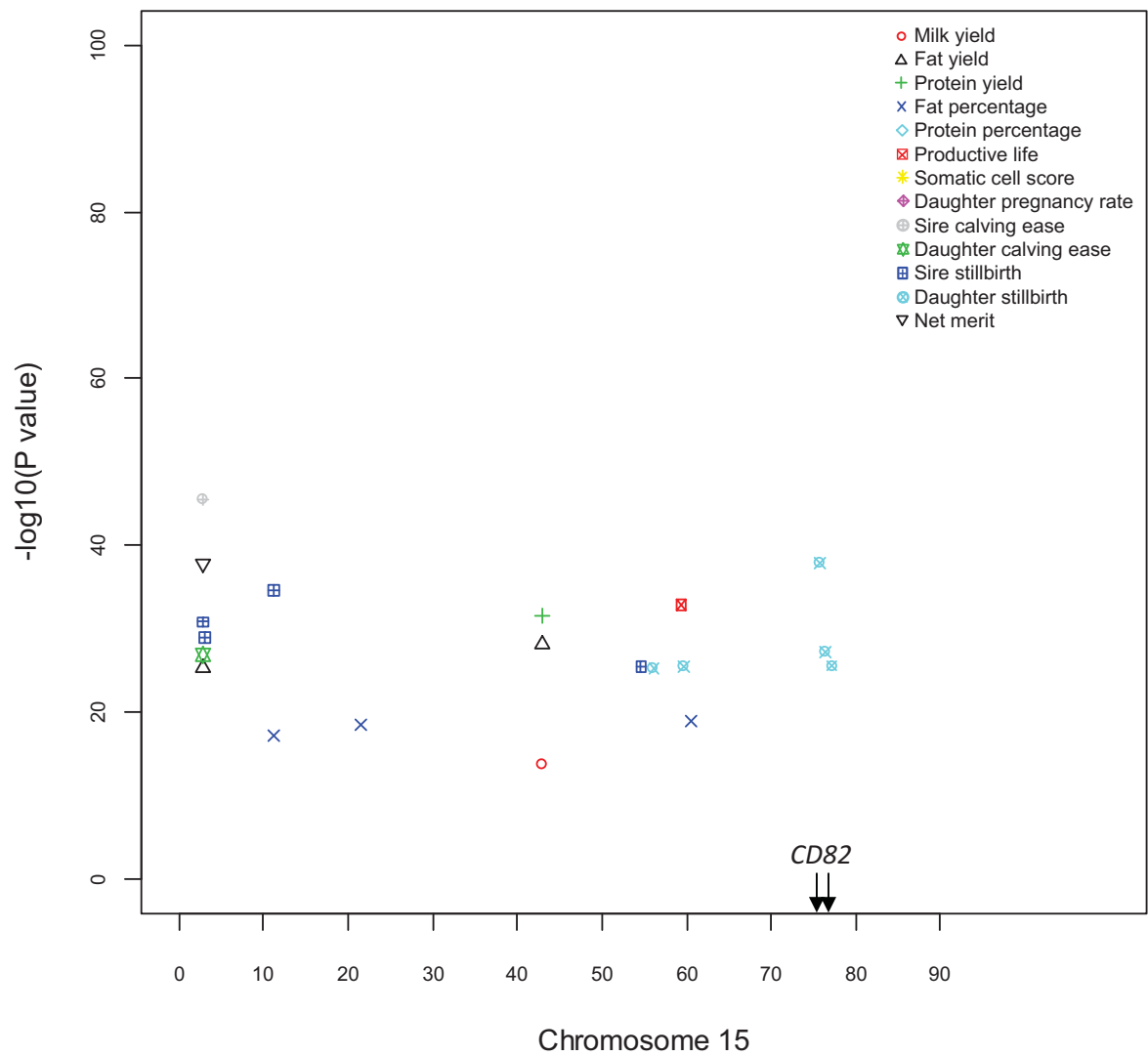

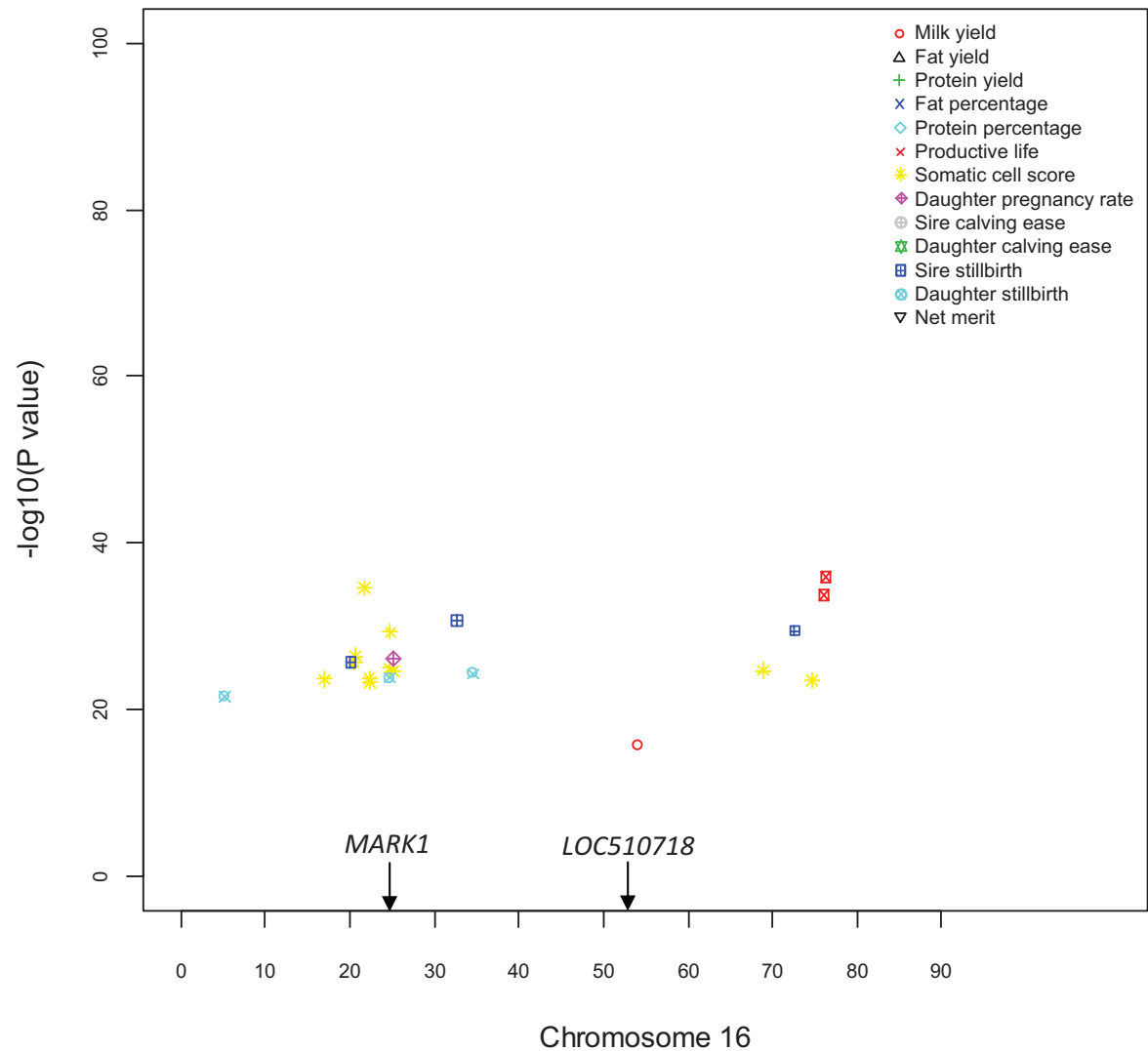

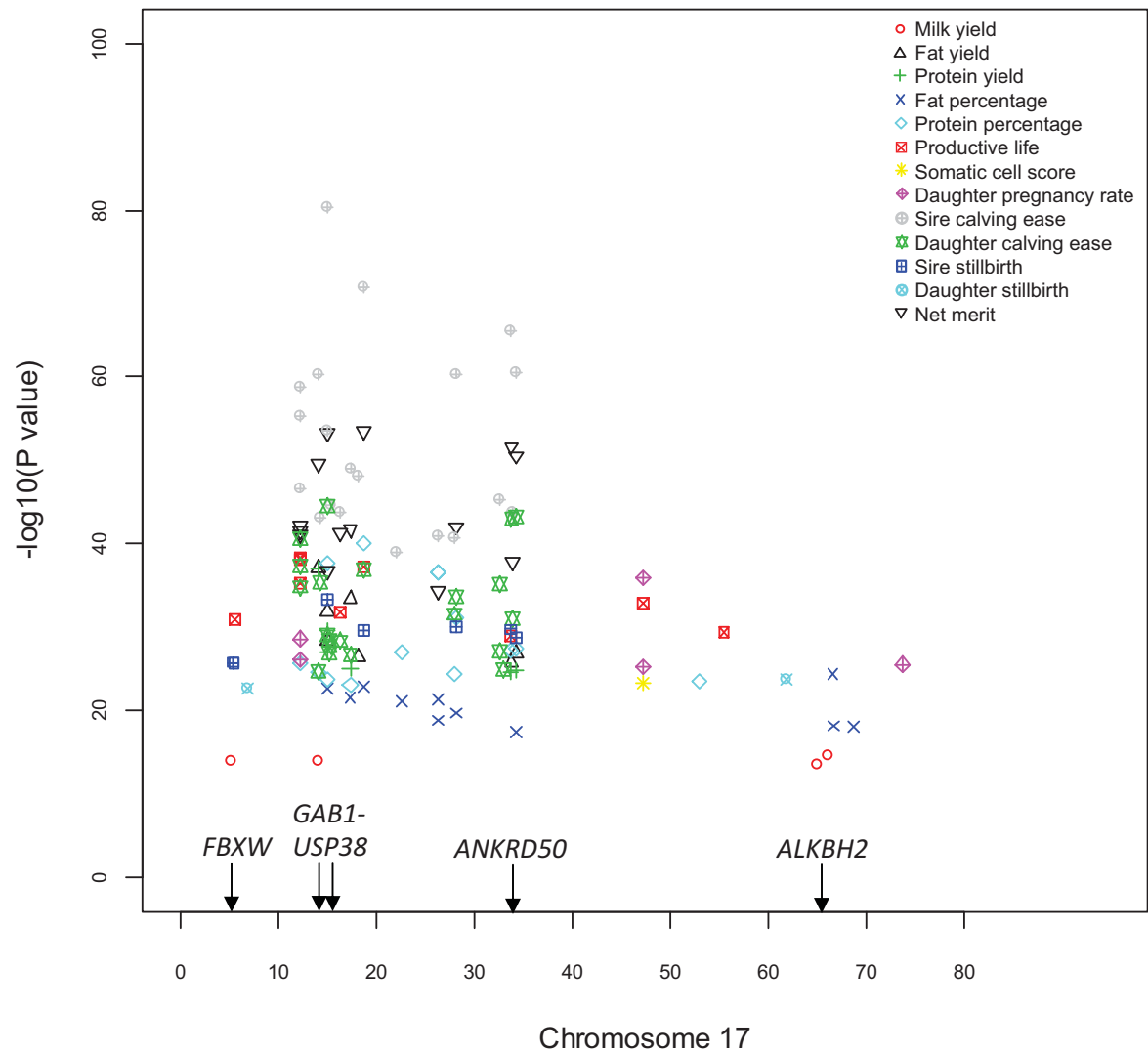

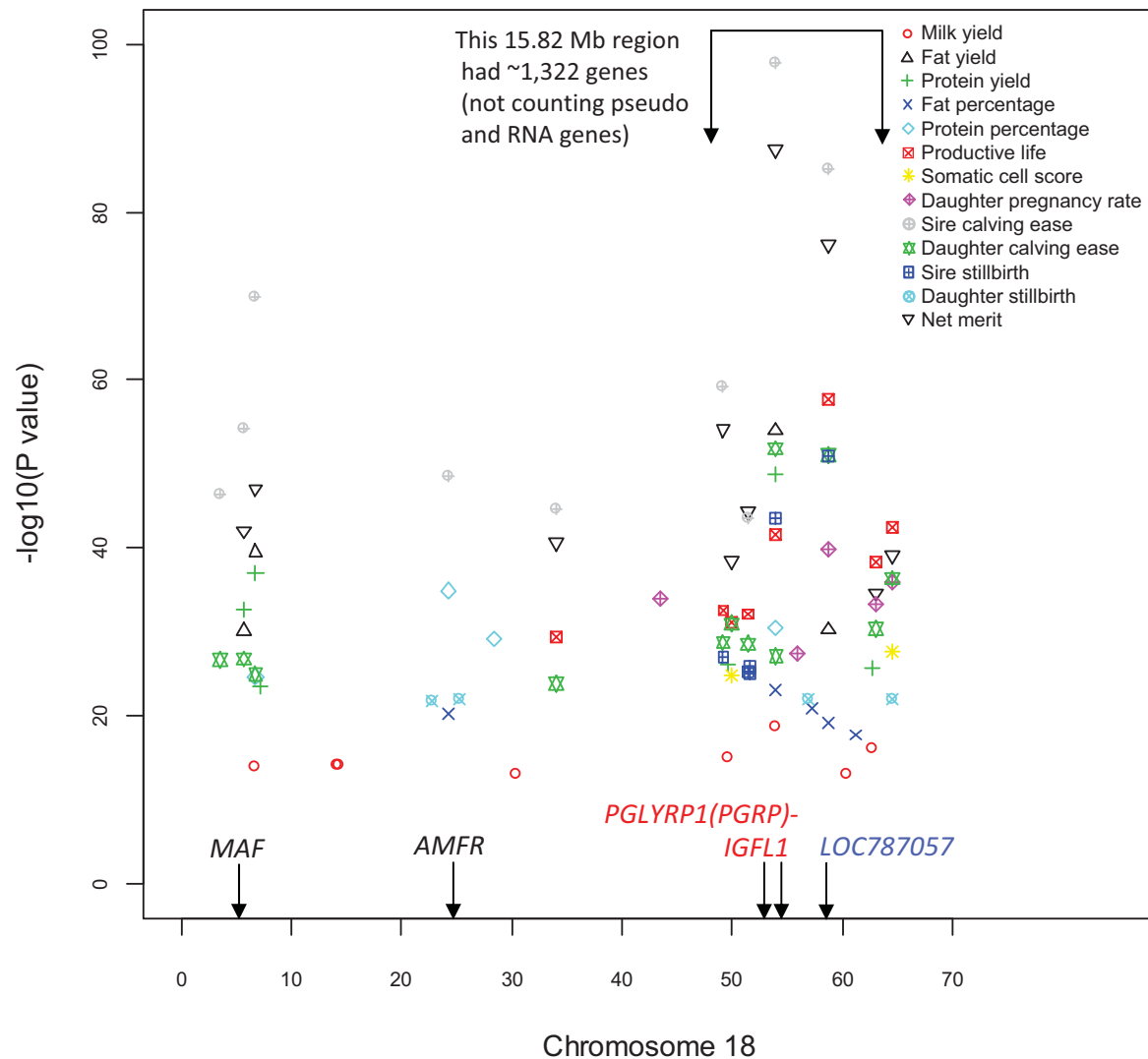

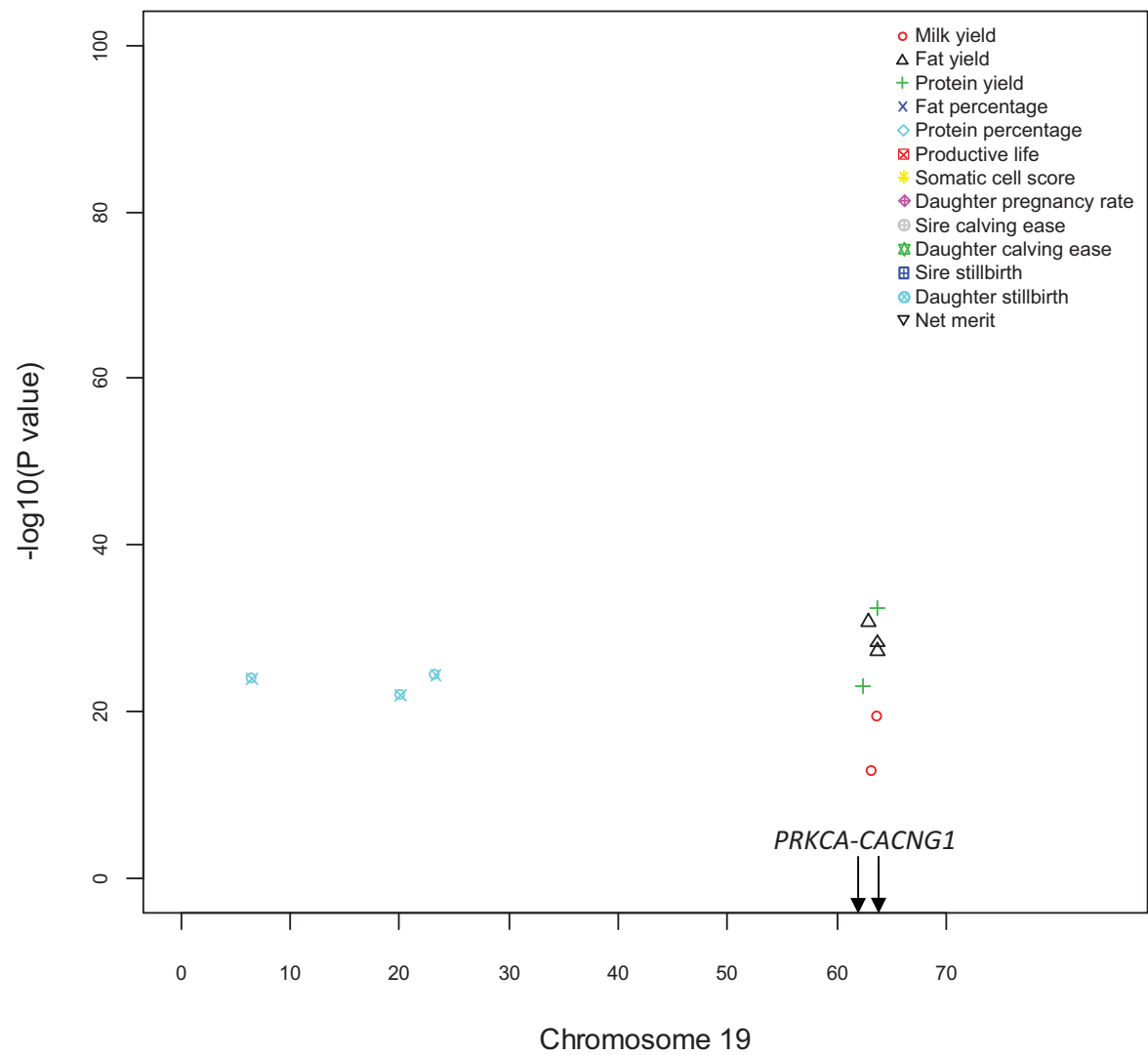

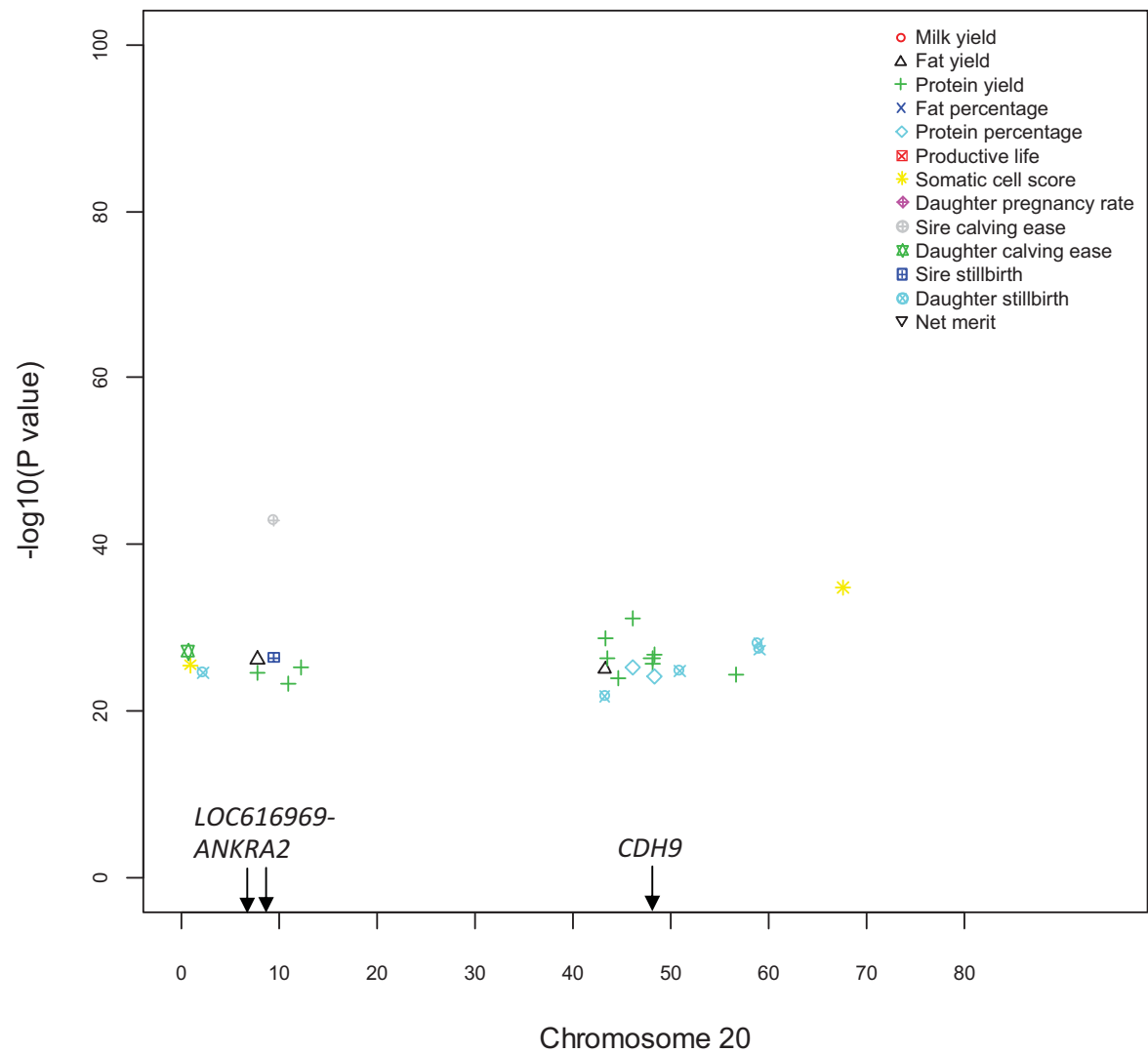

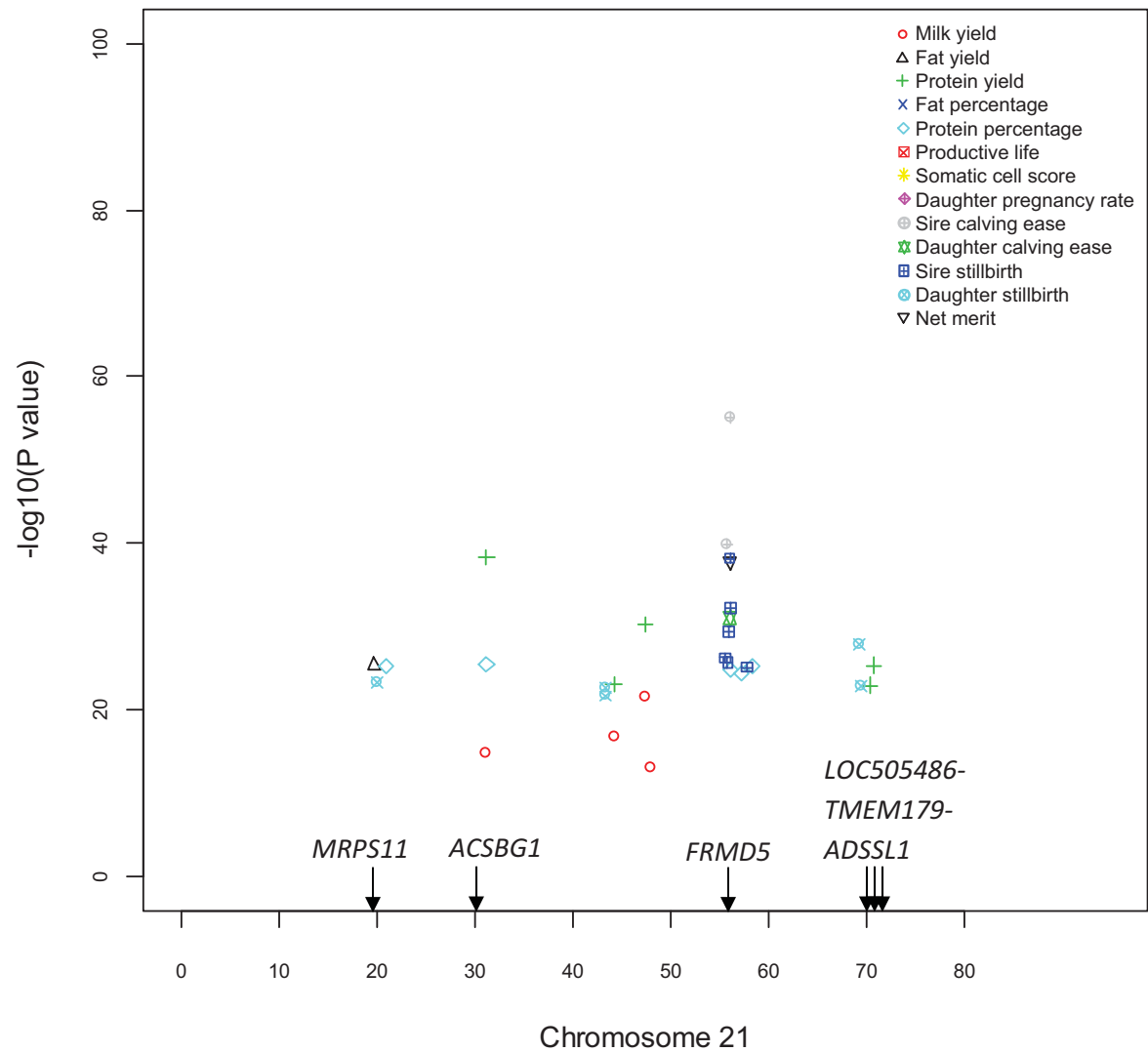

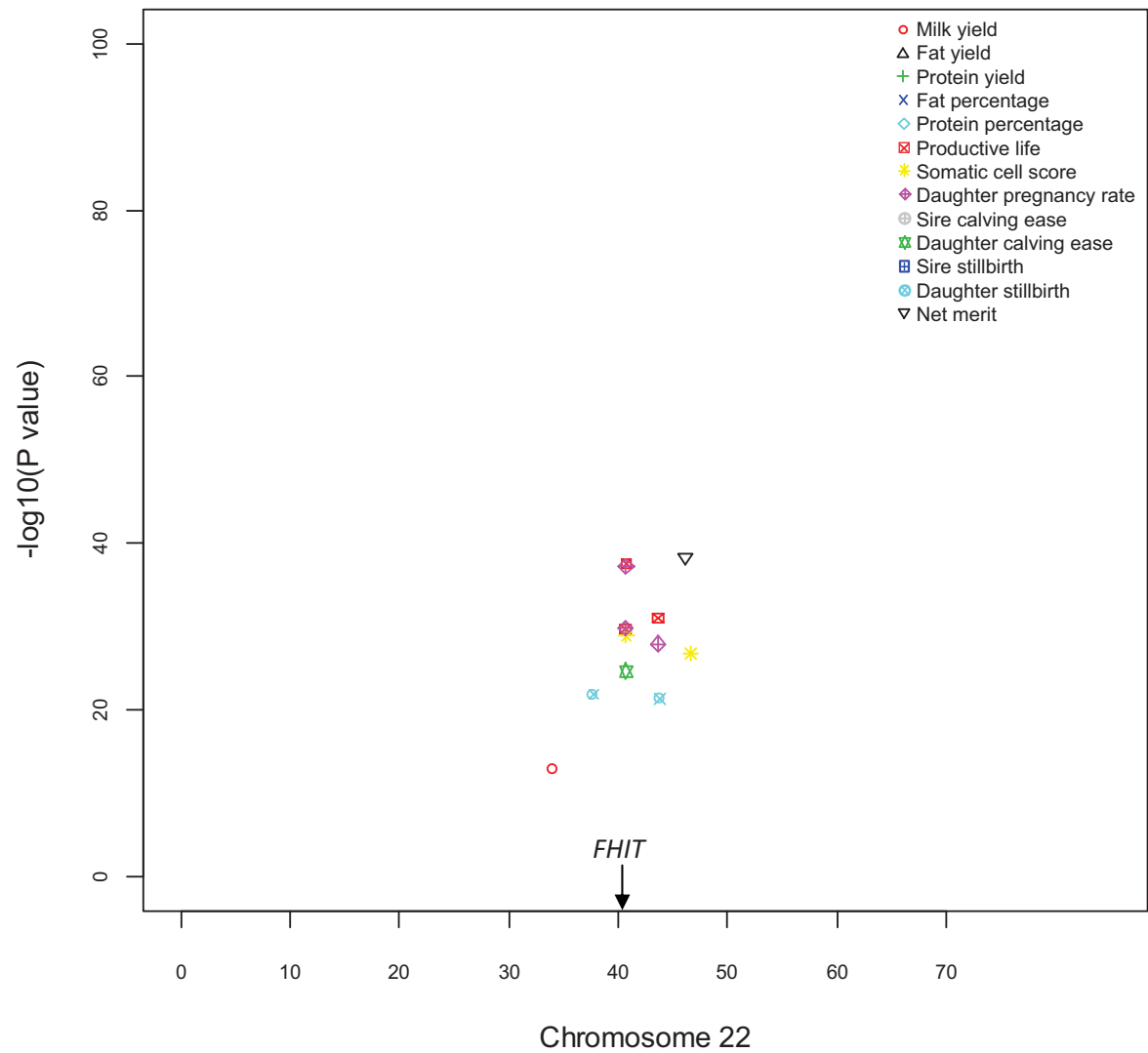

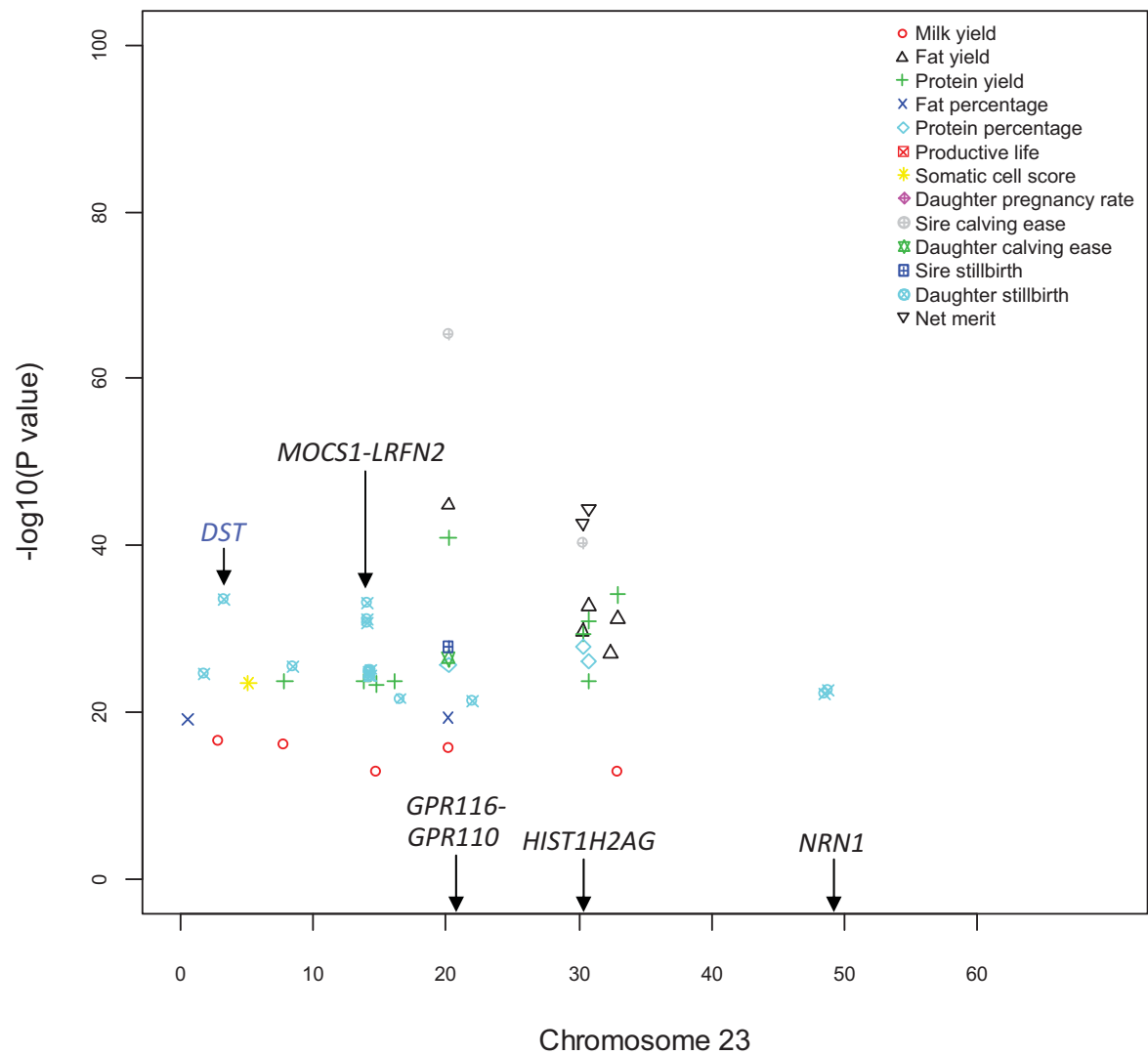

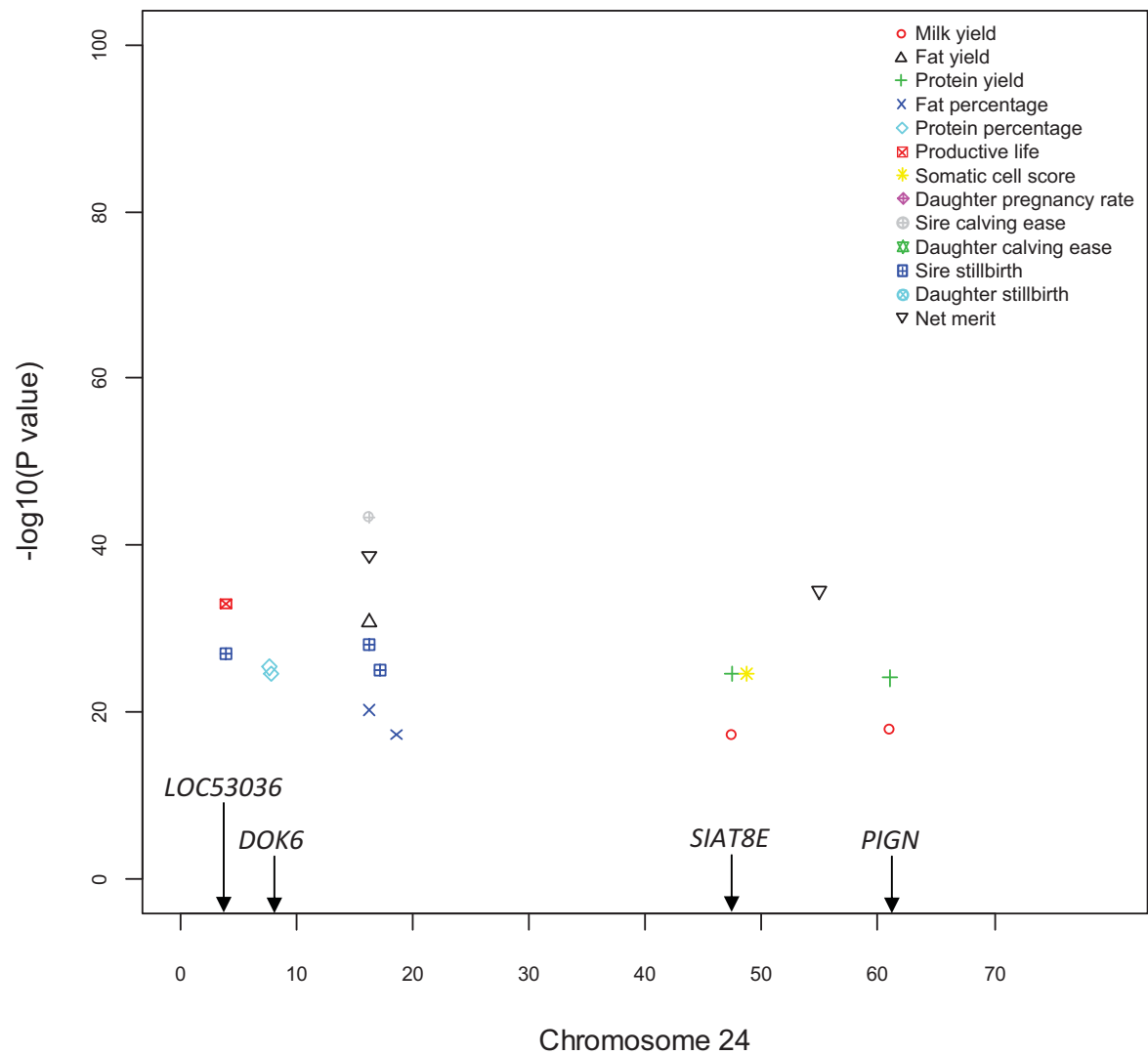

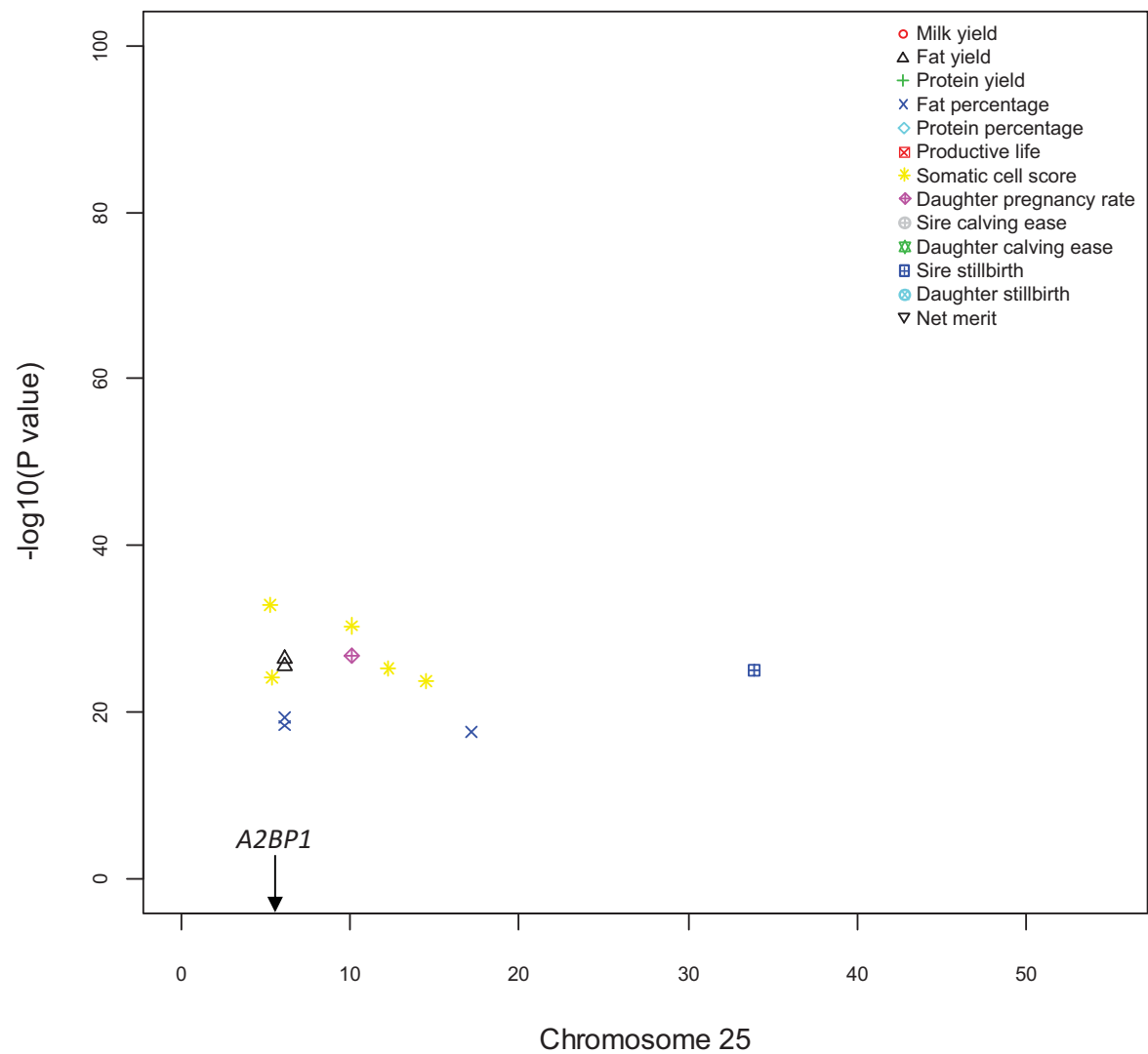

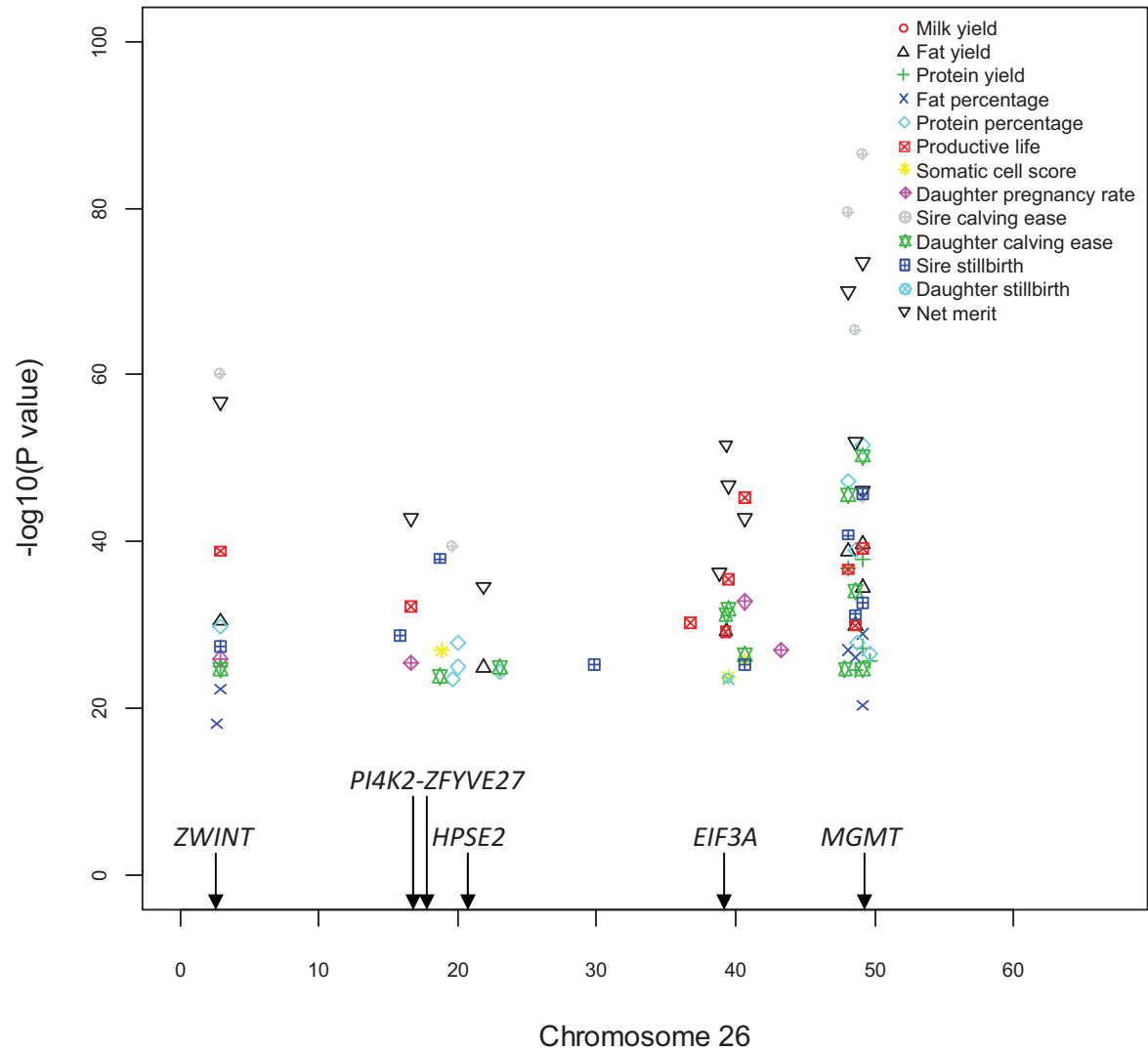

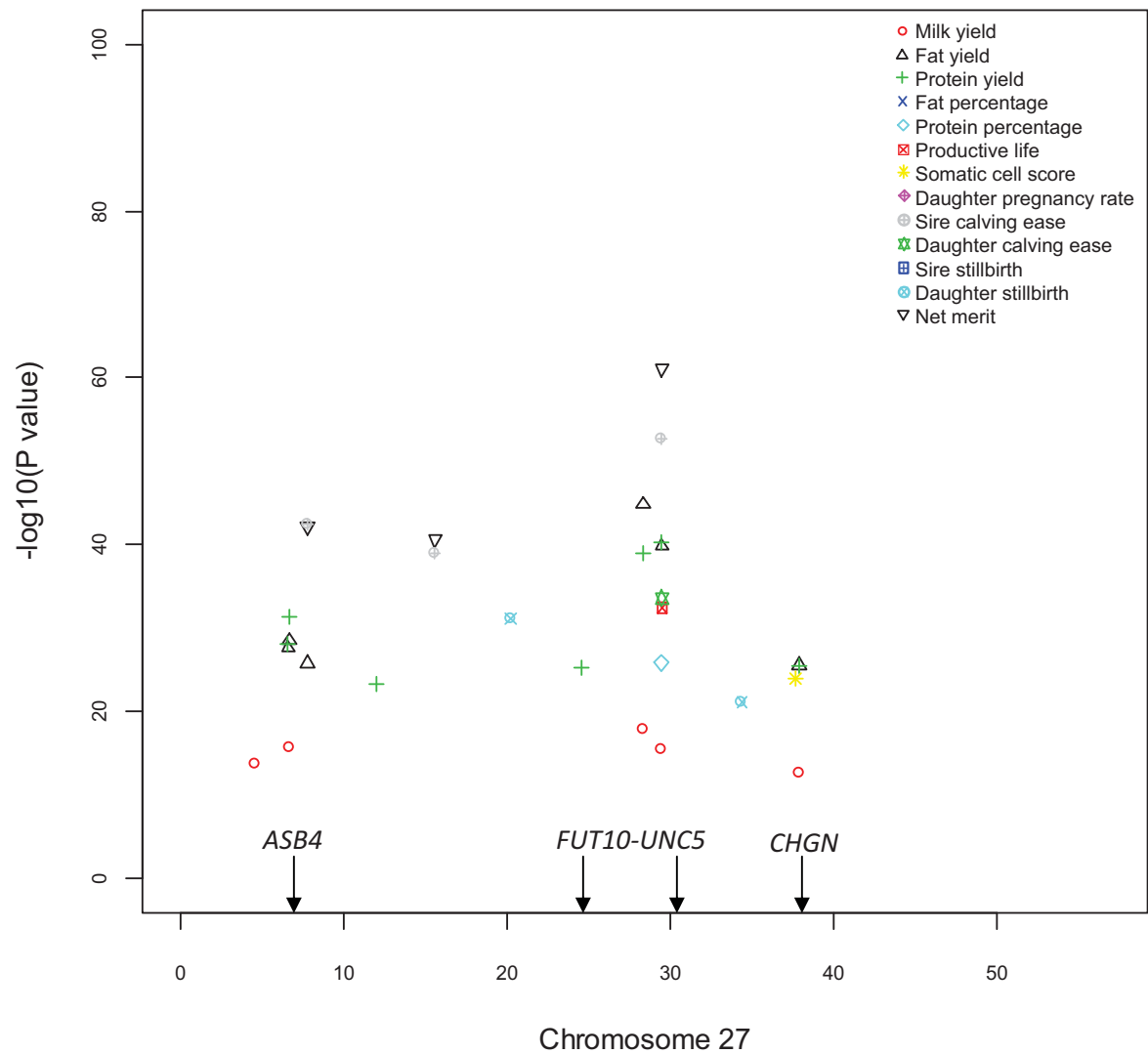

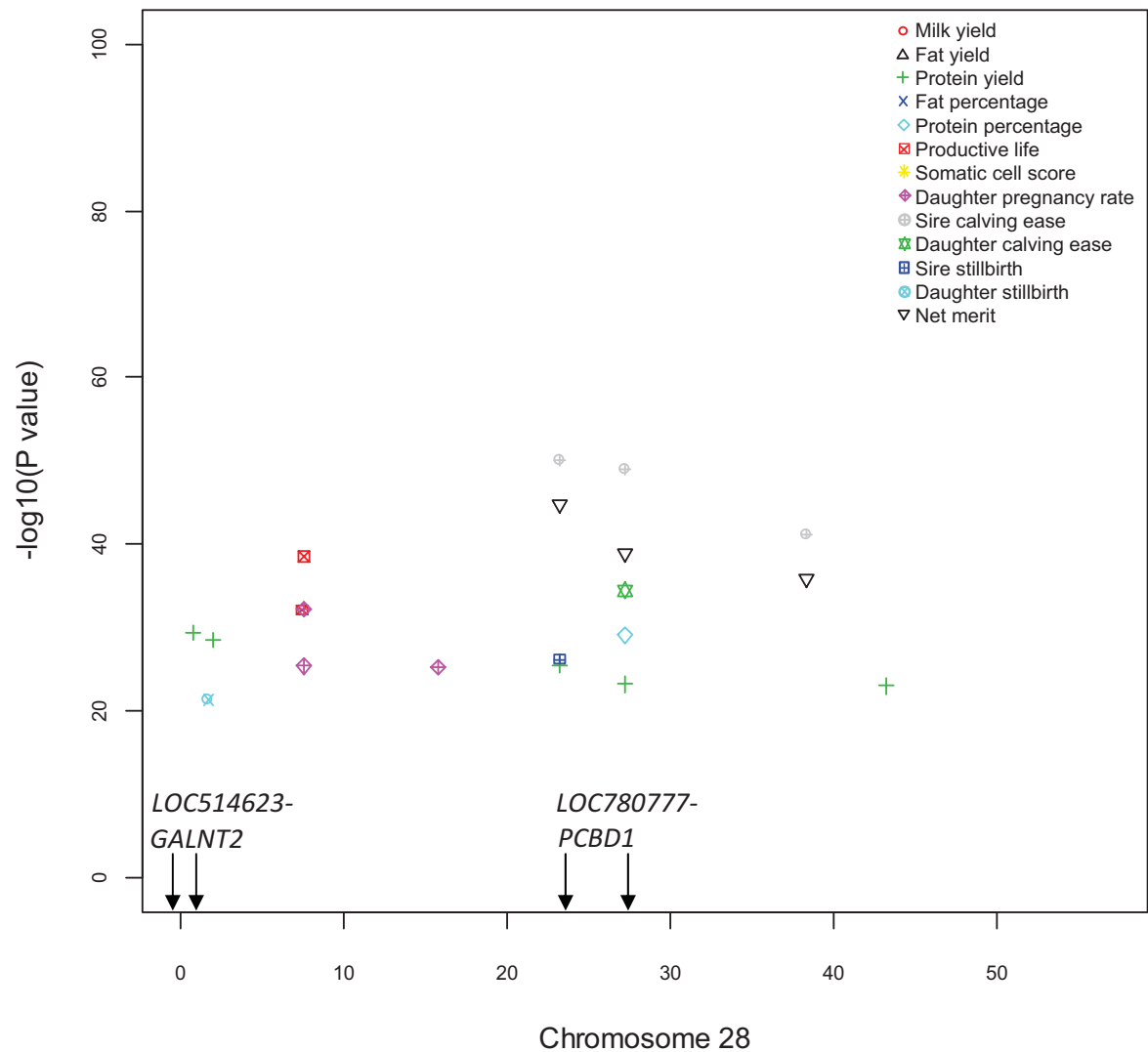

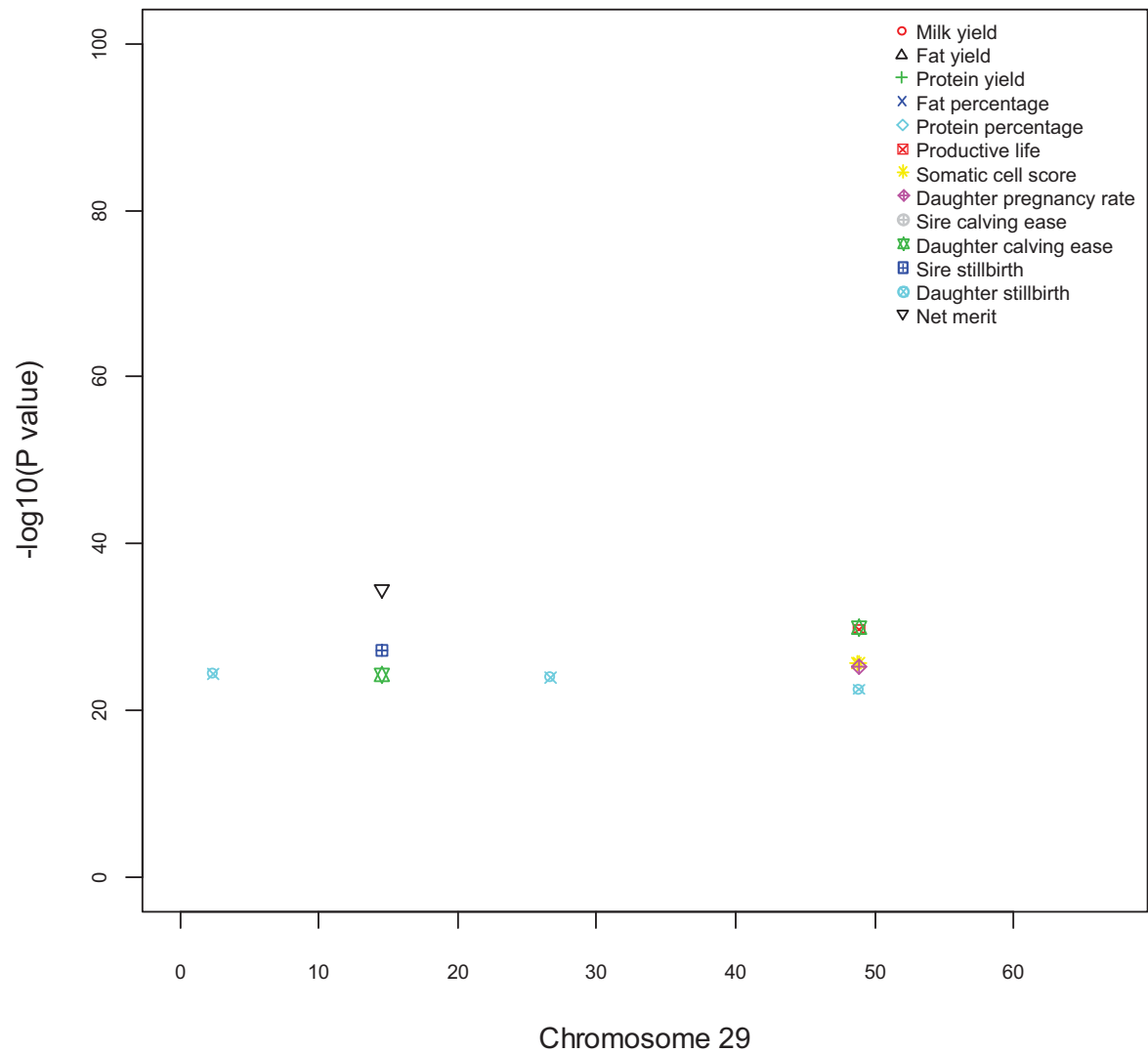

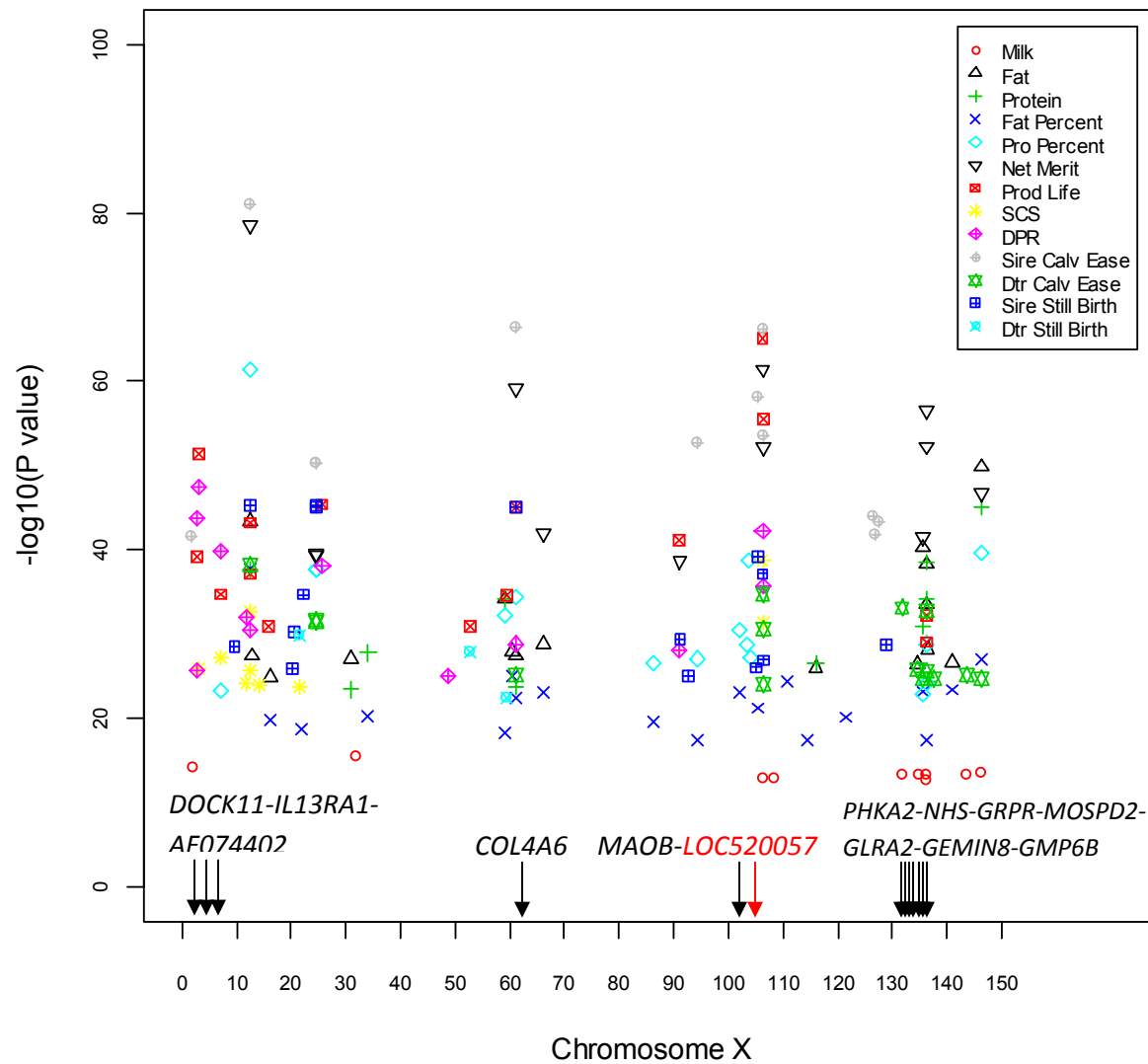

Supplement: Additional file 3 — Figure S2. Map of SNP position (Mb) by P-value and Bos taurus chromosome for 725 SNPs that comprise the top 100 effects for each of 13 production, health, and reproduction traits of contemporary U.S. Holsteins. [file 1471-2164-12-408-S3.PDF]

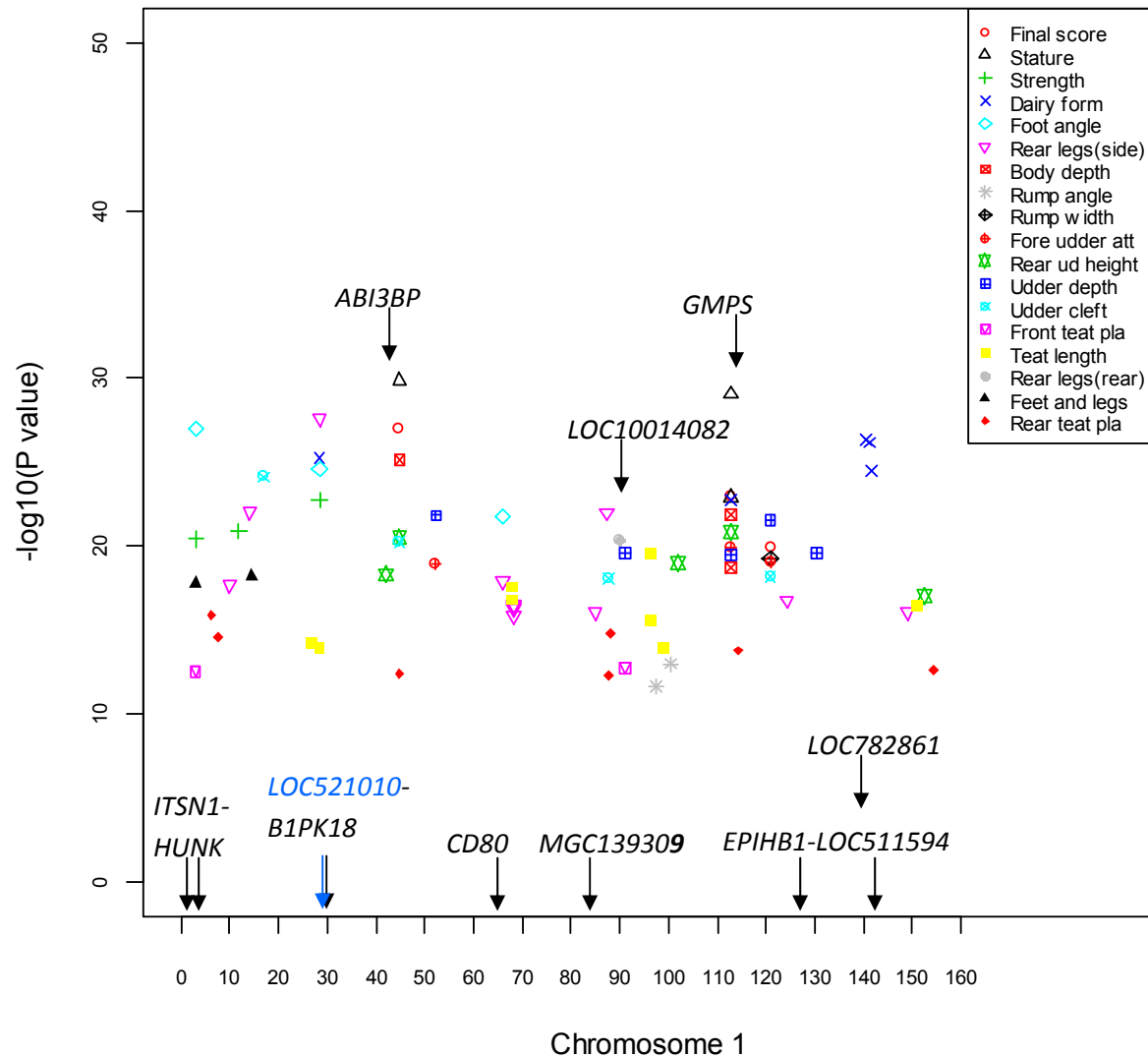

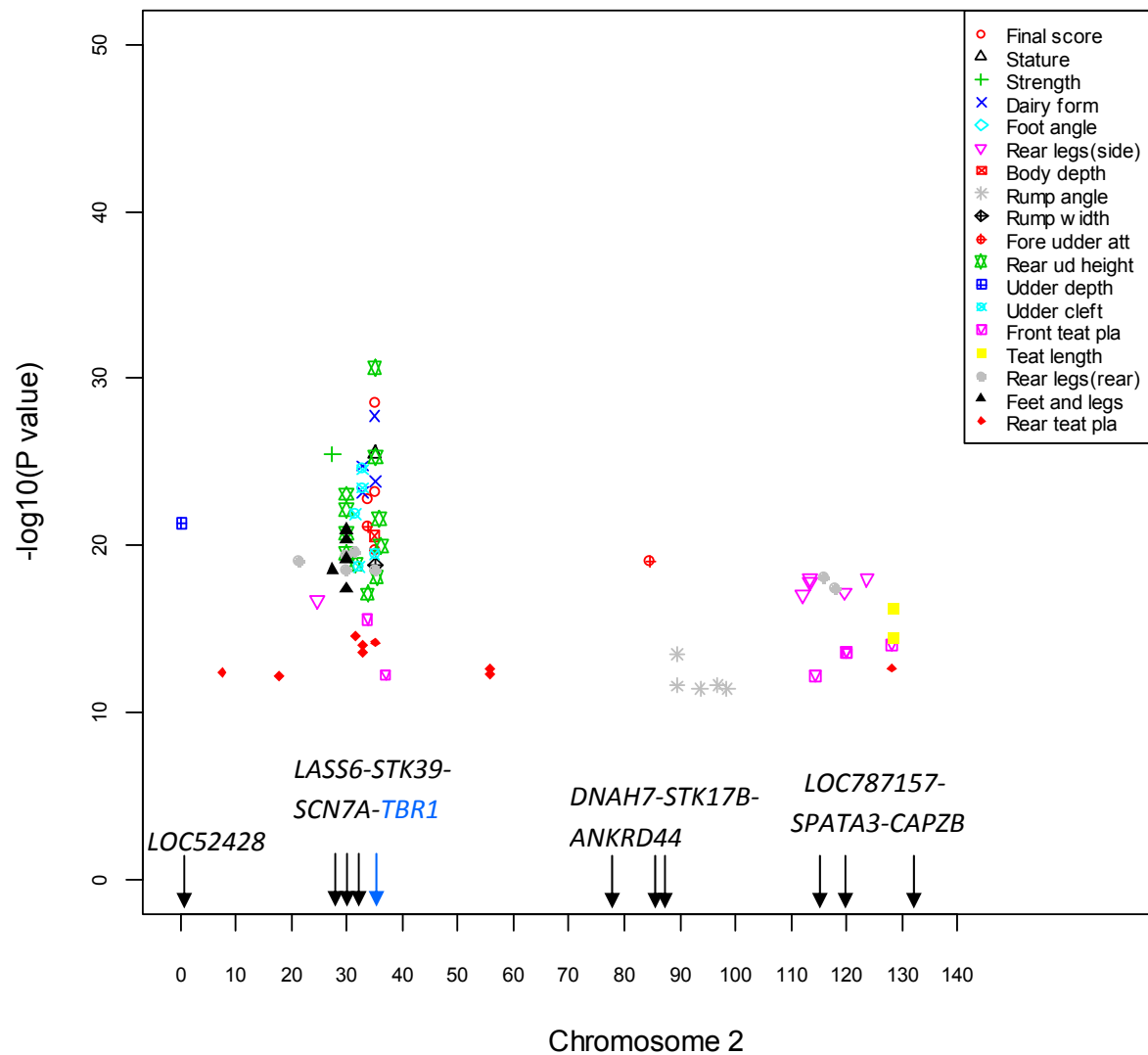

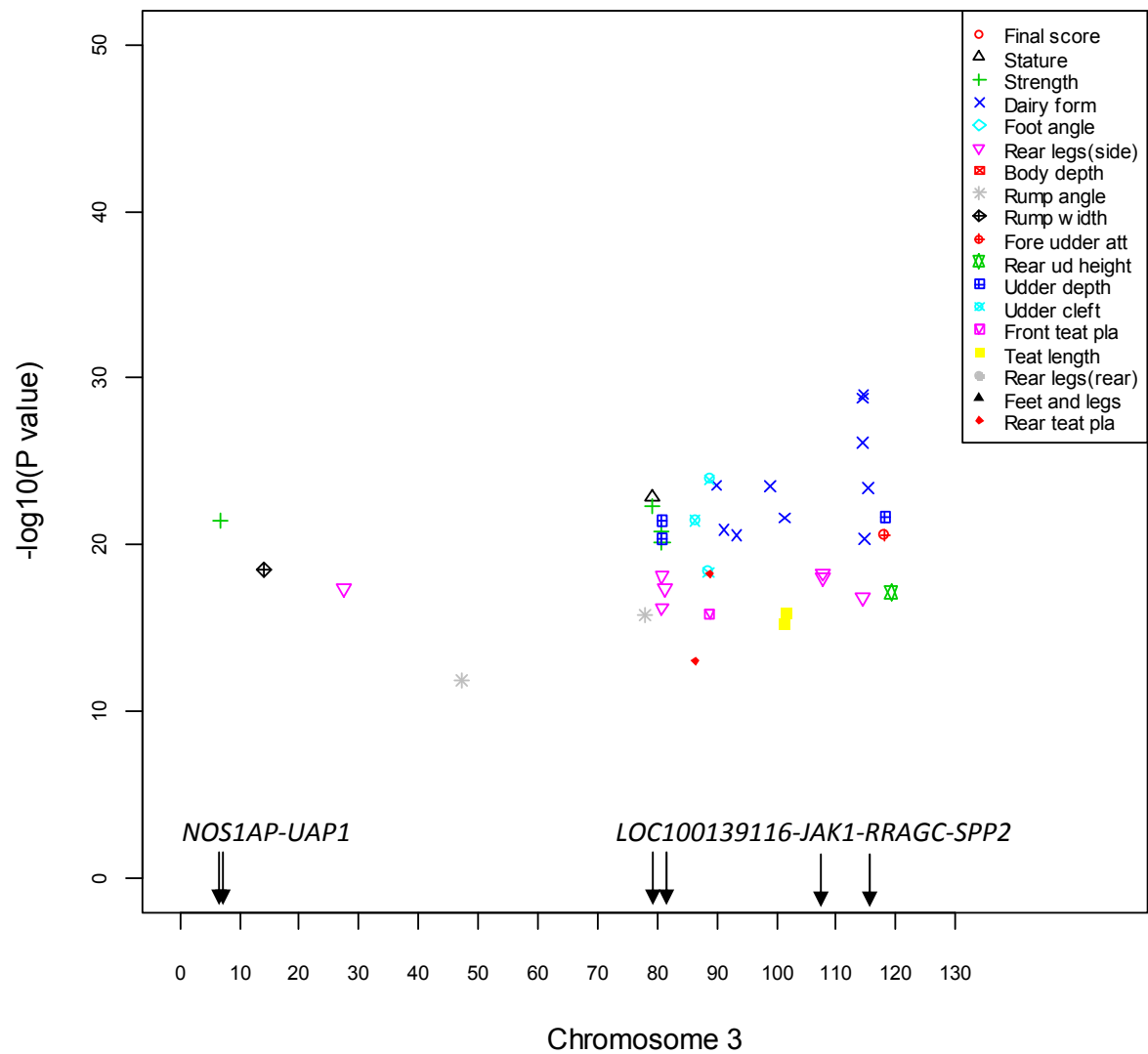

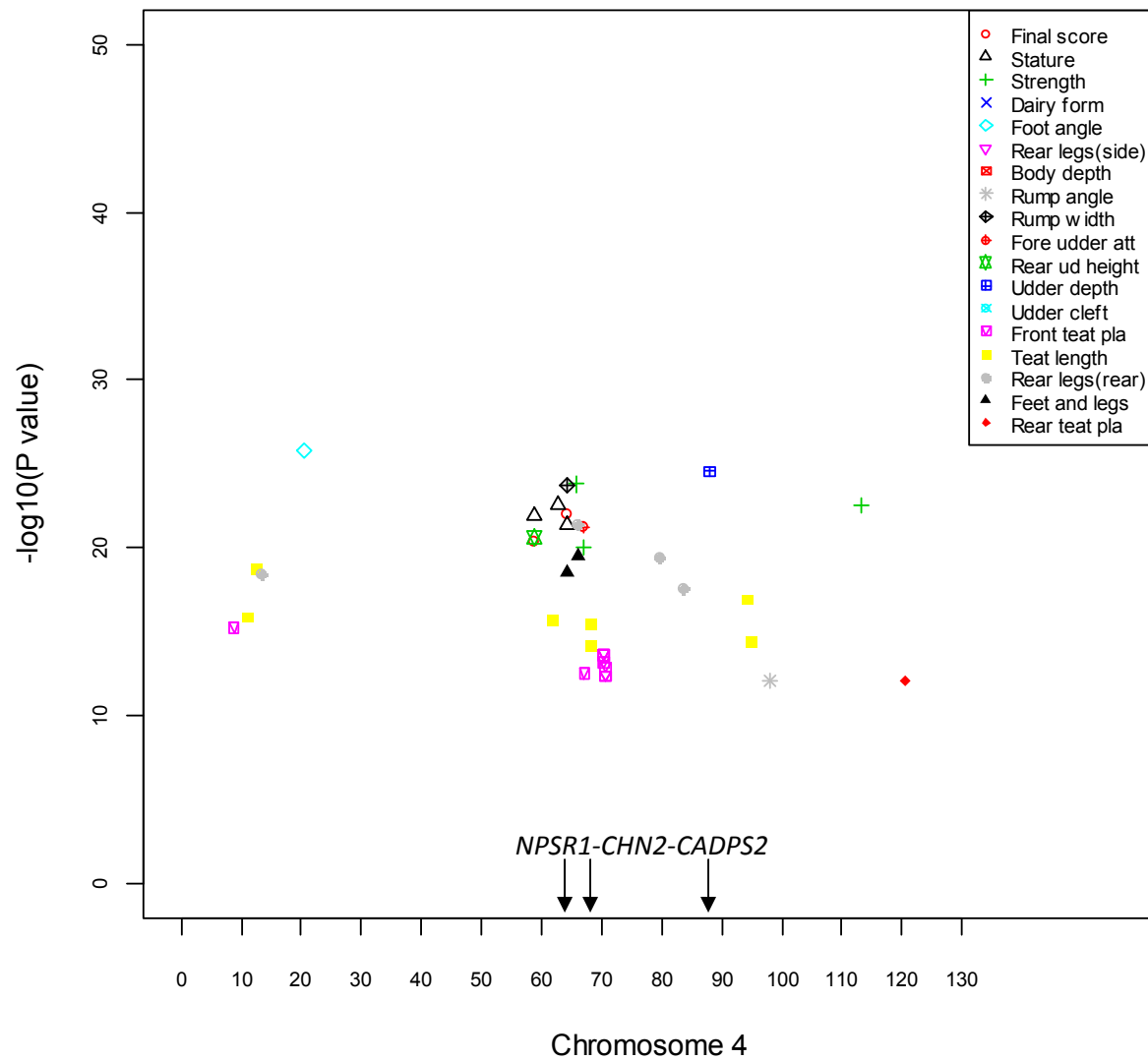

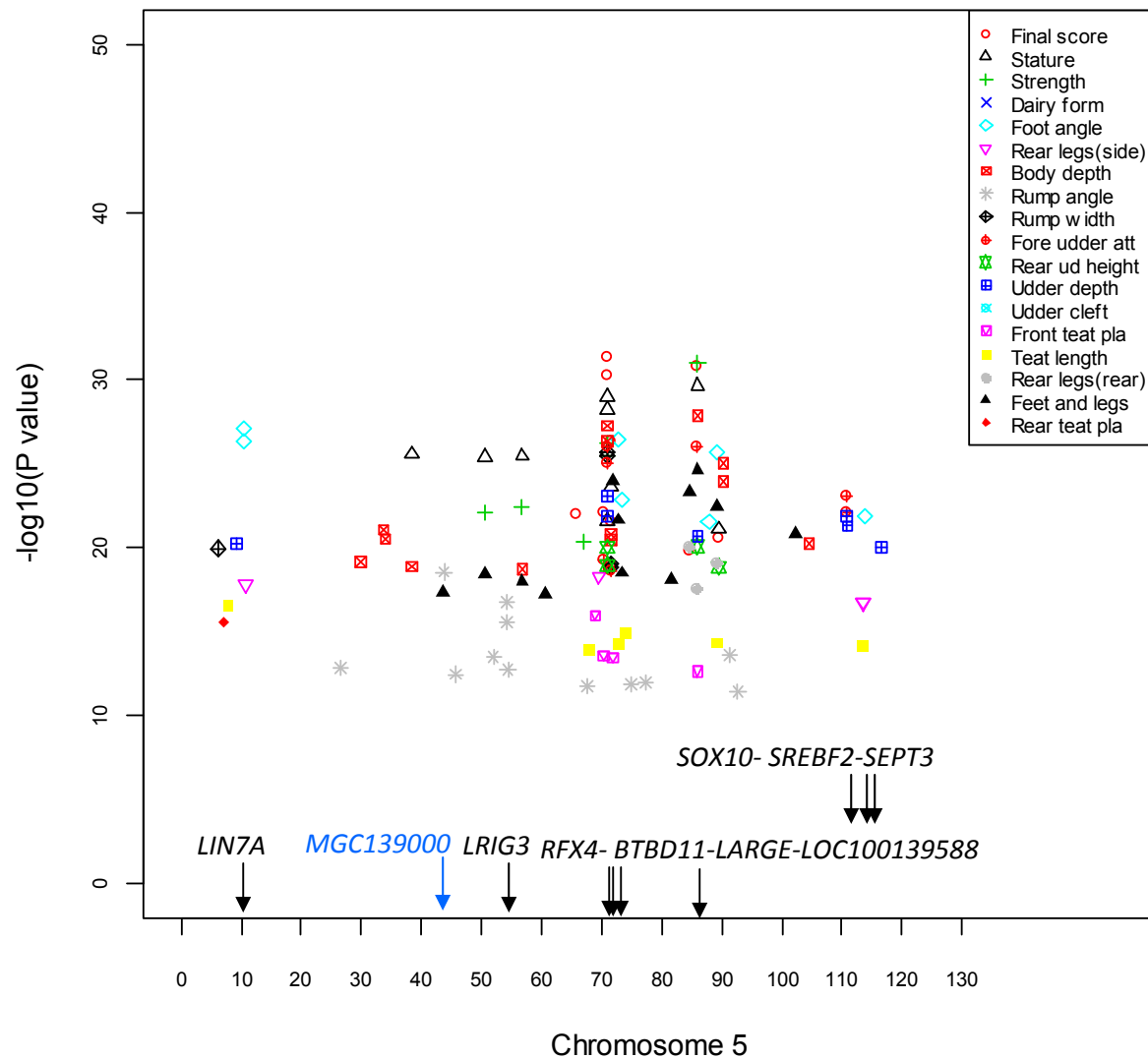

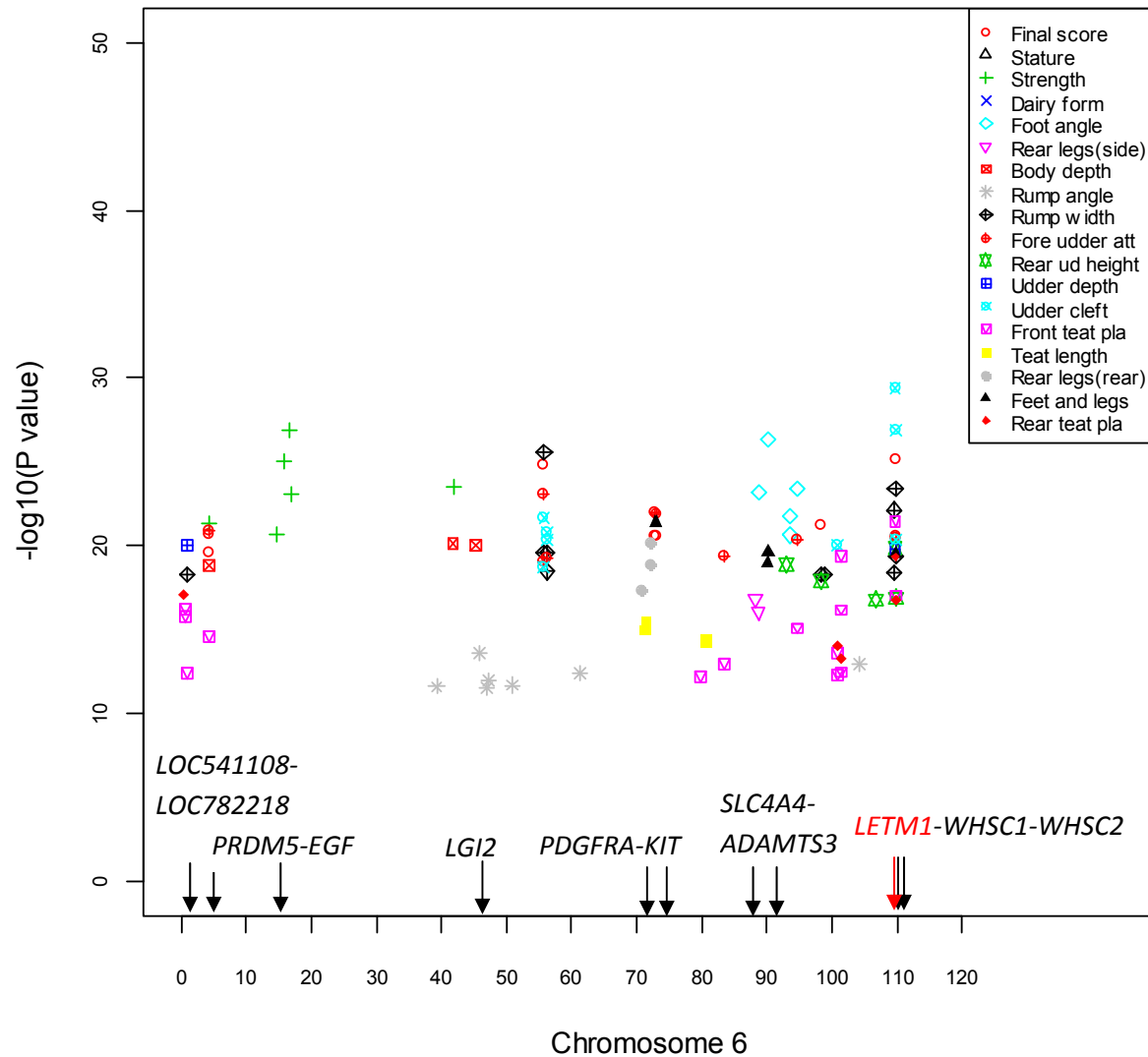

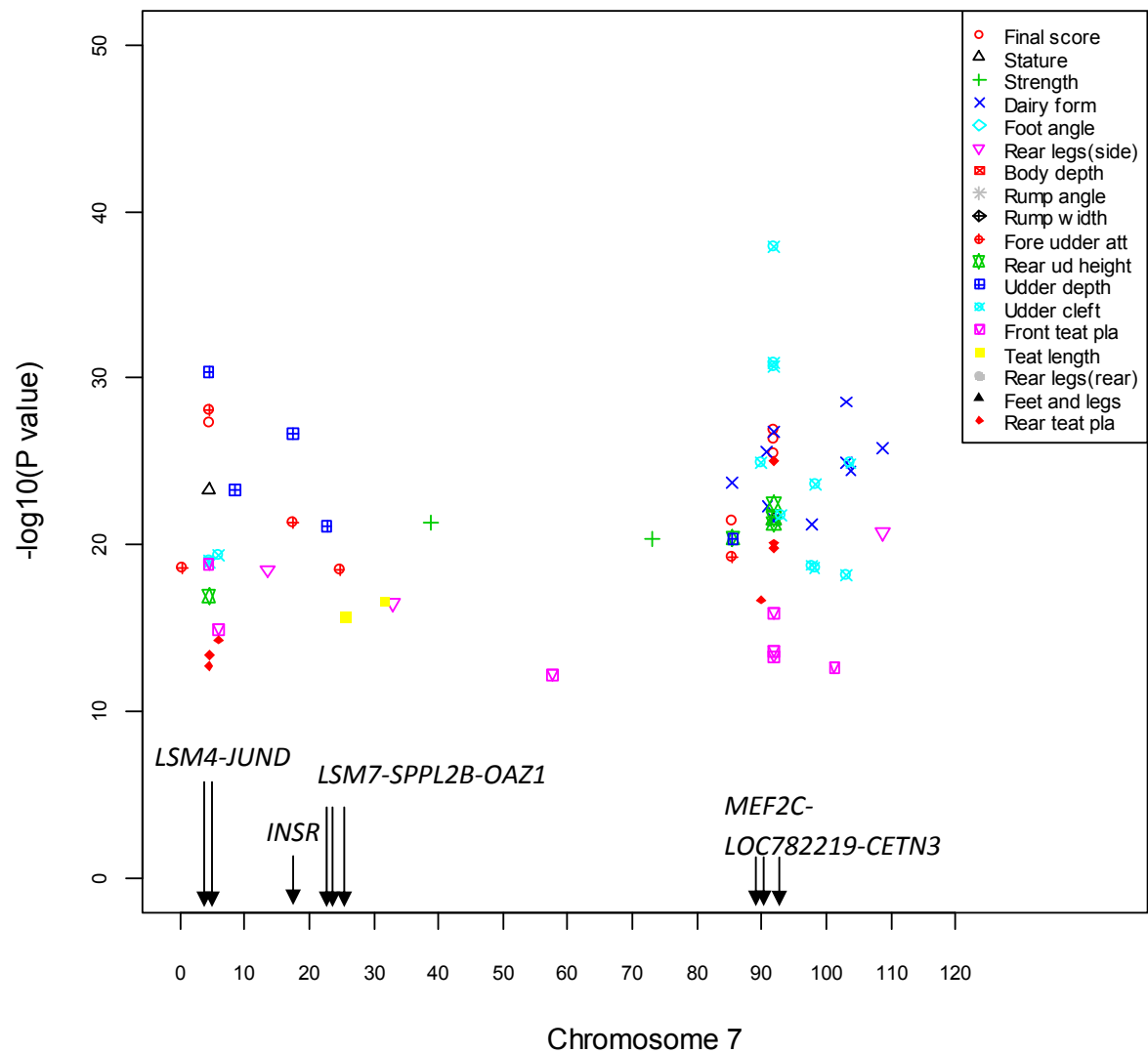

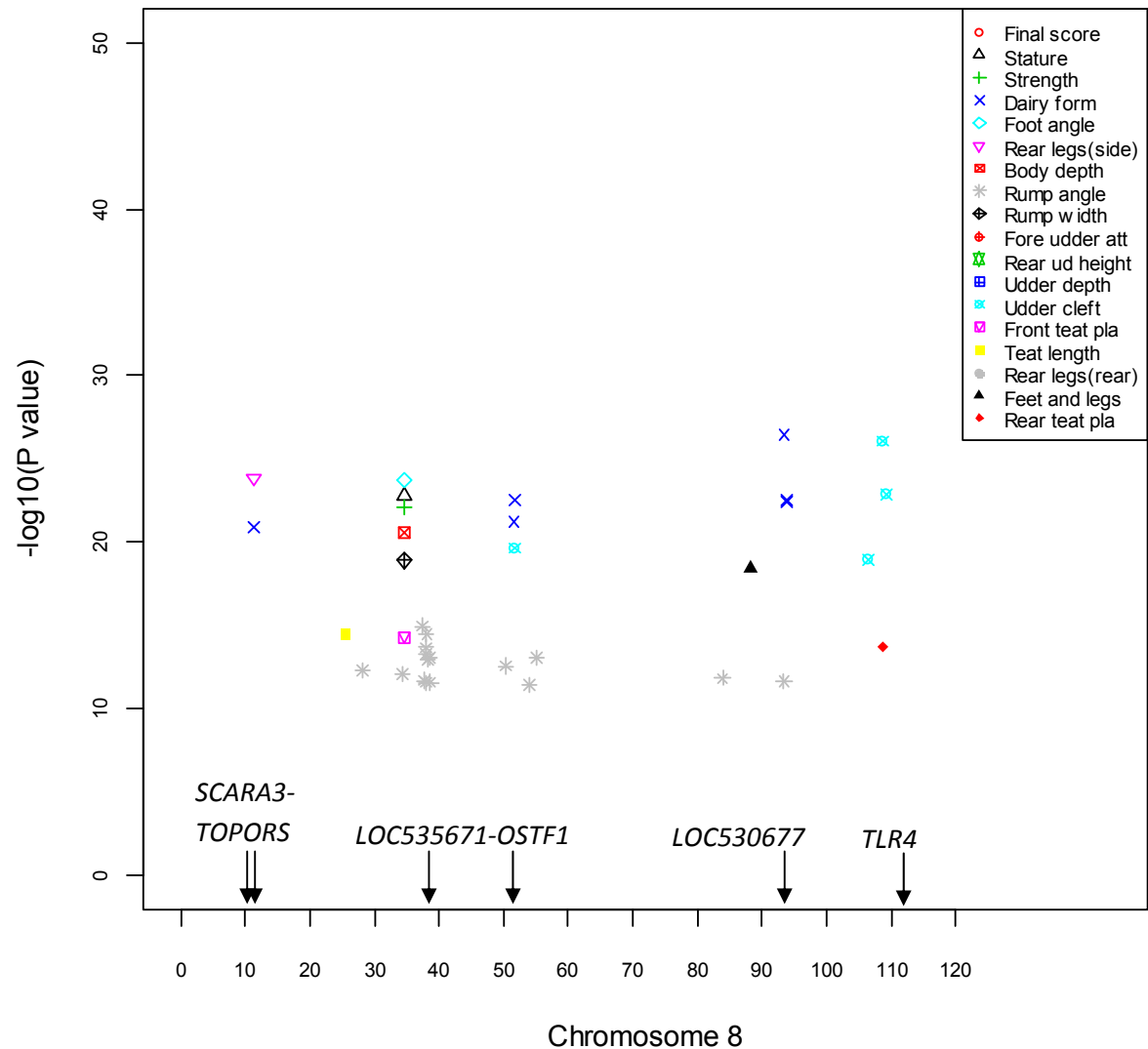

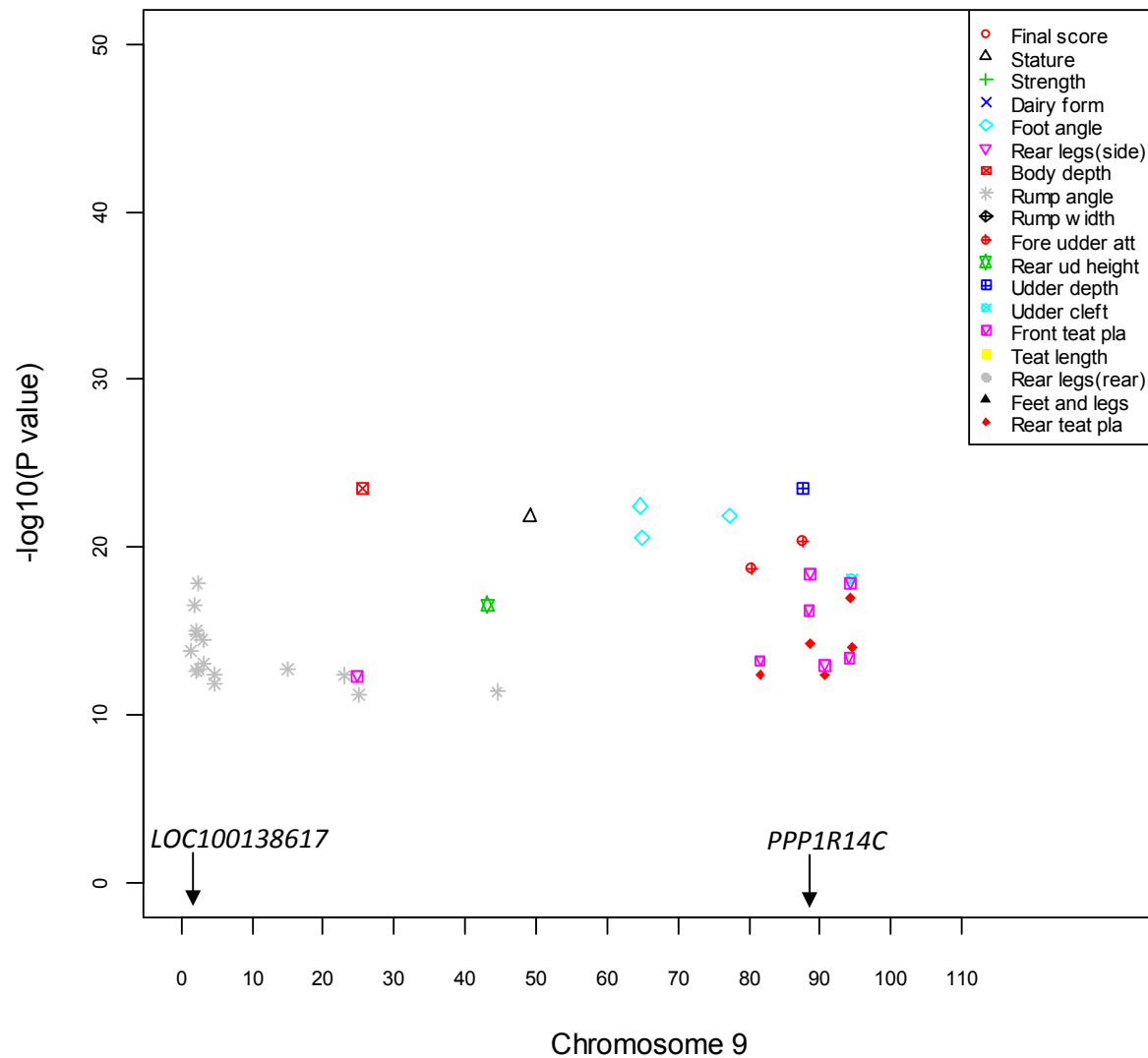

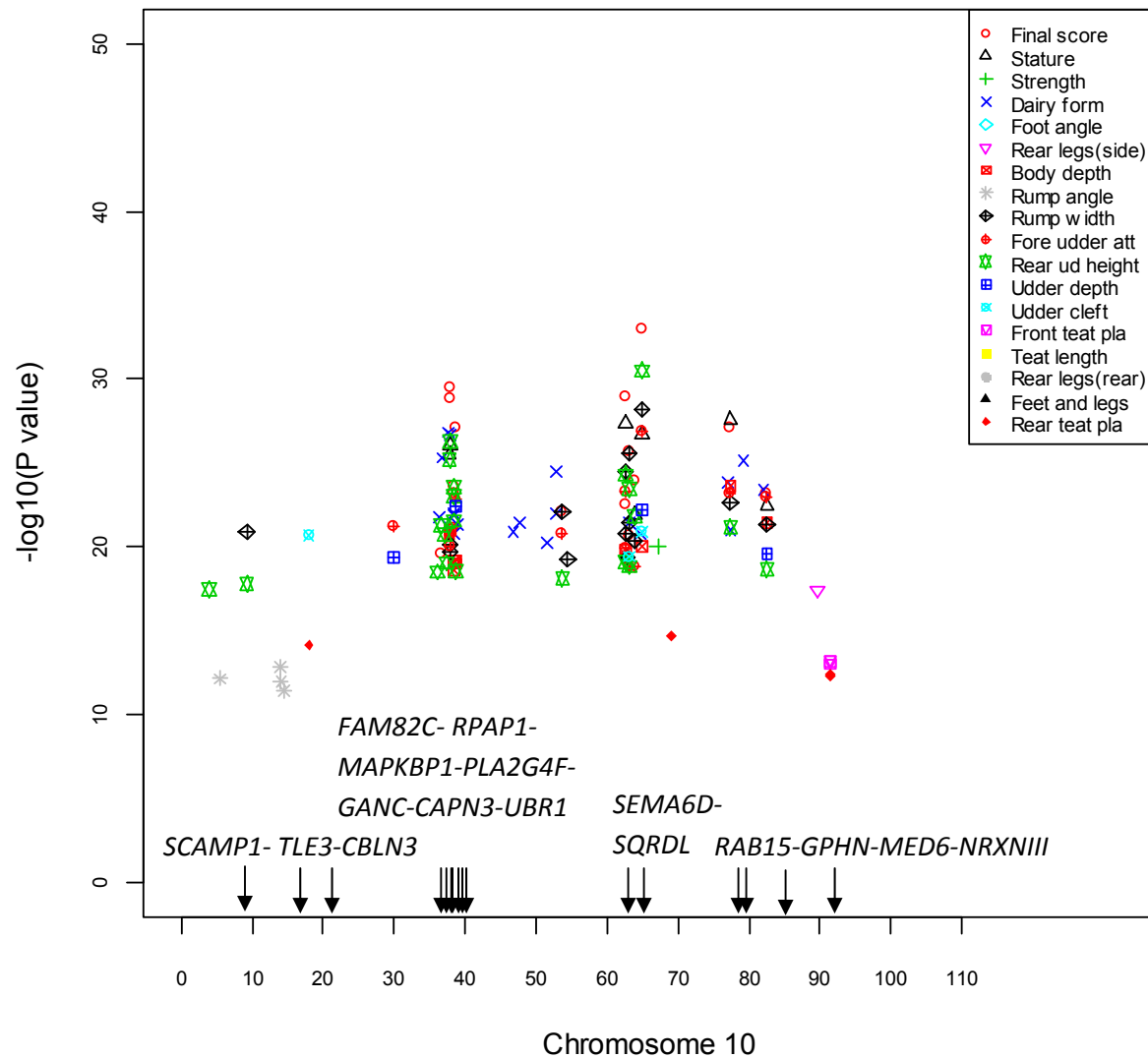

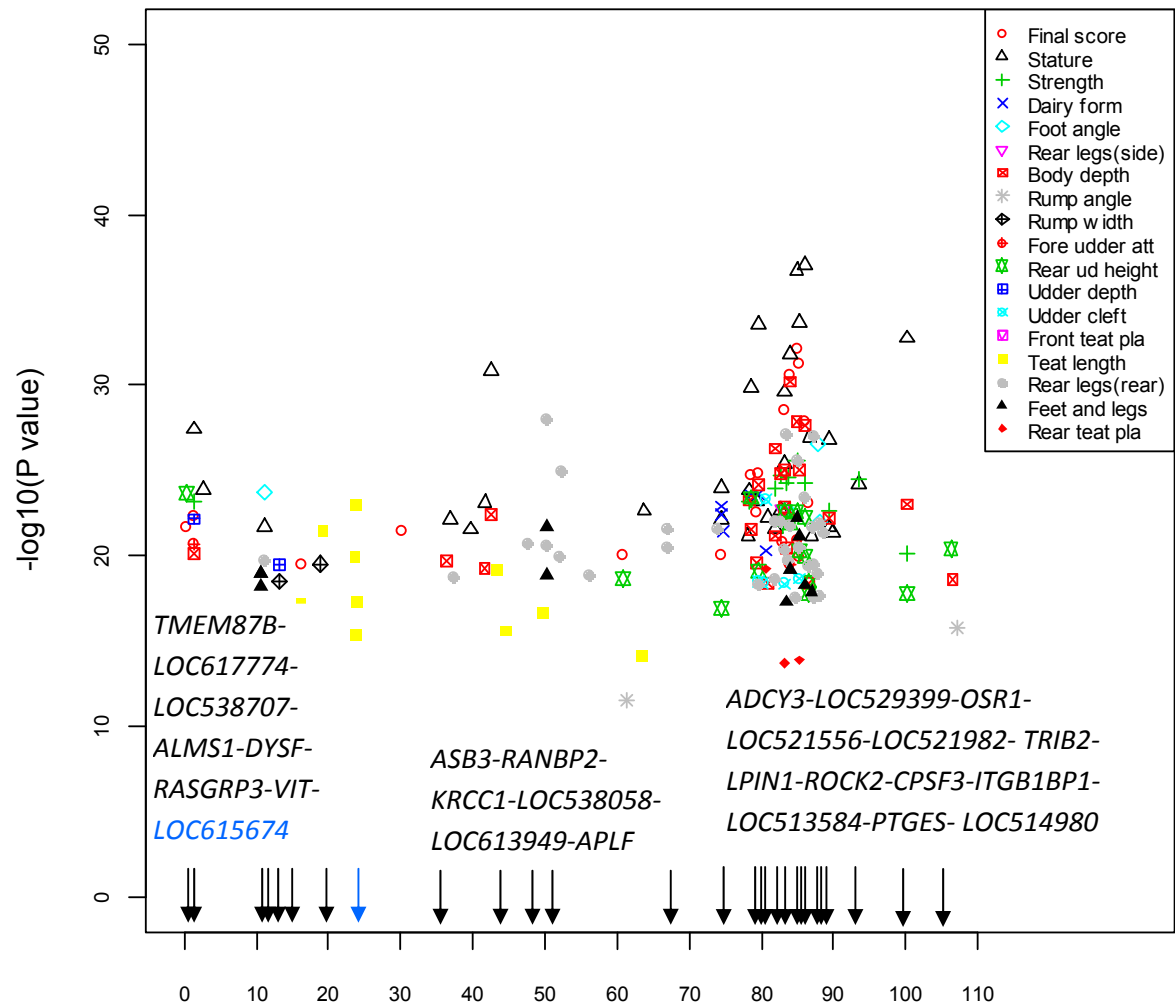

Chromosome 11

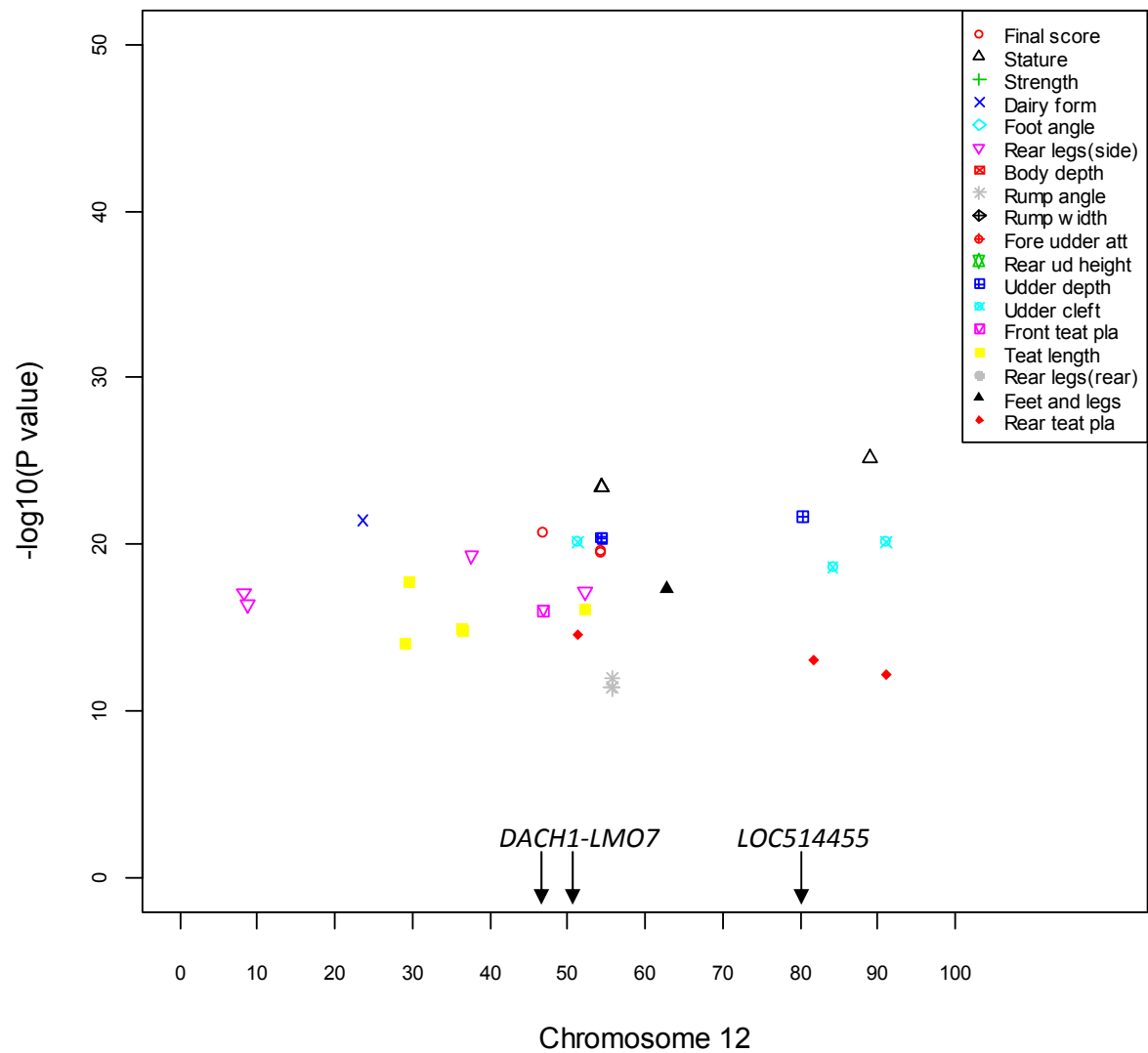

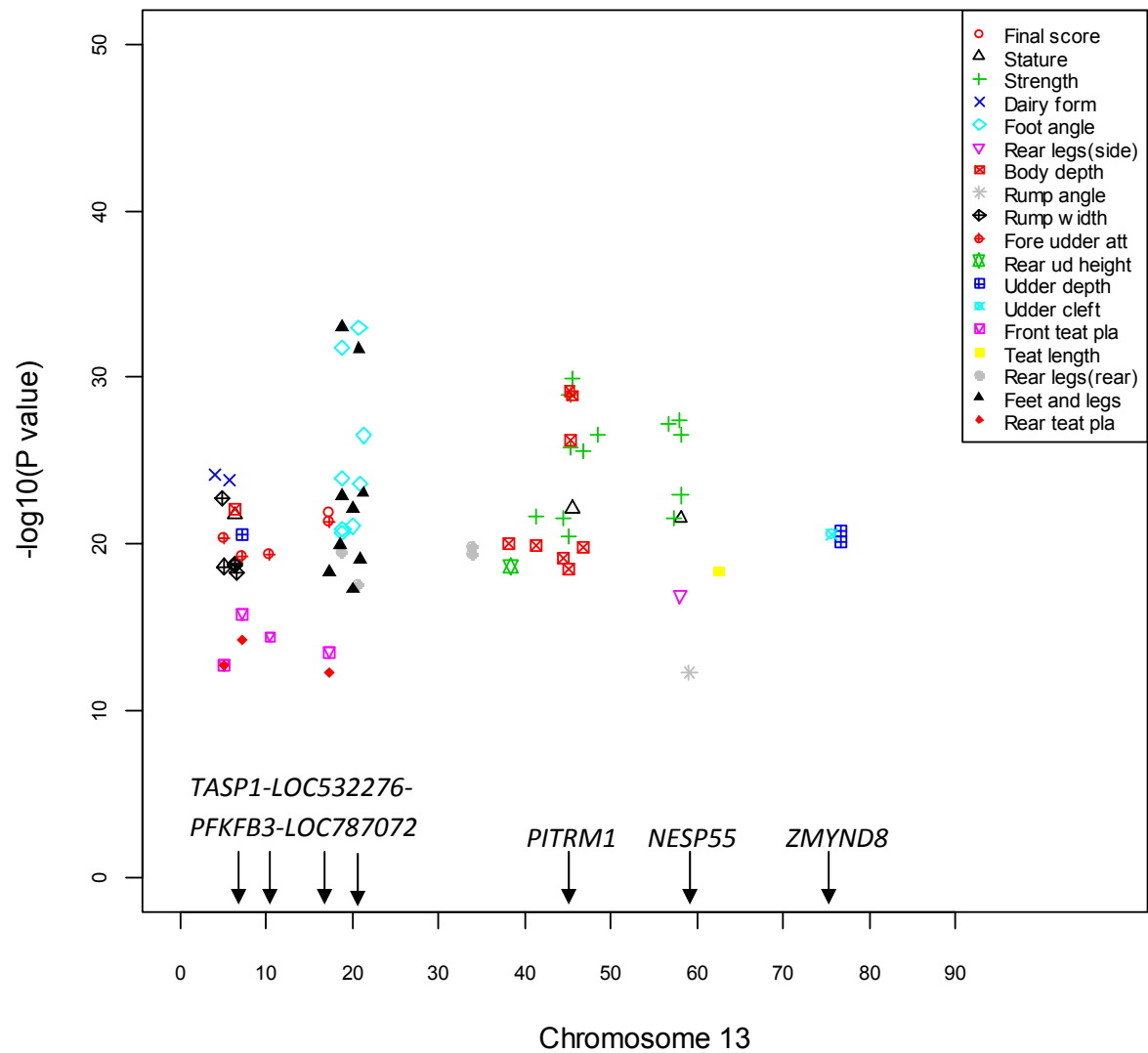

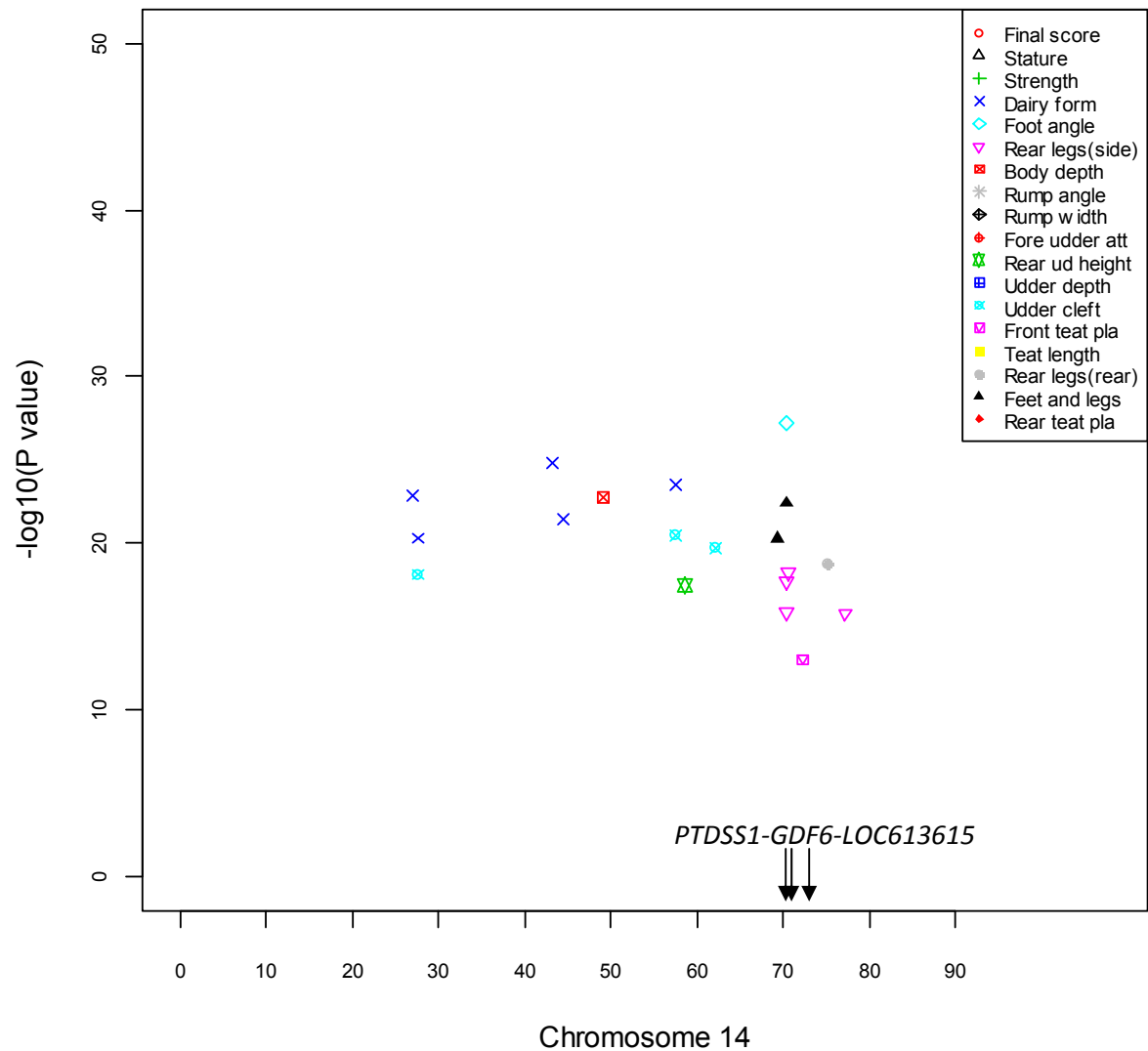

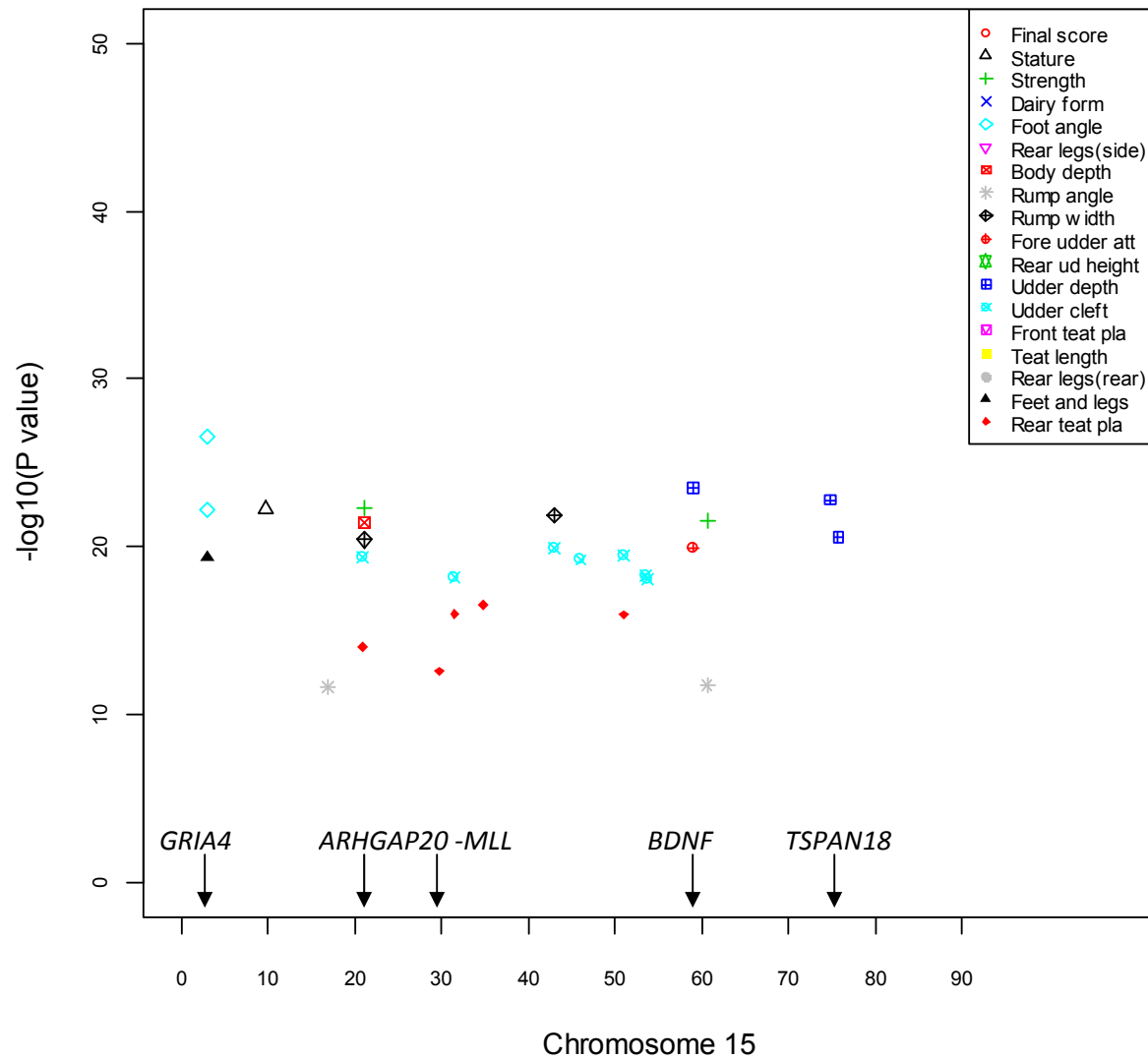

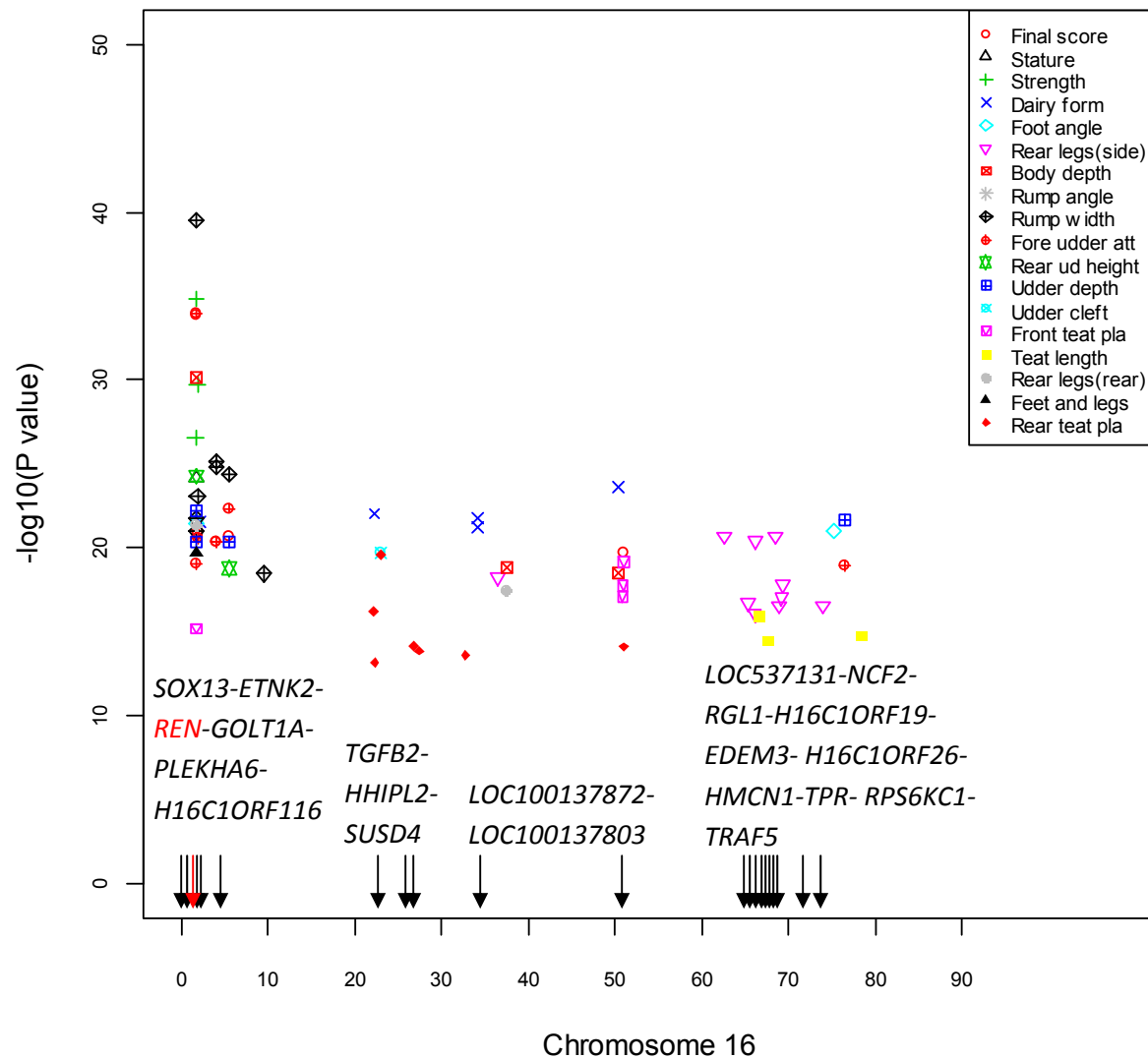

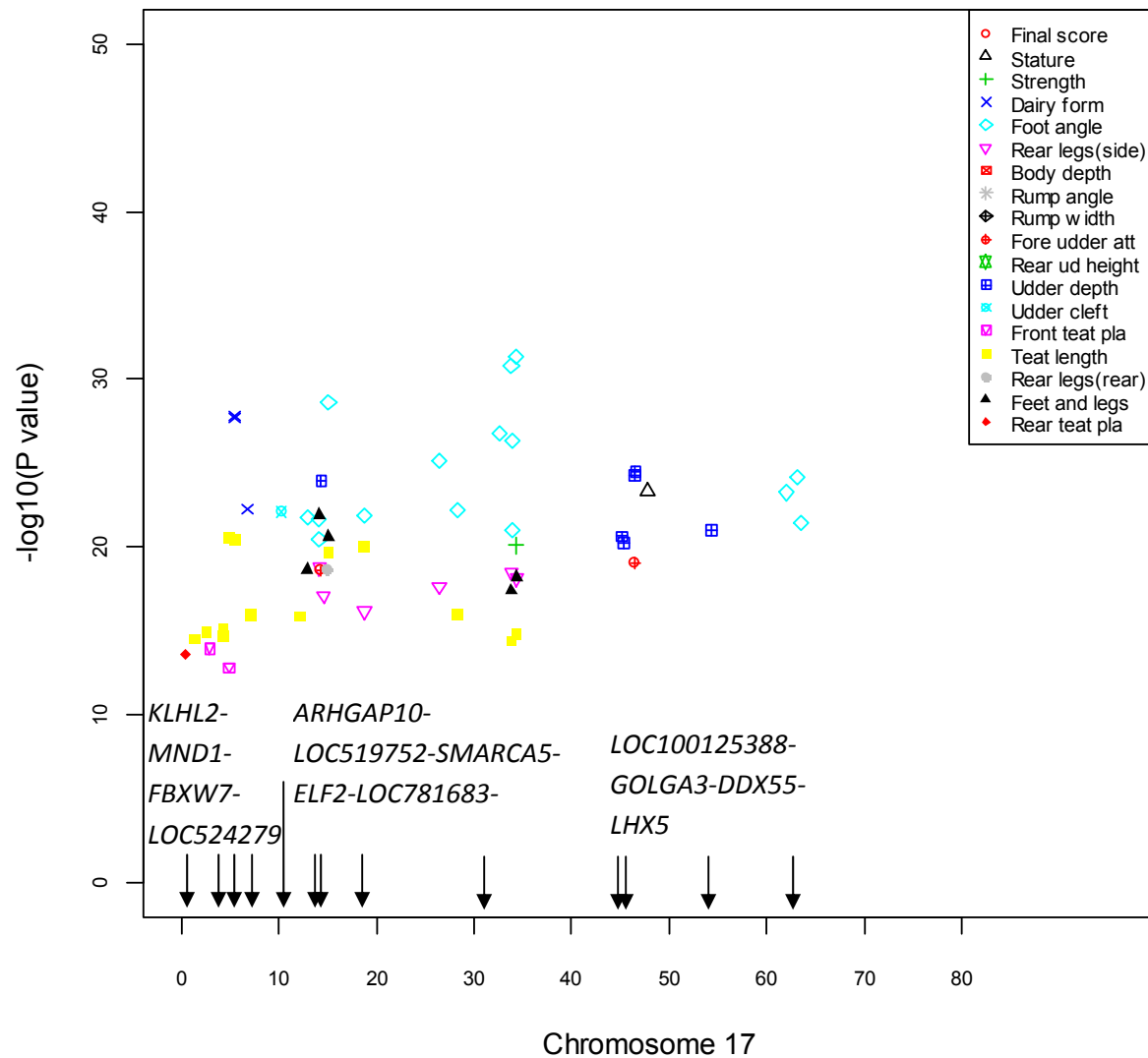

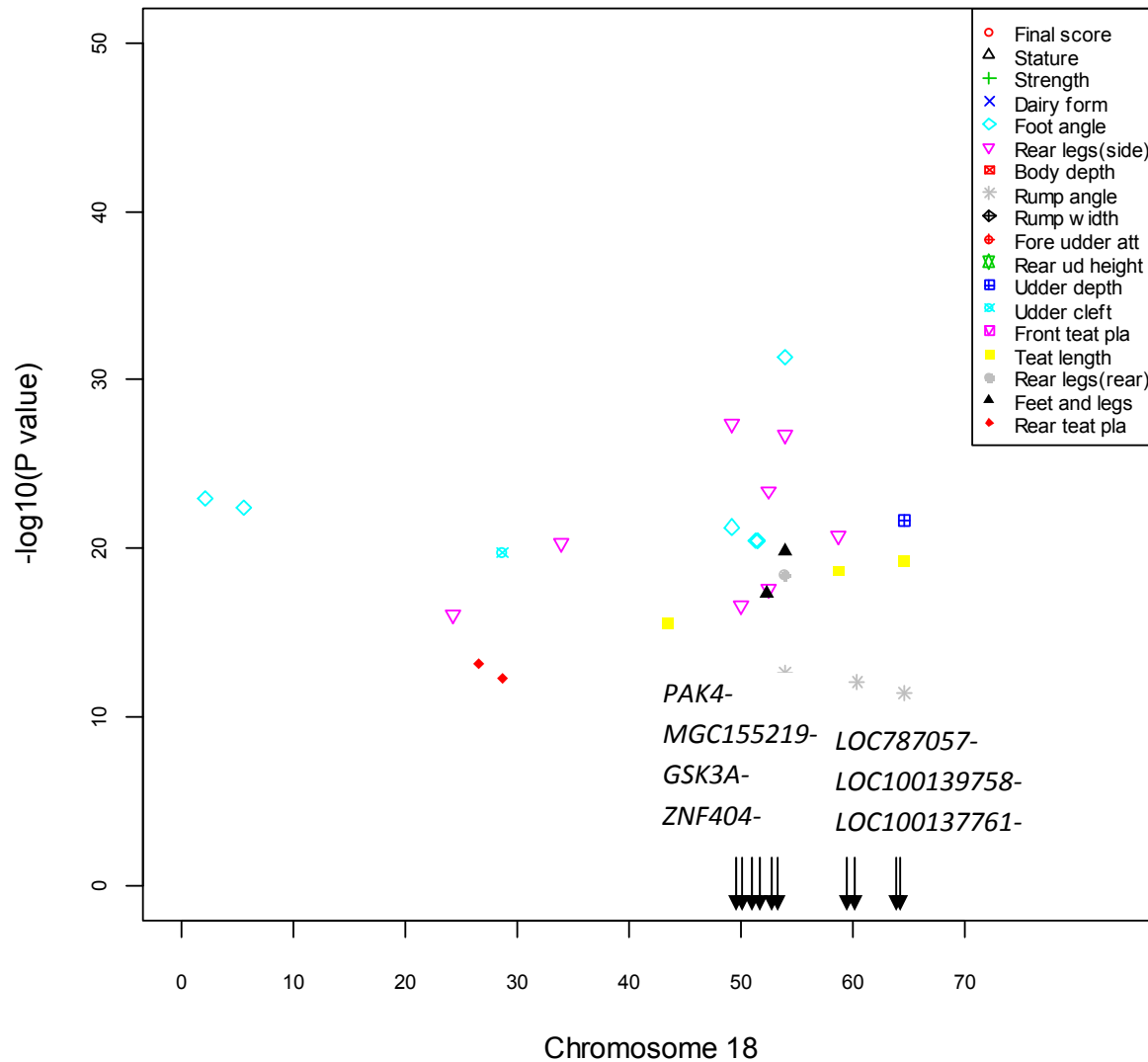

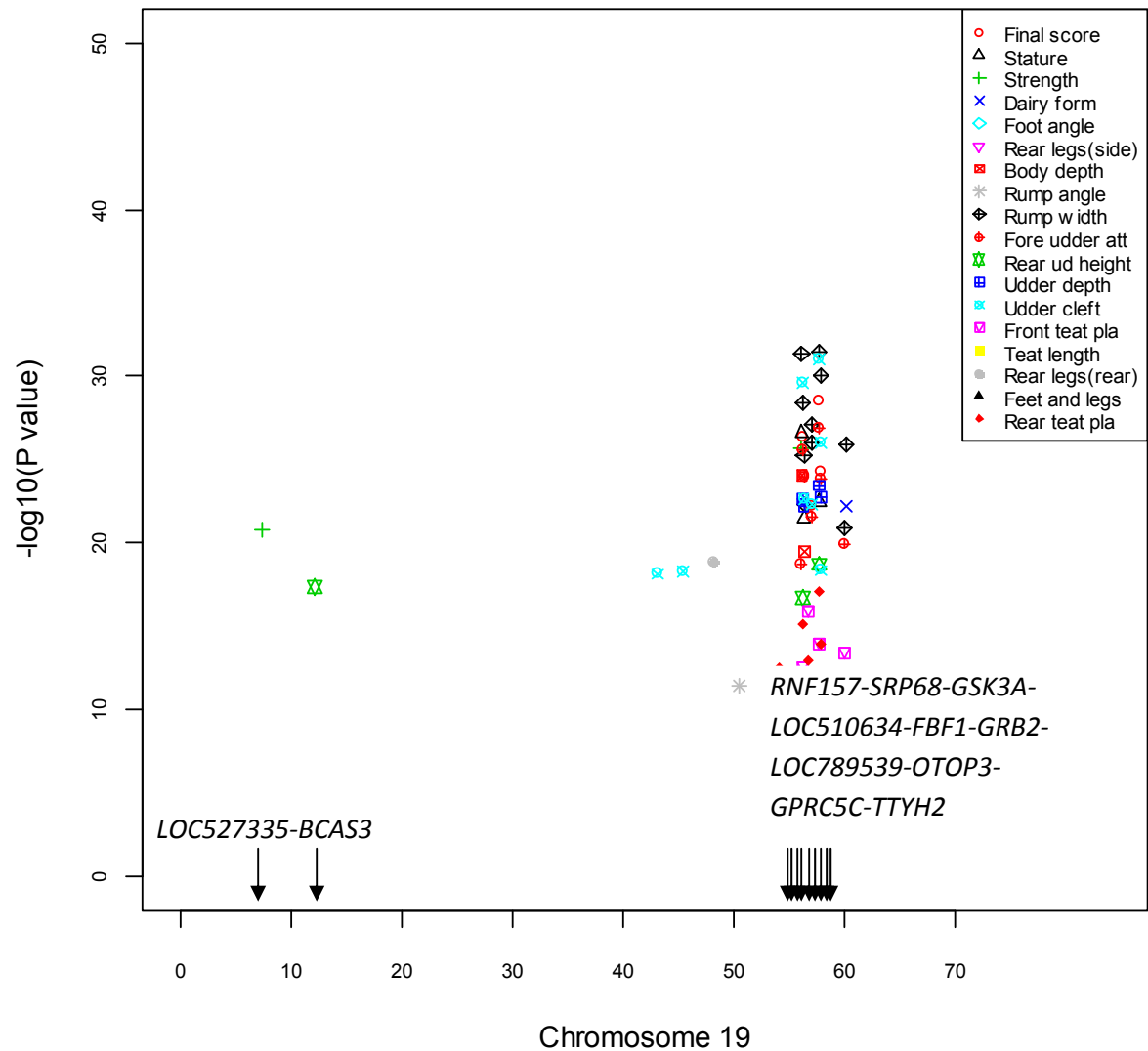

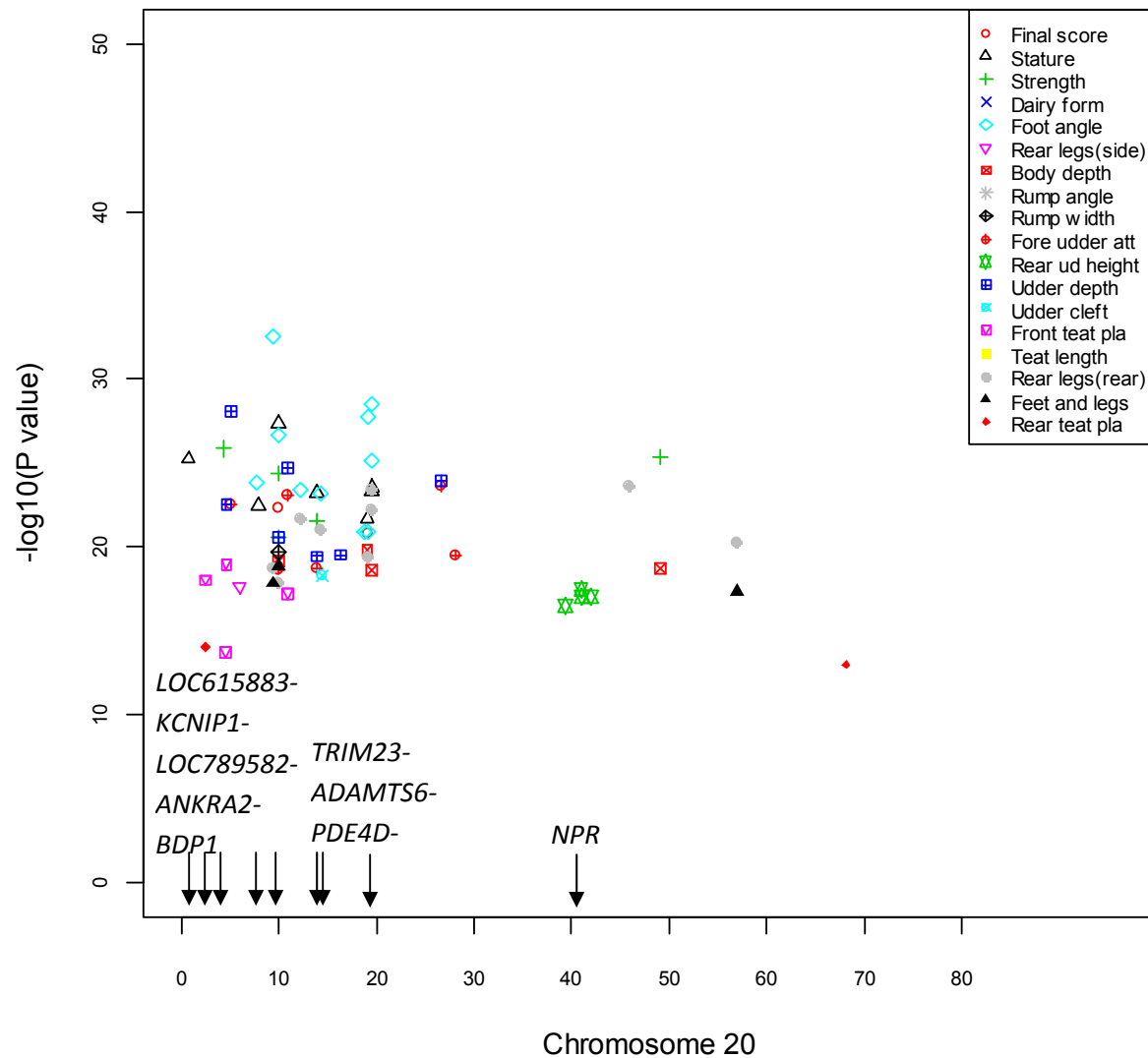

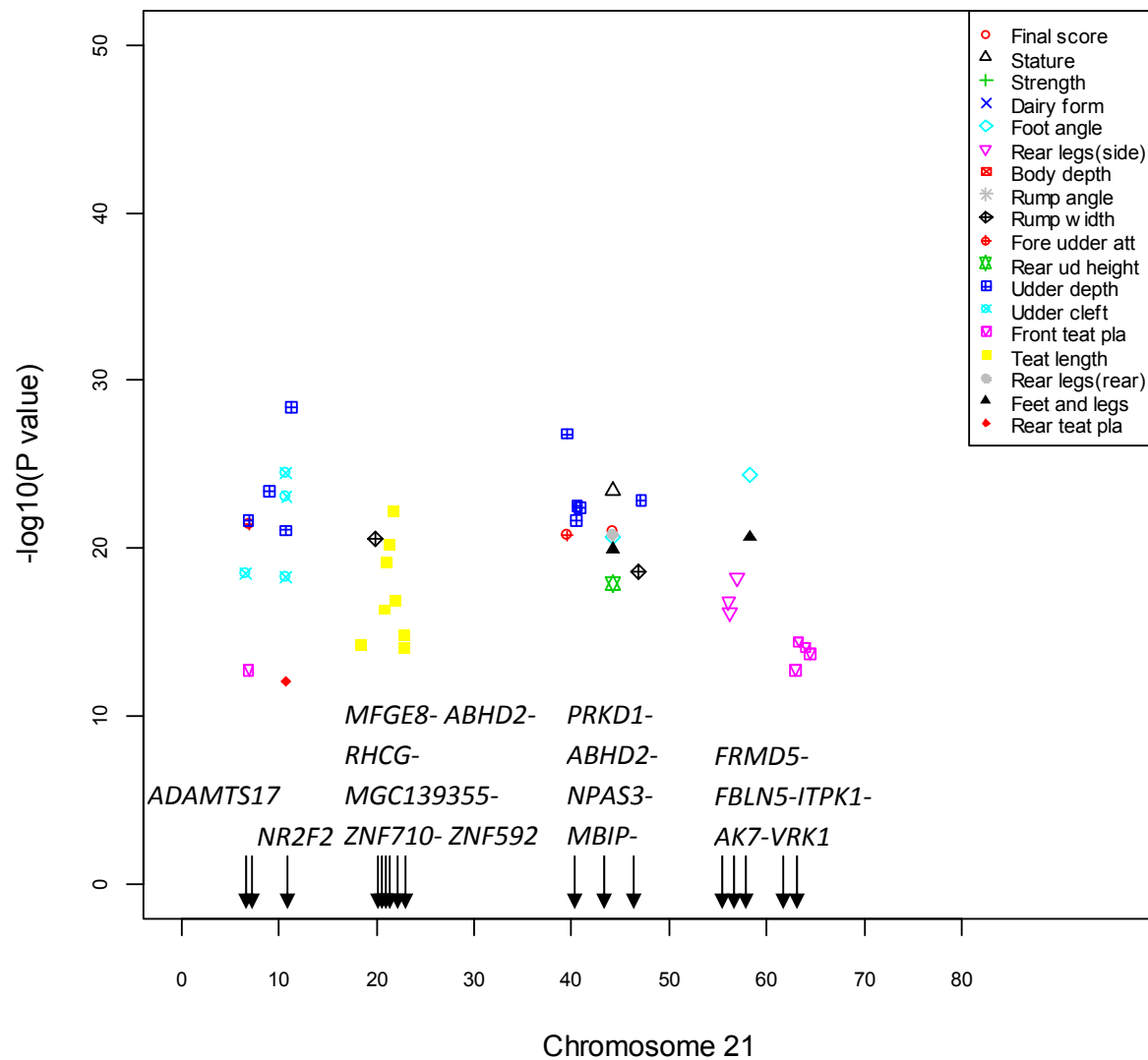

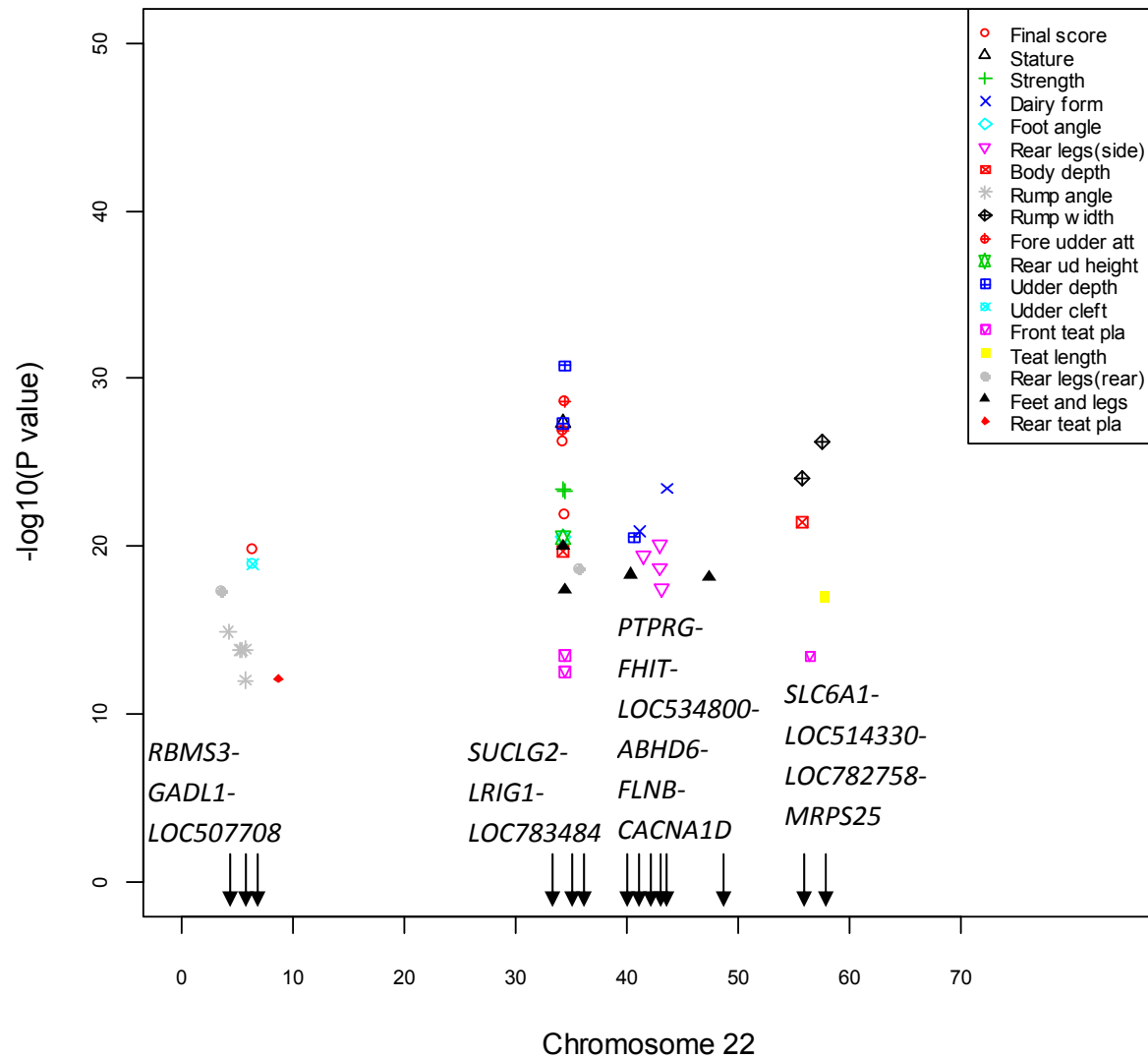

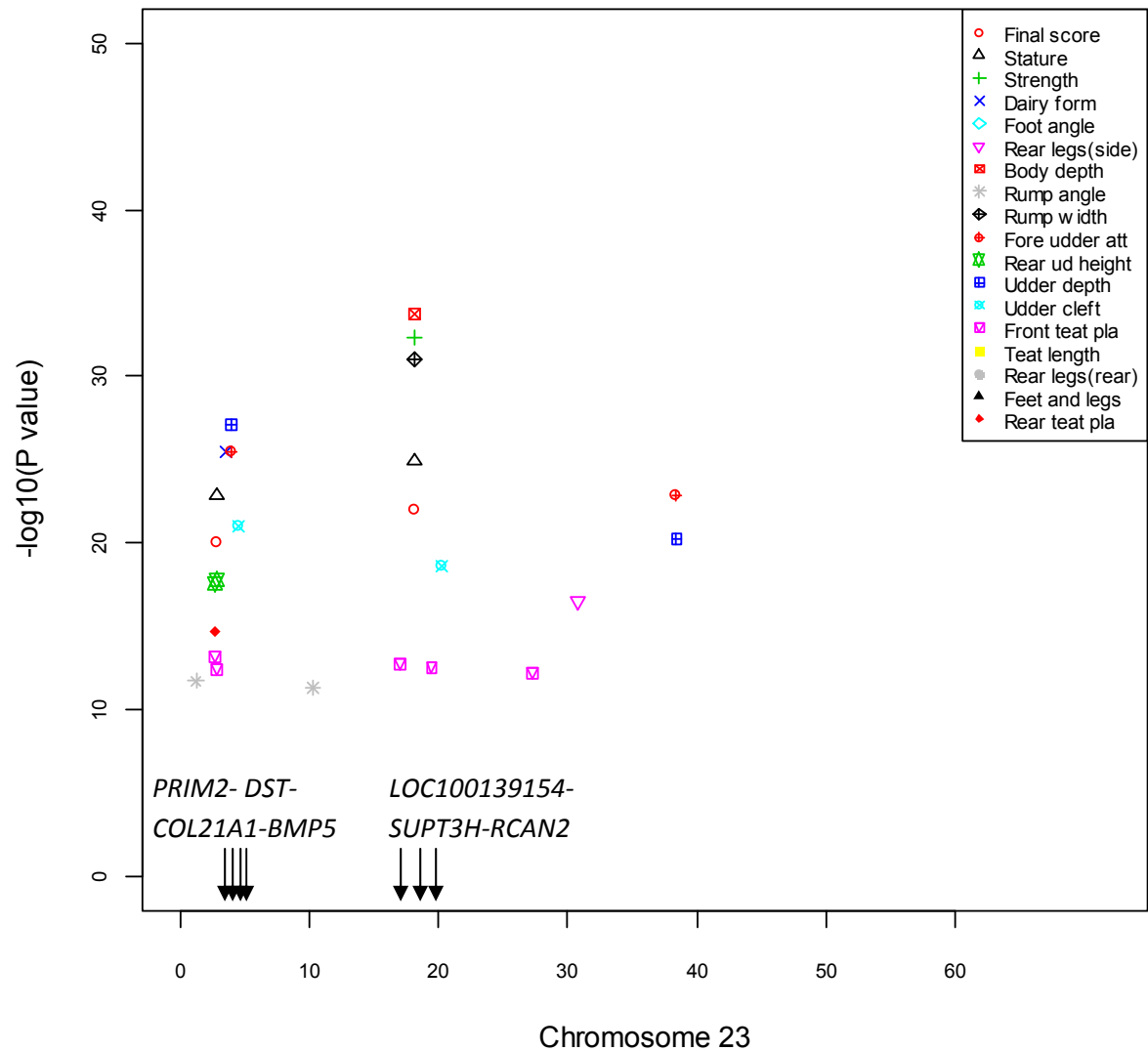

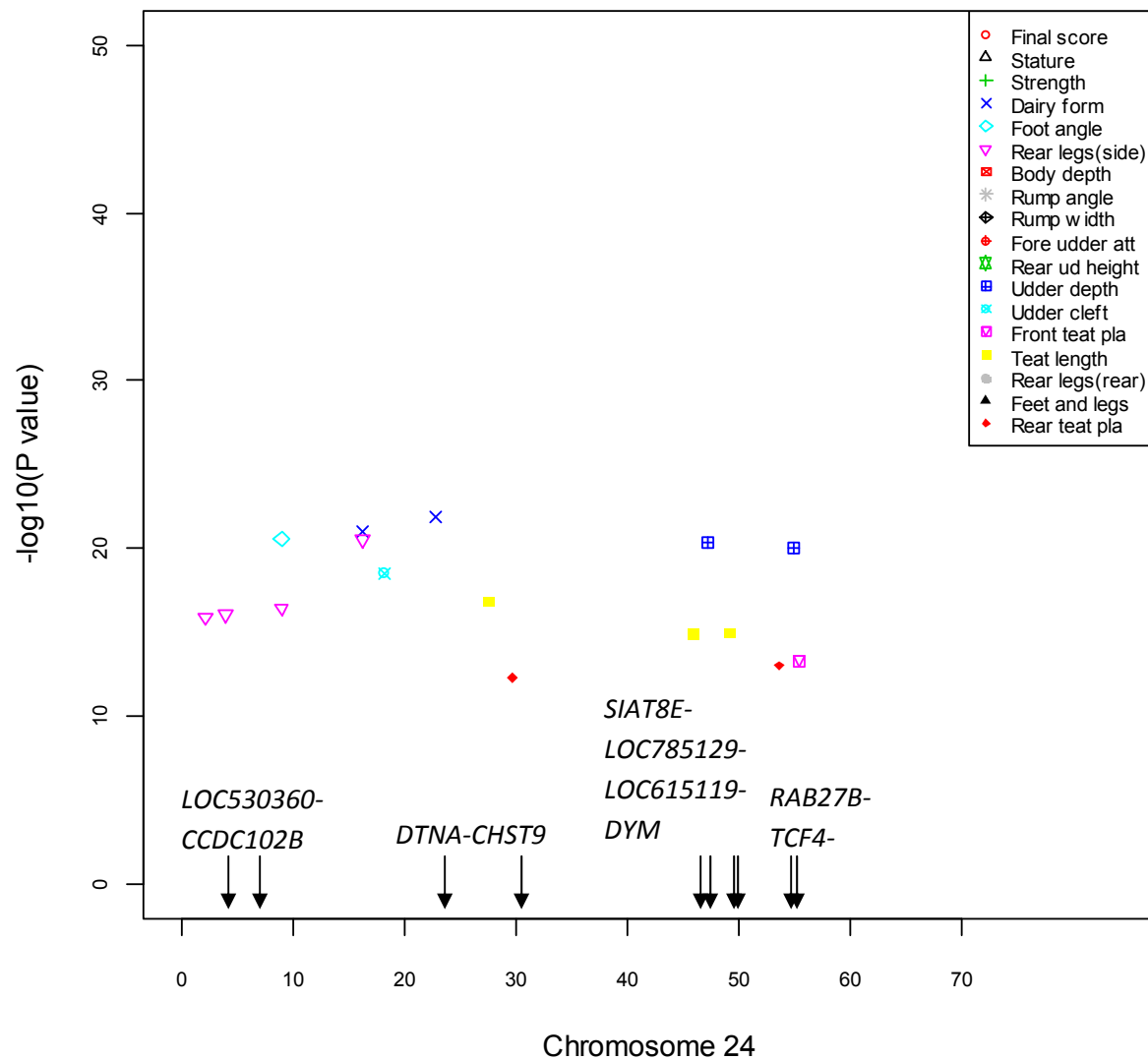

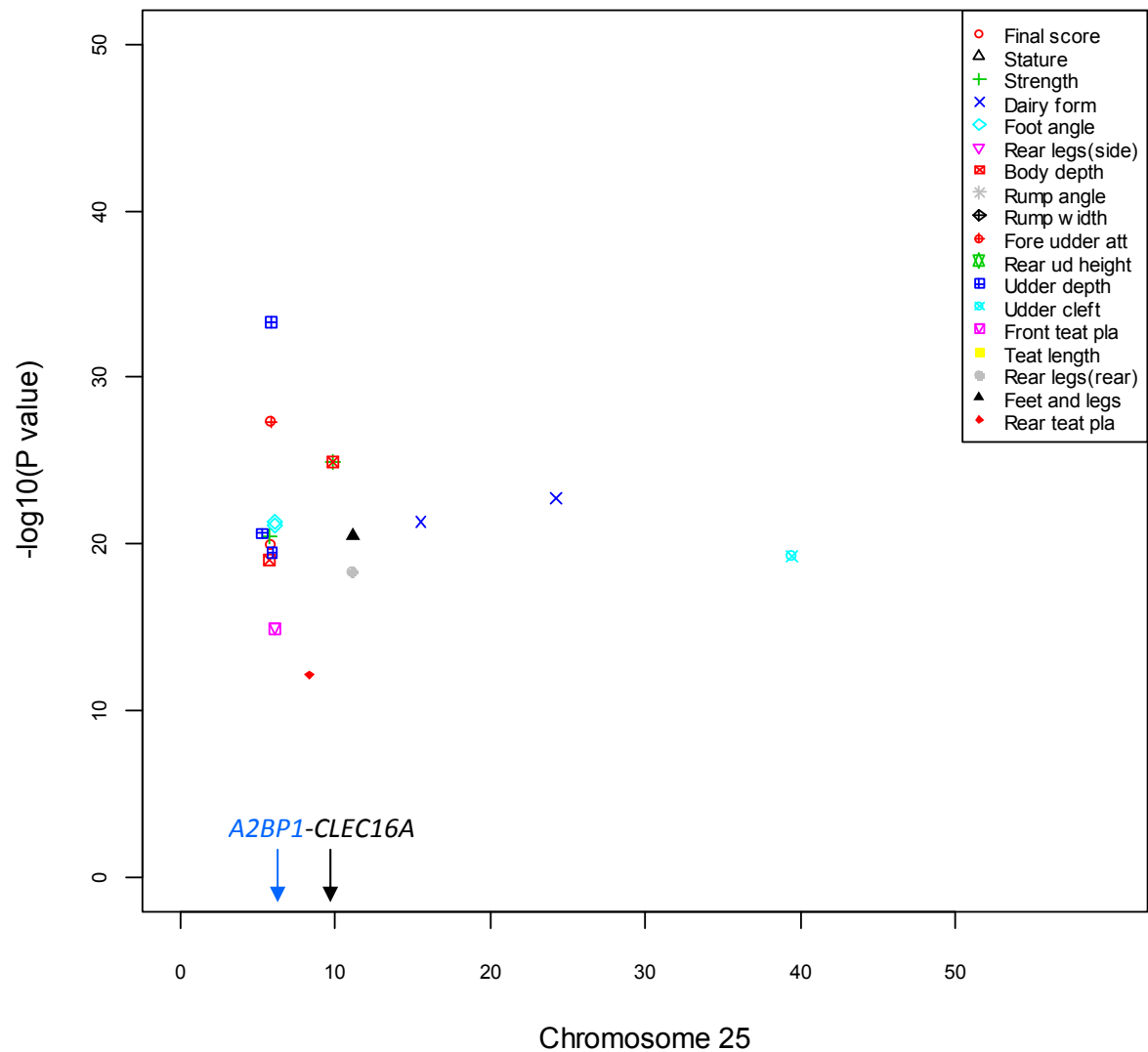

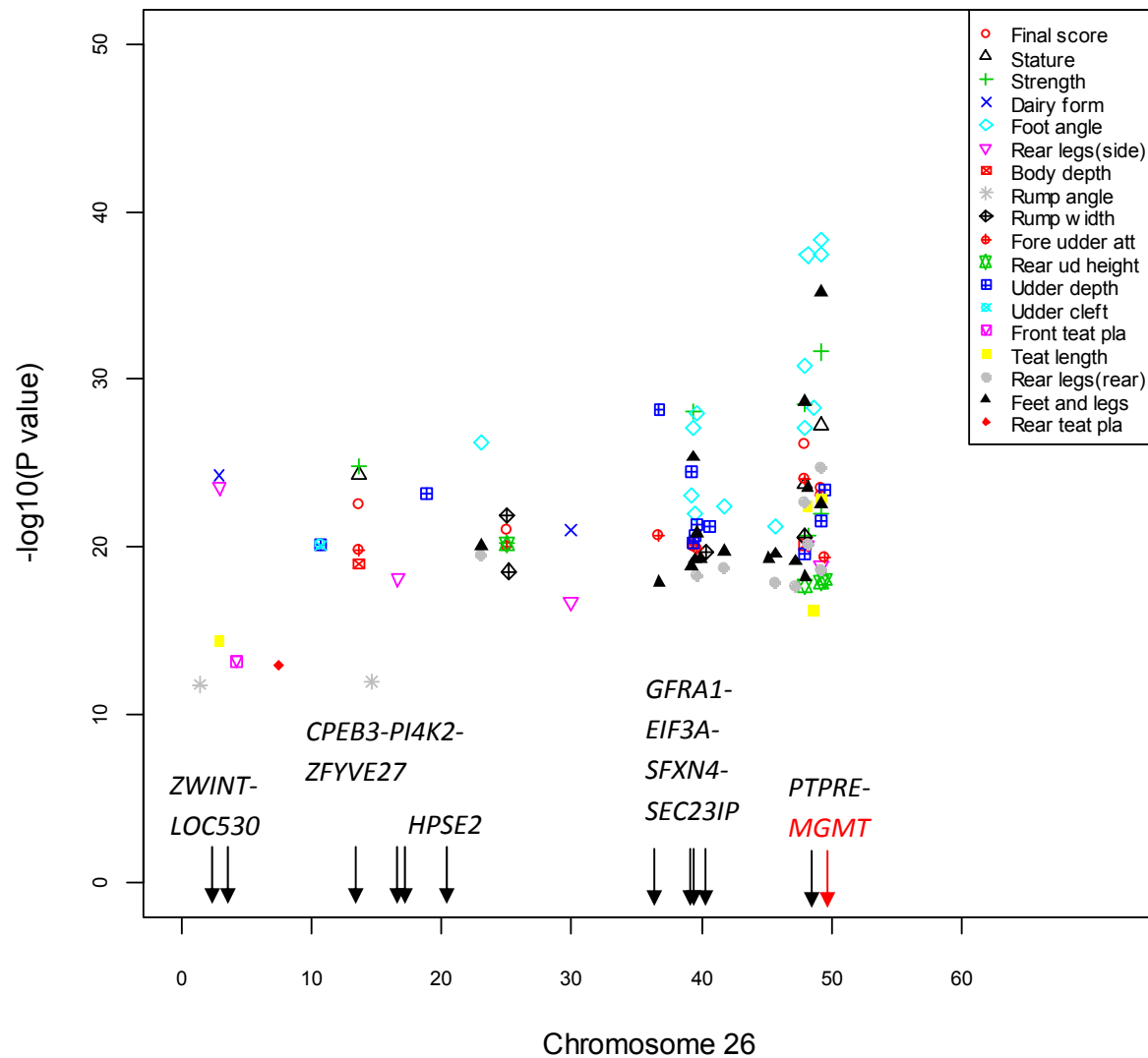

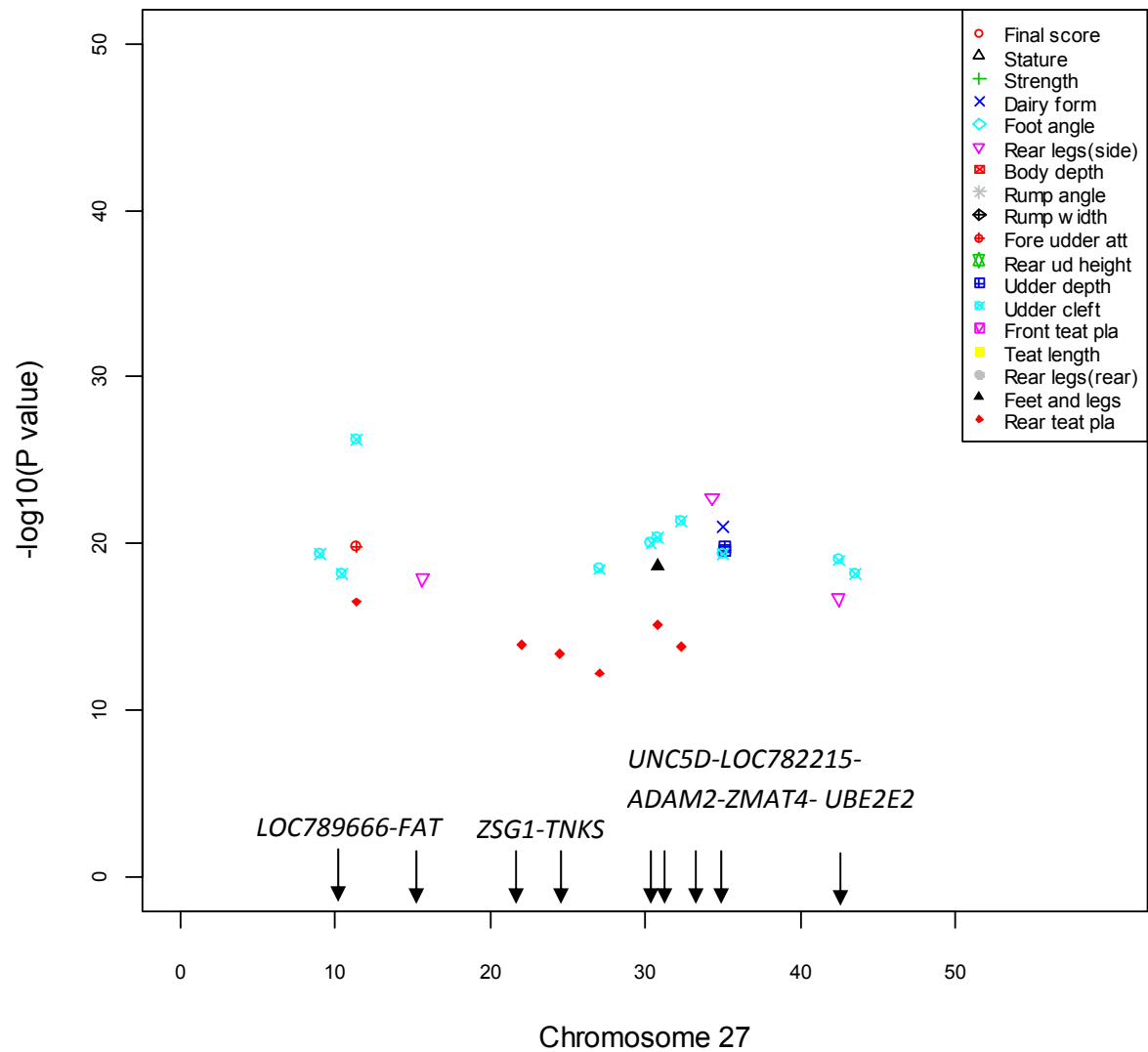

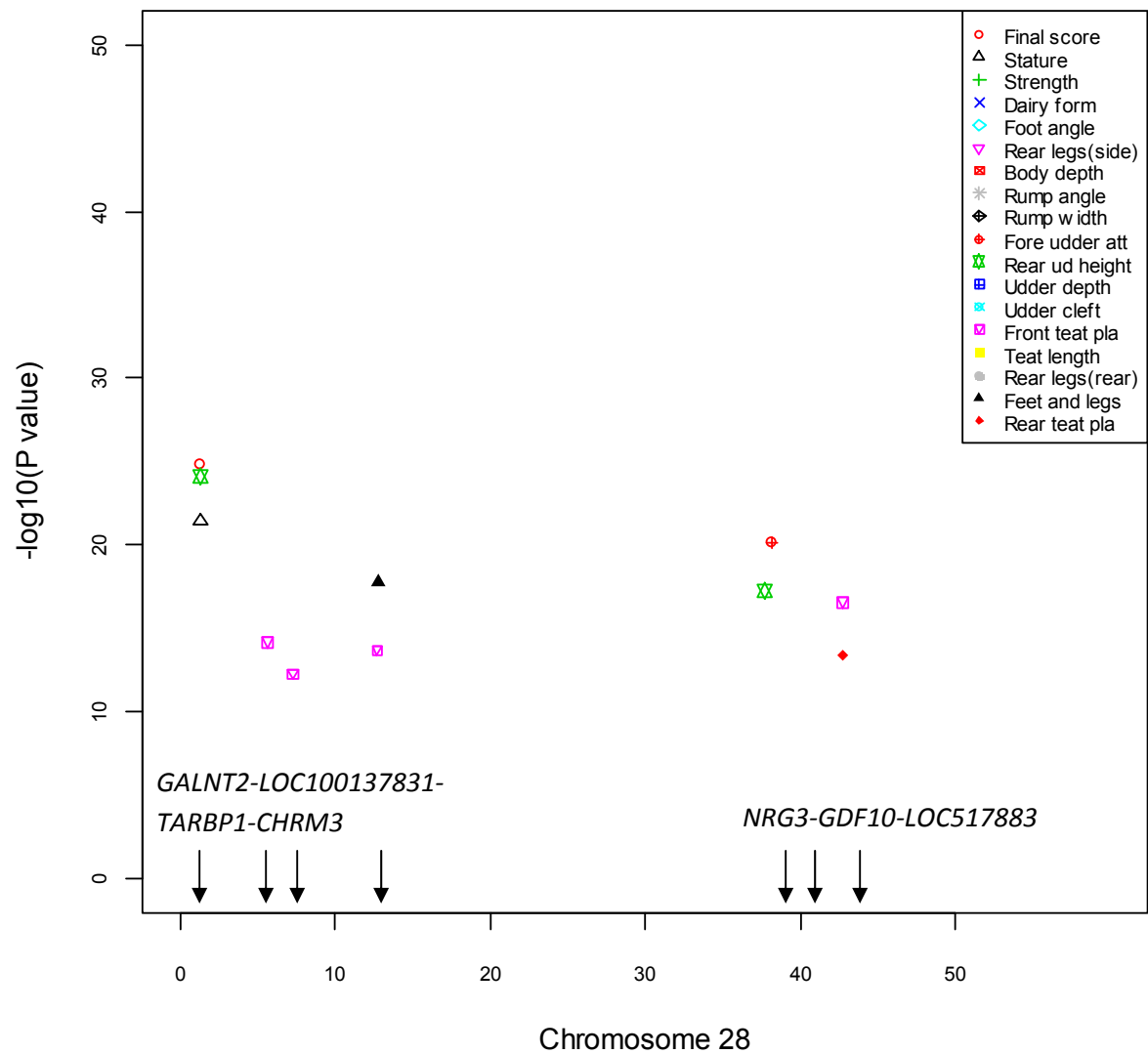

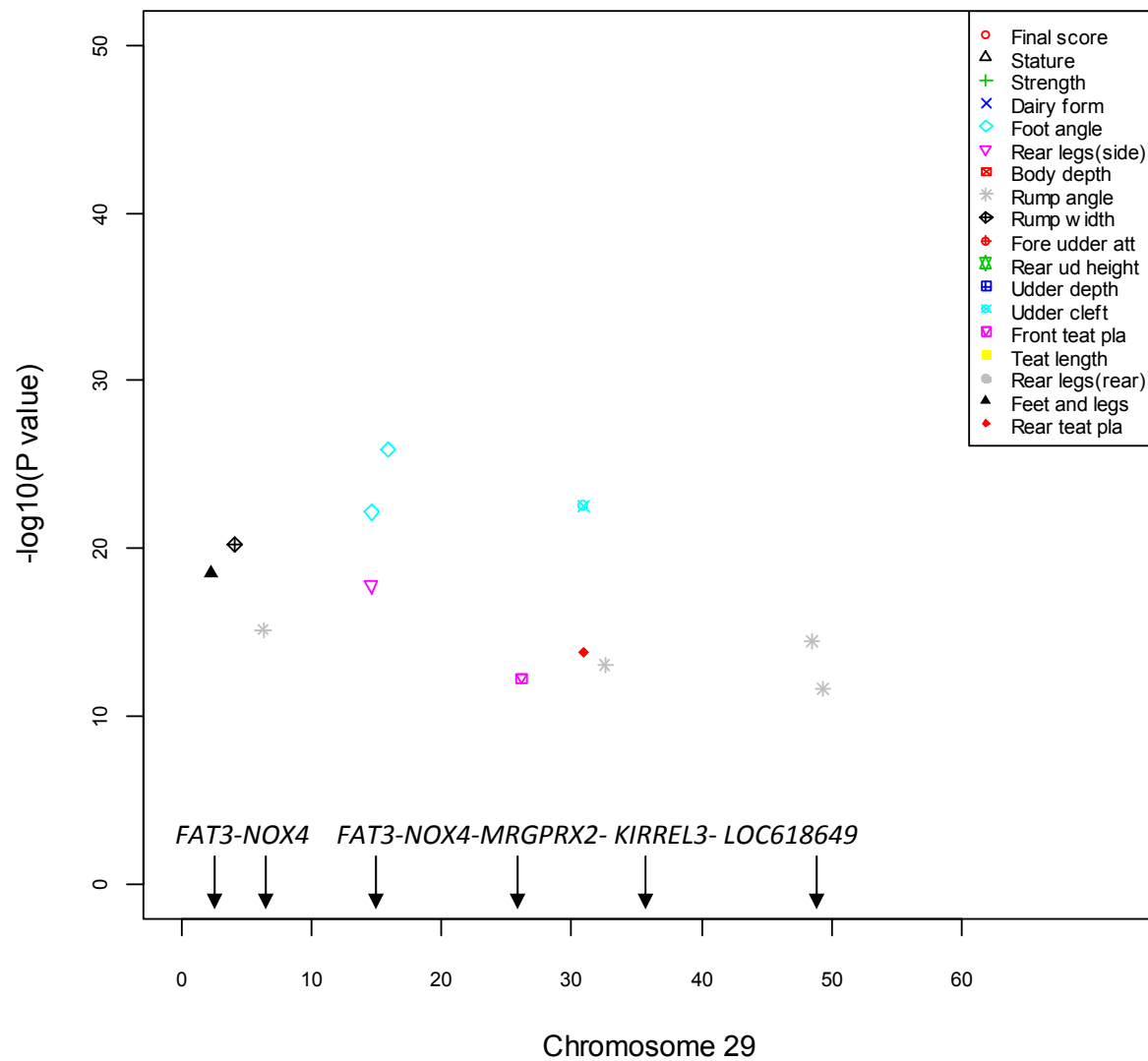

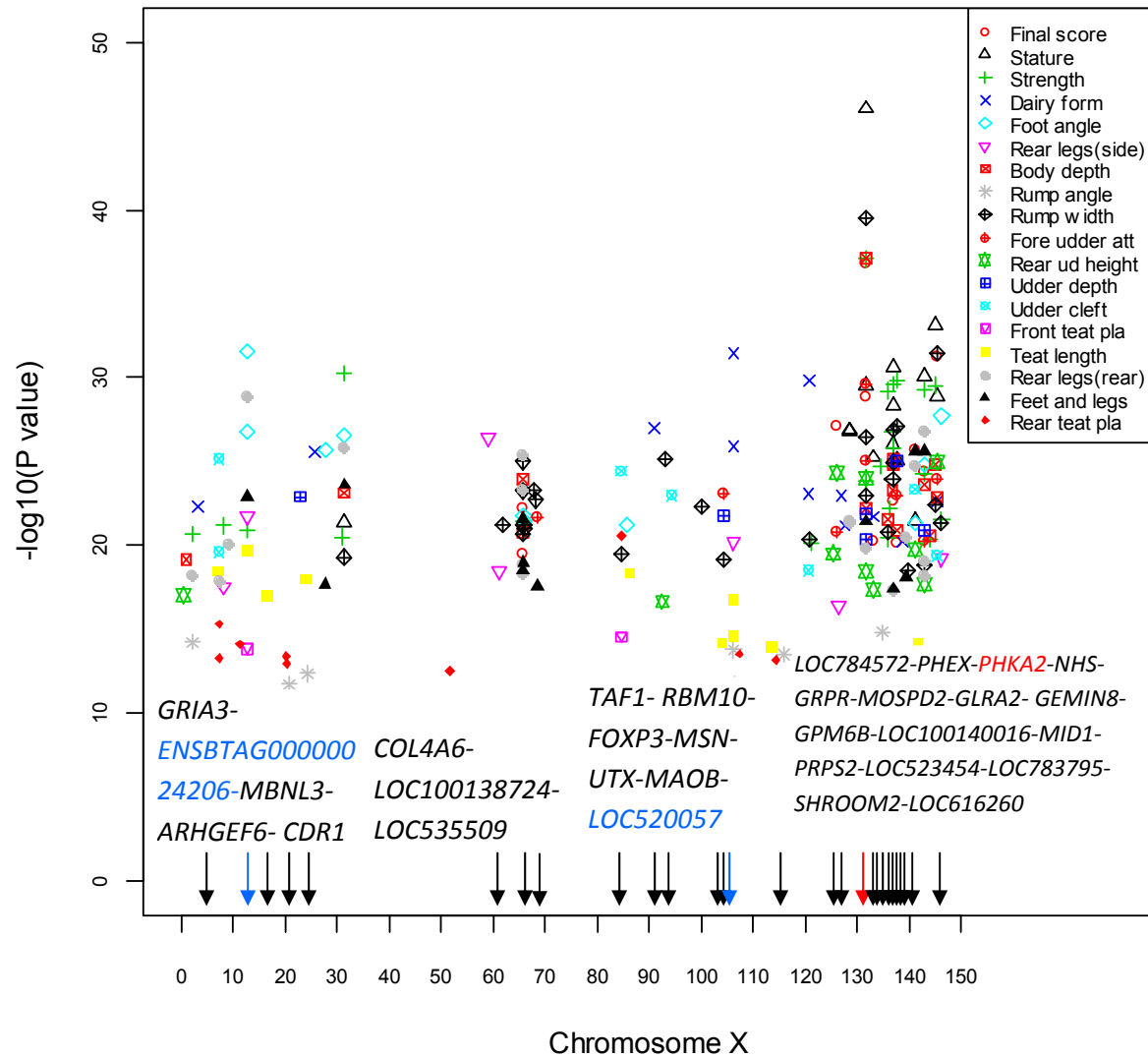

Supplement: Additional file 4 — Figure S3. Map of SNP position (Mb) by P-value and Bos taurus chromosome for 1,005 SNPs that comprise the top 100 effects for each of 18 conformation traits of contemporary U.S. Holsteins. [file 1471-2164-12-408-S4.PDF]

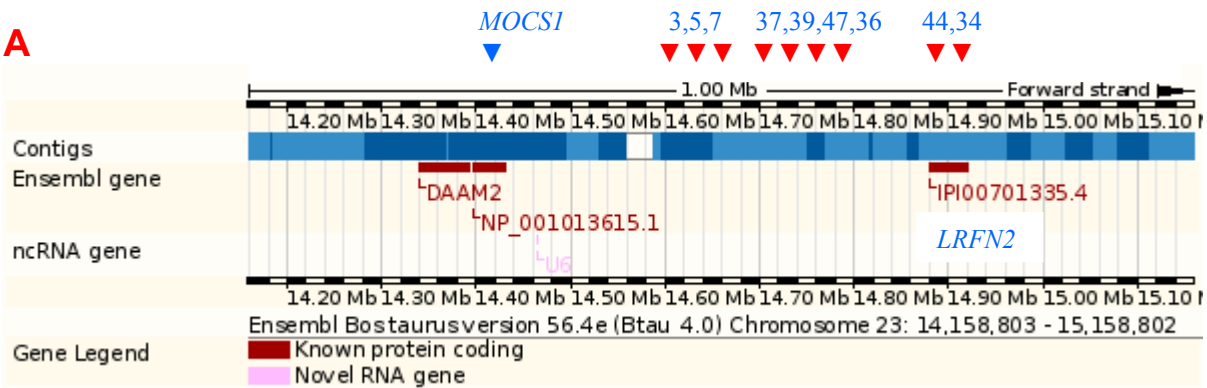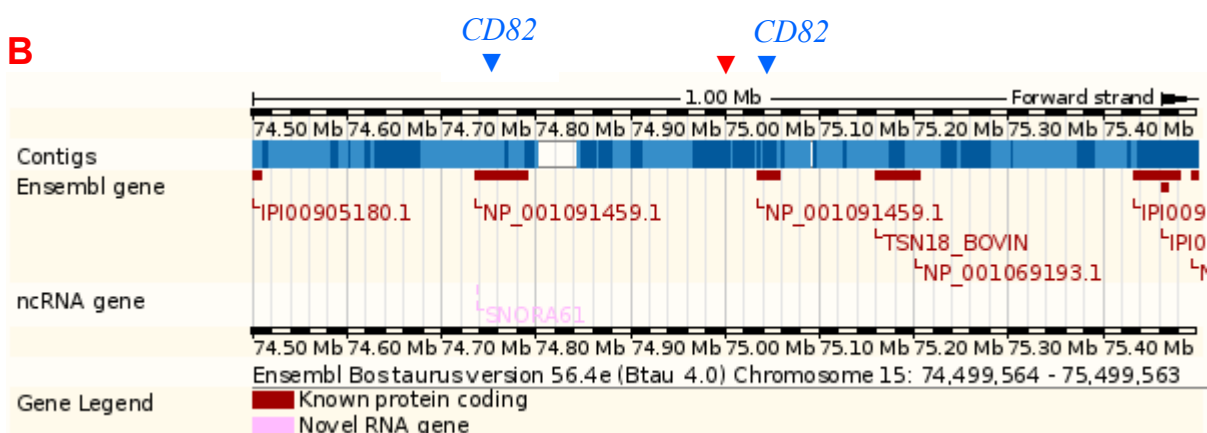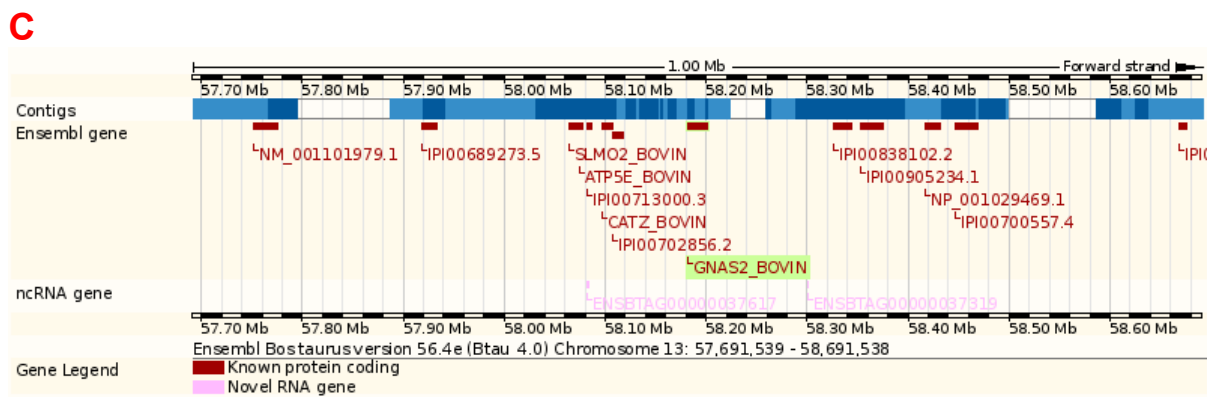

Supplement: Additional file 7 — Figure S5. Examples of gene regions associated with significant effects of SNPs for daughter stillbirth and milk yield. A) The 211.67 kb MOCS1-LRFN2 region of Bos taurus (BTA) chromosome 23 with nine highly significant effects (red arrows) for predicted transmitting ability (PTA) for daughter stillbirth. B) The most significant SNP effect (red arrow) for daughter stillbirth was 23.9 kb upstream from the second CD82 gene on BTA15. C) The BTA13 region with the most significant SNP effect (19.7 kb downstream from GNAS2 or GNAS) for PTA for milk yield. [file 1471-2164-12-408-S7.PDF]

**A**

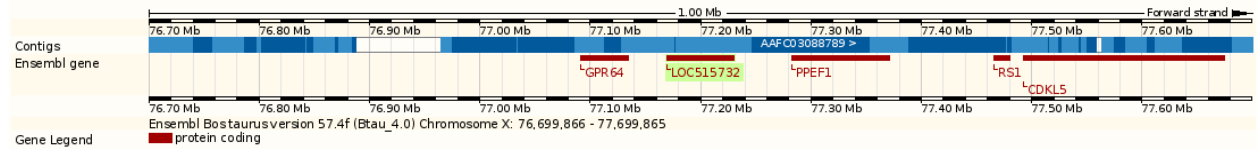

**B**

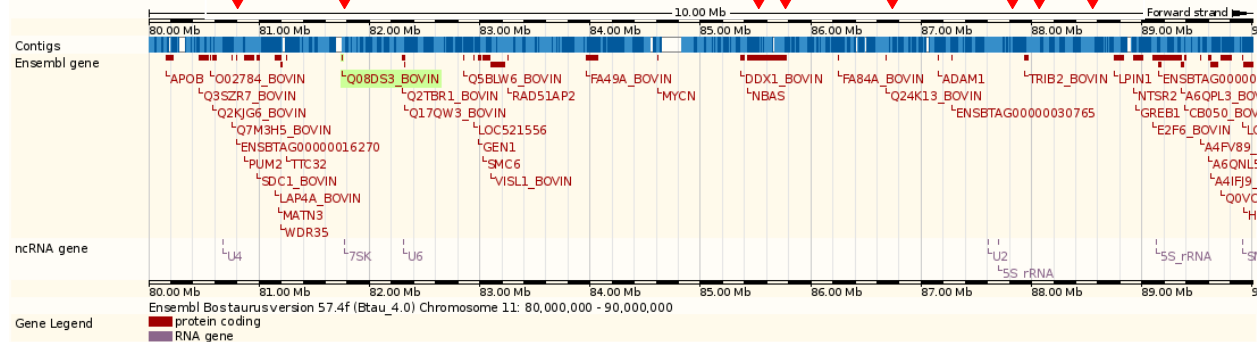

**C**

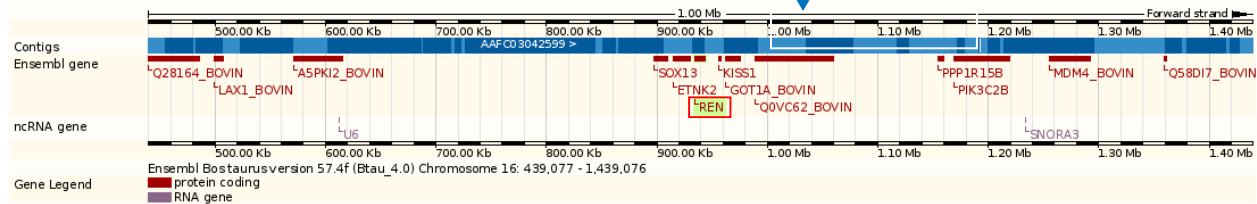

**D**

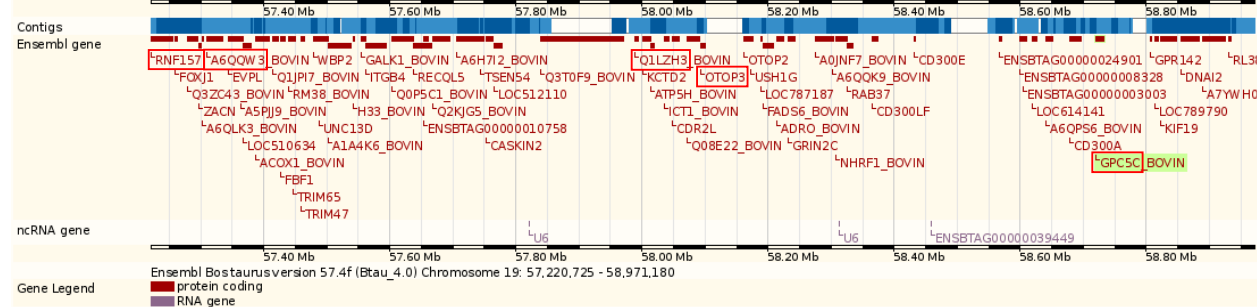

**E**

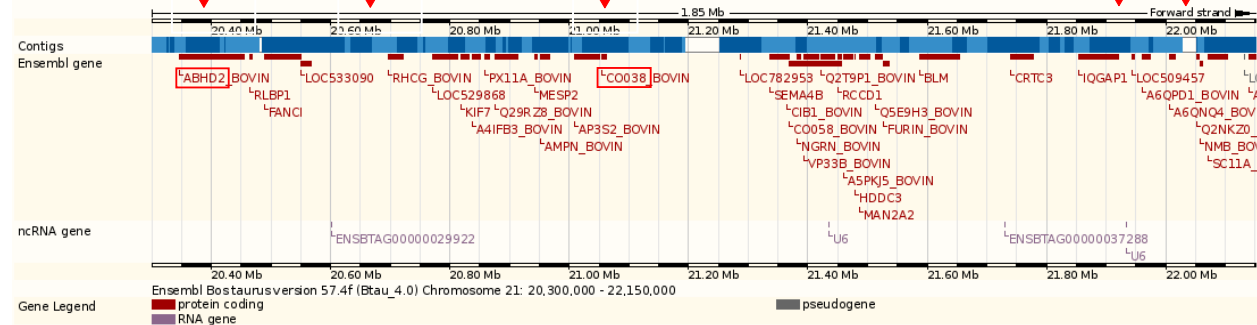

Supplement: Additional file 8 — Figure S6. Examples of gene regions associated with significant SNP effects for body conformation traits; a boxed gene contained at least one top-100 effect, and numbers above red arrows (significant SNP effects) indicate rank of SNP effect. A) The 1 Mb region of Bos taurus (BTA) chromosome X with the most significant SNP effect (LOC515732 is PHKA2) for predicted transmitting ability (PTA) for stature. B) The 10.2 Mb region of BTA 11 with eight of the top 20 SNP effects for stature. C) The 1 Mb region of BTA16 with the most significant SNP effect (REN) for PTA for strength. D) The 1.75 Mb region of BTA19 with five genes with highly significant SNP effects for rump width and udder cleft. E) A BTA21 gene cluster with three genes associated with teat length. [file 1471-2164-12-408-S8.PDF]
