# Supplementary material for: Genome-wide association analysis of thirty one production, health, reproduction and body conformation traits in contemporary U.S. Holstein cows
Source: BMC Genomics. 2011 Aug 11;12:408. doi: 10.1186/1471-2164-12-408 (PMC3176260; doi:10.1186/1471-2164-12-408)
Supplement: Additional file 5 — Table S2. Top 20 most significant SNP effects for each of the thirty one traits. [file 1471-2164-12-408-S5.PDF]

Table S2: Top 20 most significant SNP effects for each of the thirty one traits.

Table S2.1: Top 20 most significant SNP effects for PTA for milk yield

SNP	Chr	UMD position	Btau_4.0 position	A	FA	FAF	Gene region	*P* value	Effect ± SE (kg)
ARS-BFGL-BAC-12583	13	58070117	58214963	AG	G	0.291	19.7 kb D *GNAS*	1.84E-27	96 ± 8.7
BTB-01851577	3	47165828	50093240	AG	G	0.362	114 kb U *LOC781902*	1.62E-22	82 ± 8.3
Hapmap50017-BTA-52441	21	47484905	47900544	AG	G	0.220	*SLC25A21*	2.20E-22	94 ± 9.5
BTB-01141508	13	679009	679674	AC	C	0.736	85.7 kb D *TMX4*	2.95E-21	88 ± 9.2
BTA-122179-no-rs	13	1098463	1121570	AC	C	0.688	*PLCB1*	4.40E-21	85 ± 8.9
BTA-32998-no-rs	13	58043371	58276512	CG	G	0.210	81.2 kb D *GNAS*	1.51E-20	94 ± 10.0
ARS-BFGL-NGS-102011	19	63646530	64734185	AG	A	0.502	18.4 kb D *CACNG5*	3.86E-20	77 ± 8.3
BTB-01608641	3	46922624	49850076	AG	A	0.415	12 kb D *PTBP2*	1.10E-19	73 ± 8.0
BFGL-NGS-117985	18	53948569	53311437	AG	G	0.091	409 bases D *PGLYRP1*; 16 kb D *IGFL1*	1.71E-19	127 ± 13.9
BTA-33000-no-rs	13	58099969	58309152	AG	A	0.215	113.9 kb D *GNAS*	2.18E-19	90 ± 9.9
BTB-01899984	6	12357369	112027325	AG	A	0.254	1 Mb blank	3.87E-19	88 ± 9.7
BTB-01463352	3	46811548	49738986	AG	A	0.421	37.8 kb U *PTBP2*	3.99E-19	72 ± 8.0
BTB-01463390	3	46842537	49778569	AC	C	0.429	*PTBP2*	7.00E-19	72 ± 8.0
ARS-BFGL-NGS-105981	24	61062843	63230092	AG	A	0.677	24.5 kb D *PIGN*	1.08E-18	78 ± 8.8
BTB-01063707	27	28386241	31290289	AG	G	0.173	*FUT10*	1.56E-18	95 ± 10.7
BTA-83605-no-rs	9	46739973	48362592	AG	A	0.359	1 Mb blank	4.67E-18	74 ± 8.5
ARS-BFGL-NGS-42400	24	47413118	48636081	AT	A	0.643	15.8 kb D *LOC531776*	7.53E-18	74 ± 8.6
BTB-00196579	4	73367338	75364129	AG	A	0.061	424 bases U *LOC781500*	8.00E-18	67 ± 16.9
Hapmap42685-BTA-81134	8	49723924	51778383	AG	A	0.835	87.2 kb D *ANXA1*	8.86E-18	95 ± 10.9
ARS-BFGL-NGS-19142	10	90279946	91803114	AG	A	0.306	51 kb D *LOC100140505*	1.12E-17	75 ± 8.7

SNP, single nucleotide polymorphism; PTA, predicted transmitting ability; Chr, chromosome; UMD, University of Maryland; Btau_4.0, Baylor College of Medicine bovine genome assembly Build 4.0; A, SNP alleles; FA, favorable allele; FAF, FA frequency; U, upstream from; D, downstream from; SE, standard error.

Table S2.2: Top 20 most significant SNP effects for PTA for fat yield

SNP	Chr	UMD position	Btau_4.0 position	A	FA	FAF	Gene region	*P* value	Effect ± SE (kg)
BFGL-NGS-117985	18	539485697	5331143	AG	G	0.091	409 bases D *PGLYRP1*; 16 kb D *IGFL1*	9.83E-55	9.2 ± 0.57
Hapmap51582-BTA-31036	X	146244046	86367264	AG	A	0.096	113.2 kb D *LOC616260*	1.38E-50	8.5 ± 0.55
BTB-01333903	1	28362687	29095948	AG	G	0.067	220.9 kb U *FKBP2*	1.68E-49	10.0 ± 0.66
ARS-BFGL-BAC-12583	13	58070117	58214963	AG	G	0.291	33.2 kb U *GNAS*	2.80E-49	5.6 ± 0.37
BTA-32998-no-rs	13	58043371	58276512	CG	G	0.210	81.2 kb D *GNAS*	5.19E-46	6.1 ± 0.41
ARS-BFGL-BAC-29490	23	20166517	20900919	AG	A	0.112	*GPR110–GPR116*	1.45E-45	6.0 ± 0.53
BTB-01063707	27	28386241	31290289	AG	G	0.173	*FUT10*	1.55E-45	6.4 ± 0.44
BTA-33000-no-rs	13	58099969	58309152	AG	A	0.215	113.9 kb D *GNAS*	2.14E-45	6.0 ± 0.41
ARS-BFGL-NGS-1096	X	12604306	5329598	AG	A	0.074	1 Mb blank	3.66E-44	9.3 ± 0.65
ARS-BFGL-NGS-13673	1	87402236	88871762	AC	A	0.090	428.3 kb D *TTC14*	2.23E-43	8.2 ± 0.58
Hapmap51258-BTA-55319	X	135530009	80273442	AG	G	0.123	*LPP*	5.65E-41	6.9 ± 0.50
BTA-110943-no-rs	27	29524324	31986530	AG	A	0.118	1 Mb blank	1.57E-40	7.2 ± 0.52
Hapmap50642-BTA-28388	26	49137602	49579691	AG	G	0.089	29.9 kb U *MGMT*	1.76E-40	7.9 ± 0.58
BTA-03029-rs29010796	18	6752549	5696451	AC	A	0.124	200.5 kb D *MAF*	3.53E-40	6.9 ± 0.51
BFGL-NGS-111445	26	48120342	48539159	AG	A	0.089	1 Mb blank	1.66E-39	7.9 ± 0.59
BTA-16909-no-rs	X	136196550	336258	AC	C	0.124	*GLRA2*	5.10E-39	6.8 ± 0.50
BFGL-NGS-119420	13	59101909	59330402	AG	A	0.227	*ZBP1*	2.08E-38	5.3 ± 0.40
BTB-01297598	17	14080531	14889608	AG	A	0.151	384.6 kb U *HHIP*	8.97E-38	6.1 ± 0.46
ARS-BFGL-NGS-10101	5	15010779	441173	AG	A	0.263	10.5 kb U *LOC782348*	2.24E-37	4.8 ± 0.36
BTA-83605-no-rs	9	46739973	48362592	AG	A	0.359	1 Mb blank	1.63E-35	4.5 ± 0.35

SNP, single nucleotide polymorphism; PTA, predicted transmitting ability; Chr, chromosome; UMD, University of Maryland; Btau_4.0, Baylor College of Medicine bovine genome assembly Build 4.0; A, SNP alleles; FA, favorable allele; FAF, FA frequency; U, upstream from; D, downstream from; SE, standard error.

Table S2.3: Top 20 most significant SNP effects for PTA for protein yield

SNP	Chr	UMD position	Btau_4.0 position	A	FA	FAF	Gene region	*P* value	Effect ± SE (kg)
BFGL-NGS-117985	18	53948569	53311437	AG	G	0.091	409 bases D *PGLYRP1*; 16 kb D *IGFL1*	1.43E-49	6.4 ± 0.42
BTB-01333903	1	28362687	29095948	AG	G	0.067	220.9 kb U *FKBP2*	9.96E-47	7.1 ± 0.48
Hapmap51582-BTA-31036	X	146244046	86367264	AG	A	0.096	113.2 kb D *LOC616260*	9.61E-46	5.9 ± 0.40
ARS-BFGL-BAC-29490	23	20166517	20900919	AG	A	0.112	*GPR110–GPR116*	1.16E-41	5.4 ± 0.39
ARS-BFGL-BAC-12583	13	58070117	58214963	AG	G	0.291	19.7 kb D *GNAS*	2.05E-41	3.7 ± 0.27
BTA-110943-no-rs	27	29524324	31986530	AG	A	0.118	1 Mb blank	4.78E-41	5.3 ± 0.38
BTB-01063707	27	28386241	31290289	AG	G	0.173	*FUT10*	9.70E-40	4.4 ± 0.32
BTA-16909-no-rs	X	136196550	336258	AC	C	0.124	*GLRA2*	2.85E-39	4.9 ± 0.37
ARS-BFGL-NGS-19953	21	31251702	30783968	AG	G	0.245	6.4 kb U *CRABP1*	3.86E-39	3.8 ± 0.28
Hapmap50642-BTA-28388	26	49137602	49579691	AG	G	0.089	29.9 kb U *MGMT*	1.15E-38	5.7 ± 0.42
ARS-BFGL-NGS-1096	X	12604306	5329598	AG	A	0.074	1 Mb blank	1.90E-38	6.4 ± 0.48
BTB-01297598	17	14080531	14889608	AG	A	0.151	384.6 kb U *HHIP*	8.80E-38	4.4 ± 0.33
BTA-03029-rs29010796	18	6752549	5696451	AC	A	0.124	200.5 kb D *MAF*	9.03E-38	4.9 ± 0.37
ARS-BFGL-NGS-13673	1	87402236	88871762	AC	A	0.090	428.3 kb D *TTC14*	1.33E-37	5.5 ± 0.42
BFGL-NGS-111445	26	48120342	48539159	AG	A	0.089	1 Mb blank	1.43E-37	5.6 ± 0.43
BTA-32998-no-rs	13	58043371	58276512	CG	G	0.210	81.2 kb D *GNAS*	1.74E-35	3.9 ± 0.31
ARS-BFGL-NGS-4840	X	136328915	468626	AG	G	0.128	18.1 kb D *LAMP2*	7.43E-35	4.6 ± 0.37
Hapmap53617-rs29011699	23	32975635	33336457	AG	G	0.173	*ALDH5A1*	7.79E-35	4.1 ± 0.33
BTA-95509-no-rs	X	59080285	331965	AG	A	0.185	25.7 kb U *ATP1B4*	7.90E-35	4.0 ± 0.32
BTA-33000-no-rs	13	58099969	58309152	AG	A	0.215	113.9 kb D *GNAS*	3.17E-34	3.8 ± 0.30

SNP, single nucleotide polymorphism; PTA, predicted transmitting ability; Chr, chromosome; UMD, University of Maryland; Btau_4.0, Baylor College of Medicine bovine genome assembly Build 4.0; A, SNP alleles; FA, favorable allele; FAF, FA frequency; U, upstream from; D, downstream from; SE, standard error.

Table S2.4: Top 20 most significant SNP effects for PTA for fat percentage

SNP	Chr	UMD position	Btau_4.0 position	A	FA	FAF	Gene region	*P* value	Effect ± SE (%)
ARS-BFGL-NGS-4939	14	1801116	443937	AG	G	0.183	*DGAT1*	4.21E-45	0.04 ± 0.003
ARS-BFGL-NGS-56327	14	4336714	2580414	AG	G	0.322	*NIBP*	5.54E-43	0.03 ± 0.002
ARS-BFGL-NGS-57820	14	1651311	236532	AG	G	0.187	3.4 kb U *FOXH1*	4.28E-40	0.04 ± 0.003
ARS-BFGL-NGS-1096	X	12604306	5329598	AG	A	0.074	1 Mb blank	1.63E-38	0.06 ± 0.004
ARS-BFGL-NGS-100480	14	4364952	2607583	AG	G	0.381	*NIBP*	1.78E-36	0.03 ± 0.002
ARS-BFGL-NGS-18858	14	2909929	297711	AG	A	0.314	15 kb D *VPS28*	3.48E-35	0.03 ± 0.002
Hapmap30383-BTC-005848	14	1489496	76703	AG	A	0.361	*C14H8orf33–ZNF34*	1.07E-34	0.03 ± 0.002
ARS-BFGL-NGS-107379	14	2054457	679600	AG	G	0.249	460 bases U *LOC786966*	1.02E-30	0.03 ± 0.002
BFGL-NGS-116999	5	93225456	99656229	AC	C	0.221	469 kb U *LMO3*	1.05E-30	0.03 ± 0.003
Hapmap50642-BTA-28388	26	49137602	49579691	AG	G	0.089	29.9 kb U *MGMT*	1.06E-29	0.04 ± 0.004
UA-IFASA-6670	5	100206147	107086994	AG	A	0.385	*GABARAPL1*	2.76E-29	0.03 ± 0.002
BFGL-NGS-111445	26	48120342	48539159	AG	A	0.089	1 Mb blank	9.53E-28	0.04 ± 0.004
Hapmap51582-BTA-31036	X	146244046	86367264	AG	A	0.096	113.2 kb D *LOC616260*	1.08E-27	0.04 ± 0.004
BTB-01333903	1	28362687	29095948	AG	G	0.067	220.9 kb U *FKBP2*	2.73E-27	0.05 ± 0.004
ARS-BFGL-NGS-1312	26	48583446	48998305	AG	A	0.107	1 Mb blank	8.17E-27	0.04 ± 0.003
BFGL-NGS-110993	14	4808166	3059045	AC	A	0.426	65.6 kb D *KCNK9*	6.38E-26	0.02 ± 0.002
ARS-BFGL-NGS-38247	X	60363808	88311	AG	A	0.201	269.4 kb U *ATP1B4*	9.33E-26	0.03 ± 0.003
ARS-BFGL-NGS-104215	X	110719987	65759058	AC	A	0.298	*SYTL5*	3.28E-25	0.02 ± 0.002
ARS-BFGL-NGS-34106	17	66619908	67755947	AG	G	0.660	79.6 kb U *SART3*	4.65E-25	0.02 ± 0.002
Hapmap30646-BTC-002054	14	2553525	1461085	AG	A	0.608	1.3 kb U *GPIHBP1*	4.97E-25	0.02 ± 0.002

SNP, single nucleotide polymorphism; PTA, predicted transmitting ability; Chr, chromosome; UMD, University of Maryland; Btau_4.0, Baylor College of Medicine bovine genome assembly Build 4.0; A, SNP alleles; FA, favorable allele; FAF, FA frequency; U, upstream from; D, downstream from; SE, standard error.

Table S2.5: Top 20 most significant SNP effects for PTA for protein percentage

SNP	Chr	UMD position	Btau_4.0 position	A	FA	FAF	Gene region	*P* value	Effect ± SE (%)
ARS-BFGL-NGS-1096	X	12604306	5329598	AG	A	0.074	1 Mb Blank	4.83E-62	0.03 ± 0.002
Hapmap50642-BTA-28388	26	49137602	49579691	AG	G	0.089	29.9 kb U *MGMT*	3.12E-52	0.03 ± 0.002
BFGL-NGS-111445	26	48120342	48539159	AG	A	0.089	1 Mb blank	6.46E-48	0.03 ± 0.002
BTB-01333903	1	28362687	29095948	AG	G	0.067	220.9 kb U *FKBP2*	2.65E-42	0.03 ± 0.002
ARS-BFGL-BAC-34040	17	18764770	19749801	AC	C	0.108	2.9 kb D *LOC528054*	8.64E-41	0.02 ± 0.002
Hapmap51582-BTA-31036	X	146244046	86367264	AG	A	0.096	113.2 kb D *LOC616260*	2.70E-40	0.02 ± 0.002
ARS-BFGL-NGS-1312	26	48583446	48998305	AG	A	0.107	1 Mb blank	1.03E-39	0.02 ± 0.002
ARS-BFGL-NGS-60213	X	103580973	61455528	AG	A	0.203	390 kb U *CX036*	1.92E-39	0.02 ± 0.001
ARS-BFGL-NGS-61325	X	24505176	14038654	AC	C	0.216	85.7 kb D *LOC781178*	1.90E-38	0.02 ± 0.001
Hapmap28373-BTA-160078	X	24528596	14063338	CG	G	0.216	110.4 kb D *LOC781178*	2.19E-38	0.02 ± 0.002
ARS-BFGL-NGS-16708	17	15107947	15883911	AC	A	0.127	187.4 kb D *USP38*	2.27E-38	0.02 ± 0.002
BTA-46636-no-rs	17	26418537	27832494	AT	A	0.130	1 Mb blank	2.82E-37	0.02 ± 0.002
ARS-BFGL-NGS-107195	17	26398481	27812438	AG	A	0.183	1 Mb blank	2.98E-37	0.02 ± 0.001
ARS-BFGL-NGS-94891	18	24286893	23782882	AG	A	0.162	4.4 kb D *NUDT21*	1.10E-35	0.02 ± 0.001
ARS-BFGL-NGS-58480	X	61129008	34617065	AG	G	0.129	*COL4A6*	3.79E-35	0.02 ± 0.002
Hapmap26269-BTC-041695	6	71452210	72377594	AG	G	0.304	30.9 kb D *PDGFRA*	4.48E-35	0.01 ± 0.001
Hapmap32220-BTC-042831	6	71552977	72478576	AG	G	0.301	131.9 kb D *PDGFRA*	3.94E-34	0.01 ± 0.001
ARS-BFGL-NGS-36745	5	113411233	120038094	AG	A	0.157	*SREBF2*	4.31E-34	0.02 ± 0.001
ARS-BFGL-NGS-56327	14	4336714	2580414	AG	G	0.322	*NIBP*	9.13E-34	0.01 ± 0.001
ARS-BFGL-NGS-26226	9	16342252	16017242	AG	A	0.211	16.1Kb D *RPL37*	4.73E-33	0.02 ± 0.001

SNP, single nucleotide polymorphism; PTA, predicted transmitting ability; Chr, chromosome; UMD, University of Maryland; Btau_4.0, Baylor College of Medicine bovine genome assembly Build 4.0; A, SNP alleles; FA, favorable allele; FAF, FA frequency; U, upstream from; D, downstream from; SE, standard error.

Table S2.6: Top 20 most significant SNP effects for PTA for productive life

SNP	Chr	UMD position	Btau_4.0 position	A	FA	FAF	Gene region	*P* value	Effect ± SE (mo)
ARS-BFGL-NGS-18028	X	106241123	63961868	CG	C	0.131	30.1 kb U *LOC520057*	8.33E-66	1.7 ± 0.09
BTA-29287-no-rs	18	58696066	58353743	AG	A	0.114	*LOC787057*	1.70E-58	1.6 ± 0.10
ARS-BFGL-NGS-4774	7	17403976	14635617	AC	A	0.323	1.5K k D *INSR* (*Q95M43*)	2.07E-57	1.1 ± 0.07
BTA-94079-no-rs	X	106280810	64001706	AG	G	0.111	8.1 kb D *LOC520057*	3.31E-56	1.7 ± 0.10
ARS-BFGL-NGS-43785	X	3167117	277676	AG	A	0.245	80 kb U *ATP1B4*	4.42E-52	1.2 ± 0.07
BTB-01333903	1	28362687	29095948	AG	G	0.067	220.9 kb U *FKBP2*	2.12E-47	1.9 ± 0.13
ARS-BFGL-NGS-13673	1	87402236	88871762	AC	A	0.090	428.3 kb D *TTC14*	1.77E-46	1.6 ± 0.11
ARS-BFGL-NGS-36483	X	25731651	14983892	AG	A	0.271	342 kb D *LOC531026*	3.86E-46	1.1 ± 0.07
BTA-100861-no-rs	26	40648090	40780925	AG	A	0.286	234.4 kb U *PPAPDC1A*	5.20E-46	1.1 ± 0.07
ARS-BFGL-NGS-58480	X	61129008	34617065	AG	G	0.129	*COL4A6*	7.35E-46	1.4 ± 0.10
BTA-31244-no-rs	X	NA	15607221	AC	A	0.362	144.3 kb U *SLITRK4*	8.54E-45	1.0 ± 0.07
ARS-BFGL-NGS-1096	X	12604306	5329598	AG	A	0.074	1 Mb blank	5.69E-44	1.8 ± 0.13
ARS-BFGL-BAC-13086	1	25289457	25652144	CG	C	0.380	*LOC100138356*	2.53E-43	1.0 ± 0.07
BFGL-NGS-112365	18	64548360	64574451	AG	A	0.227	*LOC100137761*	3.63E-43	1.1 ± 0.08
BFGL-NGS-111315	7	13584721	10738454	AC	A	0.182	*TRMT4–LYL1*	2.52E-42	1.2 ± 0.08
BFGL-NGS-117985	18	53948569	53311437	AG	G	0.091	409 bases D *PGLYRP1*; 16 kb D *IGFL1*	2.76E-42	1.6 ± 0.11
Hapmap39671-BTA-62550	X	90910050	56543500	AC	A	0.223	22.3 kb U *LOC786669*	5.89E-42	1.1 ± 0.08
ARS-BFGL-NGS-79649	7	8401856	8573943	AC	A	0.089	*LOC513779*	8.76E-42	1.6 ± 0.11
ARS-BFGL-BAC-14883	1	14102864	14360342	AG	G	0.164	1 Mb blank	7.09E-41	1.2 ± 0.09
ARS-BFGL-NGS-58887	3	91877739	97854124	AG	G	0.214	17.9 kb U *BSND*	1.56E-40	1.1 ± 0.08

SNP, single nucleotide polymorphism; PTA, predicted transmitting ability; Chr, chromosome; UMD, University of Maryland; Btau_4.0, Baylor College of Medicine bovine genome assembly Build 4.0; A, SNP alleles; FA, favorable allele; FAF, FA frequency; U, upstream from; D, downstream from; SE, standard error.

Table S2.7: Top 20 most significant SNP effects for PTA for somatic cell score

SNP	Chr	UMD position	Btau_4.0 position	A	FA	FAF	Gene region	*P* value	Effect ± SE
ARS-BFGL-NGS-4774	7	17403976	14635617	AC	A	0.323	1.5 kb D *INSR* (*Q95M43*)	7.09E-44	0.08 ± 0.006
ARS-BFGL-NGS-77438	2	27814925	28778269	AG	A	0.253	*MIR2353–STK39*	5.82E-42	0.09 ± 0.006
BTB-00470332	11	27519615	28758517	AG	G	0.193	164.9 kb U *LOC100138228*	1.87E-40	0.09 ± 0.007
ARS-BFGL-NGS-18028	X	106241123	63961868	CG	C	0.131	30.1 kb U *LOC520057*	1.61E-39	0.11 ± 0.008
Hapmap29674-BTA-160310	2	54968718	57105486	AT	T	0.244	*LRP1B*	6.92E-37	0.08 ± 0.006
ARS-BFGL-NGS-103364	20	67606012	36687	AC	A	0.831	223.8 kb U *RANBP17*	1.33E-35	0.09 ± 0.007
ARS-BFGL-NGS-15281	16	21741980	20041296	AG	A	0.395	41.5 kb U *MGC152575*	2.36E-35	0.07 ± 0.006
ARS-BFGL-NGS-79649	7	8401856	8573943	AC	A	0.089	*LOC513779*	2.97E-34	0.12 ± 0.009
ARS-BFGL-NGS-105321	2	56040478	58227704	AT	T	0.232	*LRP1B*	9.03E-34	0.08 ± 0.006
BTB-01654826	6	88891318	90075263	AG	G	0.406	153.4 kb D *GC*	1.11E-33	0.07 ± 0.006
Hapmap25382-BTC-000577	25	5273751	5983281	AG	A	0.544	1 Mb blank	1.21E-33	0.07 ± 0.006
Hapmap42724-BTA-91683	X	12556511	5283247	AG	A	0.453	1 Mb blank	2.45E-33	0.07 ± 0.006
BTA-94079-no-rs	X	106280810	64001706	AG	G	0.111	8.1 kb D *LOC520057*	3.69E-32	0.11 ± 0.009
BTB-00495251	12	NA	52419056	AG	G	0.312	24.5 kb U *KCTD12*	4.91E-32	0.07 ± 0.006
ARS-BFGL-NGS-17376	6	88822266	90008099	AG	G	0.307	86.3 kb D *GC*	6.34E-32	0.07 ± 0.006
Hapmap55237-rs29010308	5	15083929	17272101	AG	G	0.712	70.3 kb D *ALX1*	1.91E-31	0.07 ± 0.006
ARS-BFGL-NGS-103952	13	5239814	5366934	AG	A	0.037	101.3 kb U *BTBD3*	2.47E-31	0.18 ± 0.015
BFGL-NGS-119848	25	10131703	11128818	AG	G	0.452	*LOC528008*	4.06E-31	0.07 ± 0.006
Hapmap60224-rs29001782	6	85178107	86128027	AG	G	0.521	*LOC100140490*	5.12E-31	0.07 ± 0.006
Hapmap50931-BTA-31235	7	31136178	28528437	AG	A	0.793	37.1 kb U *LOC520487*	6.06E-31	0.08 ± 0.007

SNP, single nucleotide polymorphism; PTA, predicted transmitting ability; Chr, chromosome; UMD, University of Maryland; Btau_4.0, Baylor College of Medicine bovine genome assembly Build 4.0; A, SNP alleles; FA, favorable allele; FAF, FA frequency; U, upstream from; D, downstream from; SE, standard error.

Table S2.8: Top 20 most significant SNP effects for PTA for daughter pregnancy rate

SNP	Chr	UMD position	Btau_4.0 position	A	FA	FAF	Gene region	*P* value	Effect ± SE (%)
ARS-BFGL-NGS-4774	7	17403976	14635617	AC	A	0.323	1.5Kb D *INSR* (*Q95M43*)	1.07E-48	0.7 ± 0.05
ARS-BFGL-NGS-43785	X	3167117	277676	AG	A	0.245	80Kb U *ATP1B4*	2.72E-48	0.8 ± 0.05
ARS-BFGL-NGS-68638	X	2720744	790040	AC	A	0.367	187.3Kb D *C1GALT1C1*	1.42E-44	0.7 ± 0.05
ARS-BFGL-NGS-18028	X	106241123	63961868	CG	C	0.131	30.1Kb U *LOC520057*	5.30E-43	0.9 ± 0.06
ARS-BFGL-NGS-79649	7	8401856	8573943	AC	A	0.089	*LOC513779*	1.65E-42	1.1 ± 0.08
BTA-29287-no-rs	18	58696066	58353743	AG	A	0.114	*LOC787057*	1.62E-40	0.9 ± 0.07
ARS-BFGL-NGS-94205	X	7200715	2348904	AG	A	0.464	*GRIA3*	1.66E-40	0.6 ± 0.04
BFGL-NGS-112671	7	12889689	9992685	AG	A	0.297	*CC2D1A*	6.63E-40	0.7 ± 0.05
BTA-31244-no-rs	X	NA	15607221	AC	A	0.362	144.3Kb U *SLITRK4*	1.19E-39	0.7 ± 0.05
ARS-BFGL-NGS-24528	1	129101492	130311120	AC	A	0.319	59.9Kb D *SLC25A36*	3.21E-39	0.6 ± 0.05
BTB-00067270	1	141351312	142238760	AG	A	0.224	*LOC511594*	4.85E-39	0.7 ± 0.05
ARS-BFGL-NGS-36483	X	25731651	14983892	AG	A	0.271	342Kb D *LOC531026*	6.17E-39	0.6 ± 0.05
ARS-BFGL-NGS-95575	22	40726456	41108975	AG	A	0.295	*FHIT*	6.55E-38	0.6 ± 0.05
BFGL-NGS-112365	18	64548360	64574451	AG	A	0.227	*LOC100137761*	1.20E-36	0.7 ± 0.05
BTA-117207-no-rs	17	47268519	48762074	AG	A	0.350	*LOC615031*	1.20E-36	0.6 ± 0.05
BTA-94079-no-rs	X	106280810	64001706	AG	G	0.111	8.1Kb D *LOC520057*	1.94E-36	0.9 ± 0.07
ARS-BFGL-NGS-73455	1	140524195	141310420	AG	G	0.279	1.6Kb D *ATP2C1*	7.32E-36	0.6 ± 0.05
BFGL-NGS-113451	1	135812920	137063132	AG	G	0.359	31.3Kb U *LOC781026*	1.55E-35	0.6 ± 0.05
ARS-BFGL-NGS-57673	8	84474299	87229588	AG	G	0.245	*SLC35D2*	2.93E-35	0.6 ± 0.05
BTB-00142159	3	89942617	95776746	AG	A	0.347	16.5Kb U *LOC615569*	4.10E-35	0.6 ± 0.05

SNP, single nucleotide polymorphism; PTA, predicted transmitting ability; Chr, chromosome; UMD, University of Maryland; Btau_4.0, Baylor College of Medicine bovine genome assembly Build 4.0; A, SNP alleles; FA, favorable allele; FAF, FA frequency; U, upstream from; D, downstream from; SE, standard error.

Table S2.9: Top 20 most significant SNP effects for PTA for service-sire calving ease

SNP	Chr	UMD position	Btau_4.0 position	A	FA	FAF	Gene region	*P* value	Effect ± SE (%DBH)
BFGL-NGS-117985	18	53948569	53311437	AG	G	0.091	409 bases D *PGLYRP1*; 16 kb D *IGFL1*	1.64E-98	1.4 ± 0.06
BTB-01333903	1	28362687	29095948	AG	G	0.067	220.9 kb U *FKBP2*	7.35E-97	1.6 ± 0.07
Hapmap50642-BTA-28388	26	49137602	49579691	AG	G	0.089	80.8 kb U *MGMT*	4.68E-87	1.3 ± 0.06
BTA-29287-no-rs	18	58696066	58353743	AG	A	0.114	*LOC787057*	8.61E-86	1.2 ± 0.06
ARS-BFGL-NGS-1096	X	12604306	5329598	AG	A	0.074	1 Mb blank	1.07E-81	1.4 ± 0.07
ARS-BFGL-NGS-16708	17	15107947	15883911	AC	A	0.127	187.4 kb D *USP38*	4.96E-81	1.1 ± 0.05
BFGL-NGS-111445	26	48120342	48539159	AG	A	0.089	1 Mb blank	4.25E-80	1.3 ± 0.06
ARS-BFGL-NGS-13673	1	87402236	88871762	AC	A	0.090	428.3 kb D *TTC14*	1.07E-75	1.2 ± 0.06
ARS-BFGL-BAC-34040	17	18764770	19749801	AC	C	0.108	2.9 kb D *LOC528054*	2.13E-71	1.1 ± 0.06
BTA-03029-rs29010796	18	6752549	5696451	AC	A	0.124	200.5 kb D *MAF*	1.01E-70	1.0 ± 0.06
ARS-BFGL-NGS-58480	X	61129008	34617065	AG	G	0.129	*COL4A6*	5.03E-67	1.0 ± 0.06
ARS-BFGL-NGS-18028	X	106241123	63961868	CG	C	0.131	30.1 kb U *LOC520057*	7.32E-67	1.0 ± 0.06
BTB-01308199	17	33827641	35340688	AG	A	0.121	351 kb D *ANKRD50*	3.01E-66	1.0 ± 0.06
ARS-BFGL-NGS-1312	26	48583446	48998305	AG	A	0.107	1 Mb blank	3.95E-66	1.1 ± 0.06
ARS-BFGL-BAC-29490	23	20166517	20900919	AG	A	0.112	*GPR110–GPR116*	5.59E-66	1.1 ± 0.06
BTB-00495300	12	52240216	52333042	AG	A	0.124	110.5 kb U *KCTD12*	1.17E-65	1.0 ± 0.06
ARS-BFGL-BAC-32721	17	34377274	35876692	AG	G	0.129	372.2 kb U *SPY1*	3.50E-61	1.0 ± 0.06
ARS-BFGL-NGS-25718	17	28254430	29708488	AG	A	0.113	89.3 kb D *LOC784783*	3.97E-61	1.0 ± 0.06
BTB-01297598	17	14080531	14889608	AG	A	0.151	318.7 kb U *FREM3*	6.00E-61	0.9 ± 0.05
Hapmap51817-BTA-22015	26	2915575	2430289	AG	A	0.091	61.7 kb D *ZWINT*	8.16E-61	1.1 ± 0.06

SNP, single nucleotide polymorphism; PTA, predicted transmitting ability; Chr, chromosome; UMD, University of Maryland; Btau_4.0, Baylor College of Medicine bovine genome assembly Build 4.0; A, SNP alleles; FA, favorable allele; FAF, FA frequency; %DBH, percentage of births of bull calves that are difficult in primiparous heifers; U, upstream from; D, downstream from; SE, standard error.

Table S2.10: Top 20 most significant SNP effects for PTA for daughter calving ease

SNP	Chr	UMD position	Btau_4.0 position	A	FA	FAF	Gene region	*P* value	Effect ± SE (%DBH)
BFGL-NGS-117985	18	53948569	53311437	AG	G	0.091	409 bases D *PGLYRP1*; 16 kb D *IGFL1*	1.48E-52	0.9 ± 0.06
BTA-29287-no-rs	2	58696066	58353743	AG	A	0.114	*LOC787057*	8.00E-52	0.8 ± 0.05
Hapmap50642-BTA-28388	26	49137602	49579691	AG	G	0.089	80.8 kb U *MGMT*	5.27E-51	0.9 ± 0.06
BFGL-NGS-111445	26	48120342	48539159	AG	A	0.089	1 Mb blank	2.53E-46	0.9 ± 0.06
ARS-BFGL-NGS-16708	17	15107947	15883911	AC	A	0.127	187.4 kb D *USP38*	2.48E-45	0.7 ± 0.05
ARS-BFGL-BAC-32721	17	34377274	35876692	AG	G	0.129	372.2 kb U *SPY1*	4.96E-44	0.7 ± 0.05
BTB-01308199	17	33827641	35340688	AG	A	0.121	351Kb D *ANKRD50*	8.64E-44	0.7 ± 0.05
ARS-BFGL-BAC-31505	17	12218477	12905532	AG	A	0.159	*LOC614109*	2.31E-41	0.7 ± 0.05
BTB-01333903	1	28362687	29095948	AG	G	0.067	220.9 kb U *FKBP2*	7.29E-40	0.9 ± 0.07
ARS-BFGL-NGS-1096	X	12604306	5329598	AG	A	0.074	1 Mb blank	5.99E-39	0.9 ± 0.07
ARS-BFGL-NGS-20640	17	12258111	12944672	AC	A	0.138	B *LSM6*, *LOC614109*	4.41E-38	0.7 ± 0.05
ARS-BFGL-BAC-34040	17	18764770	19749801	AC	C	0.108	2.9 kb D *LOC528054*	1.30E-37	0.7 ± 0.06
BFGL-NGS-112365	18	64548360	64574451	AG	A	0.227	*LOC100137761*	4.18E-37	0.5 ± 0.04
Hapmap39897-BTA-18318	17	14357124	15155735	AC	C	0.192	*LOC519752*	4.05E-36	0.6 ± 0.04
BFGL-NGS-112636	17	32679701	34136935	AC	A	0.156	33.1 kb U *FAT4*	5.98E-36	0.6 ± 0.05
BTB-00671736	17	12288221	12974438	AG	A	0.177	34.7 kb U *LSM6*	1.48E-35	0.6 ± 0.05
ARS-BFGL-NGS-18028	X	106241123	63961868	CG	C	0.131	30.1 kb U *LOC520057*	1.99E-35	0.7 ± 0.05
ARS-BFGL-NGS-56673	28	27229817	26332799	AG	A	0.226	*PCBD1*	3.54E-35	0.5 ± 0.04
ARS-BFGL-NGS-1312	26	48583446	48998305	AG	A	0.107	1 Mb blank	7.52E-35	0.7 ± 0.06
ARS-BFGL-NGS-25718	17	28254430	29708488	AG	A	0.113	89.3 kb D *LOC784783*	2.04E-34	0.7 ± 0.05

SNP, single nucleotide polymorphism; PTA, predicted transmitting ability; Chr, chromosome; Btau_4.0, Baylor College of Medicine bovine genome assembly Build 4.0; UMD, University of Maryland; A, SNP alleles; FA, favorable allele; FAF, FA frequency; %DBH, percentage of births of bull calves that are difficult in primiparous heifers; U, upstream from; D, downstream from; SE, standard error.

Table S2.11: Top 20 most significant SNP effects for PTA for service-sire stillbirth

SNP	Chr	UMD position	Btau_4.0 position	A	FA	FAF	Gene region	*P* value	Effect ± SE (%SB)
BTA-29287-no-rs	18	58696066	58353743	AG	A	0.114	*LOC787057*	1.19E-51	0.51 ± 0.032
BTB-01333903	1	28362687	29095948	AG	G	0.067	220.9 kb U *FKBP2*	1.14E-47	0.62 ± 0.042
Hapmap50642-BTA-28388	26	49137602	49579691	AG	G	0.089	80.8 kb U *MGMT*	1.63E-46	0.54 ± 0.036
Hapmap28373-BTA-160078	X	24528596	14063338	CG	G	0.216	110.4 kb D *LOC781178*	4.19E-46	0.38 ± 0.026
ARS-BFGL-NGS-1096	X	12604306	5329598	AG	A	0.074	1 Mb blank	5.74E-46	0.60 ± 0.041
ARS-BFGL-NGS-61325	X	24505176	14038654	AC	C	0.216	85.7 kb D *LOC781178*	7.43E-46	0.38 ± 0.026
ARS-BFGL-NGS-58480	X	61129008	34617065	AG	G	0.129	*COL4A6*	7.84E-46	0.47 ± 0.032
BFGL-NGS-117985	18	53948569	53311437	AG	G	0.091	409 bases D *PGLYRP1*; 16 kb D *IGFL1*	2.53E-44	0.52 ± 0.036
BFGL-NGS-118037	3	109036828	115646036	AG	G	0.733	37.4 kb D *ZC12A*	8.70E-42	0.34 ± 0.024
BFGL-NGS-111445	26	48120342	48539159	AG	A	0.089	1 Mb blank	1.50E-41	0.51 ± 0.037
BTA-31244-no-rs	X	NA	15607221	AC	A	0.362	144.3 kb U *SLITRK4*	5.06E-41	0.33 ± 0.024
BFGL-NGS-118669	X	105267785	62950780	AG	G	0.109	*MAOB*	6.14E-40	0.46 ± 0.034
ARS-BFGL-NGS-13189	21	56088858	56005928	AG	G	0.220	*FRMD5*	6.13E-39	0.34 ± 0.025
BFGL-NGS-111739	26	18684200	20476861	AG	A	0.263	*PI4K2A*	9.79E-39	0.32 ± 0.024
BTB-00154898	3	107830416	114293020	AG	A	0.190	105.7 kb D *RRAGC*	6.48E-38	0.36 ± 0.027
ARS-BFGL-NGS-18028	X	106241123	63961868	CG	C	0.131	30.1 kb U *LOC520057*	7.21E-38	0.42 ± 0.032
Hapmap40460-BTA-69362	3	107779560	114242949	AG	A	0.189	55.6 kb D *RRAGC*	1.26E-37	0.36 ± 0.028
Hapmap51142-BTA-109780	1	117854060	118496250	AG	G	0.859	18.1 kb D *GP171*	1.12E-36	0.40 ± 0.031
ARS-BFGL-NGS-77389	14	38344250	2357161	AG	A	0.919	15.9 kb U *EIF2C2*	1.48E-36	0.51 ± 0.040
BTB-00332985	14	54779831	50317494	AG	G	0.639	1 Mb blank	9.08E-36	0.28 ± 0.022

SNP, single nucleotide polymorphism; PTA, predicted transmitting ability; Chr, chromosome; UMD, University of Maryland; Btau_4.0, Baylor College of Medicine bovine genome assembly Build 4.0; A, SNP alleles; FA, favorable allele; FAF, FA frequency; %SB, percentage of births of bulls calves that are stillborn in primiparous heifers; U, upstream from; D, downstream from; SE, standard error.

Table S2.12: Top 20 most significant SNP effects for PTA for daughter stillbirth

SNP	Chr	UMD position	Btau_4.0 position	A	FA	FAF	Gene region	*P* value	Effect ± SE (%SB)
ARS-BFGL-NGS-26950	15	75749702	74999563	AG	G	0.848	23.9 kb U 2nd *CD82*	1.13E-38	0.67 ± 0.050
Hapmap58371-rs29014419	23	3320932	3162892	AG	A	0.476	*DST*	2.39E-34	0.45 ± 0.036
ARS-BFGL-NGS-101393	23	14063300	14634231	AG	G	0.472	202.5 kb D *MOCS1*	8.34E-34	0.46 ± 0.038
Hapmap34246-BES10_Contig295_764	27	20234255	23118868	AG	G	0.918	102 bases D *LOC781916*	6.86E-32	0.80 ± 0.067
ARS-BFGL-NGS-61941	23	14101481	14672412	AG	A	0.663	207 kb U *LRFN2*	7.78E-32	0.47 ± 0.039
ARS-BFGL-NGS-105601	8	90007976	93193613	AG	A	0.098	9.5 kb D *GA45G*	1.31E-31	0.73 ± 0.061
ARS-BFGL-NGS-105087	23	14123921	14694856	AG	G	0.663	184.5 kb U *LRFN2*	1.67E-31	0.47 ± 0.039
BFGL-NGS-111105	X	21644976	11536827	AC	C	0.137	125.7 kb U *LOC788379*	1.48E-30	0.63 ± 0.053
ARS-BFGL-NGS-54803	11	100283991	103971758	AG	G	0.721	B*USP20*; *FNBP1*	2.12E-30	0.47 ± 0.040
BFGL-NGS-114029	13	73653480	73694769	AG	A	0.714	*SERINC3*	2.79E-30	0.48 ± 0.041
ARS-BFGL-NGS-25089	23	NA	14548348	AC	A	0.608	116.6 kb D *MOCS1*	5.60E-30	0.44 ± 0.038
Hapmap48368-BTA-50297	1	126846004	127923588	AG	A	0.911	26.9 kb U *CHST2*	6.27E-30	0.74 ± 0.064
ARS-BFGL-NGS-21804	20	59016802	62511140	AG	A	0.464	237.1 kb D *TRIO*	7.60E-29	0.43 ± 0.038
BTA-104538-no-rs	X	52781880	33062305	AG	A	0.354	*IL1RAPL2*	1.18E-28	0.43 ± 0.038
Hapmap49212-BTA-53105	21	69340662	67838714	AG	A	0.216	*LOC508226*	1.34E-28	0.50 ± 0.044
ARS-BFGL-NGS-37630	11	46590323	48427054	AG	A	0.882	16.6 kb D *IL1F6*	1.56E-28	0.64 ± 0.057
ARS-BFGL-NGS-64059	20	59070717	62564938	AG	A	0.542	290.9 kb D *TRIO*	4.39E-28	0.41 ± 0.037
ARS-BFGL-NGS-103952	13	5239814	5366934	AG	A	0.037	101.3 kb U *BTBD3*	4.43E-28	1.11 ± 0.100
ARS-BFGL-NGS-38254	15	76350223	75681727	AG	G	0.805	145.7 kb D *SYT13*	6.02E-28	0.53 ± 0.048
Hapmap43471-BTA-114689	13	73369210	73430176	AG	G	0.576	*JPH2*	1.02E-27	0.42 ± 0.037

SNP, single nucleotide polymorphism; PTA, predicted transmitting ability; Chr, chromosome; UMD, University of Maryland; Btau_4.0, Baylor College of Medicine bovine genome assembly Build 4.0; A, SNP alleles; FA, favorable allele; FAF, FA frequency; %SB, percentage of births of bulls calves that are stillborn in primiparous heifers; U, upstream from; D, downstream from; SE, standard error.

Table S2.13: Top 20 most significant SNP effects for lifetime net merit, a genetic-economic index

SNP	Chr	UMD position	Btau_4.0 position	A	FA	FAF	Gene region	*P* value	Effect ± SE ($)
BFGL-NGS-117985	18	53948569	53311437	AG	G	0.091	409 bases D *PGLYRP1*; 16 kb D *IGFL1*	4.03E-88	199 ± 9.4
BTB-01333903	1	28362687	29095948	AG	G	0.067	220.9 kb U *FKBP2*	5.82E-84	223 ± 10.8
ARS-BFGL-NGS-1096	X	12604306	5329598	AG	A	0.074	1 Mb blank	3.19E-79	214 ± 10.7
BTA-29287-no-rs	18	58696066	58353743	AG	A	0.114	*LOC787057*	6.44E-77	169 ± 8.6
ARS-BFGL-NGS-13673	1	87402236	88871762	AC	A	0.090	428.3 kb D *TTC14*	5.41E-75	184 ± 9.5
Hapmap50642-BTA-28388	26	49137602	49579691	AG	G	0.089	80.8 kb U *MGMT*	2.89E-74	184 ± 9.6
BFGL-NGS-111445	26	48120342	48539159	AG	A	0.089	1 Mb blank	8.93E-71	181 ± 9.7
ARS-BFGL-NGS-18028	X	106241123	63961868	CG	C	0.131	30.1 kb U *LOC520057*	3.8E-62	147 ± 8.5
BTA-110943-no-rs	27	29524324	31986530	AG	A	0.118	1 Mb blank	8.87E-62	152 ± 8.8
ARS-BFGL-NGS-58480	X	61129008	34617065	AG	G	0.129	*COL4A6*	6.91E-60	146 ± 8.6
Hapmap51817-BTA-22015	26	2915575	2430289	AG	A	0.091	61.7 kb D *ZWINT*	1.52E-57	163 ± 9.8
BTA-16909-no-rs	X	136196550	336258	AC	C	0.124	*GLRA2*	2.63E-57	141 ± 8.5
BTA-32998-no-rs	13	58043371	58276512	CG	G	0.210	81.2 kb D *GNAS*	2.87E-55	115 ± 7.1
ARS-BFGL-NGS-4463	18	49167271	33210	AG	G	0.169	255.3 kb U *UQCRFS1*	8.6E-55	123 ± 7.6
ARS-BFGL-BAC-34040	17	18764770	19749801	AC	C	0.108	2.9 kb D *LOC528054*	2.75E-54	148 ± 9.2
ARS-BFGL-NGS-16708	17	15107947	15883911	AC	A	0.127	187.4 kb D *USP38*	5.56E-54	138 ± 8.6
ARS-BFGL-NGS-4840	X	136328915	468626	AG	G	0.128	18.1 kb D *LAMP2*	5.2E-53	136 ± 8.6
BTA-94079-no-rs	X	106280810	64001706	AG	G	0.111	8.1 kb D *LOC520057*	5.79E-53	150 ± 9.4
BTA-33000-no-rs	13	58099969	58309152	AG	A	0.215	113.9 kb D *GNAS*	9.48E-53	112 ± 7.1
ARS-BFGL-NGS-1312	26	48583446	48998305	AG	A	0.107	1 Mb blank	1.09E-52	146 ± 9.2

SNP, single nucleotide polymorphism; PTA, predicted transmitting ability; Chr, chromosome; UMD, University of Maryland; Btau_4.0, Baylor College of Medicine bovine genome assembly Build 4.0; A, SNP alleles; FA, favorable allele; FAF, FA frequency; U, upstream from; D, downstream from; SE, standard error.

Table S2.14: Top 20 most significant SNP effects for PTA for stature

SNP	Chr	UMD position	Btau_4.0 position	A	FA	FAF	Gene region	*P* value	Effect ± SE
Hapmap46795-BTA-30632	X	131766182	77183469	AG	G	0.422	*PHKA2*	9.18E-47	0.50 ± 0.034
ARS-BFGL-NGS-14236	11	86048363	88746533	AG	G	0.329	2.7 kb U *LPIN1*	9.12E-38	0.46 ± 0.035
ARS-BFGL-NGS-2015	11	84872349	87604666	AG	A	0.255	323 kb D *TRIB2*	1.99E-37	0.49 ± 0.038
Hapmap52066-rs29015690	11	85153576	87885711	AC	A	0.419	51.9 kb U *TRIB2*	1.94E-34	0.41 ± 0.033
BTA-99263-no-rs	11	79493646	81752983	AC	C	0.262	*OSR1*	2.49E-34	0.47 ± 0.038
BTA-30787-no-rs	X	145062801	1163798	AG	G	0.377	81.8 kb U *LOC100140451*	7.68E-34	0.42 ± 0.034
BFGL-NGS-114168	11	100108508	103767146	AG	A	0.265	*PRRX2*	1.76E-33	0.46 ± 0.038
ARS-BFGL-NGS-34903	11	83867859	86478143	AG	G	0.486	199.1 kb U *COPS2*	1.46E-32	0.41 ± 0.034
ARS-BFGL-NGS-25464	11	42520603	44309151	AG	A	0.464	1 Mb blank	1.40E-31	0.40 ± 0.034
Hapmap44214-BTA-15727	X	136832710	80955090	AG	A	0.433	*GEMIN8–GPM6B*	2.28E-31	0.41 ± 0.034
Hapmap38268-BTA-09661	X	142828641	85510888	AC	A	0.381	1 kb D *CLCN4*	7.65E-31	0.41 ± 0.035
ARS-BFGL-NGS-11105	11	78510118	80795967	AG	A	0.239	*LOC529399*	1.29E-30	0.46 ± 0.039
ARS-BFGL-NGS-99064	11	44813737	85547735	AG	G	0.298	*NBAS*	1.31E-30	0.43 ± 0.036
BTA-74498-no-rs	5	85980094	91980041	AG	A	0.165	381 kb D *BCAT1*	2.26E-30	0.53 ± 0.045
Hapmap22874-BTA-153755	11	83168133	85760282	AC	C	0.231	*NBAS*	2.48E-30	0.45 ± 0.039
ARS-BFGL-NGS-22907	X	131710317	77127986	AG	G	0.548	13.9 kb U *PHKA2*	2.82E-30	0.39 ± 0.034
ARS-BFGL-NGS-104897	1	112863292	114280346	AC	A	0.213	*PLCH1*	8.78E-30	0.46 ± 0.039
ARS-BFGL-NGS-3506	5	70897603	75705849	AG	G	0.374	25.9 kb U *BTBD11*	1.05E-29	0.40 ± 0.035
ARS-BFGL-NGS-12185	X	145243690	983791	AC	C	0.121	261.8 kb U *LOC100140451*	1.22E-29	0.59 ± 0.051
Hapmap39471-BTA-15720	X	136904787	81027958	AC	C	0.365	*GPM6B*	4.67E-29	0.40 ± 0.035

SNP, single nucleotide polymorphism; PTA, predicted transmitting ability; Chr, chromosome; UMD, University of Maryland; Btau_4.0, Baylor College of Medicine bovine genome assembly Build 4.0; A, SNP alleles; FA, favorable allele; FAF, FA frequency; U, upstream from; D, downstream from; SE, standard error.

Table S2.15: Top 20 most significant SNP effects for PTA for strength

SNP	Chr	UMD position	Btau_4.0 position	A	FA	FAF	Gene region	*P* value	Effect ± SE
Hapmap46795-BTA-30632	X	131766182	77183469	AG	G	0.422	*PHKA2*	8.46E-38	0.40 ± 0.030
ARS-BFGL-NGS-83607	16	1756016	937280	AG	A	0.586	*REN*	1.58E-35	0.38 ± 0.030
BTA-65964-no-rs	23	18197600	18862624	AG	G	0.899	25.6 kb U *SUPT3H*	5.00E-33	0.58 ± 0.048
Hapmap28514-BTA-163525	26	49185154	49627246	CG	G	0.149	*MGMT*	2.34E-32	0.49 ± 0.040
BTA-74498-no-rs	5	85980094	91980041	AG	A	0.165	381 kb D *BCAT1*	1.05E-31	0.48 ± 0.040
ARS-BFGL-NGS-17511	X	31394601	18918508	AG	A	0.385	28.894 kb U *AFF2*	4.97E-31	0.35 ± 0.030
BTA-99048-no-rs	13	45475151	45247268	AG	A	0.828	*PITRM1*	1.30E-30	0.46 ± 0.039
ARS-BFGL-NGS-35882	X	137586078	83553143	AG	G	0.237	*2.8 kb U ARHGAP6*	1.43E-30	0.40 ± 0.034
ARS-BFGL-NGS-96376	16	1854268	1035558	AG	G	0.603	*PLEKHA6*	1.89E-30	0.35 ± 0.030
Hapmap39471-BTA-15720	X	136904787	81027958	AC	C	0.365	*GPM6*	2.24E-30	0.36 ± 0.031
BTA-30787-no-rs	X	145062801	1163798	AG	G	0.377	81.8 kb U *LOC10014045*1	2.78E-30	0.35 ± 0.030
Hapmap38268-BTA-09661	X	142828641	85510888	AC	A	0.381	1 kb D *CLCN4*	4.91E-30	0.36 ± 0.031
ARS-BFGL-NGS-107544	X	135879672	23457	AC	C	0.312	334.2 kb U *ATP1B4*	5.87E-30	0.36 ± 0.031
ARS-BFGL-NGS-73521	13	45234264	45023457	AG	A	0.900	222.6 kb U *PITRM1*	1.05E-29	0.55 ± 0.048
ARS-BFGL-NGS-32517	26	47907512	48311186	AG	G	0.315	28.2 kb D *MKI67*	2.80E-29	0.37 ± 0.032
ARS-BFGL-NGS-61317	26	39288059	39335884	AG	A	0.229	4.5 kb U *C10ORF46*	8.14E-29	0.39 ± 0.035
BTA-32998-no-rs	13	58043371	58276512	CG	G	0.210	*GNAS–LOC100141087*	3.39E-28	0.40 ± 0.036
ARS-BFGL-NGS-14592	13	56642417	56803006	AG	G	0.334	250.1 kb U *LOC782266*	6.01E-28	0.36 ± 0.032
BFGL-NGS-110788	6	16575847	16827786	AG	G	0.341	*EGF*	1.29E-27	0.35 ± 0.031
ARS-BFGL-NGS-107943	X	136790063	80912444	AG	G	0.532	*GEMIN8–GPM6B*	1.78E-27	0.33 ± 0.030

SNP, single nucleotide polymorphism; PTA, predicted transmitting ability; Chr, chromosome; UMD, University of Maryland; Btau_4.0, Baylor College of Medicine bovine genome assembly Build 4.0; A, SNP alleles; FA, favorable allele; FAF, FA frequency; U, upstream from; D, downstream from; SE, standard error.

Table S2.16: Top 20 most significant SNP effects for PTA for body depth

SNP	Chr	UMD position	Btau_4.0 position	A	FA	FAF	Gene region	*P* value	Effect ± SE
Hapmap46795-BTA-30632	X	131766182	77183469	AG	G	0.422	*PHKA2*	7.99E-38	0.40 ± 0.030
BTA-65964-no-rs	23	18197600	18862624	AG	G	0.899	25.6 kb U *SUPT3H*	1.65E-34	0.60 ± 0.048
ARS-BFGL-NGS-34903	11	83867859	86478143	AG	G	0.486	199.1 kb U *COPS2*	6.31E-31	0.35 ± 0.030
ARS-BFGL-NGS-83607	16	1756016	937280	AG	A	0.586	*REN*	6.41E-31	0.36 ± 0.030
ARS-BFGL-NGS-73521	13	45234264	45023457	AG	A	0.900	222.6 kb U *PITRM1*	7.11E-30	0.55 ± 0.048
BTA-99048-no-rs	13	45475151	45247268	AG	A	0.828	*PITRM1*	1.21E-29	0.46 ± 0.040
ARS-BFGL-NGS-2015	11	84872349	87604666	AG	A	0.255	323 kb D *TRIB2*	1.28E-28	0.38 ± 0.034
BTA-74498-no-rs	5	85980094	91980041	AG	A	0.165	381 kb D *BCAT1*	1.47E-28	0.45 ± 0.040
ARS-BFGL-NGS-14236	11	86048363	88746533	AG	G	0.329	2.7 kb U *LPIN1*	2.17E-28	0.35 ± 0.031
ARS-BFGL-NGS-19442	5	70997483	75805133	AG	A	0.383	*BTBD11*	5.35E-28	0.34 ± 0.031
ARS-BFGL-NGS-3506	5	70897603	75705849	AG	G	0.374	25.9 kb U *BTBD11*	4.31E-27	0.34 ± 0.031
ARS-BFGL-NGS-52709	11	81819453	83850462	AG	A	0.641	158.2 kb U *FAM49A*	4.95E-27	0.36 ± 0.030
ARS-BFGL-NGS-19628	13	45404283	45220258	AC	A	0.817	25.8 kb U *PITRM1*	6.05E-27	0.43 ± 0.039
ARS-BFGL-NGS-99064	11	44813737	85547735	AG	G	0.298	*NBAS*	7.15E-26	0.35 ± 0.032
Hapmap39471-BTA-15720	X	136904787	81027958	AC	C	0.365	*GPM6B*	7.33E-26	0.33 ± 0.031
BTA-74541-no-rs	5	90255244	96336628	AG	A	0.629	394 kb D *PDE3A*	8.94E-26	0.33 ± 0.031
Hapmap52066-rs29015690	11	85153576	87885711	AC	A	0.419	51.9 kb U *TRIB2*	9.51E-26	0.31 ± 0.030
Hapmap22874-BTA-153755	11	83168133	85760282	AC	C	0.231	*NBAS*	9.58E-26	0.37 ± 0.034
Hapmap43893-BTA-60736	25	9862811	10844958	AG	G	0.746	*CLEC16A*	1.18E-25	0.36 ± 0.034
Hapmap44214-BTA-15727	X	136832710	80955090	AG	A	0.433	*GEMIN8–GPM6B*	1.37E-25	0.33 ± 0.030

SNP, single nucleotide polymorphism; PTA, predicted transmitting ability; Chr, chromosome; UMD, University of Maryland; Btau_4.0, Baylor College of Medicine bovine genome assembly Build 4.0; A, SNP alleles; FA, favorable allele; FAF, FA frequency; U, upstream from; D, downstream from; SE, standard error.

Table S2.17: Top 20 most significant SNP effects for PTA for rump width

SNP	Chr	UMD position	Btau_4.0 position	A	FA	FAF	Gene region	*P* value	Effect ± SE
ARS-BFGL-NGS-83607	16	1756016	937280	AG	A	0.586	*REN*	2.91E-40	0.42 ± 0.031
Hapmap46795-BTA-30632	X	131766182	77183469	AG	G	0.422	*PHKA2*	3.39E-40	0.42 ± 0.031
ARS-BFGL-NGS-27636	19	57655339	58732295	AG	A	0.176	*GPRC5C*	3.22E-32	0.48 ± 0.040
ARS-BFGL-NGS-12185	X	145243690	983791	AC	C	0.121	261.8 kb U *LOC100140451*	3.59E-32	0.56 ± 0.046
BTA-08388-no-rs	19	56132884	57220725	AC	C	0.685	*RNF157*	4.36E-32	0.39 ± 0.033
BTA-65964-no-rs	23	18197600	18862624	AG	G	0.899	25.6 kb U *SUPT3H*	8.73E-32	0.60 ± 0.050
ARS-BFGL-NGS-27758	19	57876833	58971180	AG	A	0.186	36.1 kb D *RPL38*	8.20E-31	0.46 ± 0.039
ARS-BFGL-NGS-52939	19	56244442	57331979	AG	A	0.172	*SRP68*	3.96E-29	0.46 ± 0.040
Hapmap41275-BTA-47175	10	64905140	69491	AG	A	0.511	*APC*	5.70E-29	0.35 ± 0.031
ARS-BFGL-NGS-35882	X	137586078	83553143	AG	G	0.237	*2.8 KB U ARHGAP6*	7.11E-28	0.40 ± 0.036
ARS-BFGL-BAC-33563	19	57017362	58011913	AG	A	0.192	*LOC789539*	7.26E-28	0.43 ± 0.038
Hapmap39471-BTA-15720	X	136904787	81027958	AC	C	0.365	*GPM6B*	1.16E-27	0.35 ± 0.032
ARS-BFGL-NGS-22907	X	131710317	77127986	AG	G	0.548	13.9 kb U *PHKA2*	3.60E-27	0.34 ± 0.031
BFGL-NGS-111847	22	57579173	58486724	AG	G	0.173	*LOC782758*	6.04E-27	0.44 ± 0.040
ARS-BFGL-NGS-71489	19	57098859	58093517	AC	A	0.454	*OTOP3*	9.15E-27	0.34 ± 0.031
ARS-BFGL-NGS-105092	19	60165029	61501059	AG	A	0.432	1 Mb blank	1.28E-26	0.34 ± 0.031
ARS-BFGL-NGS-19442	5	70997483	75805133	AG	A	0.383	*BTBD11*	1.87E-26	0.34 ± 0.032
BTB-01133946	6	55796059	55956041	AG	G	0.062	1 Mb blank	2.54E-26	0.69 ± 0.063
Hapmap53237-rs29021167	10	63095461	64551774	AG	G	0.631	173.7 kb D *SEMA6D*	2.62E-26	0.34 ± 0.032
ARS-BFGL-NGS-3506	5	70897603	75705849	AG	G	0.374	25.9 kb U *BTBD11*	3.53E-26	0.34 ± 0.032

SNP, single nucleotide polymorphism; PTA, predicted transmitting ability; Chr, chromosome; UMD, University of Maryland; Btau_4.0, Baylor College of Medicine bovine genome assembly Build 4.0; A, SNP alleles; FA, favorable allele; FAF, FA frequency; U, upstream from; D, downstream from; SE, standard error.

Table S2.18: Top 20 most significant SNP effects for PTA for dairy form

SNP	Chr	UMD position	Btau_4.0 position	A	FA	FAF	Gene region	*P* value	Effect ± SE
ARS-BFGL-NGS-18028	X	106241123	63961868	CG	G	0.869	30.9 kb U *LOC520057*	3.38E-32	0.54 ± 0.045
Hapmap39109-BTA-30591	X	120691496	364178	AC	A	0.688	6.5 kb U *ATP1B4*	1.40E-30	0.38 ± 0.033
ARS-BFGL-NGS-62557	3	11458 X55	121340162	AG	G	0.572	137 kb U *ARL4C*	9.69E-30	0.36 ± 0.031
ARS-BFGL-NGS-65126	3	114526324	121275722	AC	C	0.681	201.5 kb U *ARL4C*	1.60E-29	0.37 ± 0.032
ARS-BFGL-NGS-228	7	10 3074895	102351902	AG	G	0.581	1 Mb blank	2.67E-29	0.35 ± 0.030
ARS-BFGL-NGS-104962	17	5487182	6003582	AG	G	0.704	192 kb D *FBXW7*	1.53E-28	0.38 ± 0.034
ARS-BFGL-NGS-100791	2	35004695	36035875	AG	G	0.307	58.3 kb U *TBR1*	1.82E-28	0.37 ± 0.033
ARS-BFGL-NGS-24849	17	5511241	6027682	AC	C	0.777	216.1 kb D *FBXW7*	2.01E-28	0.38 ± 0.034
Hapmap39671-BTA-62550	X	90910050	56543500	AG	A	0.657	22.3 kb U *LOC786669*	9.57E-28	0.40 ± 0.036
BFGL-NGS-111984	10	37656583	37452334	CG	C	0.38	*PLA2G4F*	1.44E-27	0.35 ± 0.032
ARS-BFGL-NGS-43868	7	91946384	90768813	AG	A	0.614	347.4 kb U *CETN3*	1.76E-27	0.35 ± 0.031
Hapmap47063-BTA-62293	10	37852123	37647411	AG	A	0.751	*CAPN3*	1.93E-27	0.34 ± 0.031
BTB-01864543	8	93404723	96575604	AG	A	0.721	68.7 kb D *LOC530667*	3.74E-27	0.38 ± 0.034
ARS-BFGL-NGS-73455	1	140524195	141310420	AG	G	0.776	1.6 kb D *ATP2C1*	4.27E-27	0.37 ± 0.034
BTB-00067270	1	141351312	142238760	AG	G	0.582	*LOC511594*	6.49E-27	0.39 ± 0.036
ARS-BFGL-NGS-84593	3	114388249	121137679	AG	A	0.889	51.3 kb D *SPP2*	7.99E-27	0.33 ± 0.030
BTA-94079-no-rs	X	106280810	64001706	AT	T	0.668	8.1 kb D *LOC520057*	1.16E-26	0.56 ± 0.051
Hapmap56111-rs29019494	7	108638689	107881812	AC	A	0.664	404.1 kb U *EFNA5*	1.44E-26	0.35 ± 0.032
Hapmap39294-BTA-80145	7	90655911	89538307	AG	G	0.729	*MEF2C*	2.71E-26	0.35 ± 0.032
ARS-BFGL-NGS-36483	X	25731651	14983892	CG	G	0.869	342 kb D *LOC531036*	2.73E-26	0.37 ± 0.034

SNP, single nucleotide polymorphism; PTA, predicted transmitting ability; Chr, chromosome; UMD, University of Maryland; Btau_4.0, Baylor College of Medicine bovine genome assembly Build 4.0; A, SNP alleles; FA, favorable allele; FAF, FA frequency; U, upstream from; D, downstream from; SE, standard error.

Table S2.19: Top 20 most significant SNP effects for PTA for rump angle

SNP	Chr	UMD position	Btau_4.0 position	A	FA	FAF	Gene region	*P* value	Effect ± SE
BTB-00227037	5	43736571	46996518	AG	A	0.459	9.9 kb U *MGC139000*	2.70E-19	0.24 ± 0.027
BTB-01374567	9	2284075	1598499	AG	G	0.689	1 Mb blank	1.45E-18	0.26 ± 0.030
ARS-BFGL-NGS-55886	5	54231399	58154124	AG	G	0.757	*SLC16A7*	1.68E-17	0.28 ± 0.032
BTB-02036754	9	1952163	1229713	AG	G	0.205	1 Mb blank	2.62E-17	0.28 ± 0.033
BTB-00137287	3	77975986	83346371	AG	G	0.803	0.7 kb D *GADD45A*	1.46E-16	0.28 ± 0.034
ARS-BFGL-NGS-108538	11	107205272	106084	AG	G	0.782	1 Mb blank	1.55E-16	0.27 ± 0.033
BTA-58378-no-rs	5	54171859	58094584	AG	A	0.732	*SLC16A7*	2.74E-16	0.25 ± 0.030
ARS-BFGL-NGS-86658	29	6285416	6274933	AG	G	0.812	*NOX4*	6.50E-16	0.28 ± 0.034
BTB-02072771	9	2095979	1399218	AG	G	0.254	1 Mb blank	9.19E-16	0.25 ± 0.030
Hapmap49068-BTA-17115	8	37508875	39373846	AG	G	0.633	233.1 kb D *MGC127919*	1.06E-15	0.23 ± 0.028
BTB-00830411	22	4226071	4124586	AG	A	0.645	*RBMS3*	1.16E-15	0.23 ± 0.028
Hapmap48291-BTA-31854	X	134841753	79687560	AG	A	0.375	88.6 kb U *AP1S2*	1.38E-15	0.22 ± 0.028
BTB-00377789	9	2048367	1326785	AG	G	0.254	1 Mb blank	1.63E-15	0.24 ± 0.030
BTB-00378544	9	3029814	2137654	AC	A	0.543	*LOC788324*	3.28E-15	0.22 ± 0.027
BTA-98164-no-rs	8	38084197	39919118	AG	A	0.264	*LOC535671*	3.42E-15	0.24 ± 0.030
ARS-BFGL-NGS-2893	29	48501273	49692969	AG	A	0.085	*LOC618649*	3.43E-15	0.38 ± 0.048
BTB-01038990	X	2199470	266711	AG	G	0.209	1 Mb blank	5.92E-15	0.27 ± 0.034
ARS-BFGL-NGS-31434	15	NA	68425606	AC	C	0.895	*LOC783480*	6.17E-15	0.35 ± 0.045
Hapmap49653-BTA-54778	22	5741589	5635349	AG	A	0.476	75.9 kb U *LOC789738*	1.29E-14	0.21 ± 0.027
ARS-BFGL-NGS-103613	9	1427119	672234	AG	G	0.328	1 Mb Blank	1.42E-14	0.22 ± 0.028

SNP, single nucleotide polymorphism; PTA, predicted transmitting ability; Chr, chromosome; UMD, University of Maryland; Btau_4.0, Baylor College of Medicine bovine genome assembly Build 4.0; A, SNP alleles; FA, favorable allele; FAF, FA frequency; U, upstream from; D, downstream from; SE, standard error.

Table S2.20: Top 20 most significant SNP effects for PTA for fore udder attachment

SNP	Chr	UMD position	Btau_4.0 position	A	FA	FAF	Gene region	*P* value	Effect ± SE
ARS-BFGL-NGS-83607	16	1756016	937280	AG	A	0.586	*REN*	1.20E-34	0.48 ± 0.038
Hapmap46795-BTA-30632	X	131766182	77183469	AG	G	0.422	*PHKA2*	2.19E-30	0.45 ± 0.038
ARS-BFGL-BAC-2591	22	34385123	35072320	AG	G	0.514	132.7 kb D *SUCLG2*	2.40E-29	0.43 ± 0.038
ARS-BFGL-NGS-94063	7	4436640	4431857	AG	G	0.888	*CRTC1*	8.87E-29	0.68 ± 0.060
Hapmap30462-BTC-059597	25	5836611	6621976	AG	A	0.364	135.1 kb U *A2BP1*	4.50E-28	0.43 ± 0.039
Hapmap49788-BTA-88951	22	34311771	34998652	AG	A	0.668	59 kb D *SUCLG2*	1.16E-27	0.45 ± 0.040
ARS-BFGL-NGS-27636	19	57655339	58732295	AG	A	0.176	*GPRC5C*	1.17E-27	0.54 ± 0.049
Hapmap41275-BTA-47175	10	64905140	69491	AG	A	0.511	*APC*	1.22E-27	0.42 ± 0.038
ARS-BFGL-NGS-3506	5	70897603	75705849	AG	G	0.374	25.9 kb U *BTBD11*	1.02E-26	0.43 ± 0.039
BTA-74498-no-rs	5	85980094	91980041	AG	A	0.165	381 kb D *BCAT1*	1.05E-26	0.55 ± 0.051
ARS-BFGL-NGS-52939	19	56244442	57331979	AG	A	0.172	*SRP68*	2.84E-26	0.54 ± 0.050
ARS-BFGL-NGS-89394	23	3972340	2511218	AG	G	0.250	*COL21A1*	3.65E-26	0.47 ± 0.044
ARS-BFGL-NGS-22907	X	131710317	77127986	AG	G	0.548	13.9 kb U *PHKA2*	8.12E-26	0.40 ± 0.038
ARS-BFGL-NGS-19442	5	70997483	75805133	AG	A	0.383	*BTBD11*	9.64E-26	0.42 ± 0.040
ARS-BFGL-NGS-32517	26	47907512	48311186	AG	G	0.315	28.2 kb D *MKI67*	9.55E-25	0.43 ± 0.041
ARS-BFGL-NGS-12185	X	145243690	983791	AC	C	0.121	261.8 kb U *LOC100140451*	1.13E-24	0.60 ± 0.057
ARS-BFGL-NGS-46941	19	56300082	57387665	CG	G	0.231	*LOC510634*	1.17E-24	0.46 ± 0.044
ARS-BFGL-NGS-27758	19	57876833	58971180	AG	A	0.186	36.1 kb D *RPL38*	1.57E-24	0.50 ± 0.048
ARS-BFGL-NGS-31361	20	26602523	28328058	AG	A	0.879	334.5 kb D *PELO*	2.28E-24	0.61 ± 0.059
BTB-00432889	10	62653672	64107324	AG	A	0.453	164.1 kb D *SLC24A5*	4.72E-24	0.47 ± 0.046

SNP, single nucleotide polymorphism; PTA, predicted transmitting ability; Chr, chromosome; UMD, University of Maryland; Btau_4.0, Baylor College of Medicine bovine genome assembly Build 4.0; A, SNP alleles; FA, favorable allele; FAF, FA frequency; U, upstream from; D, downstream from; SE, standard error.

Table S2.21: Top 20 most significant SNP effects for PTA for rear udder height

SNP	Chr	UMD position	Btau_4.0 position	A	FA	FAF	Gene region	*P* value	Effect ± SE
ARS-BFGL-NGS-100791	2	35004695	36035875	AG	G	0.307	58.3 kb U *TBR1*	2.17E-31	0.51 ± 0.042
Hapmap41275-BTA-47175	10	64905140	69491	AG	A	0.511	*APC*	3.26E-31	0.48 ± 0.040
ARS-BFGL-NGS-13350	10	37830642	37625930	AG	A	0.550	*CAPN3*	5.36E-27	0.43 ± 0.040
BTA-95758-no-rs	2	35043749	36074929	AG	A	0.309	19.2 kb U *TBR1*	4.49E-26	0.46 ± 0.043
Hapmap48024-BTA-62291	10	37793494	37589440	AG	G	0.551	*GANC*	5.73E-26	0.42 ± 0.040
ARS-BFGL-NGS-12185	X	145243690	983791	AC	C	0.121	261.8 kb U *LOC100140451*	1.05E-25	0.64 ± 0.060
ARS-BFGL-NGS-17917	X	126011140	72897125	AG	A	0.051	*LOC784572*	4.43E-25	0.93 ± 0.089
BTB-00432889	10	62653672	64107324	AG	A	0.453	164.1 kb D *SLC24A5*	5.15E-25	0.51 ± 0.049
ARS-BFGL-NGS-83607	16	1756016	937280	AG	A	0.586	*REN*	5.20E-25	0.43 ± 0.041
ARS-BFGL-NGS-28849	28	1283286	2397050	AC	A	0.023	*GALNT2*	8.27E-25	1.40 ± 0.134
Hapmap46795-BTA-30632	X	131766182	77183469	AG	G	0.422	*PHKA2*	9.56E-25	0.43 ± 0.041
BTA-89794-no-rs	11	284026	335036	AG	G	0.116	*TMEM87B*	2.16E-24	0.63 ± 0.061
Hapmap60155-rs29010222	10	38376013	38216575	AG	A	0.414	*UBR1*	2.74E-24	0.42 ± 0.040
Hapmap53237-rs29021167	10	63095461	64551774	AG	G	0.631	173.7 kb D *SEMA6D*	3.47E-24	0.43 ± 0.042
ARS-BFGL-NGS-11105	11	78510118	80795967	AG	A	0.239	*LOC529399*	3.94E-24	0.48 ± 0.047
BTB-01045439	2	29839570	30784774	AC	A	0.382	93.1 kb U *SCN7A*	8.18E-24	0.43 ± 0.042
Hapmap34838-BES10_Contig603_291	10	38424075	38264639	AG	A	0.418	*UBR1*	9.10E-24	0.41± 0.040
ARS-BFGL-NGS-2015	11	84872349	87604666	AG	A	0.255	323 kb D *TRIB2*	2.46E-23	0.46 ± 0.045
Hapmap22874-BTA-153755	11	83168133	85760282	AC	C	0.231	*NBAS*	2.50E-23	0.47 ± 0.046
ARS-BFGL-NGS-43868	7	91946384	90768813	CG	C	0.380	347.4 kb U *CETN3*	3.29E-23	0.41 ± 0.041

SNP, single nucleotide polymorphism; PTA, predicted transmitting ability; Chr, chromosome; UMD, University of Maryland; Btau_4.0, Baylor College of Medicine bovine genome assembly Build 4.0; A, SNP alleles; FA, favorable allele; FAF, FA frequency; U, upstream from; D, downstream from; SE, standard error.

Table S2.22: Top 20 most significant SNP effects for PTA for udder depth

SNP	Chr	UMD position	Btau_4.0 position	A	FA	FAF	Gene region	*P* value	Effect ± SE
Hapmap30462-BTC-059597	25	5836611	6621976	AG	A	0.364	135.1 kb U *A2BP1*	5.24E-34	0.41 ± 0.033
ARS-BFGL-BAC-2591	22	34385123	35072320	AG	G	0.514	132.7 kb D *SUCLG2*	1.74E-31	0.39 ± 0.033
ARS-BFGL-NGS-94063	7	4436640	4431857	AG	G	0.888	*CRTC1*	4.39E-31	0.61 ± 0.052
Hapmap41291-BTA-53265	21	11329938	10046664	AG	A	0.852	1 Mb blank	3.49E-29	0.53 ± 0.046
Hapmap58322-rs29013674	26	36734119	36806927	AG	G	0.421	55.5 kb U *GFRA1*	6.13E-29	0.38 ± 0.033
ARS-BFGL-NGS-51630	20	5096097	5270231	CG	C	0.460	91.4 kb U *FAM44B*	8.50E-29	0.38 ± 0.034
Hapmap49788-BTA-88951	22	34311771	34998652	AG	A	0.668	59 kb D *SUCLG2*	4.51E-28	0.39 ± 0.035
ARS-BFGL-NGS-89394	23	3972340	2511218	AG	G	0.250	*COL21A1*	7.47E-28	0.42 ± 0.038
ARS-BFGL-NGS-65709	21	39496121	39768355	AG	A	0.829	196.8 kb U *FOXG1*	1.47E-27	0.49 ± 0.044
ARS-BFGL-NGS-4774	7	17403976	14635617	AC	A	0.323	1.5 kb D *INSR*	2.19E-27	0.38 ± 0.035
ARS-BFGL-NGS-35882	X	137586078	83553143	AG	G	0.237	*2.8 KB U ARHGAP6*	8.87E-26	0.41 ± 0.038
ARS-BFGL-NGS-58314	20	10871544	11625506	AG	A	0.567	1 Mb blank	2.07E-25	0.34 ± 0.032
Hapmap44030-BTA-18247	4	87944727	90209358	AG	G	0.277	*CADPS2*	2.75E-25	0.39 ± 0.037
ARS-BFGL-NGS-79176	17	46558798	48063466	AC	A	0.562	56 kb D *LOC100138728*	3.20E-25	0.35 ± 0.033
BFGL-NGS-113216	26	39220494	39267843	AT	A	0.400	*PRLHR*	3.22E-25	0.35 ± 0.033
ARS-BFGL-BAC-32111	17	46535046	48039716	AC	A	0.565	32.3 kb D *LOC100138728*	4.87E-25	0.35 ± 0.033
Hapmap39897-BTA-18318	17	14357124	15155735	AC	C	0.192	*LOC519752*	1.01E-24	0.43 ± 0.041
ARS-BFGL-NGS-31361	20	26602523	28328058	AG	A	0.879	334.5 kb D *PELO*	1.17E-24	0.53 ± 0.051
Hapmap47152-BTA-101314	9	87568944	89595204	AG	A	0.678	96.1 kb D *UST*	2.82E-24	0.37 ± 0.035
BTB-00607669	15	59020999	57826656	AG	A	0.570	16.8 kb D *LIN7C*	3.15E-24	0.34 ± 0.033

SNP, single nucleotide polymorphism; PTA, predicted transmitting ability; Chr, chromosome; UMD, University of Maryland; Btau_4.0, Baylor College of Medicine bovine genome assembly Build 4.0; A, SNP alleles; FA, favorable allele; FAF, FA frequency; U, upstream from; D, downstream from; SE, standard error.

Table S2.23: Top 20 most significant SNP effects for PTA for udder cleft

SNP	Chr	UMD position	Btau_4.0 position	A	FA	FAF	Gene region	*P* value	Effect ± SE
ARS-BFGL-NGS-43868	7	91946384	90768813	CG	C	0.380	347.4 kb U *CETN3*	1.20E-38	0.46 ± 0.034
ARS-BFGL-NGS-27636	19	57655339	58732295	AG	A	0.176	*GPRC5C*	8.55E-32	0.53 ± 0.044
BTA-21135-no-rs	7	91904522	90726623	AG	A	0.344	389.6 kb U *CETN3*	1.12E-31	0.42 ± 0.035
Hapmap49911-BTA-21136	7	91882019	90704153	AG	A	0.343	412.1 kb U *CETN3*	2.04E-31	0.42 ± 0.035
ARS-BFGL-NGS-52939	19	56244442	57331979	AG	A	0.172	*SRP68*	2.40E-30	0.53 ± 0.045
BTB-00283603	6	109719477	118855815	AG	G	0.421	*LETM1*	3.96E-30	0.40 ± 0.035
ARS-BFGL-NGS-104207	6	109835444	119038391	AG	G	0.453	*WHSC2*	1.26E-27	0.38 ± 0.035
BTB-00951480	27	11413201	13511496	AG	A	0.528	1 Mb blank	5.80E-27	0.38 ± 0.034
BTB-01944037	8	108772548	112370481	AG	A	0.591	56.3 kb U *TLR4*	8.63E-27	0.37 ± 0.034
ARS-BFGL-NGS-27758	19	57876833	58971180	AG	A	0.186	36.1 kb D *RPL38*	9.16E-27	0.48 ± 0.044
ARS-BFGL-NGS-7156	X	7288581	2436768	AG	G	0.452	*GRIA3*	7.28E-26	0.36 ± 0.034
Hapmap49872-BTA-115580	7	89967934	88846849	AG	A	0.461	44.7 kb D *LOC100140711*	1.07E-25	0.36 ± 0.034
BTA-80441-no-rs	7	103779001	103091828	AG	A	0.660	4.8 kb D *SLCO4C1*	1.28E-25	0.38 ± 0.036
BFGL-NGS-113793	2	32770767	33743658	AG	A	0.626	*FIGN*	2.77E-25	0.38 ± 0.036
ARS-BFGL-NGS-106313	21	10761902	9479722	AG	G	0.265	29.7 kb U *NR2F2*	3.13E-25	0.41 ± 0.039
ARS-BFGL-NGS-13027	X	84566018	49078853	AG	A	0.446	*TAF1*	3.63E-25	0.36 ± 0.034
BTB-00757888	1	16923285	17106220	AG	G	0.308	1 Mb blank	7.56E-25	0.40 ± 0.038
BTB-00141857	3	88766772	94542660	AG	A	0.077	1 Mb blank	1.24E-24	0.67 ± 0.065
Hapmap40831-BTA-88715	7	98254815	97249605	AG	G	0.755	54.3 kb D *PCSK1*	2.19E-24	0.41 ± 0.040
Hapmap41879-BTA-47145	2	32880955	33853646	AG	G	0.641	30.8 kb U *FIGN*	3.48E-24	0.37 ± 0.036

SNP, single nucleotide polymorphism; PTA, predicted transmitting ability; Chr, chromosome; Btau_4.0, Baylor College of Medicine bovine genome assembly Build 4.0; UMD, University of Maryland; A, SNP alleles; FA, favorable allele; FAF, FA frequency; U, upstream from; D, downstream from; SE, standard error.

Table S2.24: Top 20 most significant SNP effects for PTA for front teat placement

SNP	Chr	UMD position	Btau_4.0 position	A	FA	FAF	Gene region	*P* value	Effect ± SE
BTB-00283603	6	109719477	118855815	AG	G	0.421	*LETM1*	3.27E-22	0.31 ± 0.032
Hapmap35054-BES7_Contig354_1224	6	101414694	103627410	AG	A	0.669	*WDFY3*	4.02E-20	0.31 ± 0.033
BFGL-NGS-119467	16	50966489	47043974	AC	C	0.757	*LOC100137803*	6.26E-20	0.34 ± 0.037
Hapmap42635-BTA-68718	20	4618689	4745147	AG	G	0.398	*ERGIC1*	1.22E-19	0.29 ± 0.032
ARS-BFGL-NGS-94063	7	4436640	4431857	AG	G	0.888	*CRTC1*	1.49E-19	0.46 ± 0.050
ARS-BFGL-NGS-97753	9	88503741	90743220	AG	G	0.734	32.5 kb U *IYD*	4.24E-19	0.32 ± 0.036
BTB-01356178	20	2491375	561328	AG	G	0.368	*KCNIP1*	9.62E-19	0.28± 0.031
UA-IFASA-6733	9	94235535	96580538	AG	A	0.605	96 kb U *LOC514725*	1.25E-18	0.29 ± 0.032
ARS-BFGL-NGS-44177	16	50928943	47006426	AG	A	0.722	*LOC100137803*	1.78E-18	0.31 ± 0.035
ARS-BFGL-NGS-58314	20	10871544	11625506	AG	A	0.567	1 Mb blank	5.73E-18	0.27 ± 0.031
ARS-BFGL-NGS-26480	16	50896880	46974362	AC	A	0.731	*LOC100137803*	8.29E-18	0.33 ± 0.038
ARS-BFGL-NGS-104207	6	109835444	119038391	AG	G	0.453	*WHSC2*	8.94E-18	0.28 ± 0.032
ARS-BFGL-NGS-18896	28	42720661	42251712	AC	C	0.451	80 kb D *GDF10*	3.02E-17	0.26 ± 0.031
ARS-BFGL-NGS-25823	9	88448285	90579032	AG	G	0.671	*PPP1R14C*	5.49E-17	0.28 ± 0.033
ARS-BFGL-NGS-87800	6	615089	545275	AG	A	0.592	8.4 kb D *LOC541108*	6.27E-17	0.26 ± 0.031
Hapmap27696-BTC-045782	6	101427754	103640465	AG	G	0.683	*WDFY3*	6.71E-17	0.28 ± 0.033
Hapmap38362-BTA-94562	6	NA	514960	AC	C	0.594	1 kb U *LOC782218*	7.13E-17	0.26 ± 0.031
BTB-00491839	12	46917339	46825696	AC	C	0.812	61.3 kb D *DACH1*	9.89E-17	0.34 ± 0.040
Hapmap27802-BTA-49707	5	68946279	73634208	AG	A	0.438	4.5 kb D *LOC516864*	1.07E-16	0.27 ± 0.033
ARS-BFGL-NGS-43868	7	91946384	90768813	CG	C	0.380	347.4 kb U *CETN3*	1.24E-16	0.27 ± 0.032

SNP, single nucleotide polymorphism; PTA, predicted transmitting ability; Chr, chromosome; UMD, University of Maryland; Btau_4.0, Baylor College of Medicine bovine genome assembly Build 4.0; A, SNP alleles; FA, favorable allele; FAF, FA frequency; U, upstream from; D, downstream from; SE, standard error.

Table S2.25: Top 20 most significant SNP effects for PTA for rear teat placement

SNP	Chr	UMD position	Btau_4.0 position	A	FA	FAF	Gene region	*P* value	Effect ± SE
ARS-BFGL-NGS-43868	7	91946384	90768813	CG	C	0.380	347.4 kb U *CETN3*	9.46E-26	0.34 ± 0.032
ARS-BFGL-NGS-13027	X	84566018	49078853	AG	A	0.446	*TAF1*	2.69E-21	0.30 ± 0.031
BTA-21135-no-rs	7	91904522	90726623	AG	A	0.344	389.6 kb U *CETN3*	7.93E-21	0.31 ± 0.033
Hapmap49911-BTA-21136	7	91882019	90704153	AG	A	0.343	412.1 kb U *CETN3*	1.42E-20	0.31 ± 0.033
ARS-BFGL-NGS-17429	16	22942419	21329254	AG	A	0.734	1 Mb blank	2.47E-20	0.33 ± 0.035
BTB-00283603	6	109719477	118855815	AG	G	0.421	*LETM1*	4.62E-20	0.30 ± 0.032
Hapmap44494-BTA-90423	11	80637936	82646767	AG	A	0.512	59.2 kb D *LOC788214*	5.84E-20	0.30 ± 0.032
BTB-00141857	3	88766772	94542660	AG	A	0.077	1 Mb blank	5.66E-19	0.54 ± 0.060
BFGL-NGS-116174	6	426582	479837	AG	A	0.377	36.1 kb U *LOC782218*	8.42E-18	0.28 ± 0.033
ARS-BFGL-NGS-27636	19	57655339	58732295	AG	A	0.176	*GPRC5C*	8.45E-18	0.36 ± 0.042
UA-IFASA-6733	9	94235535	96580538	AG	A	0.605	96 kb U *LOC514725*	1.05E-17	0.28 ± 0.033
ARS-BFGL-NGS-104207	6	109835444	119038391	AG	G	0.453	*WHSC2*	1.72E-17	0.28 ± 0.032
Hapmap49872-BTA-115580	7	89967934	88846849	AG	A	0.461	44.7 kb D *LOC100140711*	1.94E-17	0.27 ± 0.032
BFGL-NGS-116109	15	34763290	32924180	AC	C	0.745	*LOC517332–SCN3B*	2.84E-17	0.31 ± 0.036
BTB-00951480	27	11413201	13511496	AG	A	0.528	1 Mb blank	3.06E-17	0.27 ± 0.032
ARS-BFGL-NGS-89535	16	22179897	20474158	AG	A	0.743	138.7 kb D *SPATA17*	5.61E-17	0.30 ± 0.036
BTB-00590405	15	31527773	29557995	AG	A	0.720	*ARHGEF12*	9.66E-17	0.30 ± 0.035
ARS-BFGL-NGS-38049	15	51041325	49842952	AG	A	0.333	*TRIM68*	1.08E-16	0.28 ± 0.034
BTA-43315-no-rs	1	6254730	6219142	AG	A	0.626	65.6 kb U *C1H21ORF7*	1.23E-16	0.27 ± 0.032
BFGL-NGS-118379	5	7022191	7473430	AG	G	0.779	82.8 kb U *LOC100138438*	2.84E-16	0.32 ± 0.038

SNP, single nucleotide polymorphism; PTA, predicted transmitting ability; Chr, chromosome; UMD, University of Maryland; Btau_4.0, Baylor College of Medicine bovine genome assembly Build 4.0; A, SNP alleles; FA, favorable allele; FAF, FA frequency; U, upstream from; D, downstream from; SE, standard error.

Table S2.26: Top 20 most significant SNP effects for teat length

SNP	Chr	UMD position	Btau_4.0 Position	A	FA	FAF	Gene region	*P* value	Effect ± SE
BTB-01444868	11	23741433	24949975	AC	A	0.708	98.5 kb D *LOC615674*	1.12E-23	0.29 ± 0.028
Hapmap50642-BTA-28388	26	49137602	49579691	AG	A	0.911	80.8 kb U *MGMT*	1.17E-23	0.45 ± 0.044
BFGL-NGS-111445	26	48120342	48539159	AG	G	0.911	1 Mb blank	3.72E-23	0.45 ± 0.044
ARS-BFGL-NGS-2185	21	21730828	21060959	AC	C	0.886	*MGC139355*	6.46E-23	0.40 ±0.040
ARS-BFGL-NGS-54076	11	19248965	21289389	AG	A	0.802	*VIT*	3.62E-22	0.31 ±0.032
ARS-BFGL-NGS-77485	17	4926550	5305783	AC	A	0.474	41.7 kb U *TMEM154*	2.58E-21	0.25 ±0.026
ARS-BFGL-NGS-104962	17	5487182	6003582	AG	G	0.704	192 kb D *FBXW7*	3.14E-21	0.28 ±0.029
ARS-BFGL-NGS-24849	17	5511241	6027682	AG	G	0.704	216.1 kb D *FBXW7*	3.21E-21	0.28 ±0.029
ARS-BFGL-NGS-103695	21	21333104	20671339	AG	G	0.564	25 kb U *RHCG*	5.50E-21	0.25 ±0.026
ARS-BFGL-BAC-34040[a]	17	18764770	19749801	AC	A	0.892	2.9 kb D *LOC528054*	9.03E-21	0.39 ±0.041
Hapmap59290-rs29022016	11	23599074	24790247	AG	A	0.879	60.9 kb U *LOC615674*	1.25E-20	0.41 ±0.044
ARS-BFGL-NGS-1096	X	12604306	5329598	AG	G	0.926	1 Mb blank	1.96E-20	0.47 ±0.050
ARS-BFGL-NGS-16708	17	15107947	15883911	AC	C	0.873	187.4 kb D *USP38*	2.07E-20	0.36 ±0.038
Hapmap50694-BTA-44240	1	96299260	97648248	AG	A	0.766	77.4 kb D *LOC507471*	2.40E-20	0.29 ±0.031
BFGL-NGS-112365	18	64548360	64574451	AG	G	0.773	*LOC100137761*	5.48E-20	0.28 ±0.030
BTA-51857-no-rs	21	21017358	20364700	AG	G	0.670	*ABHD2*	6.24E-20	0.25 ±0.027
ARS-BFGL-NGS-90639	11	43296086	45085020	AG	G	0.550	43.8 kb U *LOC100137915*	7.00E-20	0.24 ±0.026
Hapmap31185-BTA-159806	4	12654239	13038423	AG	A	0.108	*ASB4*	1.94E-19	0.37 ±0.041
BTA-29287-no-rs	18	58696066	58353743	AG	G	0.886	*LOC787057*	2.14E-19	0.36 ±0.040
ARS-BFGL-NGS-76683	X	7042383	2191062	AG	G	0.826	136.3 kb U *GRIA3*	3.41E-19	0.30 ±0.034

SNP, single nucleotide polymorphism; PTA, predicted transmitting ability; Chr, chromosome; UMD, University of Maryland; Btau_4.0, Baylor College of Medicine bovine genome assembly Build 4.0; A, SNP alleles; FA, favorable allele; FAF, FA frequency; U, upstream from; D, downstream from; SE, standard error.

Table S2.27: Top 20 most significant SNP effects for foot angle

SNP	Chr	UMD position	Btau_4.0 position	A	FA	FAF	Gene region	*P* value	Effect ± SE
Hapmap28514-BTA-163525	26	49185154	49627246	CG	G	0.149	*MGMT*	4.95E-39	0.64 ± 0.048
Hapmap50642-BTA-28388	26	49137602	49579691	AG	G	0.089	80.8 kb U *MGMT*	3.25E-38	0.79 ± 0.060
BFGL-NGS-111445	26	48120342	48539159	AG	A	0.089	1 Mb blank	4.00E-38	0.80 ± 0.060
BFGL-NGS-117931	13	20788173	19783855	AC	A	0.687	182.1 kb D *ARL5B*	1.03E-33	0.47 ± 0.038
ARS-BFGL-NGS-29910	20	9468693	10266581	AG	A	0.195	54.1 kb D *MAP1B*	2.74E-33	0.54 ± 0.044
BTA-25900-no-rs	13	18783020	17759292	AC	A	0.678	*PARD3*	1.49E-32	0.46 ± 0.038
ARS-BFGL-NGS-1096	X	12604306	5329598	AG	A	0.074	1 Mb blank	2.46E-32	0.82 ± 0.068
ARS-BFGL-BAC-32721	17	34377274	35876692	AG	G	0.129	372.2 kb U *SPRY1*	4.78E-32	0.63 ± 0.052
BFGL-NGS-117985	18	53948569	53311437	AG	G	0.091	0.4 kb D *PGLYRP1*	4.79E-32	0.72 ± 0.060
ARS-BFGL-NGS-32517	26	47907512	48311186	AG	G	0.315	28.2 kb D *MKI67*	1.42E-31	0.46 ± 0.038
BTB-01308199	17	33827641	35340688	AG	A	0.121	351 kb D *ANKRD50*	1.69E-31	0.63 ± 0.053
ARS-BFGL-NGS-16708	17	15107947	15883911	AC	A	0.127	187.4 kb D *USP38*	2.43E-29	0.60 ± 0.052
ARS-BFGL-BAC-33654	20	19587484	21086210	AG	A	0.112	*PDE4D*	3.17E-29	0.64 ± 0.056
ARS-BFGL-NGS-1312	26	48583446	48998305	AG	A	0.107	1 Mb blank	4.77E-29	0.64 ± 0.056
ARS-BFGL-NGS-46579	26	39655739	39777201	AT	T	0.271	*SFXN4*	9.96E-29	0.45 ± 0.039
BTB-00775883	20	19187276	20681060	AG	G	0.314	160.8 kb U *PDE4D*	1.77E-28	0.43 ± 0.038
Hapmap51582-BTA-31036	X	146244046	86367264	AG	A	0.096	113.2 kb D *LOC616260*	1.93E-28	0.66 ± 0.058
UA-IFASA-8063	14	70387887	66250582	AG	A	0.526	11.7 kb U *GDF6*	6.60E-28	0.40 ± 0.035
Hapmap54948-rs29009927	5	10482135	12423627	AG	G	0.543	*LIN7A*	7.54E-28	0.39 ± 0.035
ARS-BFGL-NGS-61317	26	39288059	39335884	AG	A	0.229	4.5 kb U *C10ORF46*	7.65E-28	0.46 ± 0.042

SNP, single nucleotide polymorphism; PTA, predicted transmitting ability; Chr, chromosome; UMD, University of Maryland; Btau_4.0, Baylor College of Medicine bovine genome assembly Build 4.0; A, SNP alleles; FA, favorable allele; FAF, FA frequency; U, upstream from; D, downstream from; SE, standard error.

Table S2.28: Top 20 most significant SNP effects for rear legs (side view)

SNP	Chr	UMD position	Btau_4.0 position	A	FA	FAF	Gene region	*P* value	Effect ± SE ($)
BTB-01333903	1	28362687	29095948	AG	A	0.933	220.9 kb U *LOC521010*	2.54E-28	0.57±0.051
ARS-BFGL-NGS-4463	18	49167271	33210	AG	A	0.831	1 Mb blank	3.81E-28	0.38 ±0.034
BFGL-NGS-117985	18	53948569	53311437	AG	A	0.909	0.4 kb D *PGLYRP1*	1.90E-27	0.49 ±0.044
BTA-95509-no-rs	X	59080285	331965	AG	G	0.815	1 Mb blank	3.94E-27	0.36 ±0.033
ARS-BFGL-NGS-61814	8	11212361	11432925	AC	A	0.662	8.3 kb D *TMEM215*	1.41E-24	0.30 ±0.028
Hapmap51817-BTA-22015	26	2915575	2430289	AG	G	0.909	14.8 kb D *LOC785617*	2.50E-24	0.46±0.044
ARS-BFGL-NGS-99872	18	52434006	51833397	AG	G	0.778	*ZNF45*	3.88E-24	0.32 ±0.031
ARS-BFGL-NGS-29650	27	34326743	36946859	AG	G	0.817	*ADAM2*	1.69E-23	0.34 ±0.034
ARS-BFGL-NGS-93601	11	82920732	85461370	AG	G	0.824	*NBAS*	1.80E-23	0.34 ±0.033
ARS-BFGL-BAC-14883	1	14102864	14360342	AG	A	0.836	1 Mb blank	8.45E-23	0.35 ±0.035
ARS-BFGL-NGS-13673	1	87402236	88871762	AC	C	0.910	428.3 kb D *TTC14*	9.73E-23	0.44 ±0.044
ARS-BFGL-NGS-1096	X	12604306	5329598	AG	G	0.926	1 Mb blank	1.77E-22	0.50 ±0.050
Hapmap56111-rs29019494	7	108638689	107881812	AT	T	0.668	404.1Kb U *EFNA5*	1.64E-21	0.27 ±0.028
BTA-29287-no-rs	18	58696066	58353743	AG	G	0.886	*LOC787057*	1.87E-21	0.39 ±0.040
BTA-39689-no-rs	16	62555582	58742814	AC	A	0.503	19.3 kb D *TOR1AIP1*	1.93E-21	0.25±0.026
Hapmap52518-rs29018446	16	68557811	64818695	AC	C	0.746	*HMCN1*	1.95E-21	0.29 ±0.030
Hapmap49666-BTA-57439	24	16306830	16735480	AG	G	0.865	1 Mb blank	2.95E-21	0.37 ±0.038
ARS-BFGL-NGS-61156	16	66221857	62401306	AC	C	0.267	48.6 kb U *RGL1*	3.80E-21	0.28 ±0.029
ARS-BFGL-NGS-107749	18	33986486	32927570	AG	G	0.953	3.9 kb U *LOC548614*	4.48E-21	0.57 ±0.060
ARS-BFGL-NGS-18028	X	106241123	63961868	CG	G	0.869	30.9 kb U *LOC520057*	5.94E-21	0.37 ±0.039

SNP, single nucleotide polymorphism; PTA, predicted transmitting ability; Chr, chromosome; UMD, University of Maryland; Btau_4.0, Baylor College of Medicine bovine genome assembly Build 4.0; A, SNP alleles; FA, favorable allele; FAF, FA frequency; U, upstream from; D, downstream from; SE, standard error.

Table S2.29: Top 20 most significant SNP effects for rear legs (rear view)

SNP	Chr	UMD position	Btau_4.0 position	A	FA	FAF	Gene region	*P* value	Effect ± SE
ARS-BFGL-NGS-10781	X	12754120	5478587	AT	T	0.386	142.868 kb U *ENSBTAG00000024206*	1.35E-29	0.38 ± 0.033
BTB-01660145	11	50264058	52206770	AG	A	0.621	*LOC538058*	1.16E-28	0.38 ± 0.034
Hapmap40477-BTA-105881	11	83526455	86116624	AG	G	0.567	129 kb U *FAM84A*	8.06E-28	0.37 ± 0.033
BFGL-NGS-114578	11	87279008	89954599	AG	G	0.668	*HPCAL1*	9.49E-28	0.38 ± 0.034
Hapmap38268-BTA-09661	X	142828641	85510888	AC	A	0.381	1 kb D *CLCN4*	1.80E-27	0.38 ± 0.035
Hapmap60265-rs29024291	X	31472189	18995329	AG	G	0.567	121.3 kb U *AFF2*	1.42E-26	0.37 ± 0.034
ARS-BFGL-NGS-2015	11	84872349	87604666	AG	A	0.255	323 kb D *TRIB2*	2.44E-26	0.40 ± 0.038
BTA-24681-no-rs	X	65614953	38365865	AG	G	0.705	120.9 kb D *TRPC5*	4.61E-26	0.40 ± 0.037
ARS-BFGL-BAC-11717	11	52195257	54071620	AG	G	0.837	1 Mb blank	1.06E-25	0.46 ± 0.044
Hapmap28514-BTA-163525	26	49185154	49627246	CG	G	0.149	*MGMT*	1.74E-25	0.48 ± 0.045
ARS-BFGL-NGS-24992	X	141204892	82221934	AG	A	0.080	3.6 kb D *FRMPD4*	1.92E-25	0.65 ± 0.062
Hapmap43873-BTA-50695	20	45819524	48503586	AG	G	0.789	1 Mb blank	2.58E-24	0.43 ± 0.041
ARS-BFGL-NGS-14236	11	86048363	88746533	AG	G	0.329	2.7 kb U *LPIN1*	4.05E-24	0.36 ± 0.035
ARS-BFGL-BAC-33654	20	19587484	21086210	AG	A	0.112	*PDE4D*	4.23E-24	0.54 ± 0.052
Hapmap54565-rs29010062	X	65746105	38492716	AG	A	0.569	390.4 kb U *LOC526223*	5.61E-24	0.35 ± 0.034
ARS-BFGL-NGS-32517	26	47907512	48311186	AG	G	0.315	28.2 kb D *MKI67*	2.17E-23	0.36 ± 0.036
Hapmap50711-BTA-49985	20	19520788	21019644	AG	G	0.134	*PDE4D*	5.99E-23	0.49 ± 0.049
Hapmap46768-BTA-117394	11	82643114	84956219	AG	G	0.671	339.6 kb U *MYCN*	9.09E-23	0.34 ± 0.034
ARS-BFGL-NGS-105586	11	81845043	83876375	AC	A	0.682	132.3 kKb U *FAM49A*	1.02E-22	0.35 ± 0.036
ARS-BFGL-BAC-16207	11	87999946	90794234	AG	A	0.405	*ITGB1BP1*	1.22E-22	0.34 ± 0.034

SNP, single nucleotide polymorphism; PTA, predicted transmitting ability; Chr, chromosome; UMD, University of Maryland; Btau_4.0, Baylor College of Medicine bovine genome assembly Build 4.0; A, SNP alleles; FA, favorable allele; FAF, FA frequency; U, upstream from; D, downstream from; SE, standard error.

Table S2.30: Top 20 most significant SNP effects for feet/legs score

SNP	Chr	UMD position	Btau_4.0 position	A	FA	FAF	Gene region	*P* value	Effect ± SE
Hapmap28514-BTA-163525	26	49185154	49627246	CG	G	0.149	*MGMT*	6.16E-36	0.54 ± 0.042
BTA-25900-no-rs	13	18783020	17759292	AC	A	0.678	*PARD3*	1.02E-33	0.41 ± 0.033
BFGL-NGS-117931	13	20788173	19783855	AC	A	0.687	182.1 kb D *ARL5B*	1.98E-32	0.40 ± 0.033
ARS-BFGL-NGS-32517	26	47907512	48311186	AG	G	0.315	28.2 kb D *MKI67*	2.23E-29	0.38 ± 0.034
Hapmap38268-BTA-09661	X	142828641	85510888	AC	A	0.381	1 kb D *CLCN4*	2.15E-26	0.35 ± 0.033
ARS-BFGL-NGS-24992	X	141204892	82221934	AG	A	0.080	3.6 kb D *FRMPD4*	2.27E-26	0.63 ± 0.058
ARS-BFGL-NGS-61317	26	39288059	39335884	AG	A	0.229	4.5 kb U *C10ORF46*	4.28E-26	0.39 ± 0.037
BTA-74498-no-rs	5	85980094	91980041	AG	A	0.165	381 kb D *BCAT1*	2.11E-25	0.45 ± 0.042
ARS-BFGL-NGS-106229	5	71749375	76653385	AG	G	0.420	*LOC100138309*	1.10E-24	0.34 ± 0.032
Hapmap60265-rs29024291	X	31472189	18995329	AG	G	0.567	121.3 kb U *AFF2*	2.49E-24	0.33 ± 0.032
BFGL-NGS-111445	26	48120342	48539159	AG	A	0.089	1 Mb blank	2.91E-24	0.55 ± 0.054
BTA-74479-no-rs	5	84613929	90582090	AG	A	0.169	286.0 kb U *IFLTD1*	4.72E-24	0.42 ± 0.041
BTA-24488-no-rs	13	21236959	20229797	AG	G	0.765	*LOC524240*	8.68E-24	0.36 ± 0.036
ARS-BFGL-NGS-10781	X	12754120	5478587	AT	T	0.386	142.868 kb U *ENSBTAG00000024206*	1.16E-23	0.32 ± 0.032
ARS-BFGL-NGS-21967	13	18762878	17739132	AG	A	0.707	*PARD3*	1.21E-23	0.34 ± 0.034
Hapmap50642-BTA-28388	26	49137602	49579691	AG	G	0.089	80.8 kb U *MGMT*	2.57E-23	0.54 ± 0.053
BTA-10187-rs29015749	5	89288565	95290942	AG	A	0.055	*SLCO1A2*	3.27E-23	0.67 ± 0.067
UA-IFASA-8063	14	70387887	66250582	AG	A	0.526	11.7 kb U *GDF6*	3.86E-23	0.31 ± 0.031
ARS-BFGL-NGS-2015	11	84872349	87604666	AG	A	0.255	323 kb D *TRIB2*	5.13E-23	0.36 ± 0.036
BTA-31857-no-rs	13	20091667	19079462	AG	A	0.526	32 kb D *NRP1*	7.11E-23	0.31 ± 0.031

SNP, single nucleotide polymorphism; PTA, predicted transmitting ability; Chr, chromosome; UMD, University of Maryland; Btau_4.0, Baylor College of Medicine bovine genome assembly Build 4.0; A, SNP alleles; FA, favorable allele; FAF, FA frequency; U, upstream from; D, downstream from; SE, standard error.

Table S2.31: Top 20 most significant SNP effects for final score, a composite index of body conformation traits

SNP	Chr	UMD position	Btau_4.0 position	A	FA	FAF	Gene region	*P* value	Effect ± SE
Hapmap46795-BTA-30632	X	131766182	77183469	AG	G	0.422	*PHKA2*	1.57E-37	0.41 ± 0.031
ARS-BFGL-NGS-83607	16	1756016	937280	AG	A	0.586	*REN*	1.35E-34	0.39 ± 0.031
Hapmap41275-BTA-47175	10	64905140	69491	AG	A	0.511	*APC*	9.16E-34	0.38 ± 0.031
ARS-BFGL-NGS-2015	11	84872349	87604666	AG	A	0.255	323 kb D *TRIB2*	7.38E-33	0.42 ± 0.035
ARS-BFGL-NGS-3506	5	70897603	75705849	AG	G	0.374	25.9 kb U *BTBD11*	3.97E-32	0.38 ± 0.032
Hapmap52066-rs29015690	11	85153576	87885711	AC	A	0.419	51.9 kb U *TRIB2*	5.86E-32	0.36 ± 0.030
ARS-BFGL-NGS-12185	X	145243690	983791	AC	C	0.121	261.8 kb U *LOC100140451*	5.96E-32	0.56 ± 0.047
BTA-74498-no-rs	5	85980094	91980041	AG	A	0.165	381 kb D *BCAT1*	1.54E-31	0.49 ± 0.041
ARS-BFGL-NGS-34903	11	83867859	86478143	AG	G	0.486	199.1 kb U *COPS2*	2.53E-31	0.37 ± 0.031
ARS-BFGL-NGS-19442	5	70997483	75805133	AG	A	0.383	*BTBD11*	5.91E-31	0.38 ± 0.032
ARS-BFGL-NGS-13350	10	37830642	37625930	AG	A	0.550	*CAPN3*	2.86E-30	0.36 ± 0.031
BTB-00432889	10	62653672	64107324	AG	A	0.453	164.1 kb D *SLC24A5*	1.21E-29	0.43 ± 0.037
ARS-BFGL-NGS-22907	X	131710317	77127986	AG	G	0.548	13.9Kb U *PHKA2*	1.35E-29	0.36 ± 0.031
Hapmap48024-BTA-62291	10	37793494	37589440	AG	G	0.551	*GANC*	1.43E-29	0.35 ± 0.031
ARS-BFGL-NGS-100791	2	35004695	36035875	AG	G	0.307	58.3 kb U *TBR1*	2.79E-29	0.38 ± 0.033
ARS-BFGL-NGS-27636	19	57655339	58732295	AG	A	0.176	*GPRC5C*	2.96E-29	0.46 ± 0.040
Hapmap22874-BTA-153755	11	83168133	85760282	AC	C	0.231	*NBAS*	3.26E-29	0.41 ± 0.036
ARS-BFGL-NGS-14236	11	86048363	88746533	AG	G	0.329	2.7 kb U *LPIN1*	1.23E-28	0.37 ± 0.033
ARS-BFGL-NGS-94063	7	4436640	4431857	AG	G	0.888	*CRTC1*	4.28E-28	0.55 ± 0.049
ARS-BFGL-NGS-17917	X	126011140	72897125	AG	A	0.051	*LOC784572*	7.71E-28	0.76 ± 0.069

SNP, single nucleotide polymorphism; PTA, predicted transmitting ability; Chr, chromosome; UMD, University of Maryland; Btau_4.0, Baylor College of Medicine bovine genome assembly Build 4.0; A, SNP alleles; FA, favorable allele; FAF, FA frequency; U, upstream from; D, downstream from; SE, standard error.
