# Supplementary material for: Genome-wide association analysis of thirty one production, health, reproduction and body conformation traits in contemporary U.S. Holstein cows
Source: BMC Genomics. 2011 Aug 11;12:408. doi: 10.1186/1471-2164-12-408 (PMC3176260; doi:10.1186/1471-2164-12-408)
Supplement: Additional file 6 — Figure S4. Gene clusters that overlapped localized concentrations of SNP effects or contained significant SNP effects on Bos taurus (BTA) chromosomes. A) BTA7 15.4 Mb gene cluster (Btau_4.0:4807980-20004663) of ~1,166 genes (not counting pseudo and RNA genes) with a mean of 75 genes/Mb. Left end was LSM4-JUND region; right end was LSM7-SPPL2B-OAZ1 region. This cluster included ARS-BFGL-NGS-4774 [1.5 kb downstream from INSR (Q95M43; highlighted in green)], which was significant for somatic cell score (1st) and daughter pregnancy rate (1st), productive life (3rd), and net merit (78th)] as well as all 10 significant SNP effects on this chromosome for productive life, 15 of the 16 significant SNP effects for daughter pregnancy rate, and 6 of the 12 significant SNP effects for somatic cell score. B) BTA14 2.81 Mb gene cluster (Btau_4.0:50872-2,859,132) of ~125 genes with a mean of 44 genes/Mb; NIBP (highlighted in green) was the largest gene (387.23 kb) in the cluster. This cluster included 19 significant SNP effects for fat percentage [SNP in DGAT1 was 1st and SNP in NIBP was 2nd], one SNP in VPS28 with effect for milk yield, and one SNP in NIBP with effects for fat yield and protein percentage. C) BTA18 15.82 Mb gene cluster (Btau_4.0:48755332-64574451) of ~1,322 genes with a mean of 83 genes/Mb. The PGLYRP1(PGRP)-IGFL1 (highlighted in green) region had the most significant SNP effects in the cluster [fat and protein yields, service-sire and daughter calving ease, and net merit (1st); service-sire stillbirth (8th); milk yield (9th); productive life (16th); and fat and protein percentages (25th)]. D) BTA3 gene cluster with significant SNPs for fat yield [BFGL-NGS-113990 (49th) and INRA-304 (53rd)]. E) BTA5 gene cluster with ARS-BFGLNGS-36745 (associated with SREBF2) and ARS-BFGL-NGS-71946 in LOC535121, which were among top 100 SNP effects for fat and protein percentages and service-sire calving ease and stillbirth, as well as other significant SNPS for service-sire stil [file 1471-2164-12-408-S6.PDF]

A

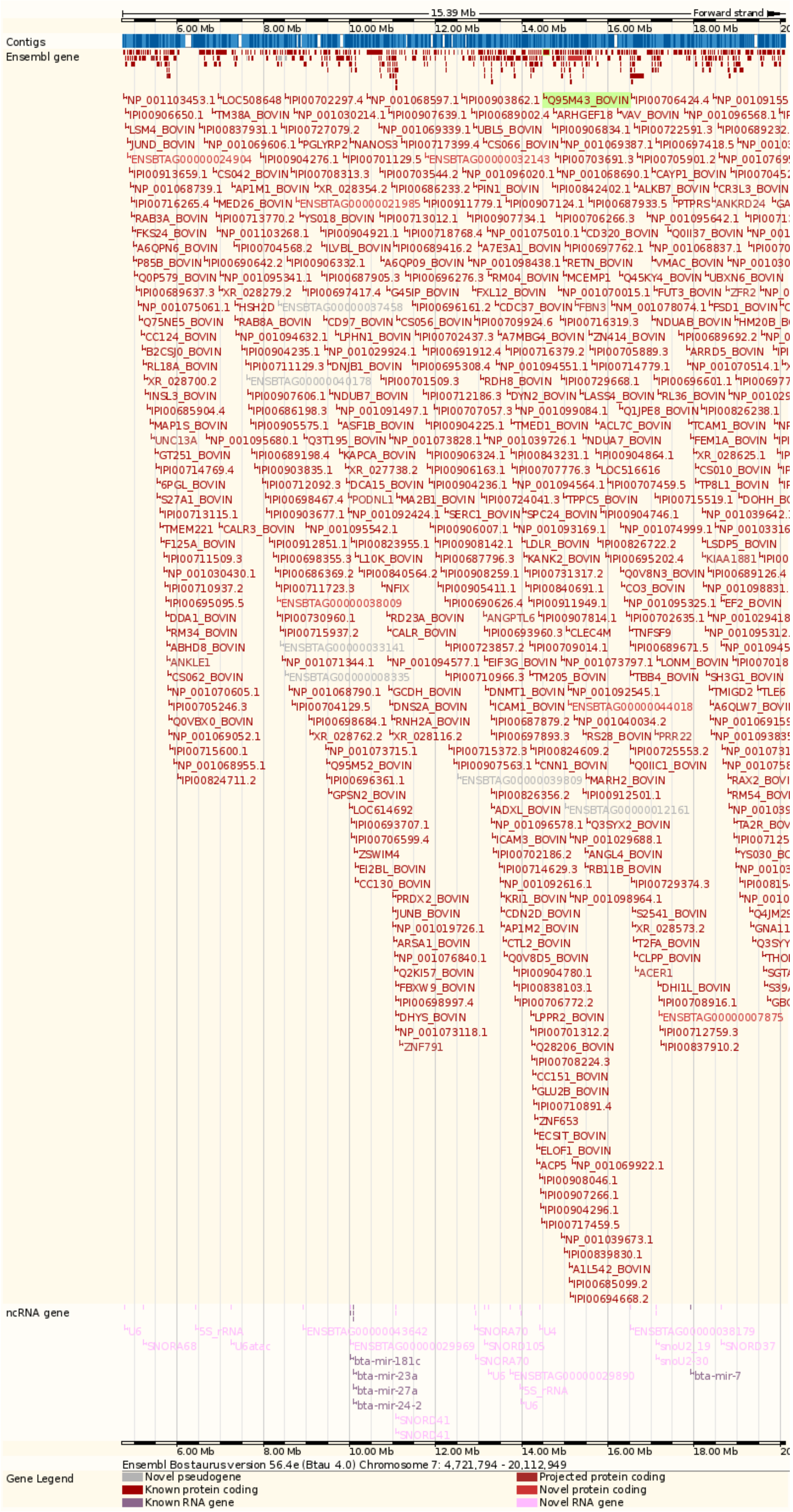
15.39 Mb
Forward strand
6.00 Mb
8.00 Mb
10.00 Mb
12.00 Mb
14.00 Mb
16.00 Mb
18.00 Mb
Contigs
Ensembl gene
Q95M43_BOVIN
ncRNA gene
Ensembl Bos taurus version 56.4e (Btau 4.0) Chromosome 7: 4,721,794 - 20,112,949
Gene Legend
Novel pseudogene
Known protein coding
Known RNA gene
Projected protein coding
Novel protein coding
Novel RNA gene

B

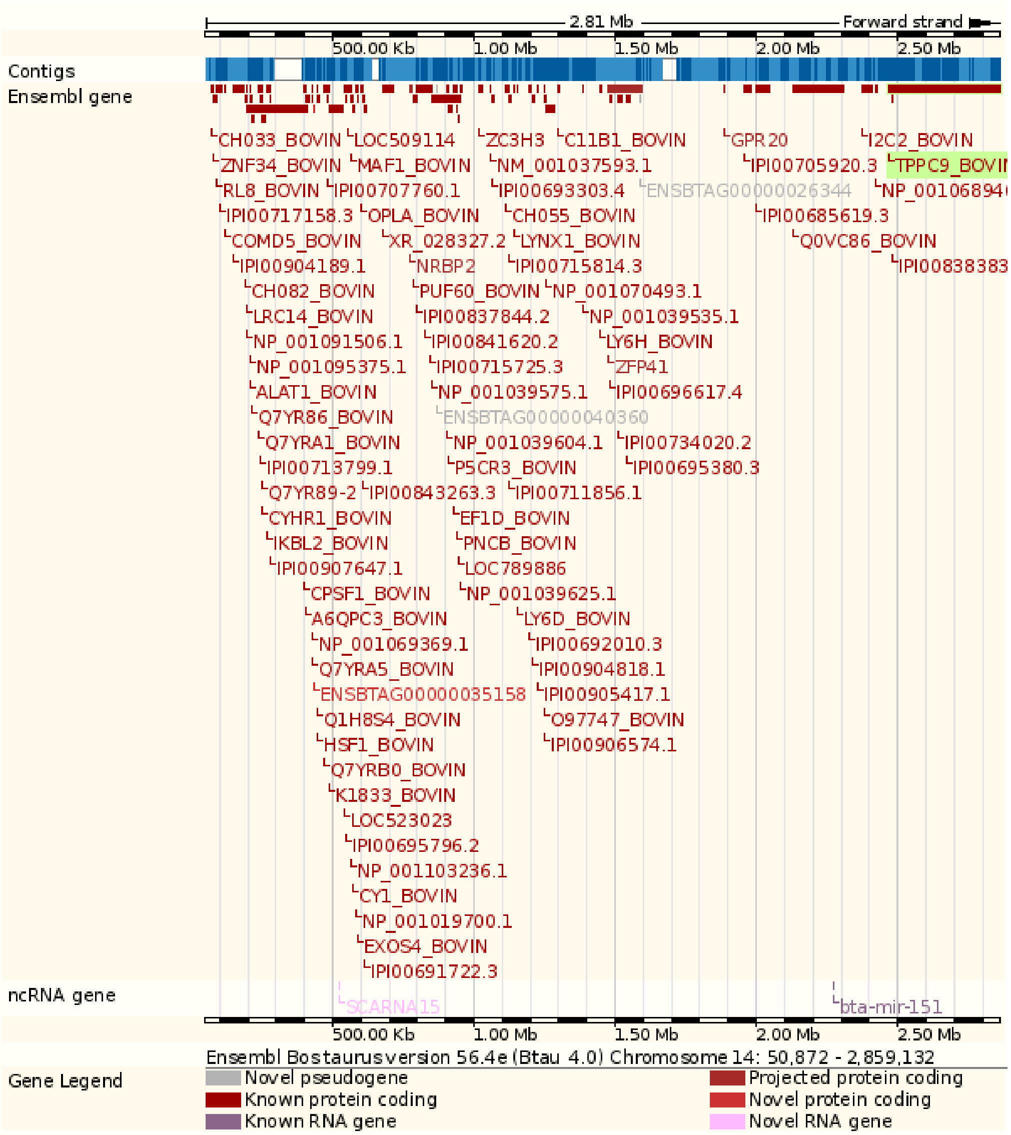
2.81 Mb
Forward strand
500.00 Kb
1.00 Mb
1.50 Mb
2.00 Mb
2.50 Mb
Contigs
Ensembl gene
ncRNA gene
SCARNA15
bta-mir-151
Ensembl Bostaurus version 56.4e (Btau 4.0) Chromosome 14: 50,872 - 2,859,132
Gene Legend
Novel pseudogene
Known protein coding
Known RNA gene
Projected protein coding
Novel protein coding
Novel RNA gene

C

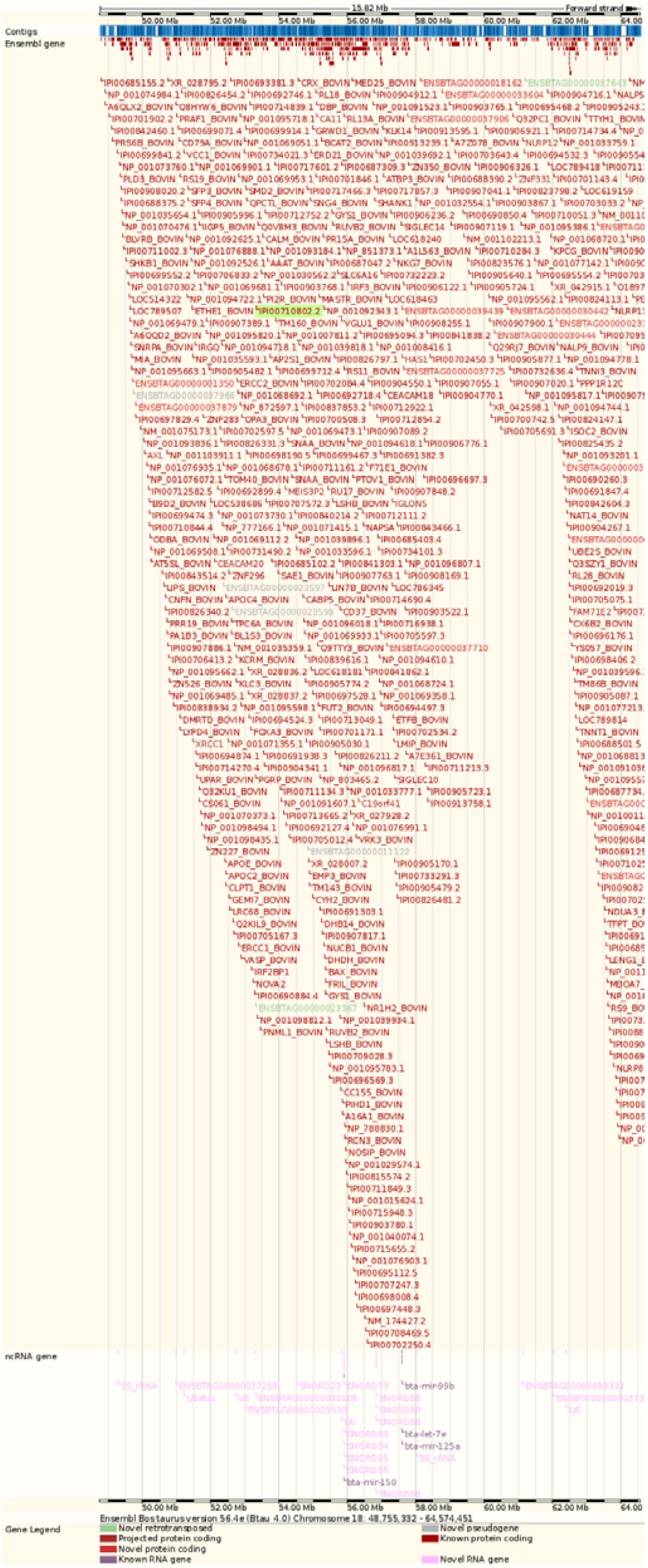

15.82 Mb
Forward strand
Contigs
Ensembl gene
ncRNA gene
Ensembl Bostaurus version 56.4e (Btau 4.0) Chromosome 18: 48,755,332 - 64,574,451
Gene Legend
Novel retrotransposed
Projected protein coding
Novel protein coding
Known RNA gene
Novel pseudogene
Known protein coding
Novel RNA gene

D

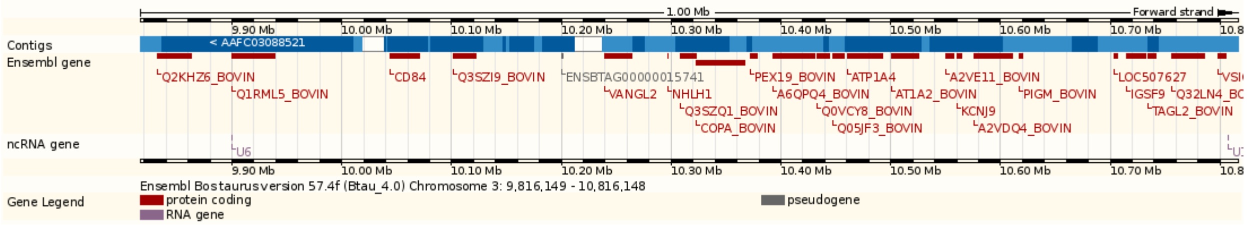
1.00 Mb
Forward strand
Contigs
< AAFC03088521
Ensembl gene
Q2KHZ6_BOVIN
Q1RML5_BOVIN
CD84
Q3SZI9_BOVIN
ENSBTAG00000015741
VANGL2
NHLH1
Q3SZQ1_BOVIN
COPA_BOVIN
PEX19_BOVIN
A6QPQ4_BOVIN
Q0VCY8_BOVIN
Q05JF3_BOVIN
ATP1A4
AT1A2_BOVIN
KCNJ9
A2VE11_BOVIN
A2VDQ4_BOVIN
PIGM_BOVIN
LOC507627
IGSF9
Q32LN4_BO
TAGL2_BOVIN
ncRNA gene
U6
9.90 Mb 10.00 Mb 10.10 Mb 10.20 Mb 10.30 Mb 10.40 Mb 10.50 Mb 10.60 Mb 10.70 Mb
Ensembl Bostaurus version 57.4f (Btau_4.0) Chromosome 3: 9,816,149 - 10,816,148
Gene Legend
protein coding
pseudogene
RNA gene

E

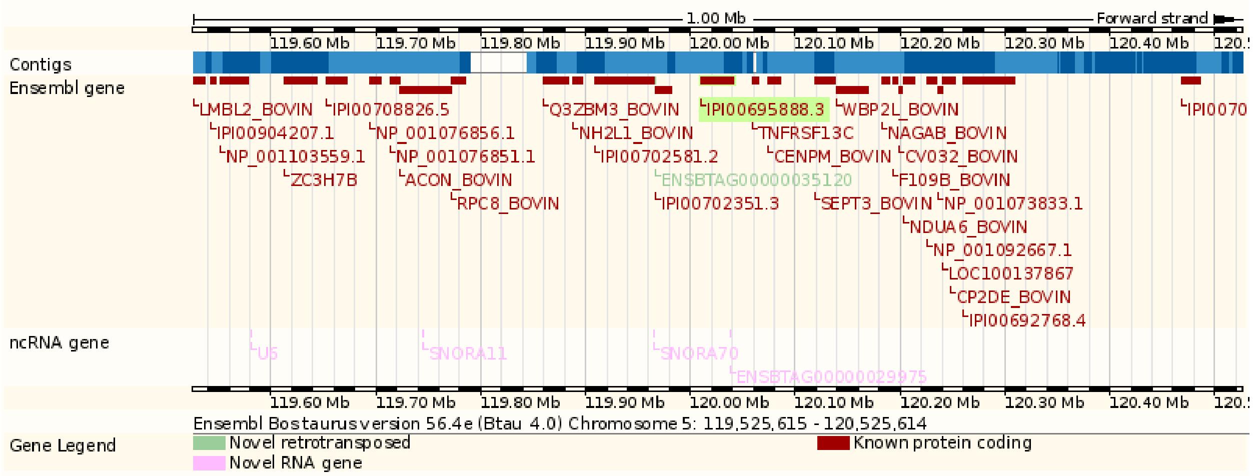
1.00 Mb
Forward strand
119.60 Mb 119.70 Mb 119.80 Mb 119.90 Mb 120.00 Mb 120.10 Mb 120.20 Mb 120.30 Mb 120.40 Mb
Contigs
Ensembl gene
LMBL2_BOVIN
IPI00904207.1
NP_001103559.1
ZC3H7B
IPI00708826.5
NP_001076856.1
NP_001076851.1
ACON_BOVIN
RPC8_BOVIN
Q3ZBM3_BOVIN
NH2L1_BOVIN
IPI00702581.2
ENSBTAG00000035120
IPI00702351.3
IPI00695888.3
TNFRSF13C
CENPM_BOVIN
SEPT3_BOVIN
WBP2L_BOVIN
NAGAB_BOVIN
CV032_BOVIN
F109B_BOVIN
NP_001073833.1
NDUA6_BOVIN
NP_001092667.1
LOC100137867
CP2DE_BOVIN
IPI00692768.4
ncRNA gene
U6
SNORA11
SNORA70
ENSBTAG00000029975
Ensembl Bostaurus version 56.4e (Btau 4.0) Chromosome 5: 119,525,615 - 120,525,614
Gene Legend
Novel retrotransposed
Known protein coding
Novel RNA gene

F

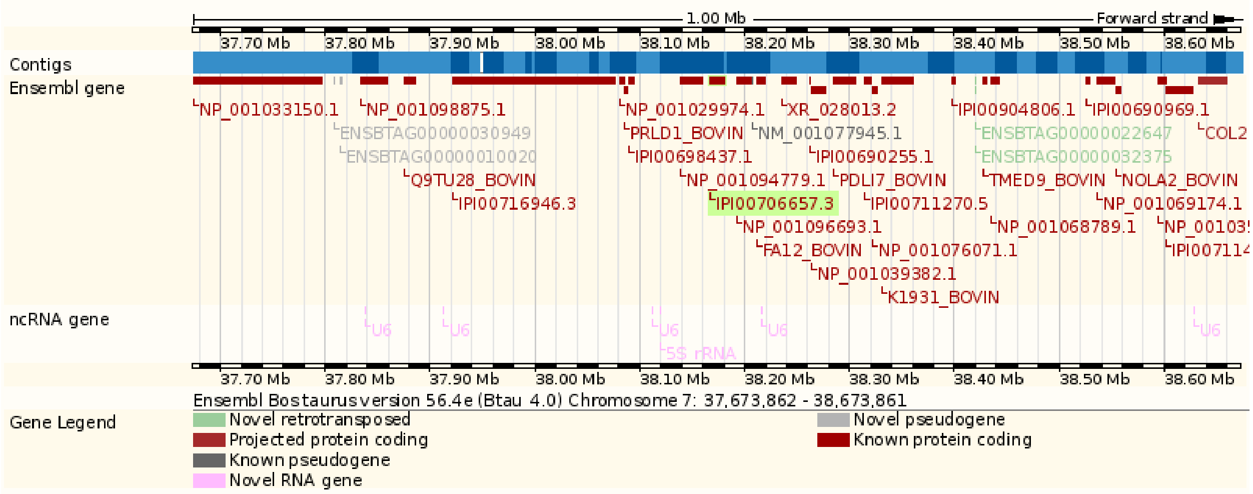
1.00 Mb
Forward strand
37.70 Mb 37.80 Mb 37.90 Mb 38.00 Mb 38.10 Mb 38.20 Mb 38.30 Mb 38.40 Mb 38.50 Mb 38.60 Mb
Contigs
Ensembl gene
NP_001033150.1
NP_001098875.1
ENSBTAG00000030949
ENSBTAG00000010020
Q9TU28_BOVIN
IPI00716946.3
NP_001029974.1
PRLD1_BOVIN
IPI00698437.1
NP_001094779.1
IPI00706657.3
NP_001096693.1
FA12_BOVIN
XR_028013.2
NM_001077945.1
IPI00690255.1
PDLI7_BOVIN
IPI00711270.5
NP_001076071.1
NP_001039382.1
K1931_BOVIN
IPI00904806.1
ENSBTAG00000022647
ENSBTAG00000032375
TMED9_BOVIN
NP_001068789.1
IPI00690969.1
NOLA2_BOVIN
NP_001069174.1
COL2
ncRNA gene
U6
5S_rRNA
Ensembl Bostaurus version 56.4e (Btau 4.0) Chromosome 7: 37,673,862 - 38,673,861
Gene Legend
Novel retrotransposed
Projected protein coding
Known pseudogene
Novel RNA gene
Novel pseudogene
Known protein coding

G

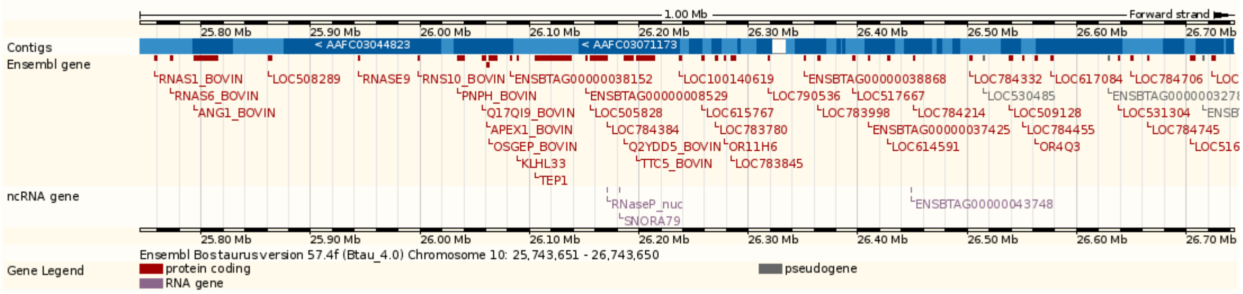
1.00 Mb
Forward strand
25.80 Mb
25.90 Mb
26.00 Mb
26.10 Mb
26.20 Mb
26.30 Mb
26.40 Mb
26.50 Mb
26.60 Mb
26.70 Mb
Contigs
< AAFC03044823
< AAFC03071173
Ensembl gene
RNAS1_BOVIN
RNAS6_BOVIN
ANG1_BOVIN
LOC508289
RNASE9
RNS10_BOVIN
PNPH_BOVIN
Q17QI9_BOVIN
APEX1_BOVIN
OSGEP_BOVIN
KLHL33
TEP1
ENSBTAG00000038152
ENSBTAG00000008529
LOC505828
LOC784384
Q2YDD5_BOVIN
TTC5_BOVIN
LOC100140619
LOC790536
LOC615767
LOC783780
OR11H6
LOC783845
ENSBTAG00000038868
LOC517667
LOC783998
ENSBTAG00000037425
LOC614591
LOC784332
LOC530485
LOC784214
LOC509128
LOC784455
OR4Q3
LOC617084
LOC784706
LOC531304
LOC784745
ncRNA gene
RNaseP_nuc
SNORA79
ENSBTAG00000043748
Ensembl Bostaurus version 57.4f (Btau_4.0) Chromosome 10: 25,743,651 - 26,743,650
Gene Legend
protein coding
RNA gene
pseudogene

H

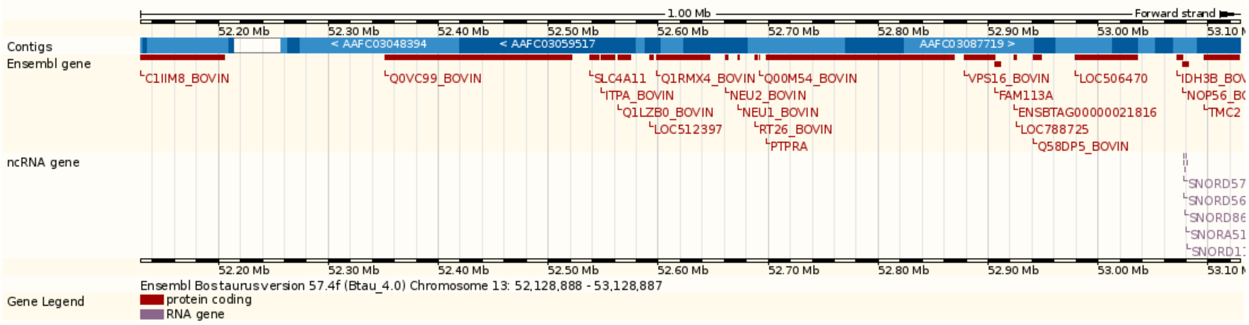
1.00 Mb
Forward strand
52.20 Mb
52.30 Mb
52.40 Mb
52.50 Mb
52.60 Mb
52.70 Mb
52.80 Mb
52.90 Mb
53.00 Mb
Contigs
< AAFC03048394
< AAFC03059517
AAFC03087719 >
Ensembl gene
C1IIM8_BOVIN
Q0VC99_BOVIN
SLC4A11
ITPA_BOVIN
Q1LZB0_BOVIN
LOC512397
Q1RMX4_BOVIN
NEU2_BOVIN
NEU1_BOVIN
RT26_BOVIN
PTPRA
Q00M54_BOVIN
VPS16_BOVIN
FAM113A
ENSBTAG00000021816
LOC788725
Q58DP5_BOVIN
LOC506470
TMC2
ncRNA gene
Ensembl Bostaurus version 57.4f (Btau_4.0) Chromosome 13: 52,128,888 - 53,128,887
Gene Legend
protein coding
RNA gene

I

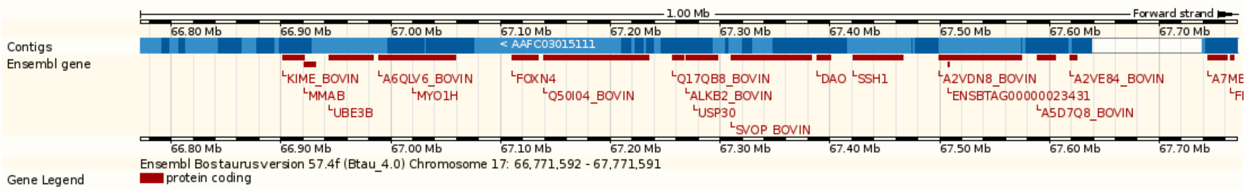
1.00 Mb
Forward strand
66.80 Mb
66.90 Mb
67.00 Mb
67.10 Mb
67.20 Mb
67.30 Mb
67.40 Mb
67.50 Mb
67.60 Mb
67.70 Mb
Contigs
< AAFC03015111
Ensembl gene
KIME_BOVIN
MMAB
UBE3B
A6QLV6_BOVIN
MYO1H
FOXN4
Q5I0I4_BOVIN
Q17QB8_BOVIN
ALKB2_BOVIN
USP30
SVOP_BOVIN
DAO
SSH1
A2VDN8_BOVIN
ENSBTAG00000023431
A2VE84_BOVIN
A5D7Q8_BOVIN
Ensembl Bostaurus version 57.4f (Btau_4.0) Chromosome 17: 66,771,592 - 67,771,591
Gene Legend
protein coding

J

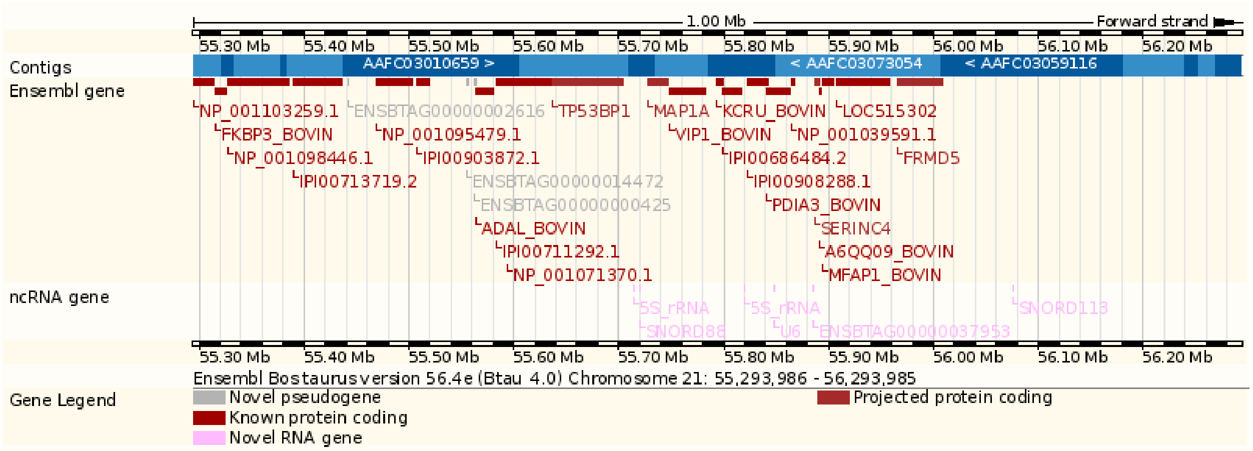
1.00 Mb
Forward strand
55.30 Mb
55.40 Mb
55.50 Mb
55.60 Mb
55.70 Mb
55.80 Mb
55.90 Mb
56.00 Mb
56.10 Mb
56.20 Mb
Contigs
AAFC03010659 >
< AAFC03073054
< AAFC03059116
Ensembl gene
NP_001103259.1
FKBP3_BOVIN
NP_001098446.1
IPI00713719.2
ENSBTAG00000002616
NP_001095479.1
IPI00903872.1
ENSBTAG00000014472
ENSBTAG00000000425
ADAL_BOVIN
IPI00711292.1
NP_001071370.1
TP53BP1
MAP1A
VIP1_BOVIN
KCRU_BOVIN
IPI00686484.2
IPI00908288.1
PDIA3_BOVIN
NP_001039591.1
SERINC4
A6QQ09_BOVIN
MFAP1_BOVIN
LOC515302
FRMD5
ncRNA gene
5S_rRNA
SNORD88
U6
ENSBTAG00000037953
SNORD113
Ensembl Bostaurus version 56.4e (Btau 4.0) Chromosome 21: 55,293,986 - 56,293,985
Gene Legend
Novel pseudogene
Known protein coding
Novel RNA gene
Projected protein coding

K

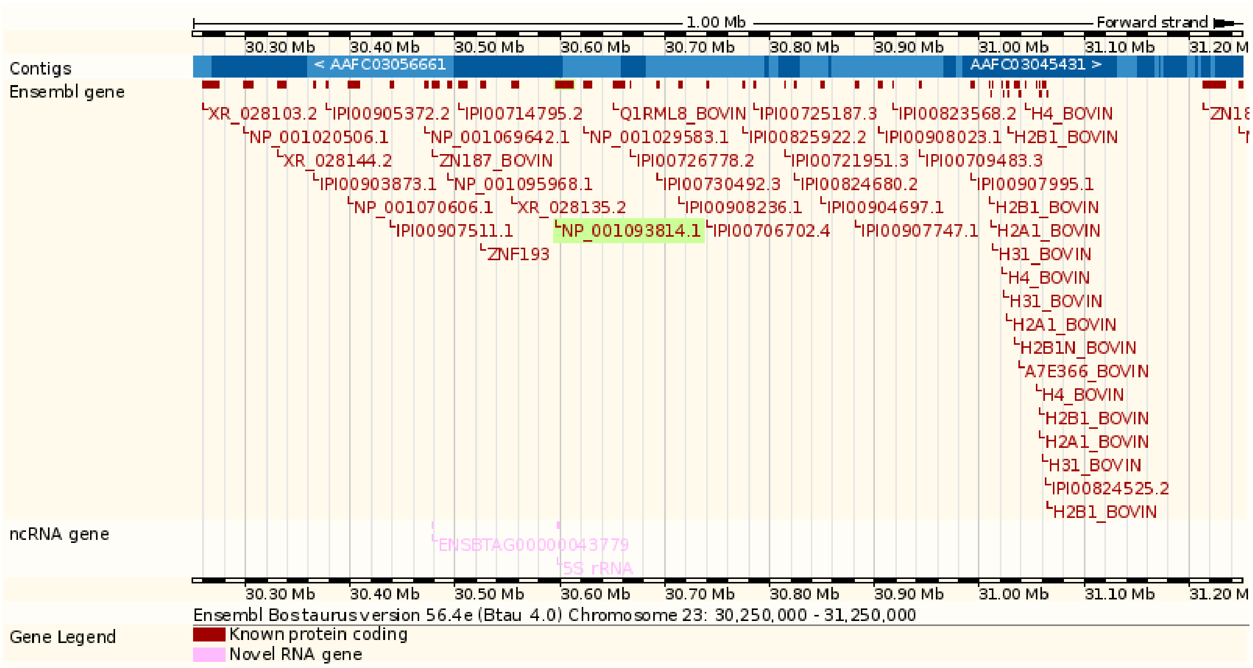
1.00 Mb
Forward strand
30.30 Mb
30.40 Mb
30.50 Mb
30.60 Mb
30.70 Mb
30.80 Mb
30.90 Mb
31.00 Mb
31.10 Mb
Contigs
< AAFC03056661
AAFC03045431 >
Ensembl gene
XR_028103.2
IPI00905372.2
IPI00714795.2
Q1RML8_BOVIN
IPI00725187.3
IPI00823568.2
H4_BOVIN
NP_001020506.1
NP_001069642.1
NP_001029583.1
IPI00825922.2
IPI00908023.1
H2B1_BOVIN
XR_028144.2
ZN187_BOVIN
IPI00726778.2
IPI00721951.3
IPI00709483.3
IPI00903873.1
NP_001095968.1
IPI00730492.3
IPI00824680.2
IPI00907995.1
NP_001070606.1
XR_028135.2
IPI00908236.1
IPI00904697.1
H2B1_BOVIN
IPI00907511.1
NP_001093814.1
IPI00706702.4
IPI00907747.1
H2A1_BOVIN
ZNF193
H31_BOVIN
H4_BOVIN
H31_BOVIN
H2A1_BOVIN
H2B1N_BOVIN
A7E366_BOVIN
H4_BOVIN
H2B1_BOVIN
H2A1_BOVIN
H31_BOVIN
IPI00824525.2
H2B1_BOVIN
ncRNA gene
ENSBTAG00000043779
5S_rRNA
Ensembl Bos taurus version 56.4e (Btau 4.0) Chromosome 23: 30,250,000 - 31,250,000
Gene Legend
Known protein coding
Novel RNA gene

L

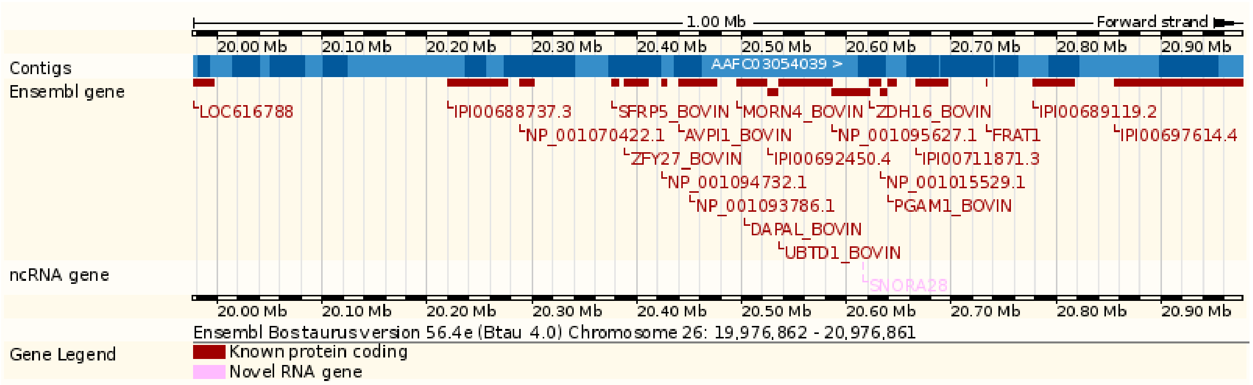
1.00 Mb
Forward strand
20.00 Mb
20.10 Mb
20.20 Mb
20.30 Mb
20.40 Mb
20.50 Mb
20.60 Mb
20.70 Mb
20.80 Mb
20.90 Mb
Contigs
AAFC03054039 >
Ensembl gene
LOC616788
IPI00688737.3
SFRP5_BOVIN
MORN4_BOVIN
ZDH16_BOVIN
IPI00689119.2
NP_001070422.1
AVPI1_BOVIN
NP_001095627.1
FRAT1
IPI00697614.4
ZFY27_BOVIN
IPI00692450.4
IPI00711871.3
NP_001094732.1
NP_001015529.1
NP_001093786.1
PGAM1_BOVIN
DAPAL_BOVIN
UBTD1_BOVIN
ncRNA gene
SNORA28
Ensembl Bos taurus version 56.4e (Btau 4.0) Chromosome 26: 19,976,862 - 20,976,861
Gene Legend
Known protein coding
Novel RNA gene

M

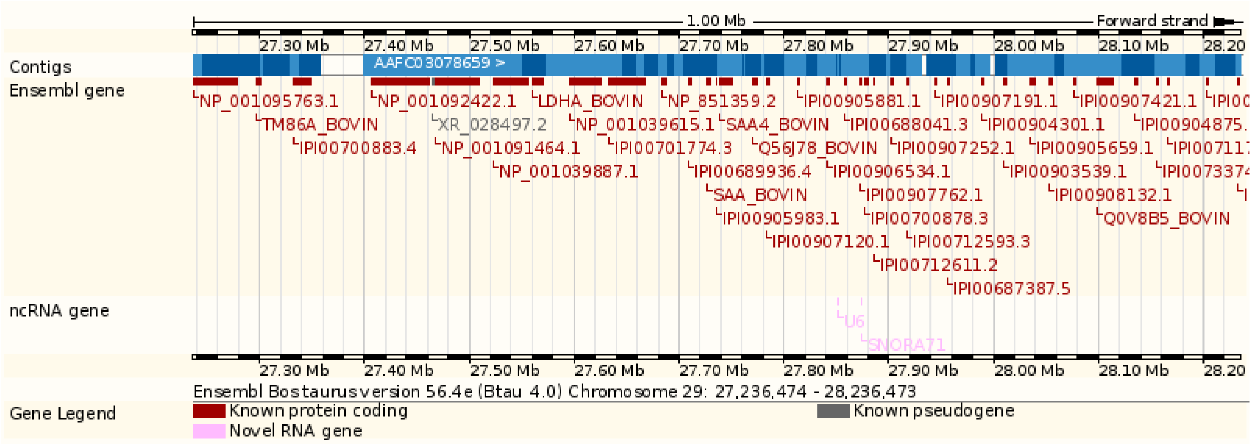
1.00 Mb
Forward strand
27.30 Mb
27.40 Mb
27.50 Mb
27.60 Mb
27.70 Mb
27.80 Mb
27.90 Mb
28.00 Mb
28.10 Mb
Contigs
AAFC03078659 >
Ensembl gene
NP_001095763.1
TM86A_BOVIN
IPI00700883.4
NP_001092422.1
XR_028497.2
NP_001091464.1
NP_001039887.1
LDHA_BOVIN
NP_001039615.1
IPI00701774.3
NP_851359.2
SAA4_BOVIN
Q56J78_BOVIN
IPI00689936.4
SAA_BOVIN
IPI00905983.1
IPI00907120.1
IPI00905881.1
IPI00688041.3
IPI00907252.1
IPI00906534.1
IPI00907762.1
IPI00700878.3
IPI00712593.3
IPI00712611.2
IPI00687387.5
IPI00907191.1
IPI00904301.1
IPI00905659.1
IPI00903539.1
IPI00908132.1
Q0V8B5_BOVIN
IPI00907421.1
ncRNA gene
U6
SNORA71
Ensembl Bostaurus version 56.4e (Btau 4.0) Chromosome 29: 27,236,474 - 28,236,473
Gene Legend
Known protein coding
Known pseudogene
Novel RNA gene

N

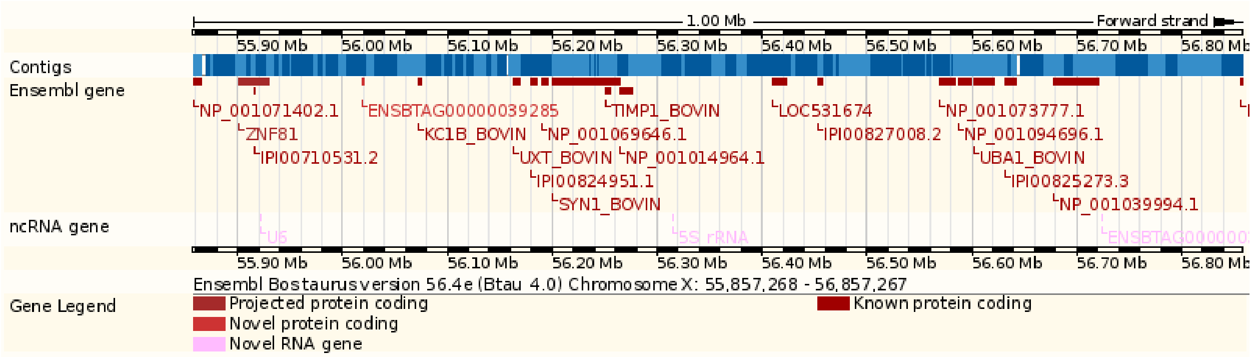
1.00 Mb
Forward strand
55.90 Mb
56.00 Mb
56.10 Mb
56.20 Mb
56.30 Mb
56.40 Mb
56.50 Mb
56.60 Mb
56.70 Mb
Contigs
Ensembl gene
NP_001071402.1
ZNF81
IPI00710531.2
ENSBTAG00000039285
KC1B_BOVIN
UXT_BOVIN
IPI00824951.1
SYN1_BOVIN
NP_001069646.1
NP_001014964.1
TIMP1_BOVIN
LOC531674
IPI00827008.2
NP_001073777.1
NP_001094696.1
UBA1_BOVIN
IPI00825273.3
NP_001039994.1
ncRNA gene
U6
5S_rRNA
Ensembl Bostaurus version 56.4e (Btau 4.0) Chromosome X: 55,857,268 - 56,857,267
Gene Legend
Projected protein coding
Known protein coding
Novel protein coding
Novel RNA gene
